# Structural Effects on the Hydride-Tunneling Kinetic Isotope Effects of NADH/NAD^+^ Model Reactions: Relating to the Donor–Acceptor Distances

**DOI:** 10.1021/acs.joc.4c03080

**Published:** 2025-02-13

**Authors:** Ava Austin, Jessica Sager, Lauren Phan, Yun Lu

**Affiliations:** Department of Chemistry, Southern Illinois University Edwardsville, Edwardsville, Illinois 62026, United States

## Abstract

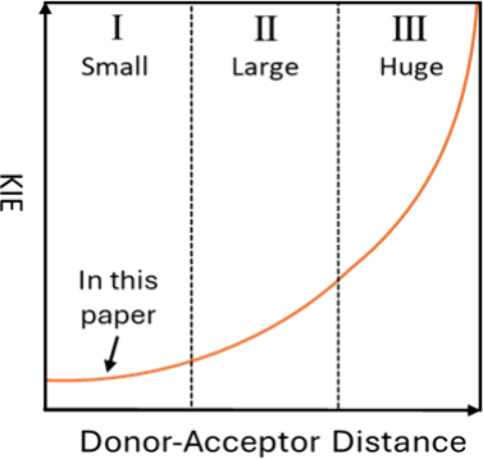

Contemporary H-tunneling theories predict that a longer donor–acceptor distance (DAD) corresponds to a larger kinetic isotope effect (KIE). Herein, hydride-tunneling reactions of NADH/NAD^+^ analogues in acetonitrile were used to examine the KIE–DAD relationship. Reaction pairs of similar tunneling-ready conformations were selected, so that additional factors influencing KIEs would be relatively fixed. Positive results were obtained, with some reaction pairs displaying a reversal of the traditional KIE−Δ*G°* relationship in favor of the KIE–DAD relationship, lending strong support to the latter.

Semiclassical transition state (TS) theory has traditionally been used to explain the kinetic isotope effects (KIEs) of H-transfer reactions. The semiclassical limits of H/D KIEs range from 2 to 7. Study of the structure–KIE relationship and its application in determining TS structures has a long history. In the early 1960s, Melander and Westheimer proposed that the maximum KIE should occur for a reaction in which the free energy change (Δ*G°*) equals zero and the TS is symmetric.^1,2^ Later, in 1980, Kresge derived a parabolic relationship linking isotope effects on activation free energies (Δ*G*^⧧^) to (Δ*G°*)^2^, based on the Marcus rate theory.^3^ Experiments supported the KIE−Δ*G°* relationship, showing that the KIE is maximal when Δ*G°* ∼ 0 and decreases for both exergonic and endergonic reactions.^3−7^ However, the structure–KIE relationship has not been theorized in the context of quantum-tunneling mechanisms, largely due to the lack of universally accepted theories for the complex H-tunneling processes.^8^

Bell was the first, in the 1960s−70s, to explain the maximum KIE at Δ*G°* ∼ 0 using his model that adds a tunnel correction to the one-dimensional energy barrier in the TS theory.^7^ In his explanation using the concept that tunneling magnifies KIEs, the tunneling probability is maximal at a symmetrical TS, making the highest KIE, and it decreases as the TS becomes reactant- or product-like. The Bell model was also used to explain the steric magnification of the KIEs of some proton-tunneling reactions.^9^ In that context, the steric effect increases the barrier, sharpening it near the TS, which enhances the tunneling efficiency.^9,10^ The model has, however, been critiqued as being oversimplified and not able to explain many subsequent observations.^8,11,12^

Since the 1980s, some research groups have studied the structure–KIE relationship for the hydride-tunneling reactions of NADH/NAD^+^ in enzymes and their analogues in solution.^5,13−18^ Kreevoy and co-workers studied the corresponding exergonic model reactions in solution and found that KIE, usually small (<6), increases when the hydride acceptor structures are modified to make the Δ*G°* less negative, whereas when the same happens to hydride donors, the KIE decreases.^14^ Klinman’s group found that KIE increases as the driving force of the reactions approaches zero in the endothermic hydride-tunneling reactions of yeast alcohol dehydrogenase.^18^ The inconsistent KIE−Δ*G°* relationship results were explained in terms of a mixed corner-cutting tunneling and over-the-barrier mechanisms, implying an uncertain relationship between structure and KIEs.

Contemporary H-tunneling models assume a longer donor–acceptor distance (DAD) for H-tunneling than tunneling of a heavier D nucleus whose vibrational wave function possesses a shorter de Broglie wavelength.^11,13,19−23^ H-Tunneling process can be ideally described as the overlap of the H-wave function (*S*_H_) inside the barrier between the reactant and product at a tunneling-ready state (TRS).^11,19−21,24^ Therefore, the KIE reflects the difference between *S*_H_ and *S*_D_, and because *S*_H_ is larger than *S*_D_, the KIE exceeds unity.^19^ For example, in the vibronic nonadiabatic H-tunneling model, the KIE is directly proportional to *S*_H_^2^/*S*_D_^2^ at the ground state level of a TRS.^19^ While the tunneling probability {*P*_tunneling_ [∝*S*_H(D)_]} decreases as the DAD increases, *S*_D_ decreases more rapidly than *S*_H_ (Figure 1, left panel), meaning that larger DADs are expected to yield larger KIEs, which become substantial as *S*_D_ approaches zero (Figure 1, right panel). This KIE–DAD relationship is intuitively correct, even if other tunneling models do not involve a straightforward mathematical relationship between the KIE and *S*_H_/*S*_D_.^21,25^

**Figure 1:**
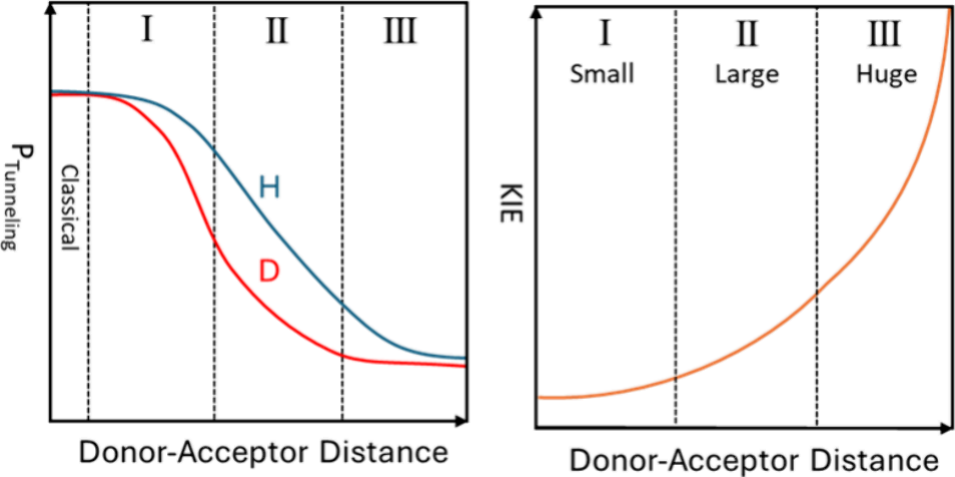
Schematic description of the predicted effect of DAD on the tunneling probability and KIE for a H-tunneling mechanism, assuming all other factors influencing KIEs remain constant.

However, structural changes produce different TRS structures and conformations and thus different “shapes and orientations” of wave functions, likely presenting an “irregular” KIE–DAD relationship. Furthermore, the KIE can be influenced by other factors, such as the different strength of system vibrations that modulate DADs and even the possible involvement of classical pathways to various extents.^14,16,19,22^ Therefore, direct experimental examination of the KIE–DAD relationship can be difficult. Nonetheless, some results have showcased the trend of increased KIEs with increasing DADs. Kreevoy, Truhlar, and co-workers computationally reproduced the aforementioned steric magnification of proton-tunneling KIEs by increasing DADs [KIEs up to 24^9,26^ (Figure 1, zones I and II)].^14^ Klinman and co-workers found that proton-coupled electron transfer in the soybean lipoxygenase reactions appeared to show an overall trend of an increased KIE (from 81 to 661^27−29^) with site-directed enzyme mutations that are strategically designed to increase DADs (zones II and III). Vibronic H-tunneling computation simulations of the KIEs by Hammes-Schiffer’s group confirmed the relationship.^30^ On the contrary, Scrutton, Hay, and co-workers found that the KIE (<6) indeed does not increase with the DAD caused by mutation in the hydride-transfer reaction mediated by the morphinone reductase, but they found that the vibrational frequency to sample DADs decreases with an increase in DAD (zone I).^22^ Together with their study of the effects of pressure on KIEs,^31^ they concluded that the decrease in vibrational frequency decreases the KIE, offsetting the DAD increase effect so that KIE does not change significantly with DAD. There have been many other hydride KIE studies in enzymes and mutants designed to vary DADs by the group of Kohen and the groups of others (<6, zone I),^20,32−38^ but the effects of the DAD on the size of KIEs were not directly discussed, possibly because the relationship is knowingly complicated by other factors due to the relatively large change in the TRS conformations caused by mutation.

We propose that comparing TRSs of similar conformations (and thus similar ways of wave function overlap) could help in the study of the KIE–DAD relationship. In this work, we use hydride-tunneling reactions of NADH/NAD^+^ analogues in acetonitrile for the study. The tunneling mechanisms of these structures in solution and NADH/NAD^+^ themselves in enzymes have been extensively studied in the literature,^5,11,16,19−22,39−41^ which include our study of the temperature dependence of hydride KIEs in solution.^24,42−48^ These reactions take place in charge-transfer (CT) complexes so that the TRS conformations could be managed to be similar and the π–π interactions and DADs could be mediated by structural variations.

Herein, we compare the hydride KIEs for reactions of two pairs of structurally similar hydride acceptors (NAD^+^ analogues). Pair I consists of 9-phenylxanthylium ion (PhXn^+^) versus 9-phenylthioxanthylium ion (PhTXn^+^), and pair II consists of PhXn^+^ versus 10-methyl-9-phenylacridinium ion (PhMA^+^) (Figure 2 top; counterions, BF_4_^–^). The two acceptor pairs differ only in O versus S as well as O versus N-CH_3_, which are far from the reaction center with the same distance. Therefore, the geometries and thus the vibrational modes of both pairs of the TRS structures are expected to be similar so that the effect of the DAD on KIEs could be studied.^44^ Due to the mismatching p orbitals between S and C, the positive charge is localized more at C-9 of the PhTXn^+^ than at PhXn^+^, and also due to the larger size of the p orbital in S, the π–π CT complexation is expected to be looser and the DAD to be longer with PhTXn^+^.^47^ In the pair II acceptors, because N is less electronegative so that the positive charge is stabilized, the CT complex is expected to be looser and the DAD to be longer with PhMA^+^ than with PhXn^+^.^47^ Therefore, the KIE is expected to be smaller for the reactions of PhXn^+^ compared to those of its S- and N-containing counterparts.

**Figure 2:**
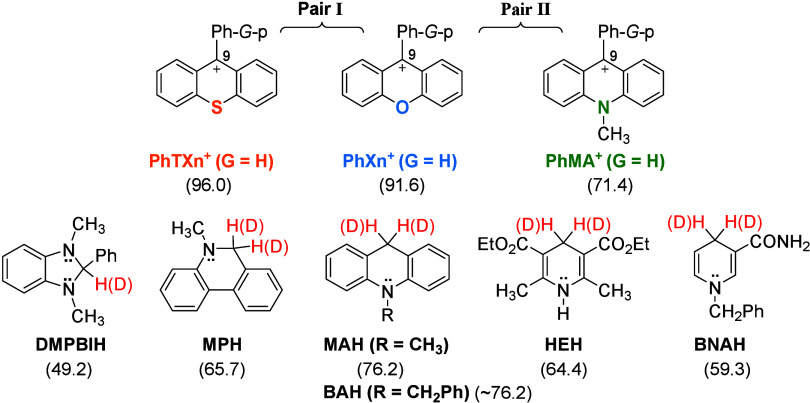
Hydride acceptors (top) and donors (bottom) and hydride affinities of their oxidized forms (−Δ*G°*_H^–^_, in kilocalories per mole^49^) in acetonitrile.

Note that PhXn^+^ is only 4.4 kcal/mol less reactive than PhTXn^+^ in acetonitrile (in contrast to 20.2 kcal/mol more reactive than PhMA^+^, from hydride affinity values in Figure 2(49)). According to the Δ*G°* difference, the reaction of PhXn^+^ would, however, have a KIE slightly larger than that of PhTXn^+^. Because, according to the DAD, the order of KIEs is expected to be the opposite, the systems could be especially used to confirm the DAD effect if a reversal of the classical KIE−Δ*G°* relationship is observed. Below, we will show this is indeed what we observed!

Six hydride donors (NADH analogues) were selected (Figure 2, bottom). They are 1,3-dimethyl-2-phenylbenzimidazoline (DMPBIH), 5-methyl-5,6-dihydrophenanthridine (MPH), 10-methylacridine (MAH), 10-benzylacridine (BAH), Hantzsch ester (HEH), and 4-benzyl-1,4-dihydronicotinamide (BNAH). The hydride affinity values (−Δ*G°*_H^–^_) of their oxidized forms in acetonitrile are listed in Figure 2 to derive the exothermicity (Δ*G°*) of the reactions. Selection of the substituted Me_2_NPhXn^+^/Me_2_NPhMA^+^ pair for comparison is due to the rate measurement limitations for the extremely fast reactions of PhXn^+^ with HEH and BNAH. Kinetics were carefully determined using the stopped-flow UV–vis spectroscopic method at temperatures across a 40 °C range. From both hydride- and deuteride-transfer rates, the isotopic activation energy differences (Δ*E*_a_ = *E*_aD_ – *E*_aH_) were derived, which reflect the temperature dependence of KIEs. The Δ*G°* values, second-order rate constants (*k*), and deuteride KIEs at 25 °C, as well as the Δ*E*_a_ values, are listed in Table 1.

**Table 1:** Δ*G°* Values, KIEs, and Their Temperature Dependences in Acetonitrile^a^

reaction systems	Δ*G°* (kcal/mol)	*k*_H_^25 °C^ (M^–1^ s^–1^)	KIE^25 °C^	Δ*E*_a_ (kcal/mol)
DMPBIH^b^				
-PhXn^+^	–42.4	4.54(0.05) × 10^4^	2.68 (0.04)	0.27 (0.06)
-PhTXn^+^	–46.8	1.66(0.02) × 10^5^	3.33 (0.05)	0.79 (0.12)
MPH				
-PhXn^+^	–25.9	3.74(0.02) × 10^3^	3.18 (0.03)	0.71 (0.05)
-PhTXn^+^	–30.3	1.31(0.01) × 10^4^	3.52 (0.04)	0.96 (0.03)
MAH^b^				
-PhXn^+^	–15.4	4.10(0.03) × 10^2^	4.08 (0.03)	0.88 (0.05)
-PhTXn^+^	–19.8	3.69(0.03) × 10^2^	4.79 (0.05)	1.08 (0.14)
BAH				
-PhXn^+^	∼−15.4	3.79(0.02) × 10^2^	4.26 (0.03)	0.89 (0.05)
-PhTXn^+^	∼−19.8	3.17(0.02) × 10^2^	4.83 (0.05)	1.04 (0.06)
HEH^b^				
-Me_2_NPhXn^+^	–22.3^c^	8.87(0.05) × 10^4^	3.56 (0.02)	0.86 (0.08)
-Me_2_NPhMA^+^	–3.0^c^	4.19(0.03) × 10	5.09 (0.06)	1.27 (0.14)
BNAH				
-Me_2_NPhXn^+^	–27.4^c^	5.62(0.04) × 10^4^	3.19 (0.03)	0.82 (0.04)
-Me_2_NPhMA^+^	–8.1^c^	8.37(0.08) × 10^–1^	4.79 (0.06)	1.14 (0.21)

a Numbers in parentheses are standard deviations.

b From ref (47).

c From −Δ*G°*_H^–^_ values of 86.7 and 67.4 kcal/mol for Me_2_NPhXn^+^ and Me_2_NPhMA^+^, respectively (Supporting Information).

All KIEs are <6. The Δ*E*_a_ values range from 0.27 to 1.27 kcal/mol, with some being outside of the semiclassical range of 1.0–1.2 kcal/mol. Although hydride-transfer reactions of NADH/NAD^+^ analogues typically exhibit small KIEs, our findings, along with other studies, indicate that these reactions display a broad Δ*E*_a_ range, extending from well below the semiclassical limit (∼0 kcal/mol) through the expected range to values well exceeding the limit (≲1.8 kcal/mol).^5,24,42,43,50^ Furthermore, it has been shown that small KIEs from such hydride-transfer reactions also align with the Marcus theory of atom transfer that incorporates a H-tunneling component.^13,16,17^ Additionally, small KIEs and similar Δ*E*_a_ values were also found in the hydride-tunneling reactions of NADH/NAD^+^ in enzymes.^22,25,41,51−54^ All of the observations have been explained by following the contemporary H-tunneling models.

The KIEs of the reactions of PhXn^+^ and Me_2_NPhXn^+^ are smaller than those of the reactions of PhTXn^+^ and Me_2_NPhMA^+^, respectively, at all temperatures (Table 1 and Figures S2–S4). The KIE difference in each pair of the reactions aligns with our expectations regarding the KIE–DAD relationship for a H-tunneling mechanism. An especially important finding is that in the PhXn^+^/PhTXn^+^ pairs of systems, the KIE is smaller for a less exergonic reaction of PhXn^+^ (less negative Δ*G°*), whereas for the Me_2_NPhXn^+^/Me_2_NPhMA^+^ reactions, the opposite is observed; i.e., the KIE is smaller for a more exergonic reaction of Me_2_NPhXn^+^ (more negative Δ*G°*). These results do not consistently align with the classical KIE−Δ*G°* relationship, further suggesting a H-tunneling mechanism and highlighting the significance of factors beyond Δ*G°* in determining KIEs.

Hammett correlations for the reactions with 9-*para*-substituted (*G*) phenyl derivatives of three hydride acceptors (*G*PhXn^+^, *G*PhTXn^+^, and *G*PhMA^+^) were determined to provide electronic structures of the TRSs and thus the DAD information for a correlation to KIEs. The Hammett constants (ρ) for reaction rates (also from Figures S2–S4) are listed in Table 2. Via comparison with those for equilibrium constants [ρ(*K*_H^–^_)] representing the full hydride ion acceptance by the cations, the partial positive charges on the acceptor moieties at the TRSs are calculated [ξ = 1 – ρ/ρ(*K*_H^–^_)] (Table 2).

**Table 2:** Hammett Constants (*ρ*) and Estimated Charges (ξ) at the TRSs in Acetonitrile

reaction system	ρ	ρ(*K*_H^–^_)^a^	estimated charge at TRS (ξ)^b^	TRS
DMPBIH/*G*PhXn^+^	1.05^a^	4.59	+0.77 on PhXn	tight
DMPBIH/*G*PhTXn^+^	0.71^a^	6.12	+0.88 on PhTXn	loose
MPH/*G*PhXn^+^	0.76	4.59	+0.83 on PhXn	tight
MPH/*G*PhTXn^+^	0.41	6.12	+0.93 on PhTXn	loose
MAH/*G*PhXn^+^	0.96^a^	4.59	+0.79 on PhXn	tight
MAH/*G*PhTXn^+^	0.57^a^	6.12	+0.91 on PhTXn	loose
BAH/*G*PhXn^+^	0.93	4.59	+0.80 on PhXn	tight
BAH/*G*PhTXn^+^	0.44	6.12	+0.93 on PhTXn	loose
HEH/*G*PhXn^+^	2.15^a^	4.59	+0.53 on PhXn^c^	tight
HEH/*G*PhMA^+^	0.61^a^	5.93	+0.90 on PhMA^c^	loose
BNAH/*G*PhXn^+^	2.65	4.59	+0.42 on PhXn^c^	tight
BNAH/*G*PhMA^+^	0.66	5.93	+0.89 on PhMA^c^	loose

a From ref (47).

b See the text.

c For the assumed reactions with PhXn^+^/PhMA^+^ only.

Among all reactions, the Hammett plots for the *G*PhXn^+^ reactions show a slope that is steeper than those of the *G*PhTXn^+^ and *G*PhMA^+^ reactions. This reflects a greater loss of positive charge in the activation process for the reactions with PhXn^+^ and Me_2_NPhXn^+^ compared with those of their respective counterparts. It is known that C–H bond changes during the hydride-transfer process could alter charges on the TRS structures. However, by comparison of the Δ*G°* values of the pair II reactions of Me_2_NPhXn^+^ versus Me_2_NPhMA^+^, the more exergonic former reaction would feature an earlier TRS in terms of the C–H bond change, leading to less positive charge loss than in the latter reaction. This discrepancy indicates that the observed greater charge loss from the Me_2_NPhXn^+^ reactions mostly results from the tighter CT complexation in the TRS, strongly suggesting a shorter DAD in this reaction.

With regard to the pair I reactions of PhXn^+^/PhTXn^+^, the observed charge loss is approximately double for PhXn^+^ compared to that for PhTXn^+^ (Table 2). Although this observed difference aligns with the C–H bond changes following Hammond’s postulate for the exergonic reactions, the Δ*G°* for the reaction of PhXn^+^ is only 4.4 kcal/mol less exergonic than for PhTXn^+^ and thus insufficient to account for the 2-fold difference in charge loss for these highly exergonic reactions. Consequently, the observed large difference in charge loss likely arises not only from the C–H bond changes during activation but also from differing CT complexations, with a tighter complex and a shorter DAD in the PhXn^+^ reaction than in the PhTXn^+^ reaction. Note that our recent report of computational simulation of the γ,γ-2CH_3_/2CD_3_ secondary KIEs on DMPBIH in reactions with Ph(T)Xn^+^ also reveals a shorter DAD in the PhXn^+^ reaction than in the PhTXn^+^ reaction.^55^

Furthermore, Table 1 shows that the Δ*E*_a_ values for reactions involving *G*PhXn^+^ are consistently and significantly smaller than their counterparts in all of the reaction pairs. Recent findings from studies of the H-transfer reactions, including the hydride-tunneling reactions of NADH/NAD^+^ in enzymes^11,20,22,25,53,54,56^ and their analogues in solution,^42−48^ have shown that smaller Δ*E*_a_ values correlate with densely populated shorter DADs facilitated by stronger heavy atom vibrations. Thus, the observed Δ*E*_a_ differences also imply shorter DADs in more tightly associated *G*PhXn^+^ systems across all of the reaction pairs. Notably, we have reported such correlations of Δ*E*_a_ values to DADs for some systems in Table 1.^47^

The analyses presented above indicate a tighter TRS for the *G*PhXn^+^ systems, with smaller KIEs, in each pair of reactions, supporting the KIE–DAD relationship. One possibility that we have not discussed is the assumed effect of DAD sampling from heavy atom vibrations on the KIEs. As noted above, stronger vibrations would produce a larger KIE, meaning that a tighter TRS would give rise to a larger KIE.^22,31^ However, our results showed otherwise, suggesting that the DAD has a much stronger effect than the vibrational frequency on KIEs in these reactions.

On the other hand, the KIE−Δ*G°* relationship appears to hold for all but the PhTXn^+^ systems [the less negative the Δ*G°*, the larger the KIE (Table 1 and Figure S5)], but it is also possible that the KIE–DAD relationship holds across all reactions including the PhTXn^+^ systems. In the latter case, it is conceivable that a less negative Δ*G°* from a weaker donor/acceptor pair gives rise to a looser TRS with a longer DAD.^43^ We acknowledge that proving the KIE–DAD relationship across all reactions together is challenging, as additional factors from very different systems may influence and complicate the observed trend.

In summary, we have investigated the relationship between the KIE and DAD in six pairs of hydride-tunneling reactions of PhXn^+^/PhTXn^+^ and Me_2_NPhXn^+^/Me_2_NPhMA^+^ in acetonitrile. In each reaction pair, the TRS conformations and ways of wave function overlaps are expected to be similar. In particular in the PhXn^+^/PhTXn^+^ reactions, the Δ*G°* values are close, minimizing their potential influence, if any, on the KIE differences and allowing the DAD effect to be more conclusively assessed. The predicted relationship suggests that a longer DAD corresponds to a larger KIE, and our findings strongly support it. Our results provide valuable insights for developing future theoretical frameworks and contribute to a better understanding of KIEs in both solution and enzymes.

## Data Availability

The data underlying this study are available in the published article and its Supporting Information.

## Supporting Information Available

Procedures for determining the rate constants, Arrhenius plots of KIEs, Hammett plots, KIE−Δ*G°* correlation, estimate of the −Δ*G°*_H^–^_ values for Me_2_NPhXn^+^ and Me_2_NPhMA^+^, and primary kinetic data (PDF)

## References

[ref1] Melander L. In Isotope Effects on Reaction Rates; Ronald Press: New York, 1960; pp 24–32.

[ref2] Westheimer F. H. 61, 265–73. THE mangitude of the Primary kinetic isotope effect for compounds of hydrogen and deuterium. Chem. Rev. 1961, 61, 265–273. 10.1021/cr60211a004.

[ref3] Kreevoy M. M.; Oh S.-W. Relation between rate and equilibrium constants for proton-transfer reactions. J. Am. Chem. Soc. 1973, 95, 4805–4810. 10.1021/ja00796a007.

[ref4] Bordwell F. G.; Boyle W. J. Kinetic Isotope Effects for Nitroalkanes and Their Relationship to Transition-State Structure in Proton-Transfer Reactions. J. Am. Chem. Soc. 1975, 97, 3447–3452. 10.1021/ja00845a028.

[ref5] Powell M. F.; Bruice T. C. Effect of isotope scrambling and tunneling on the kinetic and product isotope effects for reduced nicotinamide adenine dinucleotide model hydride transfer reactions. J. Am. Chem. Soc. 1983, 105, 7139–7149. 10.1021/ja00362a019.

[ref6] Anne A.; Fraoua S.; Hapiot P.; Moiroux J.; Saveant J.-M. Steric and Kinetic Isotope Effects in the Deprotonation of Cation Radicals of NADH Synthetic Analogs. J. Am. Chem. Soc. 1995, 117, 7412–7421. 10.1021/ja00133a014.

[ref7] Bell R. P. Liversidge lecture. Recent advances in the study of kinetic hydrogen isotope effects. Chem. Soc. Rev. 1974, 3, 513–544. 10.1039/cs9740300513.

[ref8] O’Ferrall R. A. M. Introduction to a symposium in print on tunnelling. J. Phys. Org. Chem. 2010, 23, 559–560. 10.1002/poc.1746.

[ref9] Lewis E. S.; Funderburk L. Rates and isotope effects in the proton transfers from 2-nitropropane to pyridine bases. J. Am. Chem. Soc. 1967, 89, 2322–2327. 10.1021/ja00986a013.

[ref10] Melander L.; Saunders W. H. Jr. In Reaction Rates of Isotopic Molecules, 2nd ed.; Wiley: New York, 1980.

[ref11] Nagel Z. D.; Klinman J. P. Update 1 of: Tunneling and dynamics in enzymatic hydride transfer. Chem. Rev. 2010, 110, PR41–PR67. 10.1021/cr1001035. 21141912 PMC4067601

[ref12] Stojković V.; Kohen A. Enzymatic H-transfer: Quantum tunneling and coupled motion from kinetic isotope effects. Isr. J. Chem. 2009, 49, 163–173. 10.1560/IJC.49.2.163.

[ref13] Kreevoy M. M.; Ostovic D.; Truhlar D. G.; Garrett B. C. Phenomenological manifestations of large-curvature tunneling in hydride-transfer reactions. J. Phys. Chem. 1986, 90, 3766–3774. 10.1021/j100407a052.

[ref14] Kim Y.; Truhlar D. G.; Kreevoy M. M. An experimentally based family of potential energy surfaces for hydride transfer between NAD+ analogs. J. Am. Chem. Soc. 1991, 113, 7837–7847. 10.1021/ja00021a002.

[ref15] Lee I.-S. H.; Jeoung E. H.; Kreevoy M. M. Marcus Theory of a Parallel Effect on R for Hydride Transfer Reaction between NAD+ Analogues. J. Am. Chem. Soc. 1997, 119, 2722–2728. 10.1021/ja963768l.

[ref16] Han Lee I.-S.; Jeoung E. H.; Kreevoy M. M. Primary Kinetic Isotope Effects on Hydride Transfer from 1,3-Dimethyl-2-phenylbenzimidazoline to NAD+ Analogues. J. Am. Chem. Soc. 2001, 123, 7492–7496. 10.1021/ja004232+. 11480968

[ref17] Kil H. J.; Lee I.-S. H. Primary Kinetic Isotope Effects on Hydride Transfer from Heterocyclic Compounds to NAD + Analogues. J. Phs. Chem. A 2009, 113, 10704–10709. 10.1021/jp905937x. 19746950

[ref18] Rucker J.; Cha Y.; Jonsson T.; Grant K. L.; Klinman J. P. Role of internal thermodynamics in determining hydrogen tunneling in enzyme-catalyzed hydrogen transfer reactions. Biochemistry 1992, 31, 11489–11499. 10.1021/bi00161a030. 1445883

[ref19] Layfield J. P.; Hammes-Schiffer S. Hydrogen Tunneling in Enzymes and Biomimetic Models. Chem. Rev. 2014, 114, 3466–3494. 10.1021/cr400400p. 24359189 PMC3981923

[ref20] Kohen A. Role of Dynamics in Enzyme Catalysis: Substantial vs. Semantic Controversies. Acc. Chem. Res. 2015, 48, 466–473. 10.1021/ar500322s. 25539442 PMC4334245

[ref21] Truhlar G. D. Tunneling in enzymatic and nonenzymatic hydrogen transfer reactions. J. Phy. Org. Chem. 2010, 23, 660–676. 10.1002/poc.1676.

[ref22] Pudney C. R.; Johannissen L.; Sutcliffe M. J.; Hay S.; Scrutton N. S. Direct Analysis of Donor-Acceptor Distance and Relationship to Isotope Effects and the Force Constant for Barrier Compression in Enzymatic H-Tunneling Reactions. J. Am. Chem. Soc. 2010, 132, 11329–11335. 10.1021/ja1048048. 20698699

[ref23] Klinman J. P. A new model for the origin of kinetic hydrogen isotope effects. J. Phy. Org. Chem. 2010, 23, 606–612. 10.1002/poc.1661.

[ref24] Liu Q.; Zhao Y.; Hammann B.; Eilers J.; Lu Y.; Kohen A. A Model Reaction Assesses Contribution of H-Tunneling and Coupled Motions to Enzyme Catalysis. J. Org. Chem. 2012, 77, 6825–6833. 10.1021/jo300879r. 22834675

[ref25] Roston D.; Cheatum C. M.; Kohen A. Hydrogen Donor-Acceptor Fluctuations from Kinetic Isotope Effects: A Phenomenological Model. Biochemistry 2012, 51, 6860–6870. 10.1021/bi300613e. 22857146 PMC3448806

[ref26] Lewis E. S.; Allen J. D. Ionization of nitroalkanes by substituted pyridines. J. Am. Chem. Soc. 1964, 86, 2022–2024. 10.1021/ja01064a023.

[ref27] Meyer M. P.; Tomchick D. R.; Klinman J. P. Enzyme structure and dynamics affect hydrogen tunneling: The impact of a remote side chain (I553) in soybean lipoxygenase-1. Proc. Nat. Acad. Sci. U.S.A. 2008, 105, 1146–1151. 10.1073/pnas.0710643105. PMC2234106 18216254

[ref28] Hu S.; Soudackov A. V.; Hammes-Schiffer S.; Klinman J. P. Enhanced Rigidification within a Double Mutant of Soybean Lipoxygenase Provides Experimental Support for Vibronically Nonadiabatic Proton-Coupled Electron Transfer Models. ACS Catal. 2017, 7, 3569–3574. 10.1021/acscatal.7b00688. 29250456 PMC5724529

[ref29] Hu S.; Offenbacher A. R.; Thompson E. M.; Gee C. L.; Wilcoxen J.; Carr C. A. M.; Prigozhin D. M.; Yang V.; Alber T.; Britt R. D.; Fraser J. S.; Klinman J. P. Biophysical Characterization of a Disabled Double Mutant of Soybean Lipoxygenase: The “Undoing” of Precise Substrate Positioning Relative to Metal Cofactor and an Identified Dynamical Network. J. Am. Chem. Soc. 2019, 141, 1555–1567. 10.1021/jacs.8b10992. 30645119 PMC6353671

[ref30] Li P.; Soudackov A. V.; Hammes-Schiffer S. Fundamental insights into proton-coupled electron transfer in soybean lipoxygenase from quantum mechanical/molecular mechanics free energy simulations. J. Am. Chem. Soc. 2018, 140, 3068–3076. 10.1021/jacs.7b13642. 29392938 PMC5849423

[ref31] Hay S.; Sutcliffe M. J.; Scrutton N. S. Promoting motions in enzyme catalysis probed by pressure studies of kinetic isotope effects. Proc. Natl. Acad. Sci. U.S.A. 2007, 104, 507–512. 10.1073/pnas.0608408104. 17202258 PMC1766415

[ref32] Loveridge E. J.; Evans R. M.; Allemann R. K. Solvent Effects on Environmentally Coupled Hydrogen Tunnelling During Catalysis by Dihydrofolate Reductase from Thermotoga maritima. Chem.-Eur. J. 2008, 14 (34), 10782–10788. 10.1002/chem.200801804. 18924193

[ref33] Wang Z.; Singh P. N.; Czekster M. C.; Kohen A.; Schramm V. L. Protein Mass-Modulated Effects in the Catalytic Mechanism of Dihydrofolate Reductase: Beyond Promoting Vibrations. J. Am. Chem. Soc. 2014, 136, 8333–8341. 10.1021/ja501936d. 24820793 PMC4063187

[ref34] Geddes A.; Paul C. E.; Hay S.; Hollmann F.; Scrutton N. S. Donor–Acceptor Distance Sampling Enhances the Performance of “Better than Nature” Nicotinamide Coenzyme Biomimetics. J. Am. Chem. Soc. 2016, 138, 11089–11092. 10.1021/jacs.6b05625. 27552302

[ref35] Ranasinghe C.; Guo Q.; Sapienza P. J.; Lee A. L.; Quinn D. M.; Cheatum C. M.; Kohen A. Protein Mass Effects on Formate Dehydrogenase. J. Am. Chem. Soc. 2017, 139, 17405–17413. 10.1021/jacs.7b08359. 29083897 PMC5800309

[ref36] Howe G. W.; van der Donk W. A. Temperature-Independent Kinetic Isotope Effects as Evidence for a Marcus-like Model of Hydride Tunneling in Phosphite Dehydrogenase. Biochemistry 2019, 58, 4260–4268. 10.1021/acs.biochem.9b00732. 31535852 PMC6852621

[ref37] Ranasinghe C.; Pagano P.; Sapienza P. J.; Lee A. L.; Kohen A.; Cheatum C. M. Isotopic Labeling of Formate Dehydrogenase Perturbs the Protein Dynamics. J. Phys. Chem. B 2019, 123, 10403–10409. 10.1021/acs.jpcb.9b08426. 31696711

[ref38] Singh P.; Vandemeulebroucke A.; Li J.; Schulenburg C.; Fortunato G.; Kohen A.; Hilvert D.; Cheatum C. M. Evolution of the Chemical Step in Enzyme Catalysis. ACS Catal. 2021, 11, 6726–6732. 10.1021/acscatal.1c00442.

[ref39] Yasui S.; Ohno A. Model studies with nicotinamide derivatives. Bioorg. Chem. 1986, 14, 70–96. 10.1016/0045-2068(86)90019-2.

[ref40] Kim Y.; Kreevoy M. M. The experimental manifestations of corner-cutting tunneling. J. Am. Chem. Soc. 1992, 114, 7116–7123. 10.1021/ja00044a024.

[ref41] Schowen R. L. The Strengths and Weaknesses of Model Reactions for the Assesement if Tunneling in Enzymic Reactions. In Quantum tunnelling in enzyme catalyzed reactions; Allemann R., Scrutton N., Eds.; The Royal Society of Chemistry: London, 2009; Vol. 13, pp 291–313.

[ref42] Lu Y.; Wilhelm S.; Bai M.; Maness P.; Ma L. Replication of the Enzymatic Temperature Dependency of the Primary Hydride Kinetic Isotope Effects in Solution: Caused by the Protein Controlled Rigidity of the Donor-Acceptor Centers?. Biochemistry 2019, 58, 4035–4046. 10.1021/acs.biochem.9b00574. 31478638

[ref43] Maness P.; Koirala S.; Adhikari P.; Salimraftar N.; Lu Y. Substituent Effects on Temperature Dependence of Kinetic Isotope Effects in Hydride-Transfer Reactions of NADH/NAD+ Analogues in Solution: Reaction Center Rigidity Is the Key. Org. Lett. 2020, 22, 5963–5967. 10.1021/acs.orglett.0c02049. 32662653

[ref44] Bai M.; Koirala S.; Lu Y. Direct Correlation Between Donor-Acceptor Distance and Temperature Dependence of Kinetic Isotope Effects in Hydride-Tunneling Reactions of NADH/NAD+ Analogues. J. Org. Chem. 2021, 86, 7500–7507. 10.1021/acs.joc.1c00497. 34037396

[ref45] Adhikari P.; Song M.; Bai M.; Rijal P.; DeGroot N.; Lu Y. Solvent Effects on the Temperature Dependence of Hydride Kinetic Isotope Effects: Correlation to the Donor–Acceptor Distances. J. Phys. Chem. A 2022, 126, 7675–7686. 10.1021/acs.jpca.2c06065. 36228057

[ref46] Bai M.; Pratap R.; Salarvand S.; Lu Y. Correlation of Temperature Dependence of Hydride Kinetic Isotope Effects with Donor-Acceptor Distances in Two Solvents of Different Polarities. Org. Biol. Chem. 2023, 21, 5090–5097. 10.1039/D3OB00718A. PMC10339711 37278324

[ref47] Beach A.; Adhikari P.; Singh G.; Song M.; DeGroot N.; Lu Y. Structural Effects on the Temperature Dependence of Hydride Kinetic Isotope Effects of the NADH/NAD+ Model Reactions in Acetonitrile: Charge-Transfer Complex Tightness Is a Key. J. Org. Chem. 2024, 89, 3184–3193. 10.1021/acs.joc.3c02562. 38364859 PMC10913049

[ref48] Singh G.; Austin A.; Bai M.; Bradshaw J.; Hammann B. A.; Kabotso D. E. K.; Lu Y. Study of the Effects of Remote Heavy Group Vibrations on the Temperature Dependence of Hydride Kinetic Isotope Effects of the NADH/NAD+ Model Reactions. ACS Omega 2024, 9, 20593–20600. 10.1021/acsomega.4c02383. 38737086 PMC11080011

[ref49] Zhu X. Q.; Deng F. H.; Yang J. D.; Li X. T.; Chen Q.; Lei N. P.; Meng F. K.; Zhao X. P.; Han S. H.; Hao E. J.; Mu Y. Y. A classical but new kinetic equation for hydride transfer reactions. Org. Biomol. Chem. 2013, 11, 6071–6089. 10.1039/c3ob40831k. 23917398

[ref50] Lu Y.; Zhao Y.; Handoo K. L.; Parker V. D. Hydride-exchange reactions between NADH and NAD^+^ model compounds under non-steady-state conditions. Apparent and Real kinetic isotope effects. Org. Biomol. Chem. 2003, 1, 173–181. 10.1039/b208186e. 12929407

[ref51] Basran J.; Sutcliffe M. J.; Scrutton N. S. Deuterium Isotope Effects during Carbon–Hydrogen Bond Cleavage by Trimethylamine Dehydrogenase: Implications for mechanism and vibrationally assisted hydrogen tunneling in wild-type and mutant enzymes. J. Biol. Chem. 2001, 276, 24581–24587. 10.1074/jbc.M101178200. 11304539

[ref52] Wang Z.; Kohen A. Thymidylate synthase catalyzed H-transfers: Two chapters in one tale. J. Am. Chem. Soc. 2010, 132, 9820–9825. 10.1021/ja103010b. 20575541 PMC2912445

[ref53] Stojkoviç V.; Perissinotti L.; Willmer D.; Benkovic S.; Kohen A. Effects of the donor acceptor distance and dynamics on hydride tunneling in the dihydrofolate reductase catalyzed reaction. J. Am. Chem. Soc. 2012, 134, 1738–1745. 10.1021/ja209425w. 22171795 PMC4341912

[ref54] Pagano P.; Guo Q.; Ranasinghe C.; Schroeder E.; Robben K.; Häse F.; Ye H.; Wickersham K.; Aspuru-Guzik A.; Major D. T.; Gakhar L.; Kohen A.; Cheatum C. M. Oscillatory Active-Site Motions Correlate with Kinetic Isotope Effects in Formate Dehydrogenase. ACS Catal. 2019, 9, 11199–11206. 10.1021/acscatal.9b03345. 33996196 PMC8118594

[ref55] Bai M.; Singh G.; Lu Y. Rigidity Analysis of Hydride Tunneling Ready States from Secondary Kinetic Isotope Effects and Hammett Correlations: Relating to the Temperature Dependence of Kinetic Isotope Effects. J. Phys. Org. Chem. 2025, 38, e70002 10.1002/poc.70002. PMC12863401 41635746

[ref56] Klinman J. P.; Kohen A. Hydrogen Tunneling Links Protein Dynamics to Enzyme Catalysis. Annu. Rev. Biochem. 2013, 82, 471–496. 10.1146/annurev-biochem-051710-133623. 23746260 PMC4066974

