# Supplementary material for: Structural Effects on the Hydride-Tunneling Kinetic Isotope Effects of NADH/NAD+ Model Reactions: Relating to the Donor–Acceptor Distances
Source: J Org Chem. 2025 Feb 13;90(8):3110–5. doi: 10.1021/acs.joc.4c03080 (PMC11877500; doi:10.1021/acs.joc.4c03080)
Supplement: Supplementary file 1 — jo4c03080_si_001.pdf [file jo4c03080_si_001.pdf]

Supporting Information

Structural Effects on the Hydride-Tunneling Kinetic Isotope Effects of NADH/NAD+ Model Reactions: Relating to the Donor-Acceptor Distances

Ava Austin, Jessica Sager[†], Lauren Phan[†], Yun Lu*

Department of Chemistry, Southern Illinois University Edwardsville, Edwardsville, Illinois 62026, United States

yulu@siue.edu

Procedures…………. …………………………………………………………………………………… S2
Plots of temperature dependence of KIEs and Hammett correlations of reaction rates (Figures S2-4)…. S3
Plot of the correlation between ln(KIE) and ΔG° for the reactions except those of $PhTXn^+$ (Figure S5). S5
Raw rate constants (Tables S1-13) ………………..…....……………….……………………………………. S5
Estimate of the Hydride Affinities of Me_2NPhXn^+ and Me_2NPhMA^+………………..…....………...…. S8
References ……………………………………………………………………………………………..... S8
Data availability statement.…………………………………………………………………………….... S9

Procedures

Syntheses of the hydride donors of MPH, BAH and BNAH and their deuterated analogues, as well as the hydride acceptors of $PhXn^+BF_4^-$, $PhTXn^+BF_4^-$, $Me_2NPhXn^+BF_4^-$, $Me_2NPhMA^+BF_4^-$, as well as other substituted *G*$PhXn^+BF_4^-$ and *G*$PhTXn^+BF_4^-$, have been reported by our group.[1-3] These compounds were usually purified with 2-3 times (mostly 3 times) recrystallizations. NMR and melting point were used to characterize the structures. The deuterium content is generally > 98%, while BNAH-4,4-d,d contains 96%D by NMR.

Kinetic measurements followed the same procedures as reported in the recent papers of ours.[2-3] Here we provide more details about how the data is collected and fitted to derive a pseudo-first order rate constant (k^{pfo}). All of the rate constants in acetonitrile reported in the paper can be found in Tables S1 to S9 that include the temperature and concentration conditions.

The pseudo-first order kinetics were determined on the SF-61DX2 Hi-Tech KinetAsyst double-mixing stopped-flow instrument. At first, a rate constant is estimated according to our experience. The *Abs* - time data of the corresponding 10 - 15 half-lives is used to derive an initial k^{pfo} by fitting the 12 half-live data (99.99% completion of the reaction). The process is repeated many times by constantly changing the half-live time until a consistent k^{pfo} is obtained. Using this k^{pfo} value, we calculated the half-life time, and run the formal kinetic measurements for 13.5 half-lives for six times, with one minute time interval between runs to allow equilibration of temperature of the reaction solutions before injecting into the reaction cell in the stopped-flow instrument. Using the software in the computer interfaced to the instrument, we fit the 12 half-lives *Abs* - time data. For the Tables S6 and S9 results only, we collected and fitted 3 half-lives data to derive the rate constants due to very slow reactions. If a small spike of the absorbance change is observed at the very beginning due to mixing of the solutions, the initial data selected for the fit would exclude that part of the data. This initial data point is usually only within 1% of the reaction. Figure S1 shows one example as to how the fit looks like (R^2 value) and how the k^{pfo} is derived.

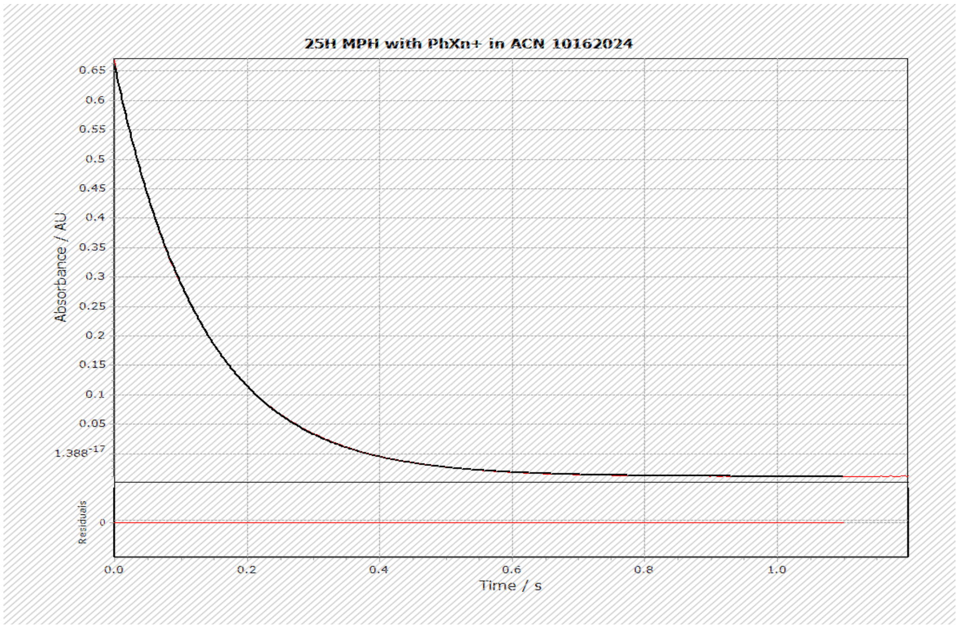

Figure S1. The fit to the averaged Abs - time data from six kinetic runs for the reaction between MPH (0.0020M) and $PhXn^+$ (0.0002M) in acetonitrile at 25°C at 473 nm (due to $PhXn^+$) following the first-order kinetic law (Definition: Y = -A*exp(-R*X) + C; R is k^{pfo}). This is copied from the Kinetic Studio report generated from the fitting software from the instrument (k^{pfo} = 7.57251 (s^{-1}), linear regression coefficient R^2 = 0.99996). The top red kinetic trace (with 13.5 $t_{1/2}$) is from the experiment, and the black curve (with 12.0 $t_{1/2}$) is the fit.

Six measurements of k^{pfo}'s for the reactions of two isotopologues for 1° KIE derivations at different temperatures were made on the same day and repeated on the other days. For a ΔE_a determination, kinetics was determined over a temperature range of 40 °C, and the E_{aH} and E_{aD} were derived, respectively. A typical kinetic procedure is as follows. Six kinetic runs of 13.5 half-lives of the reaction were measured for each isotopic reaction back-to-back. The procedure was then repeated at other temperatures as quickly as possible (*e.g.*, 5, 15, 25, 35, 45 °C, in order) so that the instrument settings were kept the same and the aging of the reaction solutions was minimal (While the solutions are already stable, they were wrapped with aluminum foil and kept in refrigerator between runs to eliminate any possible error source.). Repetitions of kinetic measurements sometimes used different batches of substrates and solvents, and sometimes were done by different workers. That was to eliminate the effect of possible different impurity from unknown sources or the human errors on the KIE measurements. Therefore, one KIE value was obtained from *at least* 18 repetitions (mostly 3 times (days) of measurements with 6 repetitions each time (day)). For the "very" slow reaction of BNAH with Me_2NPhMA^+, 9 repetitions were done (3 times (days) of measurements with 3 repetitions each time (day)). Pooled standard deviations were reported. All of the kinetic results (from the extent of reaction of close to 1% to 99.98% (corresponding to 12 half-lives)) were fitted very well/excellently to the first-order rate law for k^{pfo} derivation and to the Arrhenius correlations for E_a derivation, both with R^2 = 0.9990 – 1.0000, mostly closer to or sometimes even equal to 1.0000! Other details about the kinetic measurements, including the exceptions, as well as the raw data can be found from Tables S1 to S9.

<u>Plots of Temperature Dependence of KIEs and Hammett Correlations</u>

Note that we have reported the corresponding plots for the reactions of DMPBIH with $PhXn^+$ vs. $PhTXn^+$, BAH with $PhXn^+$ vs. $PhTXn^+$, and HEH with Me_2NPhXn^+ vs. Me_2NPhMA^+.[3] It should be emphasized that in the KIE – 1/T plots, at all temperatures, KIEs for the reactions of $PhTXn^+$ and Me_2NPhMA^+ are larger than those for $PhXn^+$ and Me_2NPhXn^+, respectively.

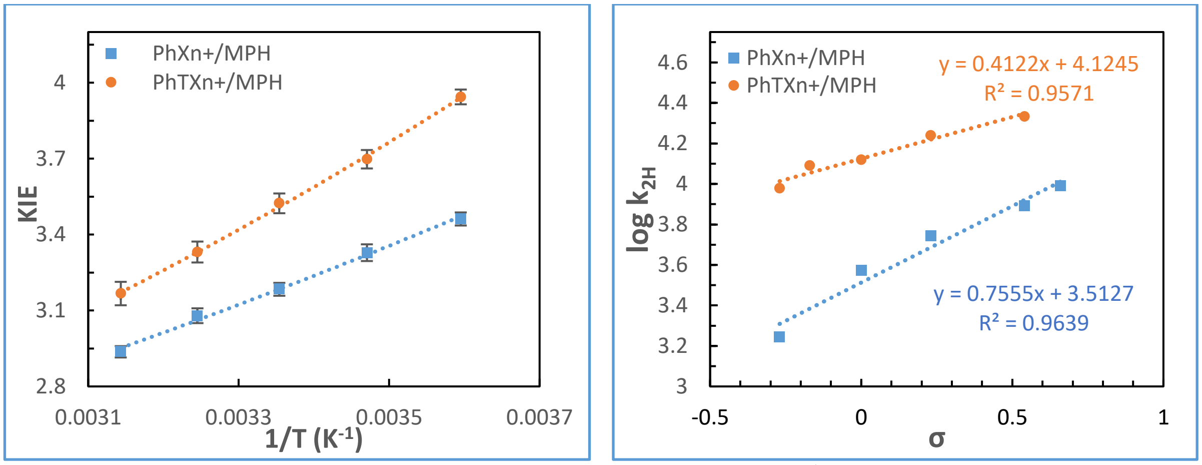

G$PhXn^+$: *G* = MeO, H, Br, CF_3, CN
G$PhTXn^+$: *G* = MeO, Me, H, Cl, CF_3

Figure S2. (Left) The Arrhenius plot of KIEs for hydride transfer reactions from MPH to Ph(T)Xn^+ (temperatures are 5, 15, 25, 35, and 45 °C, respectively; Lines are nonlinear exponential fit to the experiments); (Right) The linear Hammett correlations on *G*Ph(T)Xn^+ at 25 °C.

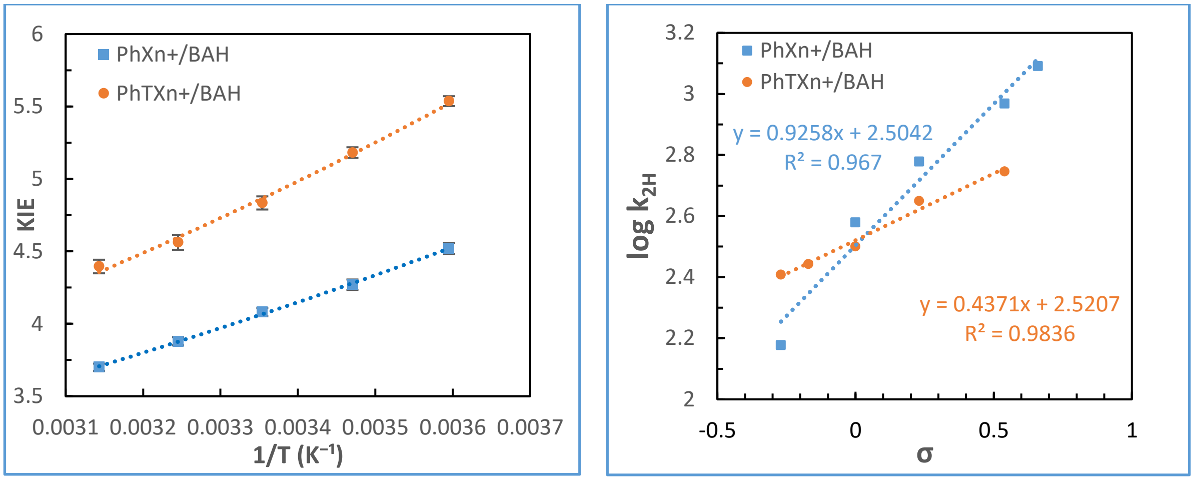

*G*PhXn$^+$: *G* = MeO, H, Br, CF_3, CN
*G*PhTXn$^+$: *G* = MeO, Me, H, Cl, CF_3

Figure S3. (Left) The Arrhenius plot of KIEs for hydride transfer reactions from BAH to Ph(T)Xn$^+$ (temperatures are 5, 15, 25, 35, and 45 ℃, respectively; Lines are nonlinear exponential fit to the experiments); (Right) The linear Hammett correlations on *G*Ph(T)Xn$^+$ at 25 ℃.

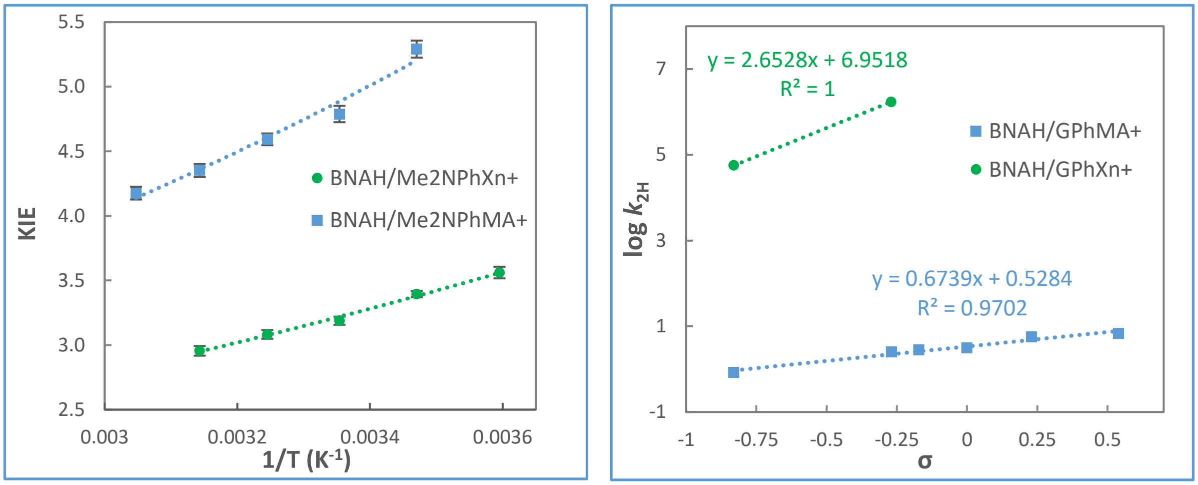

*G*PhMA$^+$: *G* = Me_2N, MeO, Me, H, Br, CF_3
*G*PhXn$^+$: *G* = Me_2N, MeO

Figure S4. (Left) The Arrhenius plot of KIEs for hydride transfer reactions from BNAH to Me_2NPhXn^+ and Me_2NPhMA^+ (left, temperatures are 5, 15, 25, 35, and 45 ℃ (or 15 – 55 ℃), respectively; Lines are nonlinear exponential fit to the experiments); (Right) The linear Hammett correlations on *G*PhXn$^+$ and *G*PhMA$^+$ at 25 ℃. Note that there are only two data points for the Hammett plot for the reactions of BNAH with *G*PhXn$^+$. This is because the rates of the other *G*PhXn$^+$ are beyond the instrument limit to determine the accurate rate constants. The same Hammett plot of the two data points was done for the reactions of HEH with *G*PhXn$^+$, which were already published by us.[3]

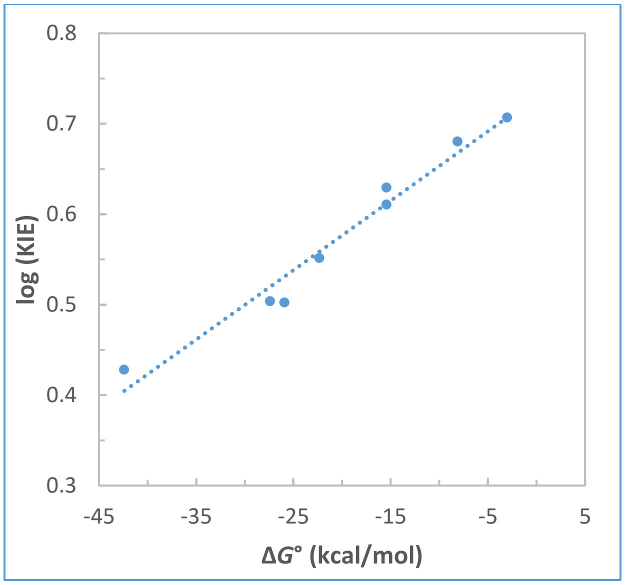

Figure S5. The log(KIE) – Δ*G*° correlation for the reactions of $PhXn^+$, Me_2NPhXn^+, and Me_2NPhMA^+ with various hydride donors in acetonitrile at 25 °C. Data points from left to right are for DMPBIH/$PhXn^+$, BNAH/Me_2NPhXn^+, MPH/$PhXn^+$, HEH/Me_2NPhXn^+, MAH/$PhXn^+$(and BAH/$PhXn^+$), BNAH/Me_2NPhMA^+, and HEH/Me_2NPhMA^+. Note that the $PhTXn^+$ reactions are not included. The KIE – DAD correlation of all reactions *including those of* $PhTXn^+$ cannot be plotted due to lack of the DAD data but appears to be plausible as discussed in the paper.

Raw Kinetic Data

Table S1. The temperature effects on the rate constants and 1° KIEs of the hydride transfer reaction from MPH to $PhXn^+$ in acetonitrile [a,b]

Temp (°C)	k_{2H} ($M^{-1}s^{-1}$)	k_{2D} ($M^{-1}s^{-1}$)	1° KIE
45.0	7.61(0.04) x 10^3	2.59(0.01) x 10^3	2.94 (0.02)
35.0	5.46(0.03) x 10^3	1.77(0.01) x 10^3	3.08 (0.03)
25.0	3.74(0.02) x 10^3	1.18(0.01) x 10^3	3.18 (0.03)
15.0	2.54(0.02) x 10^3	7.62(0.04) x 10^2	3.33 (0.03)
5.0	1.66(0.01) x 10^3	4.79(0.03) x 10^2	3.46 (0.03)

[a] Repeated on three different days with 6 repetitions each day. Numbers in parenthesis are the pooled standard deviations S(pooled). [b] [MPH] = 0.0020 M, [$PhXn^+$] = 0.00020 M. Absorbance decay at 473 nm due to $PhXn^+$ was followed for kinetic measurements.

Table S2. The temperature effects on the rate constants and 1° KIEs of the hydride transfer reaction from MPH to $PhTXn^+$ in acetonitrile [a,b]

Temp (°C)	k_{2H} ($M^{-1}s^{-1}$)	k_{2D} ($M^{-1}s^{-1}$)	1° KIE
45.0	2.22(0.03) x 10^4	7.02(0.05) x 10^3	3.17 (0.05)
35.0	1.71(0.02) x 10^4	5.14(0.03) x 10^3	3.33 (0.04)
25.0	1.31(0.01) x 10^4	3.73(0.03) x 10^3	3.52 (0.04)
15.0	9.74(0.06) x 10^3	2.63(0.02) x 10^3	3.70 (0.04)
5.0	7.11(0.04) x 10^3	1.80(0.01) x 10^3	3.94 (0.03)

[a] Repeated on three different days with 6 repetitions each day. Numbers in parenthesis are the pooled standard deviations S(pooled). [b] [MPH] = 0.0020 M, [$PhTXn^+$] = 0.00020 M. Absorbance decay at 493 nm due to $PhTXn^+$ was followed for kinetic measurements.

Table S3. The temperature effects on the rate constants and 1° KIEs of the hydride transfer reaction from BAH to $PhXn^+$ in acetonitrile [a,b]

**Temp (°C)**	$\boldsymbol{k_{2H}}$ **($M^{-1}s^{-1}$)**	$\boldsymbol{k_{2D}}$ **($M^{-1}s^{-1}$)**	**1° KIE**
45.0	8.13(0.06) x10^2	2.08(0.02) x10^2	3.90 (0.04)
35.0	5.64(0.04) x10^2	1.37(0.01) x10^2	4.12 (0.05)
25.0	3.79(0.02) x10^2	8.90(0.04) x10^1	4.26 (0.03)
15.0	2.50(0.02) x10^2	5.52(0.02) x10^1	4.53 (0.03)
5.0	1.60(0.01) x10^2	3.35(0.01) x10^1	4.79 (0.04)

[a] Repeated on three different days with 6 repetitions each day. Numbers in parenthesis are the pooled standard deviations S(pooled). [b] [BAH] = 0.0030 M, [$PhXn^+$] = 0.00020 M. Absorbance decay at 473 nm due to $PhXn^+$ was followed for kinetic measurements.

Table S4. The temperature effects on the rate constants and 1° KIEs of the hydride transfer reaction from BAH to $PhTXn^+$ in acetonitrile [a,b]

**Temp (°C)**	$\boldsymbol{k_{2H}}$ **($M^{-1}s^{-1}$)**	$\boldsymbol{k_{2D}}$ **($M^{-1}s^{-1}$)**	**1° KIE**
45.0	6.59(0.06) x 10^2	1.50(0.01) x 10^2	4.40 (0.05)
35.0	4.57(0.04) x 10^2	1.00(0.01) x 10^2	4.56 (0.05)
25.0	3.17(0.02) x 10^2	6.56(0.05) x 10^1	4.83 (0.05)
15.0	2.13(0.01) x 10^2	4.11(0.02) x 10^1	5.18 (0.04)
5.0	1.38(0.01) x 10^2	2.50(0.01) x 10^1	5.54 (0.03)

[a] Repeated on three different days with 6 repetitions each day. Numbers in parenthesis are the pooled standard deviations S(pooled). [b] [BAH] = 0.0030 M, [$PhTXn^+$] = 0.00020 M. Absorbance decay at 493 nm due to $PhTXn^+$ was followed for kinetic measurements.

Table S5. The temperature effects on the rate constants and 1° KIEs of the hydride transfer reaction from BNAH to Me_2NPhXn^+ in acetonitrile [a,b]

**Temp (°C)**	$\boldsymbol{k_{2H}}$ **($M^{-1}s^{-1}$)**	$\boldsymbol{k_{2D}}$ **($M^{-1}s^{-1}$)**	**1° KIE**
45.0	7.93(0.07) x 10^4	2.69(0.04) x 10^4	2.96 (0.03)
35.0	6.73(0.06) x 10^4	2.19(0.01) x 10^4	3.08 (0.03)
25.0	5.62(0.04) x 10^4	1.77(0.01) x 10^4	3.19 (0.03)
15.0	4.69(0.02) x 10^4	1.38(0.01) x 10^4	3.39 (0.02)
5.0	3.84(0.02) x 10^4	1.08(0.01) x 10^4	3.56 (0.05)

[a] Repeated on three different days with 6 repetitions each day. Numbers in parenthesis are the pooled standard deviations S(pooled). [b] [BNAH] = 4.87_5x10^{-4} M, [Me_2NPhXn^+] = 3.75x10^{-5} M. Absorbance decay 660 nm due to Me_2NPhXn^+ was followed for kinetic measurements.

Table S6. The temperature effects on the rate constants and 1° KIEs of the hydride transfer reaction from BNAH to Me_2NPhMA^+ in acetonitrile [a,b]

**Temp (°C)**	$\boldsymbol{k_{2H}}$ **($M^{-1}s^{-1}$)**	$\boldsymbol{k_{2D}}$ **($M^{-1}s^{-1}$)**	**1° KIE**
55.0	3.371(0.040)	0.821(0.003)	4.11 (0.05)
45.0	2.213(0.024)	0.509(0.002)	4.35 (0.05)
35.0	1.390(0.009)	0.303(0.002)	4.59 (0.05)
25.0	0.837(0.008)	0.175(0.002)	4.79 (0.06)
15.0	0.484(0.004)	0.092(0.001)	5.29 (0.07)

[a] Repeated on three different days with 3 repetitions each day. Numbers in parenthesis are the pooled standard deviations S(pooled). [b] [BNAH] = 0.0060 M, [Me_2NPhMA^+] = 0.00010 M. Absorbance decay 560 nm due to Me_2NPhMA^+ was followed for kinetic measurements.

Table S7. The rate constants (for Hammett correlations) of the hydride transfer reactions from MPH to *G*PhXn$^+$ and *G*PhTXn$^+$ in acetonitrile at 25 °C [a]

*G*PhXn$^+$	$k_{2H}^{25°C}$ ($M^{-1}s^{-1}$) [b]	*G*PhTXn$^+$	$k_{2H}^{25°C}$ ($M^{-1}s^{-1}$) [c]
MeO	1.75(0.01) x10^3	MeO	9.50(0.06) x10^3
H	3.74(0.02) x10^3	Me	1.23(0.01) x10^4
Br	5.54(0.04) x10^3	H	1.31(0.01) x10^4
CF_3	7.79(0.05) x10^3	Cl	1.74(0.01) x10^4
CN	9.74(0.06) x10^3	CF_3	2.15(0.01) x10^4

[a] Data was repeated on the same day with 24 repetitions using two batches of the donor (large excess) solutions except for the reactions of PhXn$^+$ and PhTXn$^+$ themselves that were repeated on three days with 6 repetitions each day (see Tables S1-S2). Numbers in parenthesis are the pooled standard deviations (S(pooled)). [b] [MPH] = 2.00x10^{-3} M with [MeOPhXn$^+$] = 6.23x10^{-5} M, [PhXn$^+$] = 2.00x10^{-4} M, [BrPhXn$^+$] = 2.00x10^{-4} M, [CF_3PhXn$^+$] = 2.00x10^{-4} M, [CNPhXn$^+$] = 2.00x10^{-4} M. Absorbance decay at 496, 473, 493, 480, 480 nm due to MeO-, H, Br-, CF_3-, and CN-substituted PhXn$^+$ were followed for kinetic measurements; [c] [MPH] = 2.00x10^{-3} M with [MeOPhTXn$^+$] = 1.00x10^{-4} M, [MePhTXn$^+$] = 1.25x10^{-4} M, [PhTXn$^+$] = 2.00x10^{-4} M, [ClPhTXn$^+$] = 1.50x10^{-4} M, [CF_3PhTXn$^+$] = 2.00x10^{-4} M. Absorbance decay at 530, 496, 493, 497, 496 nm due to MeO-, Me-, H, Cl-, and CF_3-substituted PhTXn$^+$ were followed for kinetic measurements.

Table S8. The rate constants of the hydride transfer reaction from BAH to *G*PhXn$^+$ and *G*PhTXn$^+$ in acetonitrile at 25 °C [a]

*G*PhXn$^+$	$k_{2H}^{25°C}$ ($M^{-1}s^{-1}$) [d]	*G*PhTXn$^+$	$k_{2H}^{25°C}$ ($M^{-1}s^{-1}$) [e]
MeO [b]	1.50(0.01) x 10^2	MeO	2.56(0.02) x 10^2
H [c]	3.79(0.02) x 10^2	Me	2.77(0.02) x 10^2
Br	6.01(0.04) x 10^2	H [c]	3.17(0.02) x 10^2
CF_3	9.30(0.06) x 10^2	Cl	4.46(0.03) x 10^2
CN	1.23(0.07) x 10^3	CF_3	5.58(0.07) x 10^2

[a] Numbers in parenthesis are the pooled standard deviations (S(pooled)). Repeated on one day with 24 repetitions, 12 repetitions for each donor solution; [b] repeated on two different days with 12 repetitions each day; [c] repeated on three different days with 6 repetitions each day for PhXn$^+$ and PhTXn$^+$ (see Tables S3-S4); [d] [BAH] = 2.00x10^{-3} M with [MeOPhXn$^+$] = 6.225x10^{-5} M, ([BAH] = 3.00x10^{-3} M for [PhXn$^+$] = 2.00x10^{-4} M), [BrPhXn$^+$] = 2.00x10^{-4} M, [CF_3PhXn$^+$] = 2.00x10^{-4} M, [CNPhXn$^+$] = 2.00x10^{-4} M. Absorbance decay at 496 nm, 473 nm, 493 nm, 480 nm, and 480 nm due to MeO-, H, Br-, CF_3-, and CN-substituted PhXn$^+$ was followed for kinetic measurements. [e] [BAH] = 2.00x10^{-3} M with [MeOPhTXn$^+$] = 1.00x10^{-4} M, [MePhTXn$^+$] = 1.25x10^{-4} M, ([BAH] = 3.00x10^{-3} M only for [PhTXn$^+$] = 2.00x10^{-4} M), [ClPhTXn$^+$] = 1.50x10^{-4} M, [CF_3PhTXn$^+$] = 2.00x10^{-4} M. Absorbance decay at 530 nm, 496 nm, 493 nm, 497 nm, and 496 nm due to MeO, Me, H, Cl, and CF3 substituted PhTXn$^+$ was followed for kinetic measurements.

Table S9. The rate constants of the hydride transfer reaction from BNAH to Me_2NPhXn$^+$ and Me_2NPhMA$^+$ in acetonitrile at 25 °C [a]

*G*PhXn$^+$	$k_{2H}^{25°C}$ ($M^{-1}s^{-1}$) [b]	*G*PhMA$^+$	$k_{2H}^{25°C}$ ($M^{-1}s^{-1}$) [c]
Me_2N	56.23(0.37) x 10^3	Me_2N	0.4837 (0.0040)
MeO	26.56(0.02) x 10^3	MeO	1.8300 (0.0003)
		Me	2.0892 (0.0010)
		H	2.2889 (0.0004)
		Br	4.3208 (0.0008)
		CF_3	5.1646 (0.0005)

[a] Repeated on three different days with 3 repetitions each day for MeOPhXn$^+$ and Me_2NPhMA$^+$, and 6 repetitions each day for all other *G*PhXn$^+$ and *G*PhMA$^+$. Numbers in parenthesis are the pooled standard deviations (S(pooled)). [b] [BNAH] = 4.87$_5$x10^{-4} M, [Me_2NPhXn$^+$] = 3.75x10^{-5} M; [BNAH] = 6.23x10^{-4}M, [MeOPhXn$^+$] = 6.23x10^{-5}M. Absorbance decay at 660 nm and 496 nm due to Me_2NPhXn$^+$ and MeOPhXn$^+$ was followed for kinetic measurements, respectively. [c] [BNAH] = 0.0060 M with [Me_2NPhMA$^+$] = 0.00010 M, [MeOPhMA$^+$] = 0.00010 M, [MePhMA$^+$] = 0.00015 M, [PhMA+] = 0.00015 M, [BrPhMA$^+$] = 0.00012 M, [CF_3PhMA$^+$] = 0.00015 M. Absorbance decay at 560 nm, 434 nm, 426 nm, 436 nm, 425 nm, and 424 nm due to Me_2N, MeO, Me, H, Br, and CF_3 substituted PhMA$^+$ was followed for kinetic measurements.

Estimate of the Hydride Affinities of Me_2NPhXn^+ and Me_2NPhMA^+

The hydride affinities ($-\Delta G°_{H^-}$) of the two cations have not been reported in literature. In this paper, we used the Hammett correlations of the $\log(K_{H^-})$ with substituent constants (σ), which we have reported for other *G*$PhXn^+$ and *G*$PhMA^+$ systems,[3] to estimate them. The K_{H^-} is the equilibrium constant for a cation to accept a hydride ion at 25 °C in acetonitrile. From the *SI* of the Ref. 3, we have the following mathematic relationship,

For the *G*$PhXn^+$ systems,

$$\log(K_{H^-}) = 4.5923 \bullet \sigma + 67.373$$ (from *G* = *p*-MeO, *p*-Me, H, *p*-Cl, *p*-Br, *m*-MeO, *m*-Me, *m*-CF_3)

For the *G*$PhMA^+$ systems,

$$\log(K_{H^-}) = 5.9261 \bullet \sigma + 54.292$$ (from *G* = *p*-MeO, *p*-Me, H, *p*-Cl, *p*-CF_3)

We extrapolated the linear relationships to the respective *p*-Me_2N-substituted cations (σ = -0.83) and calculated the $-\Delta G°_{H^-}$ values for the two cations at 25 °C.

References

1. Maharjan, B.; Raghibi Boroujeni, M.; Lefton, J.; White, O. R.; Razzaghi, M.; Hammann, B. A.; Derakhshani-Molayousefi, M.; Eilers, J. E.; Lu, Y., Steric Effects on the Primary Isotope Dependence of Secondary Kinetic Isotope Effects in Hydride Transfer Reactions in Solution: Caused by the Isotopically Different Tunneling Ready State Conformations? *J. Am. Chem. Soc.* **2015,** *137*, 6653 - 6661.
2. Singh, G.; Austin, A.; Bai, M.; Bradshaw, J.; Hammann, B. A.; Kabotso, D. E. K.; Lu, Y., Study of the Effects of Remote Heavy Group Vibrations on the Temperature Dependence of Hydride Kinetic Isotope Effects of the NADH/NAD+ Model Reactions. *ACS Omega* **2024,** *9* (18), 20593-20600.
3. Beach, A.; Adhikari, P.; Singh, G.; Song, M.; DeGroot, N.; Lu, Y., Structural Effects on the Temperature Dependence of Hydride Kinetic Isotope Effects of the NADH/NAD+ Model Reactions in Acetonitrile: Charge-Transfer Complex Tightness Is a Key. *J. Org. Chem* **2024,** *89* (5), 3184–3193.

Data Availability Statement

We present the primary kinetic data for all of the rate constants reported in the above Tables S1-S9. We directly copy the original data from the corresponding excel data file. Due to the decimal point place difference in between the two places, data may slightly differ at the last digit of their numbers. Meanwhile, we provide the *Abs* – time (t) data for the measurements of the pseudo first-order rate constants (k^{pfo}'s).

Primary kinetic data for the rate constants in Table S1

Day 1 data (October 16, 2024)

	Pseudo-first-order rate constants k^{pfo} (s^{-1})									
**Temp (°C)**	**Trial H1**	**Trial H2**	**Trial H3**	**Trial H4**	**Trial H5**	**Trial H6**	**Average k_H^{pfo} (s^{-1})**	**Stdev**	**k_{2H} (M^{-1}s^{-1})**	**Stdev [a]**
45	15.2749	15.2848	15.2889	15.4212	15.3697	15.3904	15.3383	0.0631	7.67E+03	31.5448
35	10.9109	10.9786	11.0365	10.9221	10.9198	11.0683	10.9727	0.0670	5.49E+03	33.4814
25	7.5621	7.5605	7.6063	7.5813	7.6097	7.5524	7.5727	0.0326	3.79E+03	16.2798
15	5.1115	5.0866	5.1389	5.1309	5.1038	5.1296	5.1169	0.0198	2.56E+03	9.9064
5	3.3211	3.3266	3.3531	3.3544	3.3126	3.3413	3.3348	0.0174	1.67E+03	8.6944
**Temp (°C)**	**Trial D1**	**Trial D2**	**Trial D3**	**Trial D4**	**Trial D5**	**Trial D6**	**Average k_D^{pfo} (s^{-1})**	**Stdev**	**k_{2D} (M^{-1}s^{-1})**	**Stdev [a]**
45	5.1057	5.1765	5.1484	5.1623	5.1395	5.1622	5.1491	0.0248	2.57E+03	12.4083
35	3.5157	3.5378	3.5478	3.5455	3.5849	3.6031	3.5558	0.0322	1.78E+03	16.1039
25	2.3519	2.3345	2.3654	2.3668	2.3211	2.3539	2.3489	0.0179	1.17E+03	8.9709
15	1.5186	1.5265	1.5426	1.5393	1.5394	1.5384	1.5341	0.0094	7.67E+02	4.7157
5	0.9625	0.9695	0.9669	0.9570	0.9599	0.9562	0.9620	0.0054	4.81E+02	2.6817

[a] = (Stdev(for k^{pfo})/k^{pfo})*k_{2H}

Six kinetic runs together with the averaged data	First-order kinetic fit of the averaged data as an example
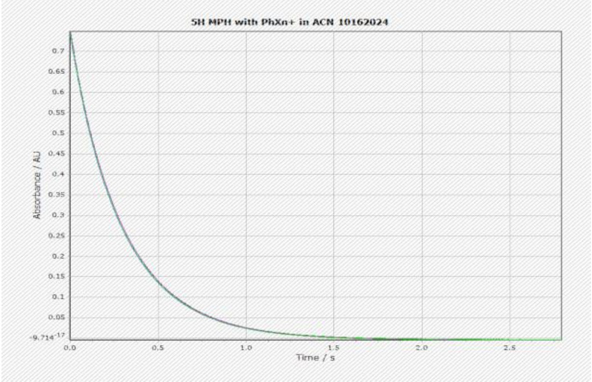	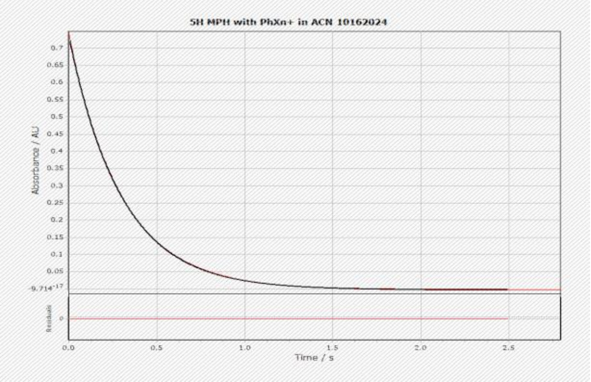

5°C H

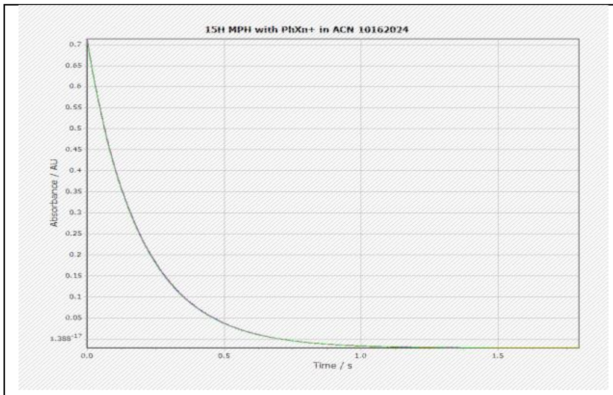

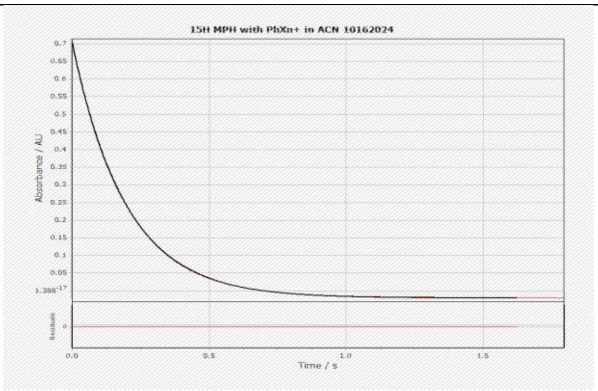

15°C H

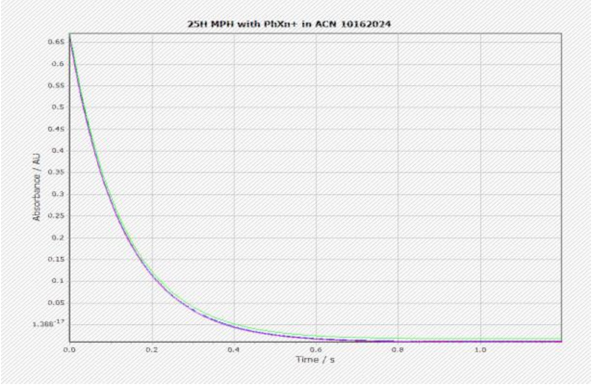

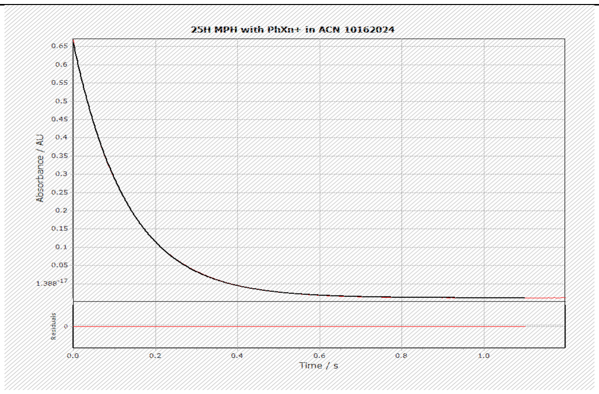

25°C H

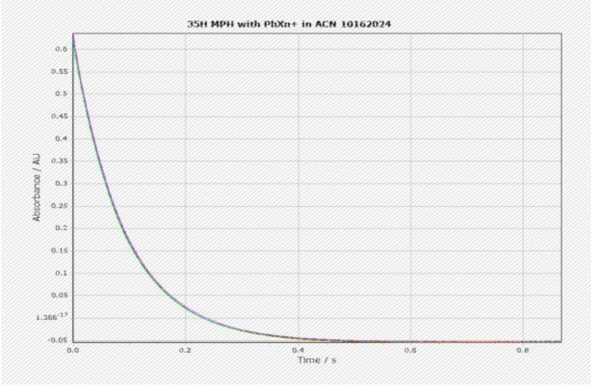

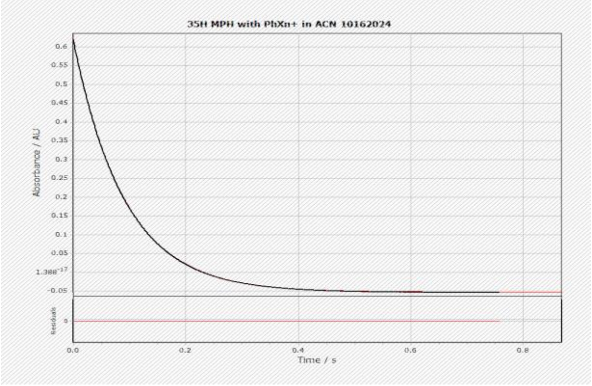

35°C H

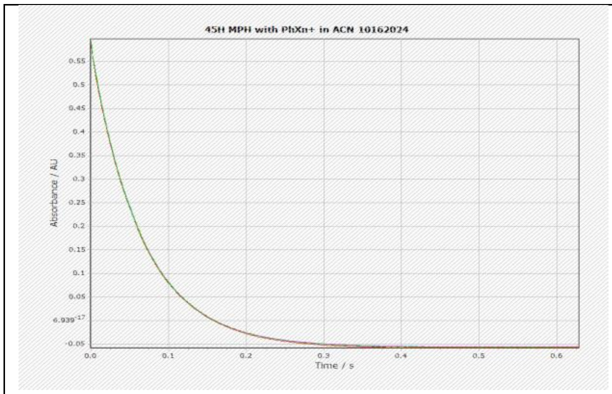
45H MPH with PhXn+ in ACN 10162024
Absorbance / AU
Time / s

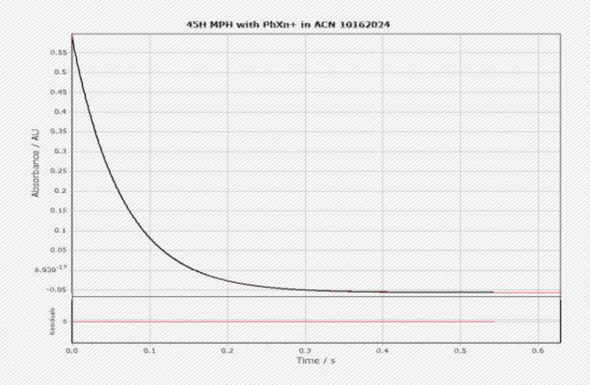
45H MPH with PhXn+ in ACN 10162024
Absorbance / AU
Residuals
Time / s

45℃ H

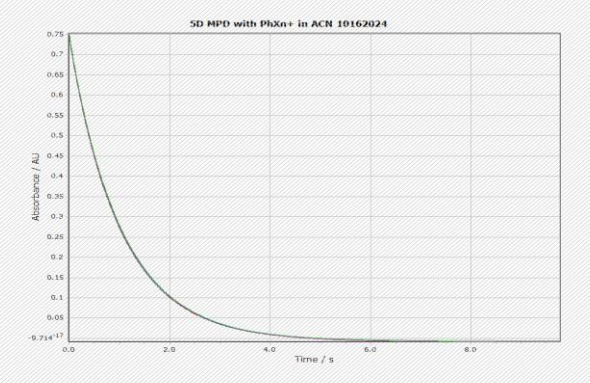
5D MPD with PhXn+ in ACN 10162024
Absorbance / AU
Time / s

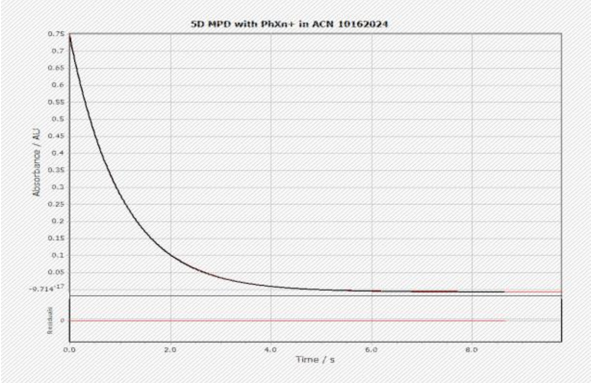
5D MPD with PhXn+ in ACN 10162024
Absorbance / AU
Residuals
Time / s

5℃ D

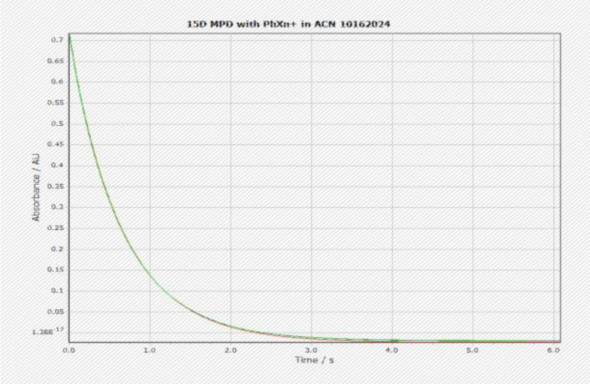
15D MPD with PhXn+ in ACN 10162024
Absorbance / AU
Time / s

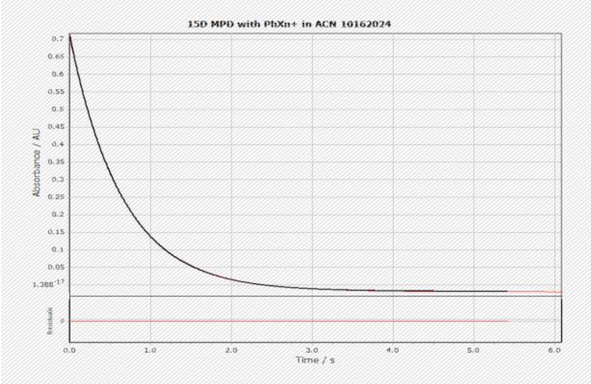
15D MPD with PhXn+ in ACN 10162024
Absorbance / AU
Residuals
Time / s

15℃ D

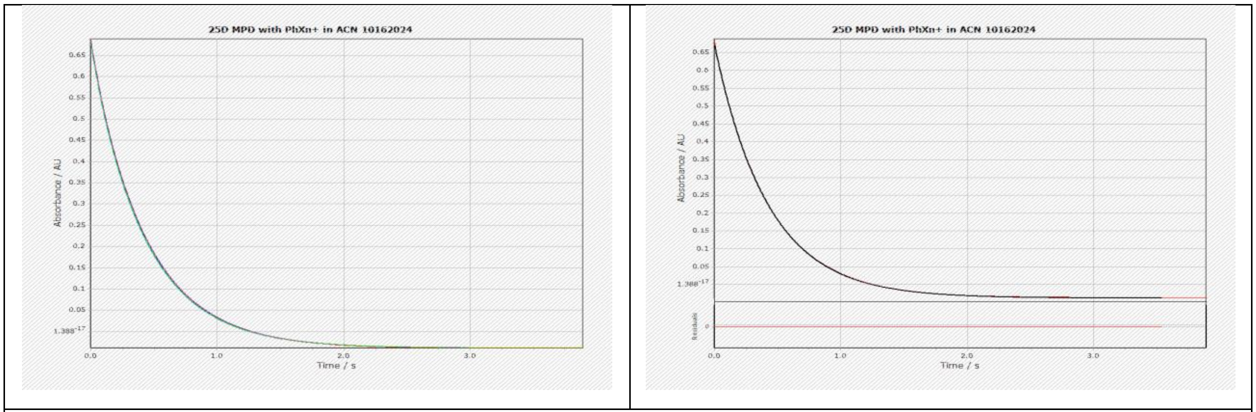

25°C D

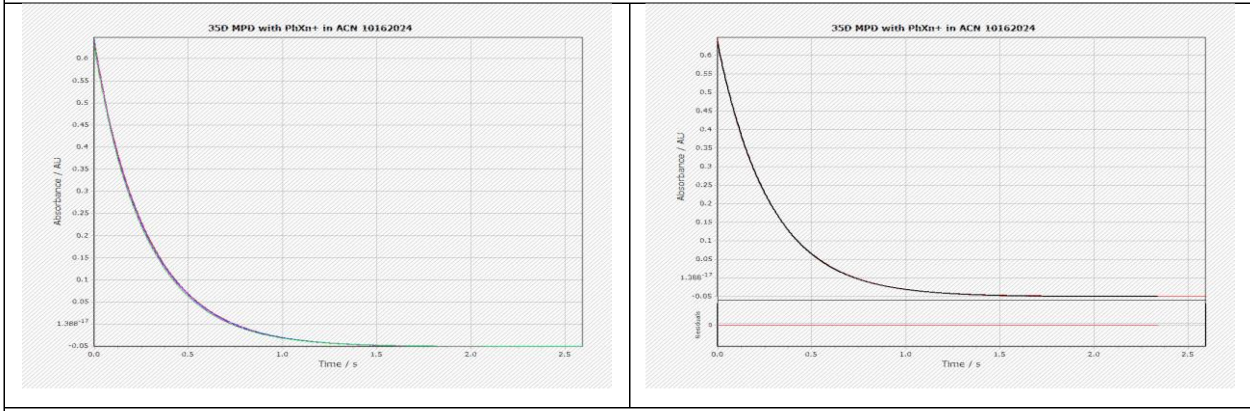

35°C D

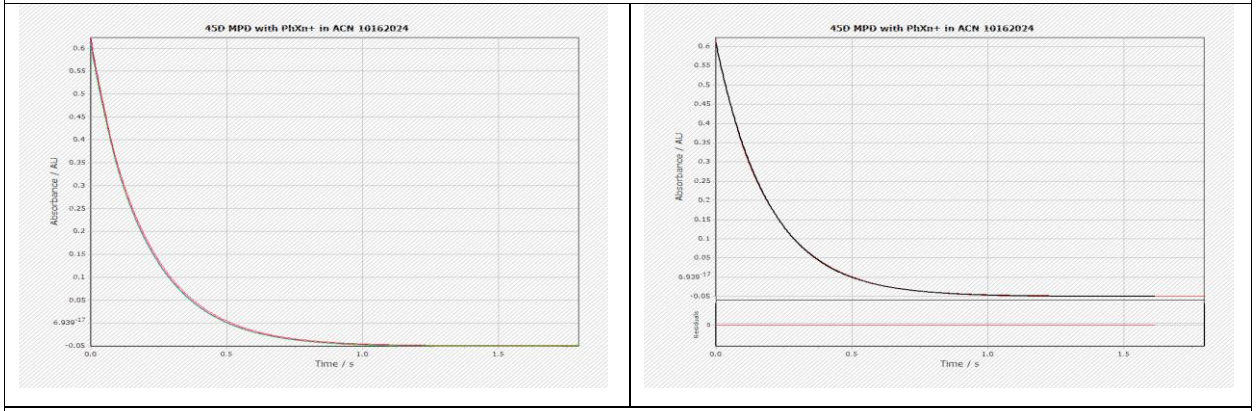

45°C D

Day 2 data (October 22, 2024)

	Pseudo-first-order rate constants k^{pfo} (s^{-1})									
**Temp (°C)**	**Trial H1**	**Trial H2**	**Trial H3**	**Trial H4**	**Trial H5**	**Trial H6**	**Average k_H^{pfo} (s^{-1})**	**Stdev**	**k_{2H} ($M^{-1}s^{-1}$)**	**Stdev [a]**
45	14.9338	14.9251	14.8089	14.8622	15.1272	15.0345	14.9486	0.1158	7.47E+03	57.8864
35	10.7175	10.7518	10.7374	10.7634	10.8016	10.8415	10.7689	0.0454	5.38E+03	22.6924
25	7.3328	7.3440	7.2972	7.2832	7.2648	7.3403	7.3104	0.0332	3.66E+03	16.5967
15	5.1028	5.0047	4.9739	5.0206	4.9714	4.9658	5.0065	0.0518	2.50E+03	25.9100
5	3.2924	3.2794	3.2887	3.2877	3.2572	3.3107	3.2860	0.0175	1.64E+03	8.7546

Temp (°C)	Trial D1	Trial D2	Trial D3	Trial D4	Trial D5	Trial D6	Average k_D^{pfo} (s^{-1})	Stdev	k_{2D} ($M^{-1}s^{-1}$)	Stdev [a]
45	5.1613	5.1338	5.1430	5.1055	5.1434	5.1717	5.1431	0.0230	2.57E+03	11.5008
35	3.5130	3.5357	3.4835	3.5062	3.4976	3.5209	3.5095	0.0182	1.75E+03	9.1069
25	2.3245	2.3182	2.3046	2.3298	2.3157	2.3483	2.3235	0.0148	1.16E+03	7.4136
15	1.5158	1.5095	1.5202	1.4995	1.5293	1.5119	1.5144	0.0101	7.57E+02	5.0522
5	0.9533	0.9598	0.9530	0.9440	0.9436	0.9556	0.9515	0.0065	4.76E+02	3.2355

[a] = (Stdev(for k^{pfo})/k^{pfo})*k_{2H}

Six kinetic runs together with the averaged data	First-order kinetic fit of the averaged data as an example
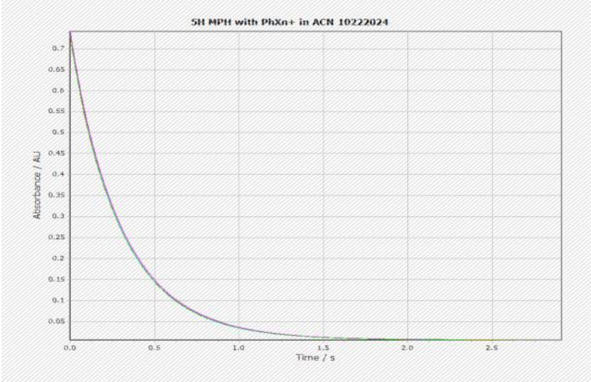	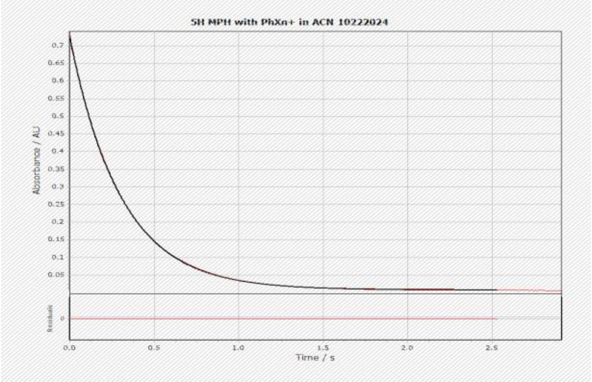
5°C H	
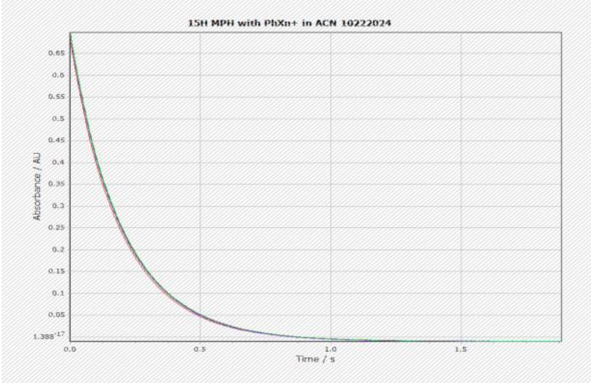	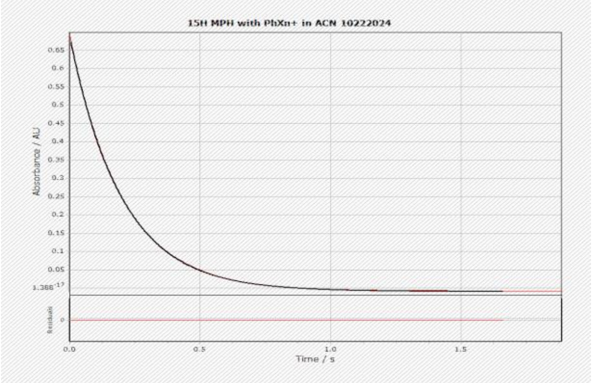
15°C H	
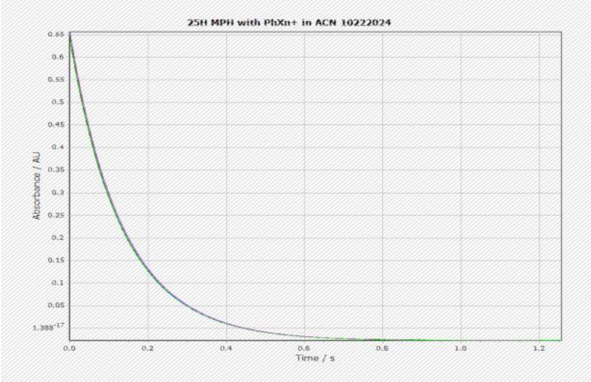	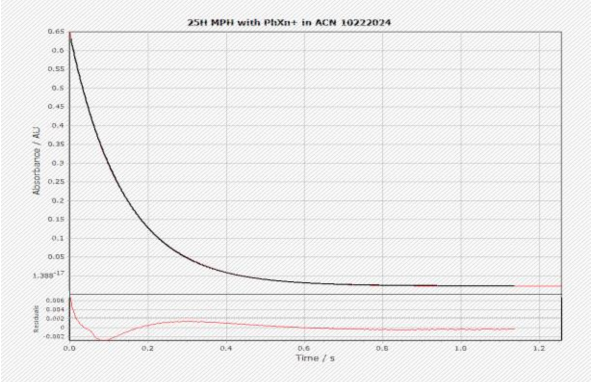
25°C H	

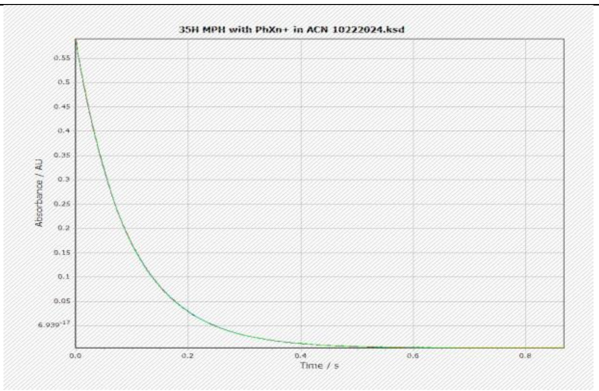
35H MPH with PhXn+ in ACN 10222024.ksd
Absorbance / AU
Time / s

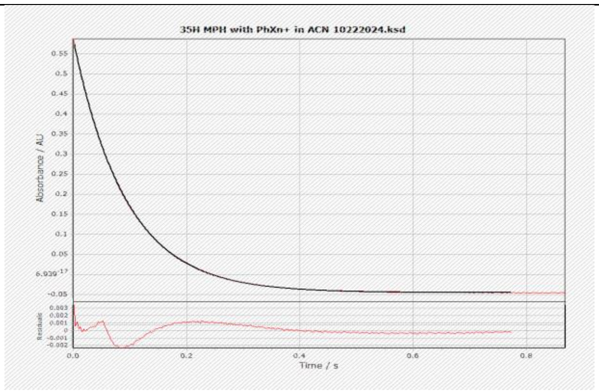
35H MPH with PhXn+ in ACN 10222024.ksd
Absorbance / AU
Residuals
Time / s

35℃ H

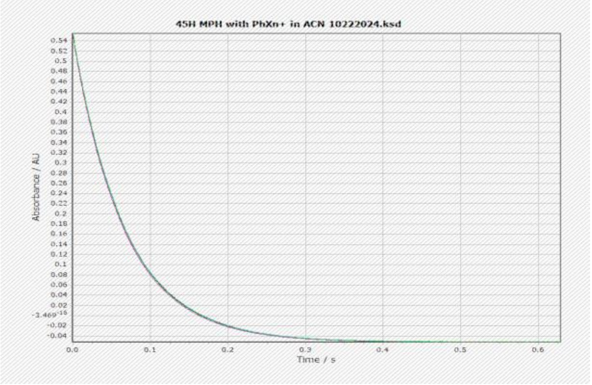
45H MPH with PhXn+ in ACN 10222024.ksd
Absorbance / AU
Time / s

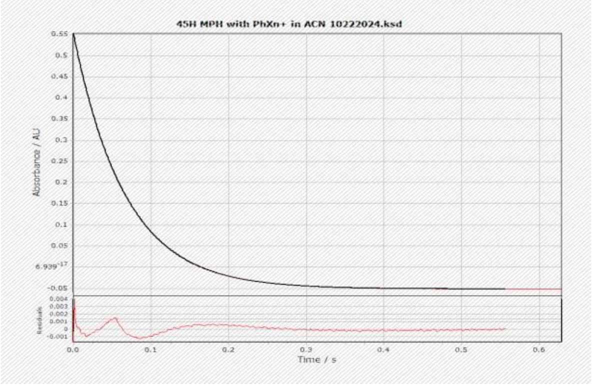
45H MPH with PhXn+ in ACN 10222024.ksd
Absorbance / AU
Residuals
Time / s

45℃ H

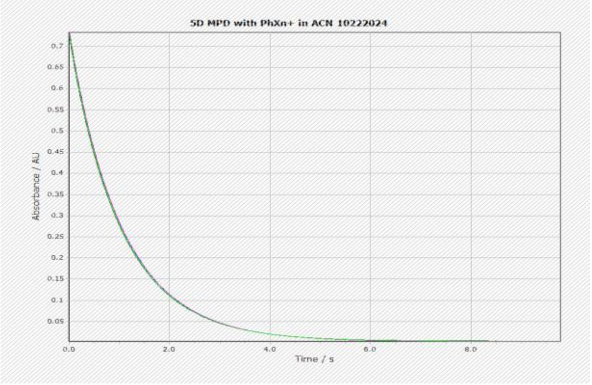
5D MPD with PhXn+ in ACN 10222024
Absorbance / AU
Time / s

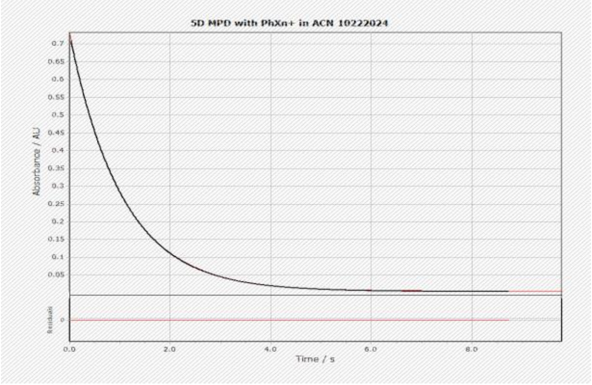
5D MPD with PhXn+ in ACN 10222024
Absorbance / AU
Residuals
Time / s

5℃ D

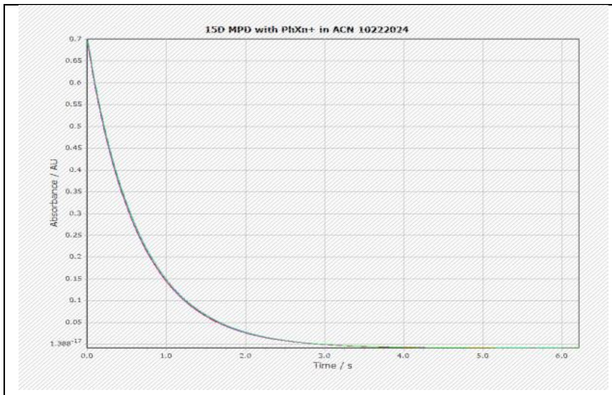

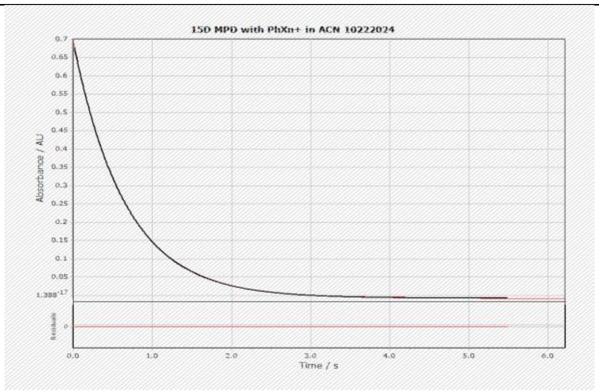

15°C D

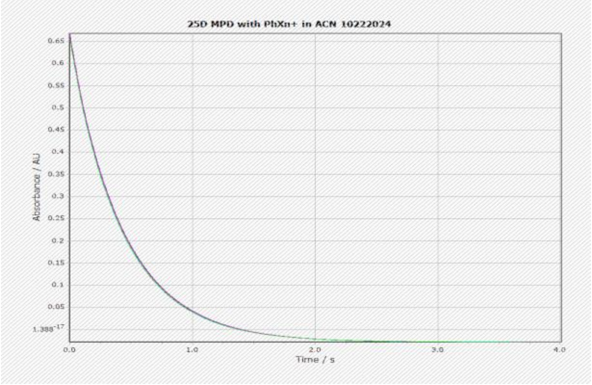

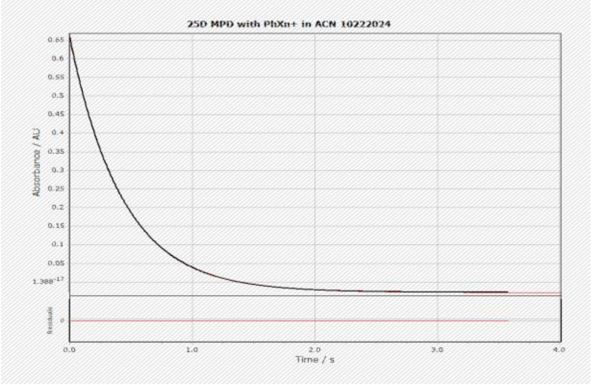

25°C D

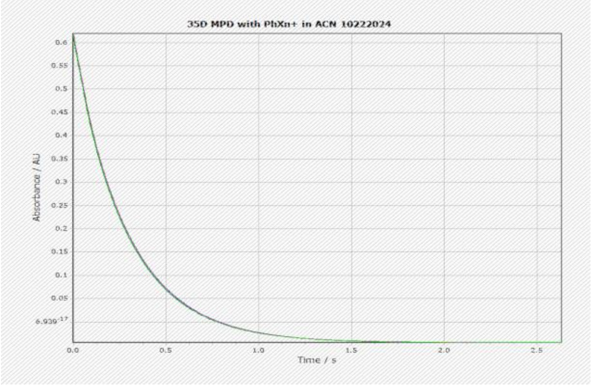

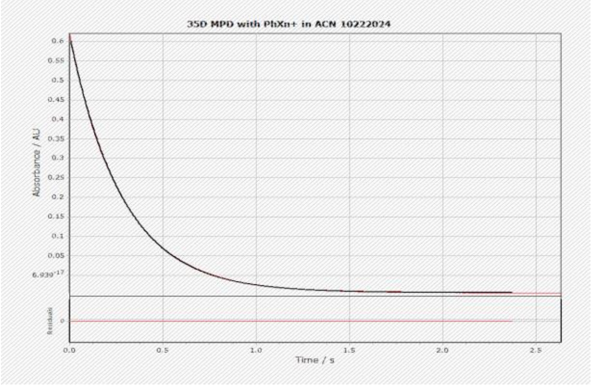

35°C D

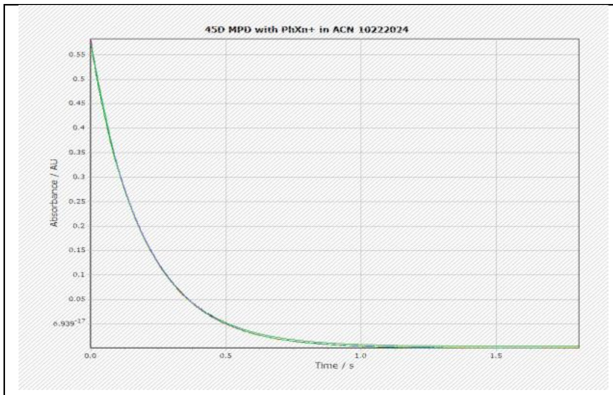

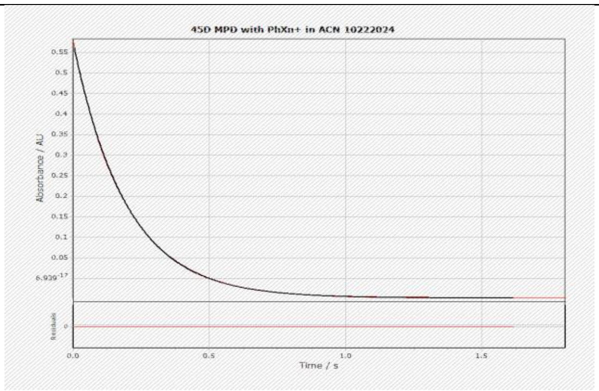

45°C D

Day 3 data (November 1, 2024)

Pseudo-first-order rate constants

Temp (°C)	Trial H1	Trial H2	Trial H3	Trial H4	Trial H5	Trial H6	Average k_H^{pfo} (s^{-1})	Stdev	k_{2H} (M^{-1}s^{-1})	Stdev [a]
	k^{pfo} (s^{-1})									
45	15.4062	15.3159	15.4748	15.3839	15.3123	15.4181	15.3852	0.0627	7.69E+03	31.3632
35	11.0229	10.9637	10.9353	11.0393	11.0734	11.1204	11.0258	0.0685	5.51E+03	34.2459
25	7.5572	7.5426	7.5969	7.6092	7.5687	7.5371	7.5686	0.0292	3.78E+03	14.5778
15	5.0604	5.0775	5.0914	5.1316	5.0883	5.0805	5.0883	0.0238	2.54E+03	11.9174
5	3.3107	3.3325	3.3206	3.3183	3.3365	3.3262	3.3241	0.0095	1.66E+03	4.7718

Temp (°C)	Trial D1	Trial D2	Trial D3	Trial D4	Trial D5	Trial D6	Average k_D^{pfo} (s^{-1})	Stdev	k_{2D} (M^{-1}s^{-1})	Stdev [a]
45	5.2456	5.2919	5.2027	5.2558	5.2795	5.2811	5.2594	0.0327	2.63E+03	16.3569
35	3.5403	3.5936	3.6076	3.5540	3.6034	3.5544	3.5756	0.0293	1.79E+03	14.6314
25	2.3626	2.4037	2.3702	2.3752	2.3897	2.3758	2.3795	0.0148	1.19E+03	7.3961
15	1.4972	1.5214	1.5287	1.5261	1.5328	1.5244	1.5218	0.0127	7.61E+02	6.3281
5	0.9617	0.9646	0.9620	0.9510	0.9609	0.9566	0.9595	0.0049	4.80E+02	2.4454

[a] = (Stdev(for k^{pfo})/k^{pfo})*k_{2H}

Six kinetic runs together with the averaged data	First-order kinetic fit of the averaged data as an example

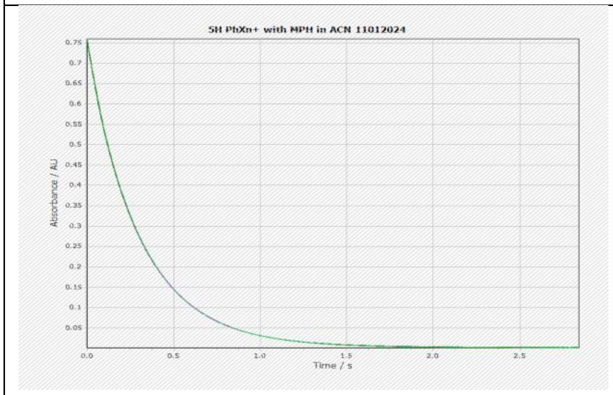

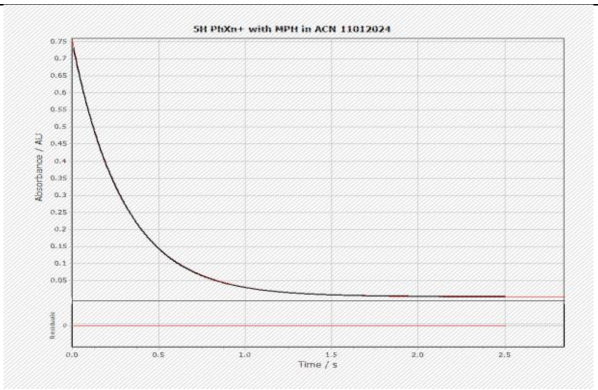

5°C H

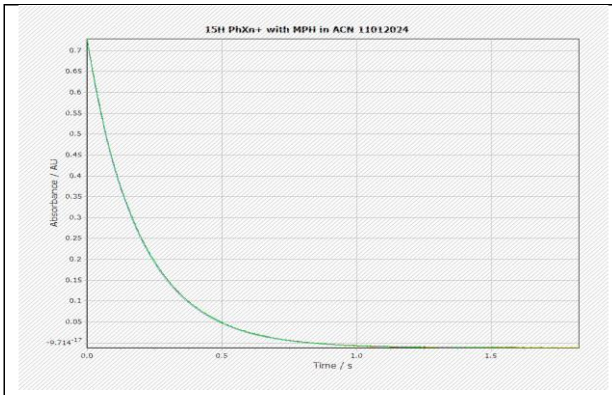

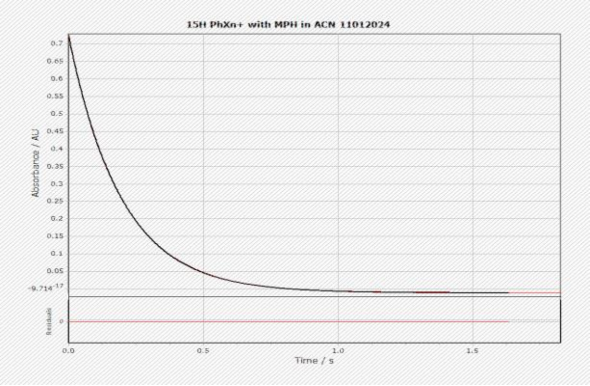

15°C H

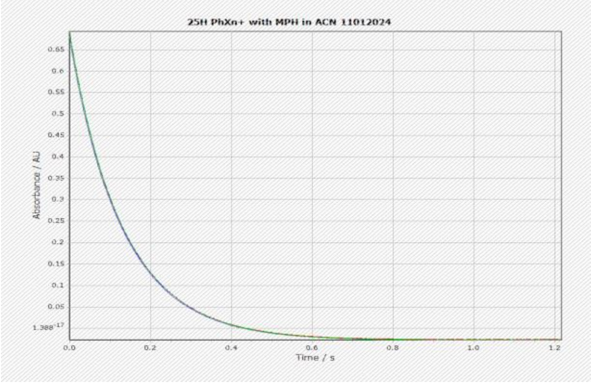

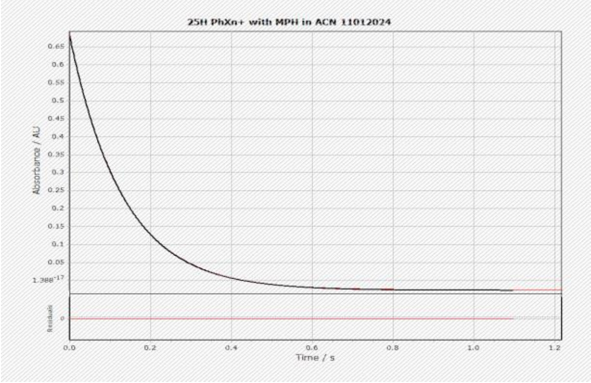

25°C H

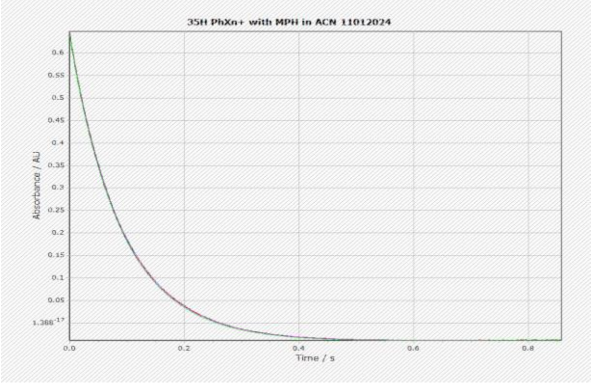

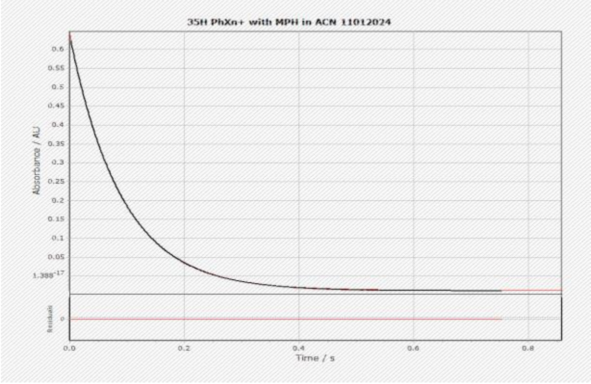

35°C H

<table>
<tr><td>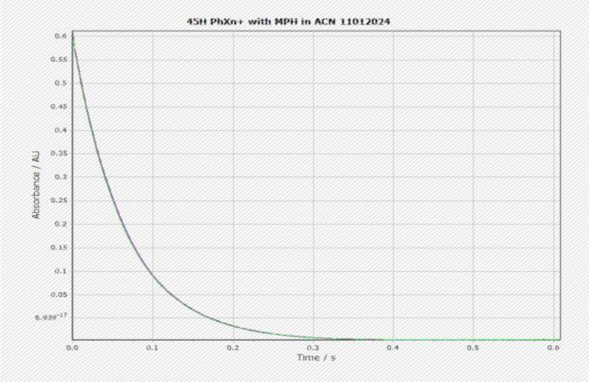
</td><td>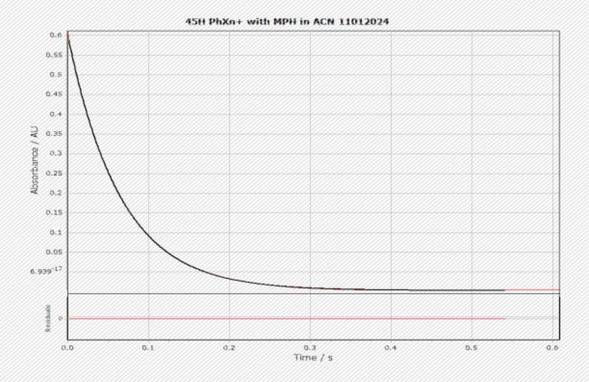
</td></tr>
<tr><td colspan="2">45°C H</td></tr>
<tr><td>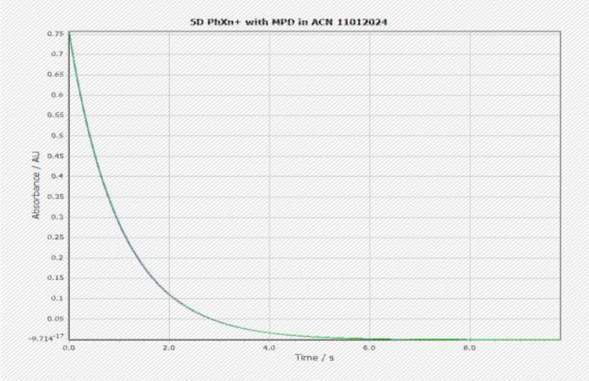
</td><td>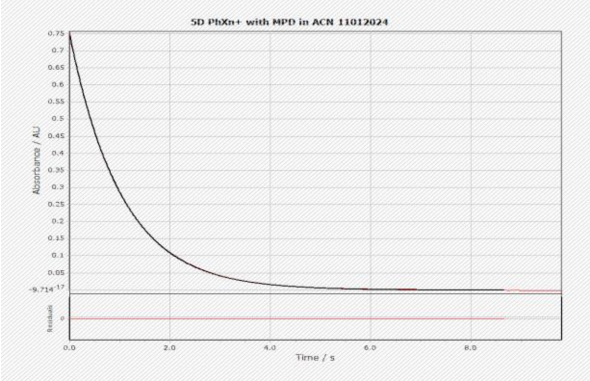
</td></tr>
<tr><td colspan="2">5°C D</td></tr>
<tr><td>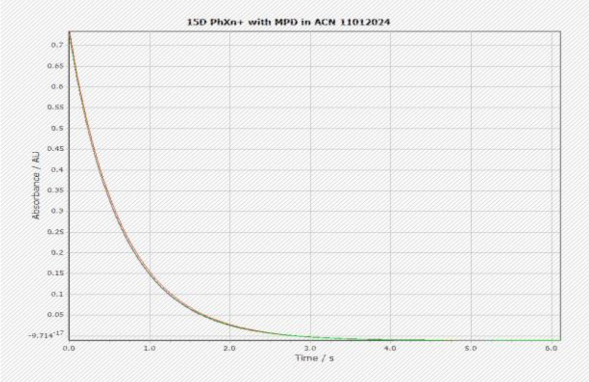
</td><td>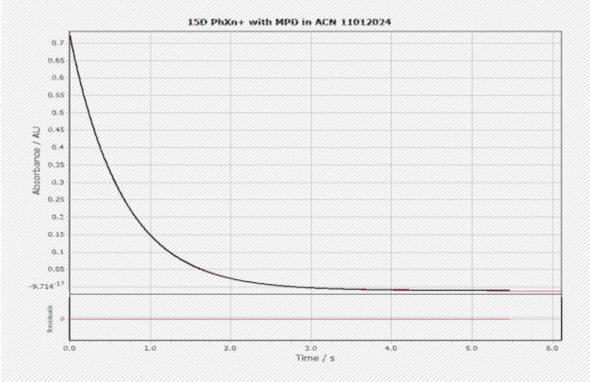
</td></tr>
<tr><td colspan="2">15°C D</td></tr>
</table>

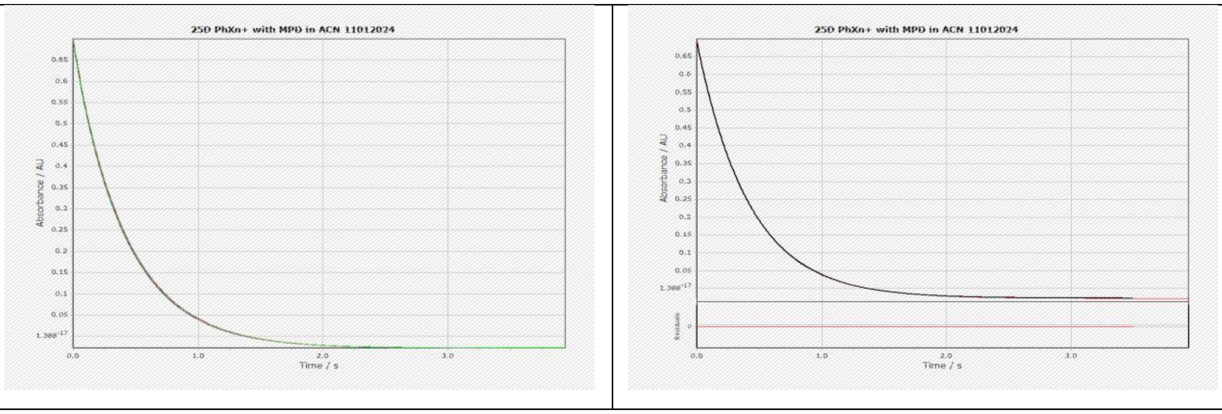

25°C D

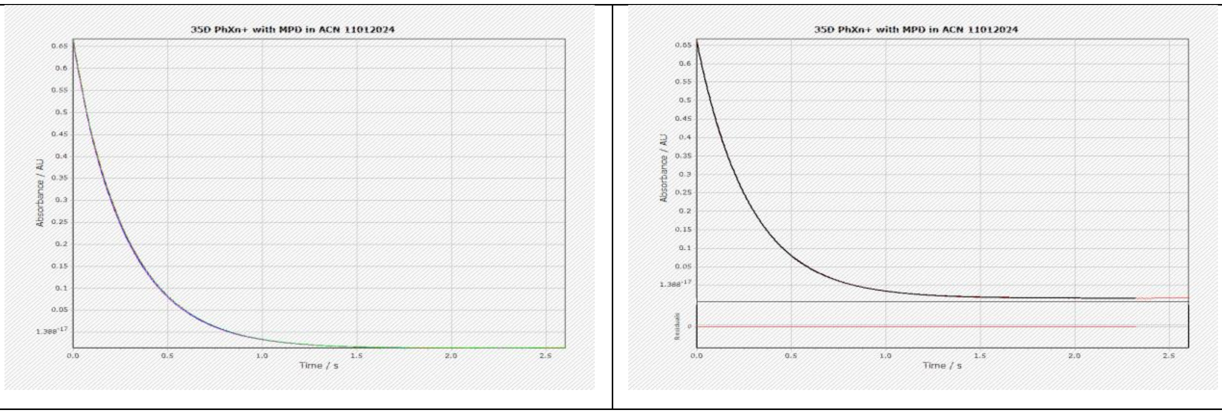

35°C D

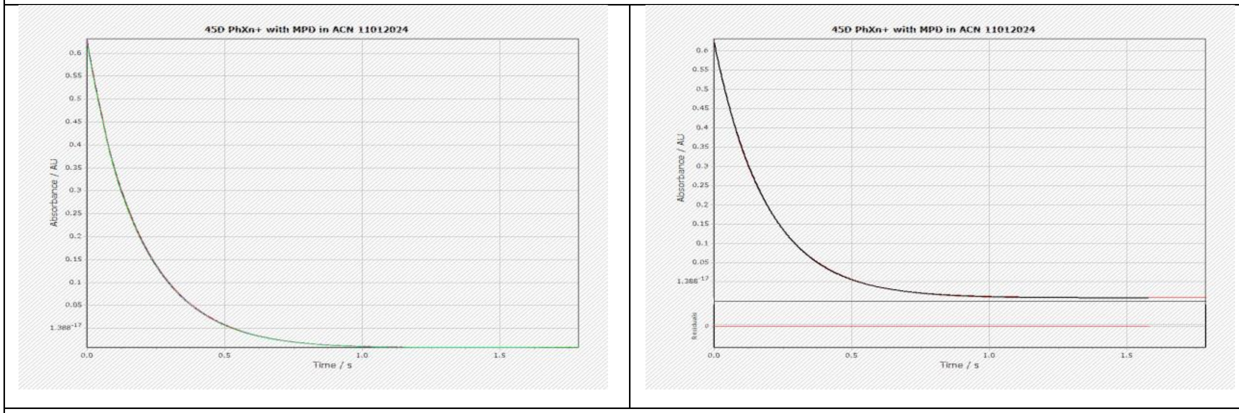

45°C D

Primary kinetic data for the rate constants in Table S2

Day 1 data (October 23, 2024)

	Pseudo-first-order rate constants k^{pfo} (s^{-1})									
**Temp (°C)**	**Trial H1**	**Trial H2**	**Trial H3**	**Trial H4**	**Trial H5**	**Trial H6**	**Average k_H^{pfo} (s^{-1})**	**Stdev**	**k_{2H} ($M^{-1}s^{-1}$)**	**Stdev [a]**
45	42.9021	43.4963	43.8100	43.8265	43.2506	44.7612	43.6744	0.6373	2.18E+04	318.6333
35	33.5091	34.0292	33.9534	33.7412	33.6769	34.0238	33.8223	0.2128	1.69E+04	106.3898
25	25.8034	25.7478	25.8690	25.7807	26.0115	25.5489	25.7936	0.1519	1.29E+04	75.9664

15	19.2735	19.1528	19.4142	19.1263	19.2649	19.0197	19.2086	0.1380	9.60E+03	69.0083
5	14.1342	13.9818	13.9623	13.9448	14.0483	13.9858	14.0095	0.0704	7.00E+03	35.2230

**Temp (°C)**	**Trial D1**	**Trial D2**	**Trial D3**	**Trial D4**	**Trial D5**	**Trial D6**	**Average k_D^{pfo} (s^{-1})**	**Stdev**	**k_{2D} ($M^{-1}s^{-1}$)**	**Stdev [a]**
45	14.1478	13.9870	13.9657	14.0656	13.9932	14.0238	14.0305	0.0671	7.02E+03	33.5608
35	10.2765	10.2575	10.2722	10.3411	10.4200	10.3068	10.3123	0.0606	5.16E+03	30.2861
25	7.4495	7.4488	7.3659	7.5079	7.3941	7.4258	7.4320	0.0494	3.72E+03	24.7023
15	5.1889	5.2083	5.2893	5.2687	5.3192	5.2718	5.2577	0.0495	2.63E+03	24.7679
5	3.6135	3.6229	3.5950	3.6125	3.6182	3.6302	3.6154	0.0119	1.81E+03	5.9516

[a] = (Stdev(for k^{pfo})/k^{pfo})*k_{2H}

Six kinetic runs together with the averaged data	First-order kinetic fit of the averaged data as an example
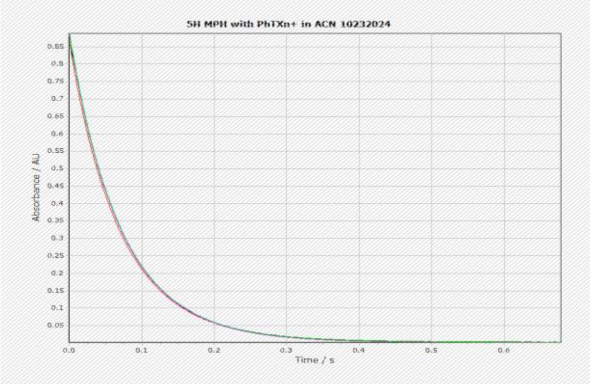	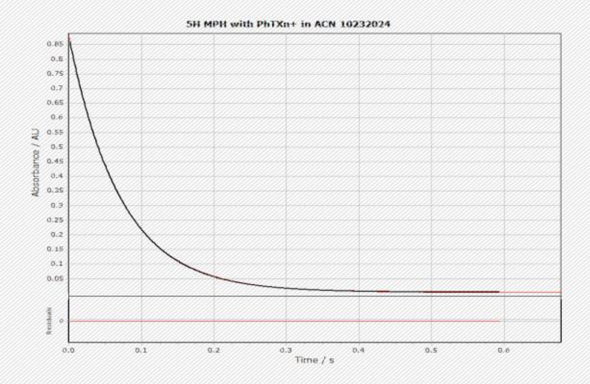
5°C H	
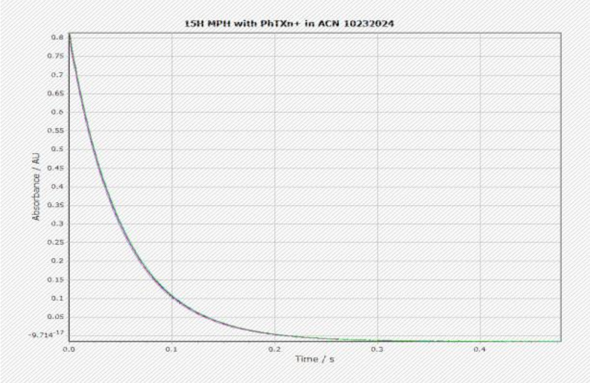	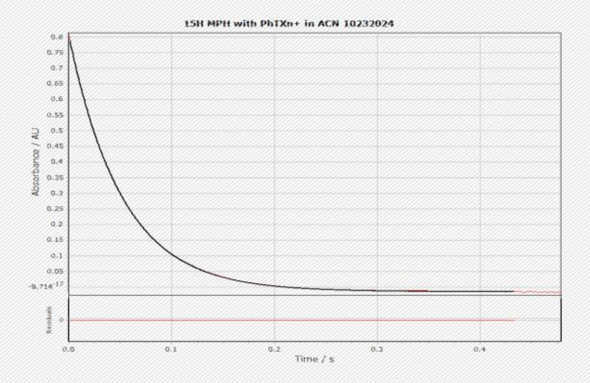
15°C H	

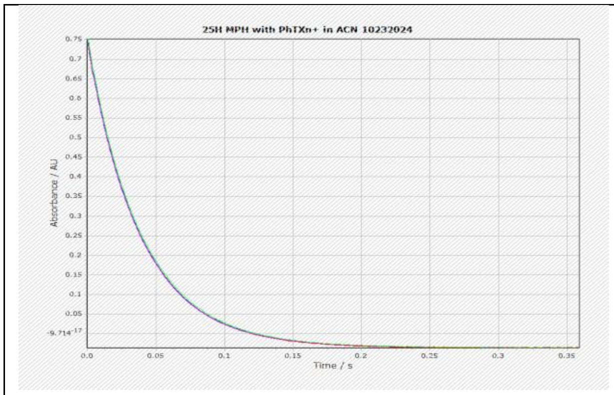

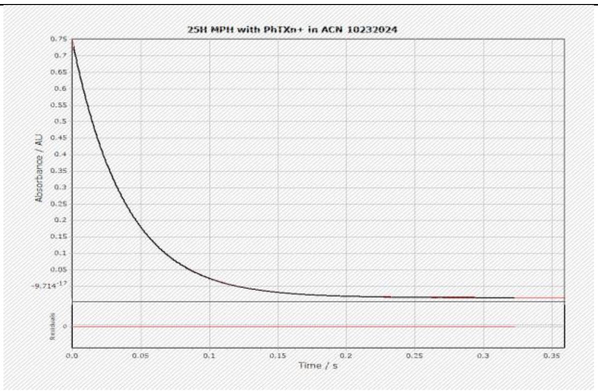

25°C H

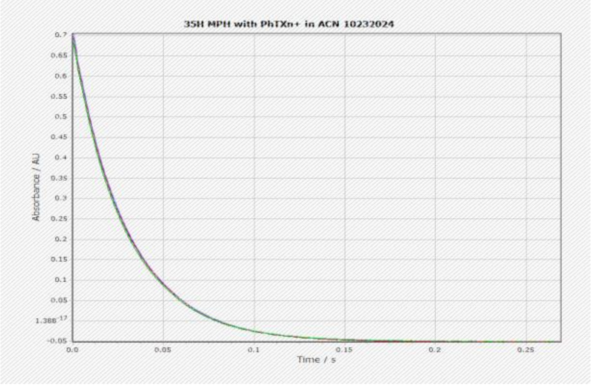

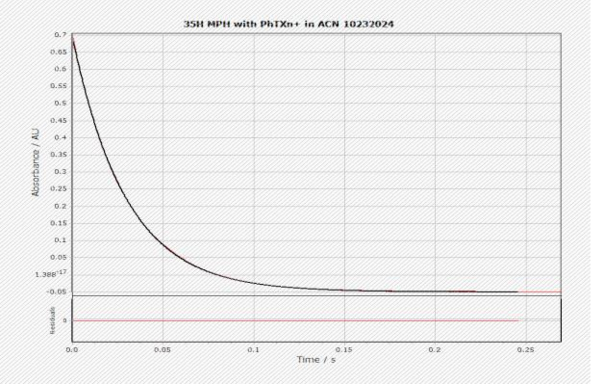

35°C H

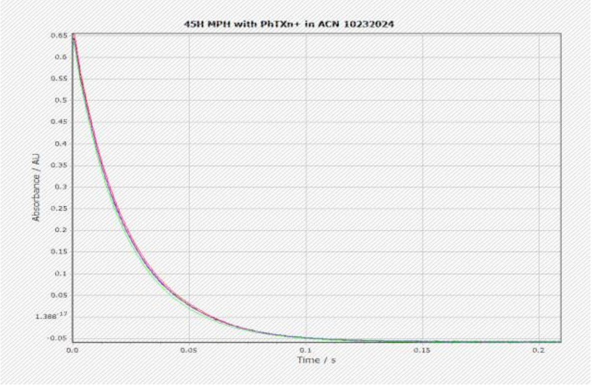

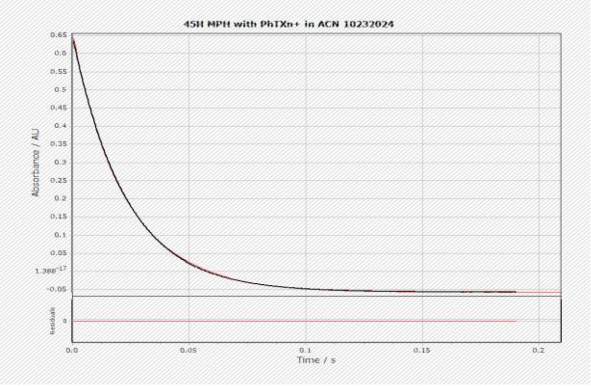

45°C H

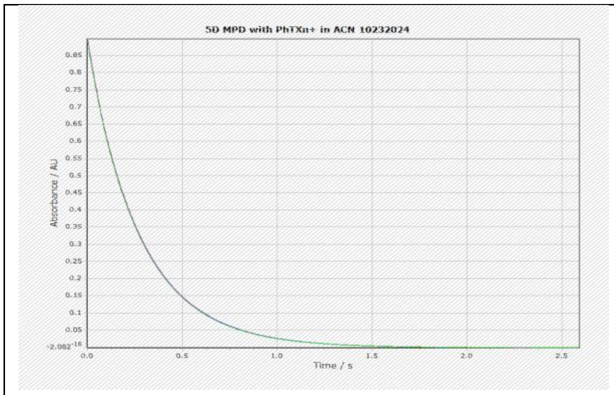

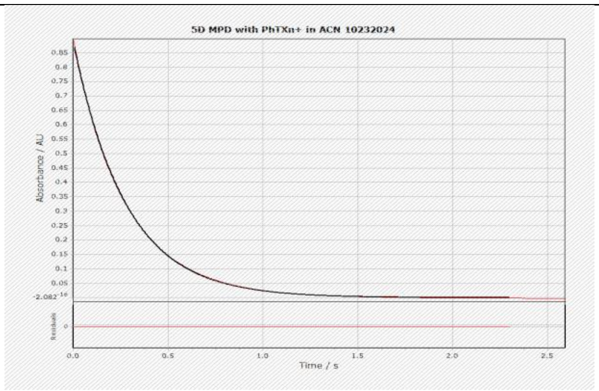

5°C D

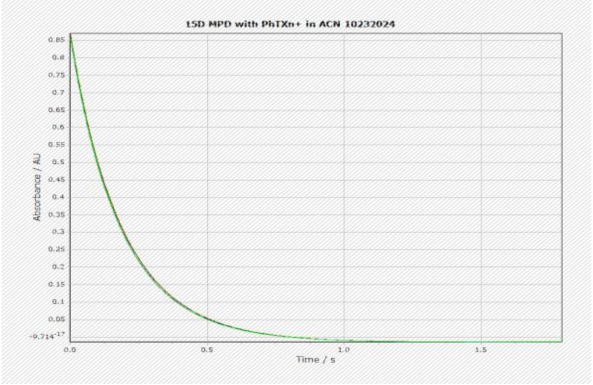

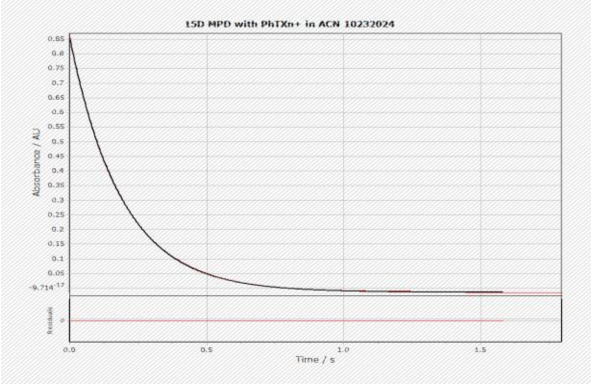

15°C D

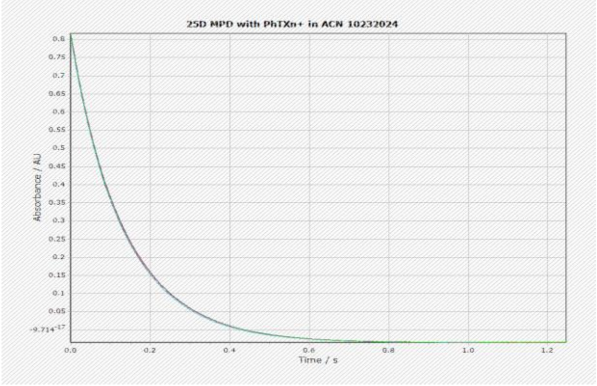

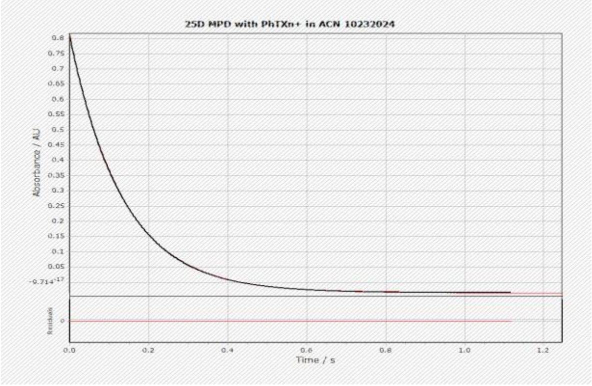

25°C D

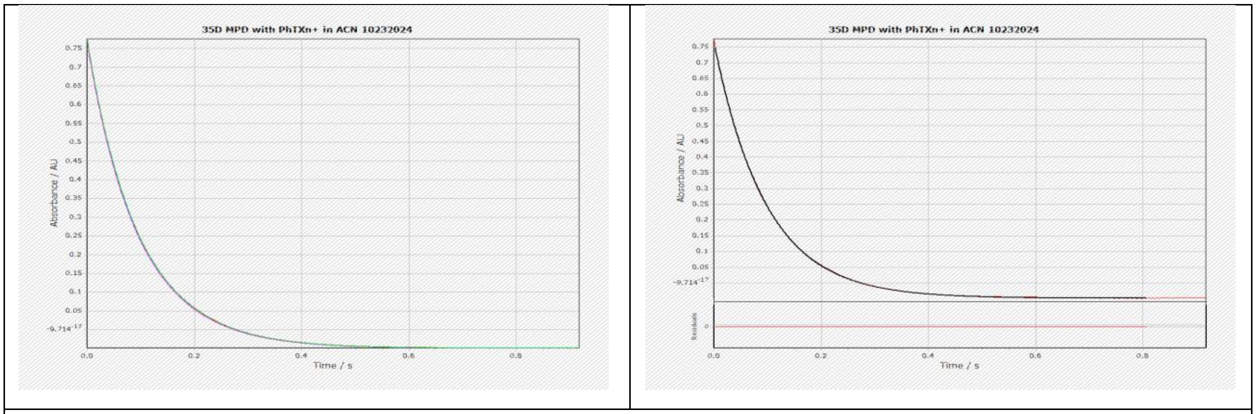

35°C D

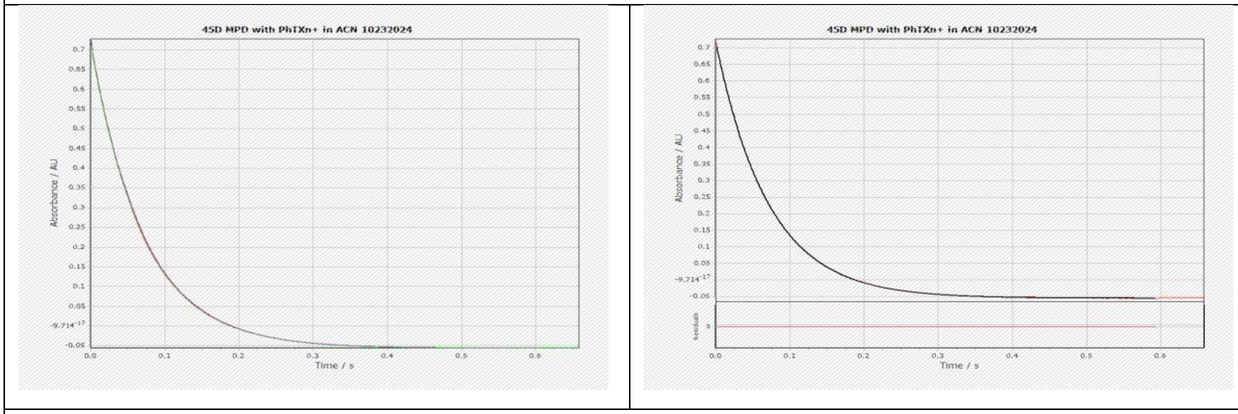

45°C D

Day 2 data (October 30, 2024)

Pseudo-first-order rate constants

	k^{pfo} (s^{-1})									
**Temp (°C)**	**Trial H1**	**Trial H2**	**Trial H3**	**Trial H4**	**Trial H5**	**Trial H6**	**Average k_H^{pfo} (s^{-1})**	**Stdev**	**k_{2H} (M^{-1}s^{-1})**	**Stdev [a]**
45	44.3018	44.4957	44.8588	43.9747	44.5634	44.9795	44.5289	0.3668	2.23E+04	183.4219
35	34.1660	34.6445	34.1369	33.9847	33.7059	34.4080	34.1743	0.3263	1.71E+04	163.1709
25	26.6896	26.4046	26.1876	26.3413	26.0852	26.8467	26.4258	0.2923	1.32E+04	146.1317
15	19.6262	19.6740	19.6428	19.6330	19.3542	19.6278	19.5930	0.1183	9.80E+03	59.1602
5	14.2987	14.3886	14.3969	14.3960	14.3155	14.3995	14.3659	0.0460	7.18E+03	22.9794
**Temp (°C)**	**Trial D1**	**Trial D2**	**Trial D3**	**Trial D4**	**Trial D5**	**Trial D6**	**Average k_D^{pfo} (s^{-1})**	**Stdev**	**k_{2D} (M^{-1}s^{-1})**	**Stdev [a]**
45	14.3185	14.0281	14.2065	14.1793	14.3322	14.0588	14.1872	0.1269	7.09E+03	63.4519
35	10.1742	10.3657	10.3807	10.2730	10.3663	10.2574	10.3027	0.0818	5.15E+03	40.8860
25	7.5539	7.3212	7.5178	7.4450	7.4773	7.4493	7.4607	0.0801	3.73E+03	40.0400
15	5.3139	5.2925	5.3257	5.2431	5.2128	5.2691	5.2762	0.0431	2.64E+03	21.5680
5	3.5938	3.5935	3.6123	3.6426	3.6017	3.6180	3.6103	0.0186	1.81E+03	9.3199

[a] = (Stdev(for k^{pfo})/k^{pfo})*k_{2H}

Six kinetic runs together with the averaged data	First-order kinetic fit of the averaged data as an example
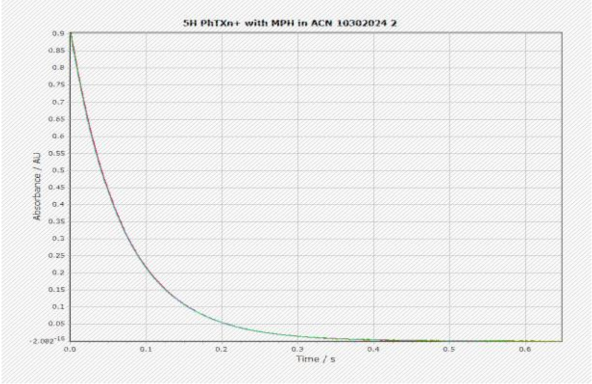	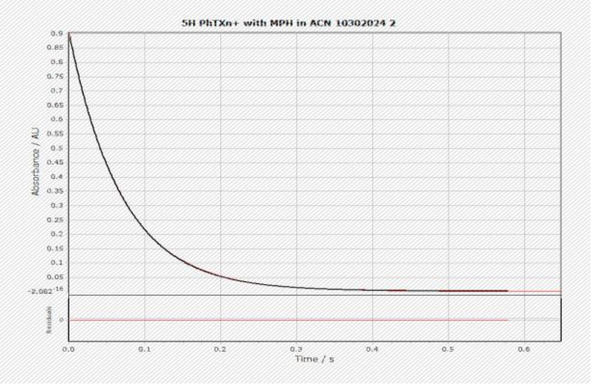
5℃ H	
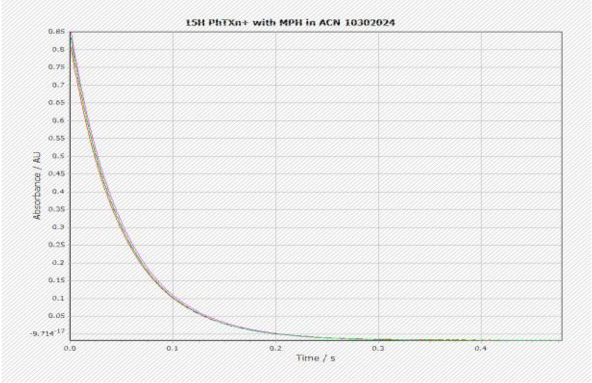	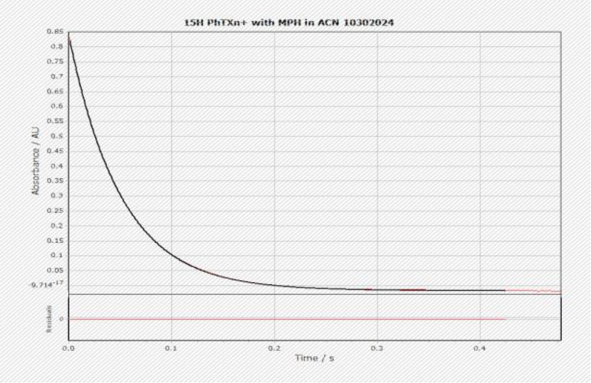
15℃ H	
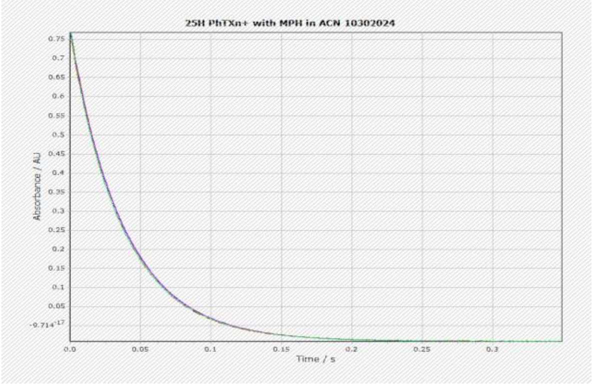	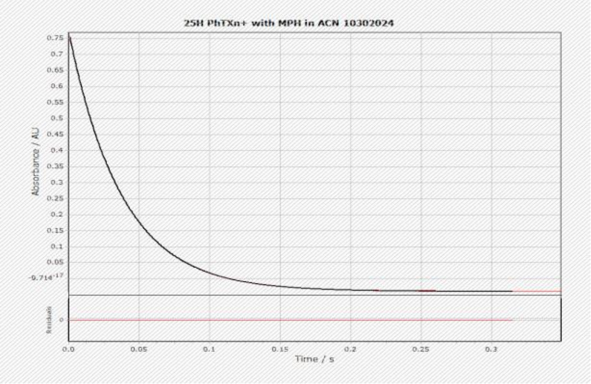
25℃ H	

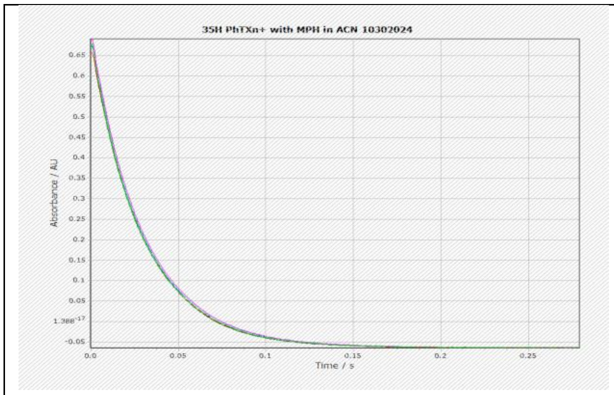
35H PhTXn+ with MPH in ACN 10302024
Absorbance / AU
Time / s

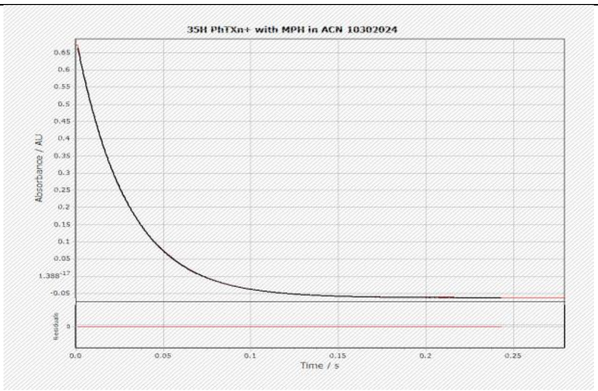
35H PhTXn+ with MPH in ACN 10302024
Absorbance / AU
Time / s

35℃ H

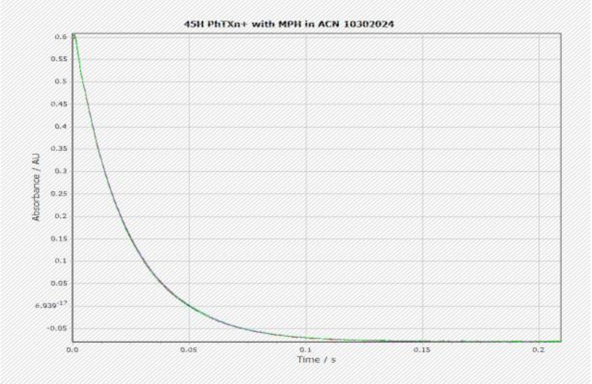
45H PhTXn+ with MPH in ACN 10302024
Absorbance / AU
Time / s

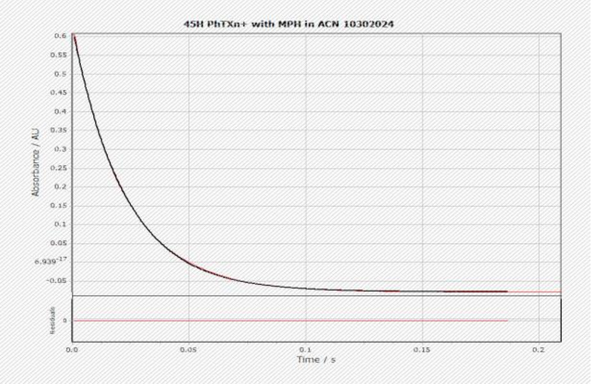
45H PhTXn+ with MPH in ACN 10302024
Absorbance / AU
Time / s

45℃ H

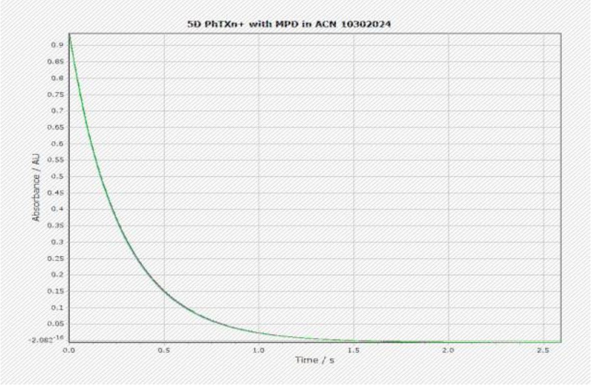
5D PhTXn+ with MPD in ACN 10302024
Absorbance / AU
Time / s

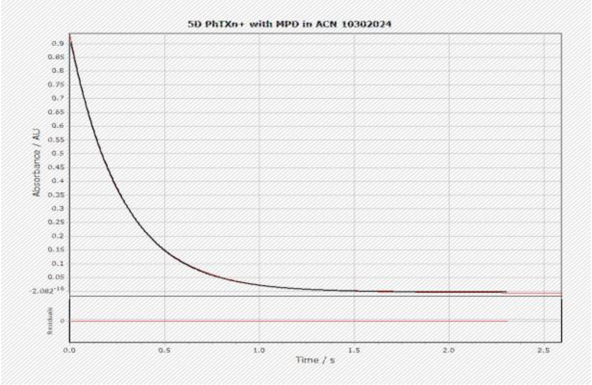
5D PhTXn+ with MPD in ACN 10302024
Absorbance / AU
Time / s

5℃ D

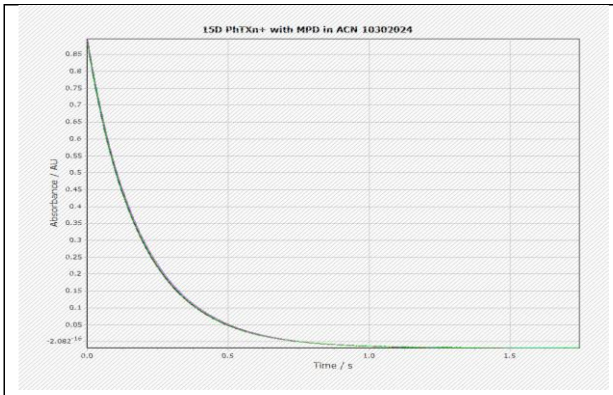

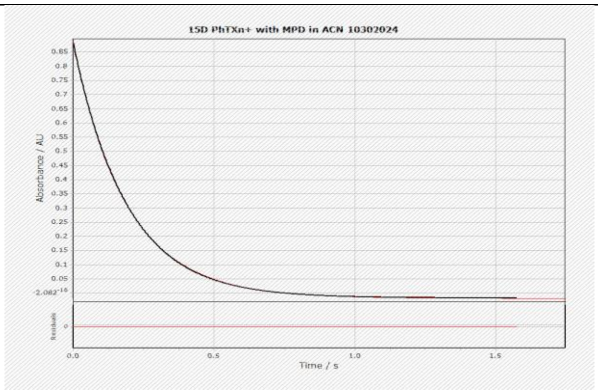

15°C D

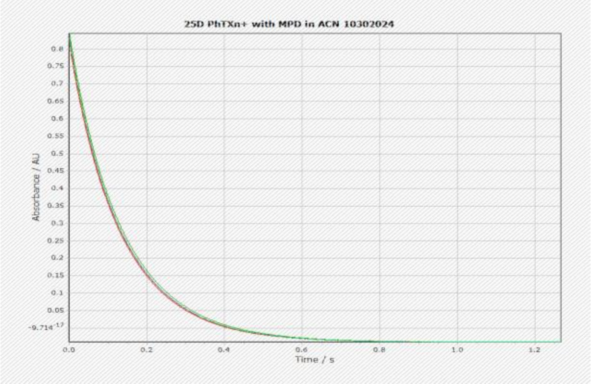

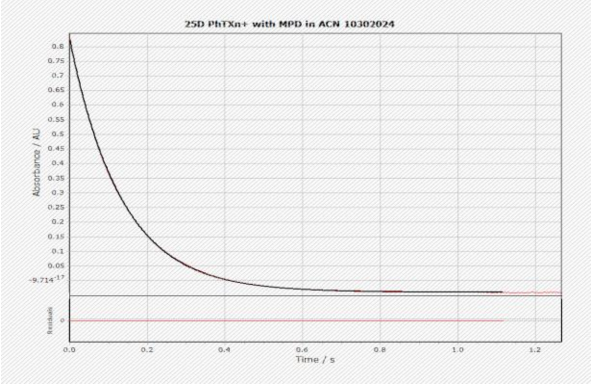

25°C D

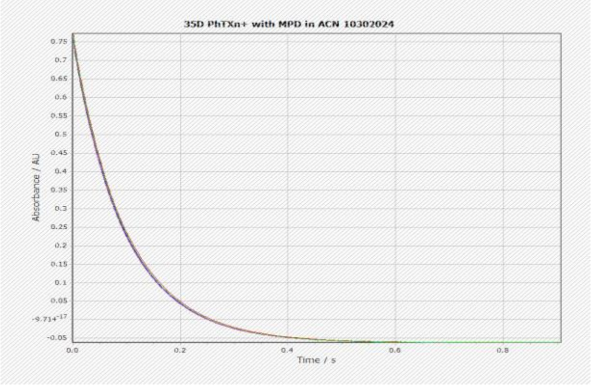

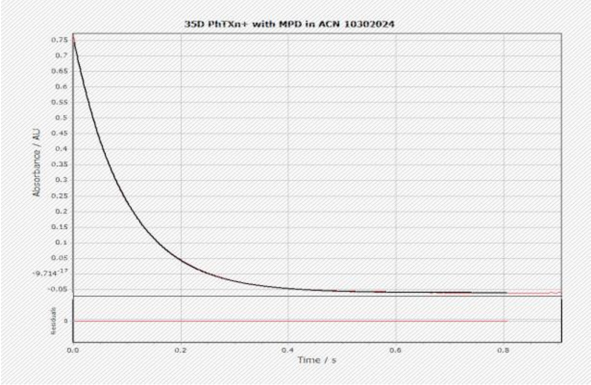

35°C D

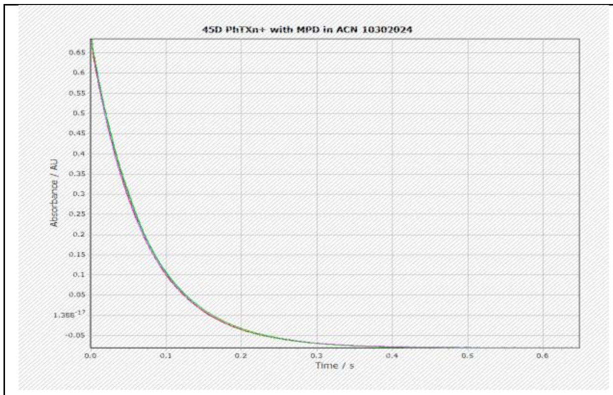

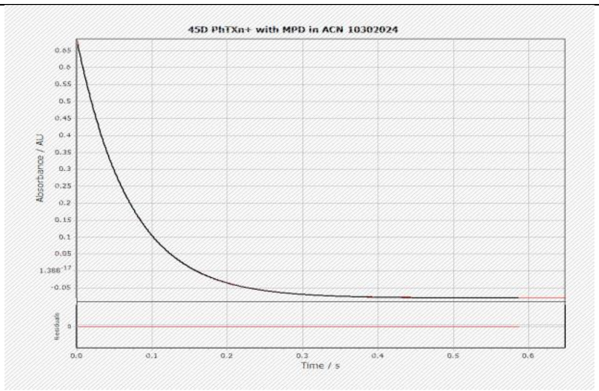

45°C D

Day 3 data (November 6, 2024)

Pseudo-first-order rate constants

	k^{pfo} (s^{-1})									
**Temp (°C)**	**Trial H1**	**Trial H2**	**Trial H3**	**Trial H4**	**Trial H5**	**Trial H6**	**Average k_H^{pfo} (s^{-1})**	**Stdev**	**k_{2H} (M^{-1}s^{-1})**	**Stdev [a]**
45	44.2574	44.3682	45.3631	45.1182	45.8779	45.1962	45.1968	0.5310	2.26E+04	265.4777
35	34.5474	35.2516	34.0874	35.1701	34.6248	34.4821	34.6939	0.4420	1.73E+04	220.9758
25	26.8717	26.5236	26.5390	26.4154	26.4086	26.5382	26.5494	0.1689	1.33E+04	84.4340
15	19.5331	19.5321	19.7133	19.7053	19.5718	19.6364	19.6153	0.0821	9.81E+03	41.0651
5	14.2749	14.2075	14.3783	14.2334	14.4947	14.2827	14.3119	0.1069	7.16E+03	53.4395

**Temp (°C)**	**Trial D1**	**Trial D2**	**Trial D3**	**Trial D4**	**Trial D5**	**Trial D6**	**Average k_D^{pfo} (s^{-1})**	**Stdev**	**k_{2D} (M^{-1}s^{-1})**	**Stdev [a]**
45	13.9203	13.8827	13.9374	14.0747	13.6380	14.0051	13.9097	0.1495	6.95E+03	74.7558
35	10.1192	10.1174	10.2331	10.2377	10.2592	10.3273	10.2157	0.0826	5.11E+03	41.2902
25	7.4628	7.4331	7.4839	7.4883	7.4798	7.4332	7.4635	0.0250	3.73E+03	12.5187
15	5.2749	5.2937	5.2893	5.2566	5.2644	5.2121	5.2651	0.0296	2.63E+03	14.8078
5	3.6247	3.5776	3.6218	3.5831	3.5809	3.6054	3.5989	0.0212	1.80E+03	10.6186

[a] = (Stdev(for k^{pfo})/k^{pfo})*k_{2H}

Six kinetic runs together with the averaged data	First-order kinetic fit of the averaged data as an example
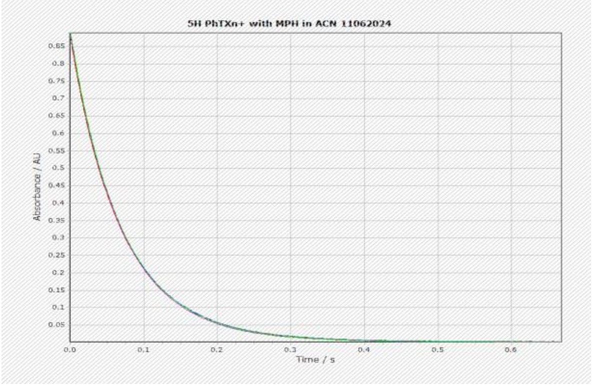	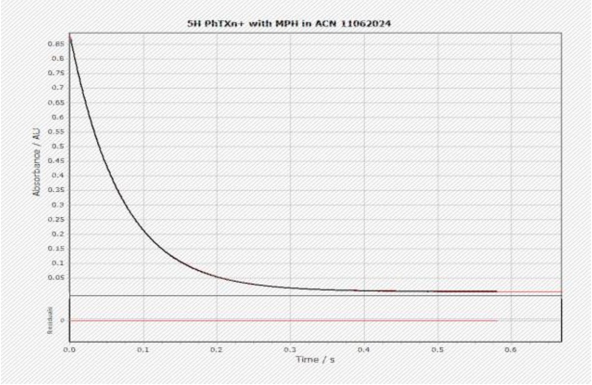

5°C H

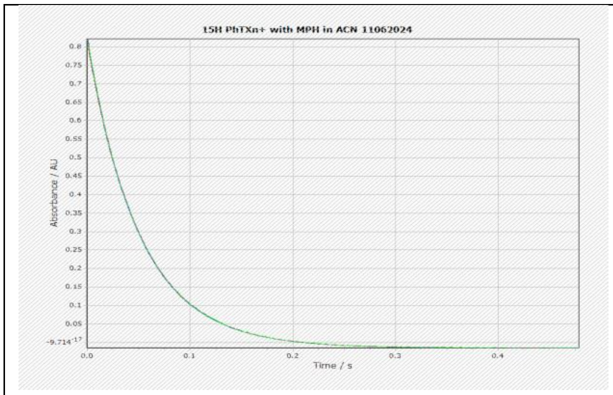

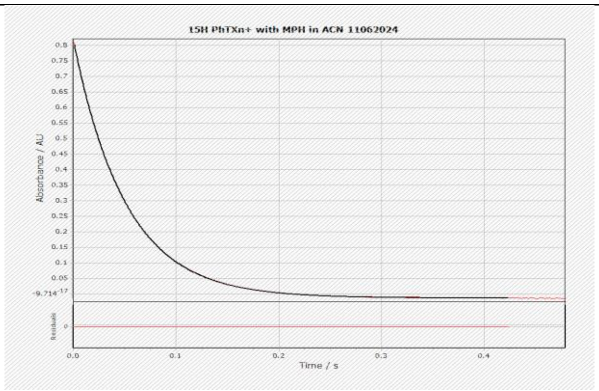

15°C H

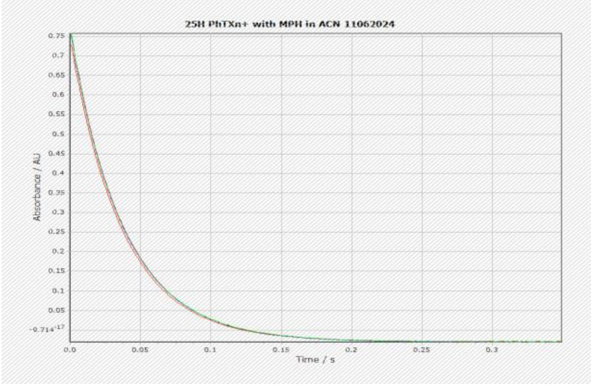

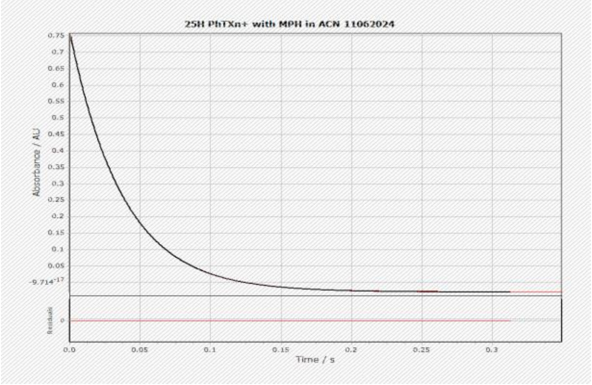

25°C H

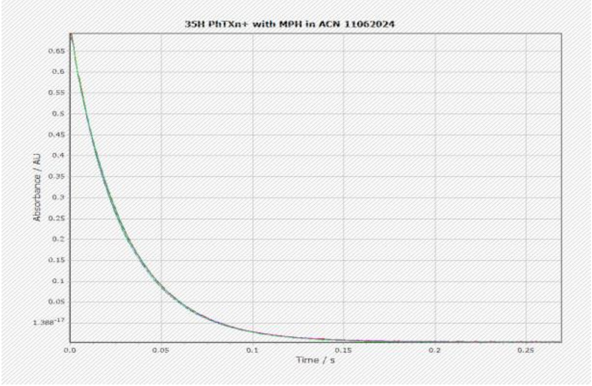

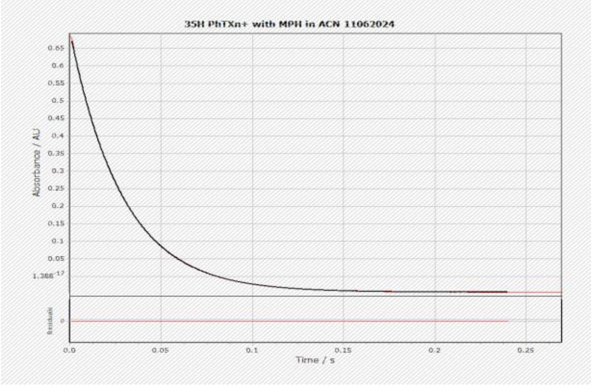

35°C H

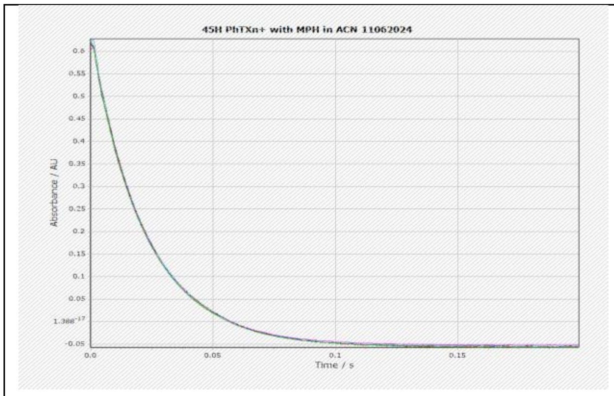

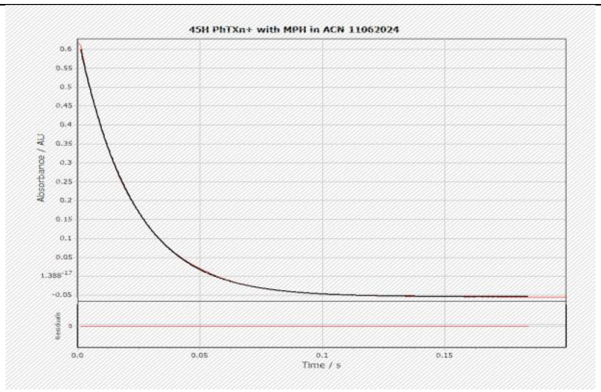

45°C H

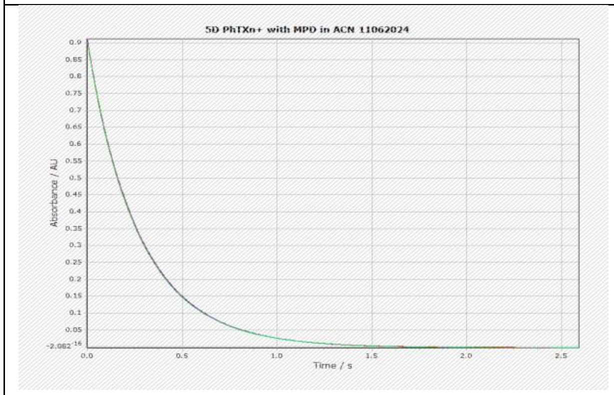

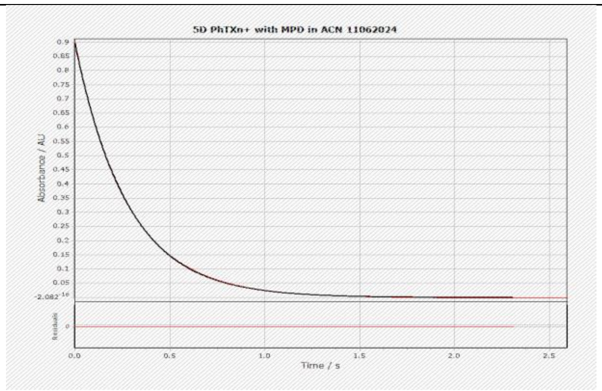

5°C D

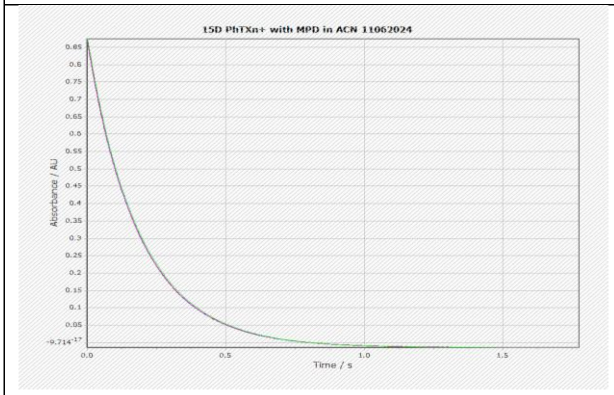

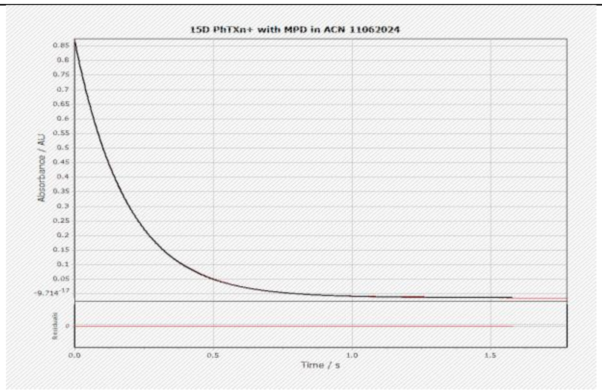

15°C D

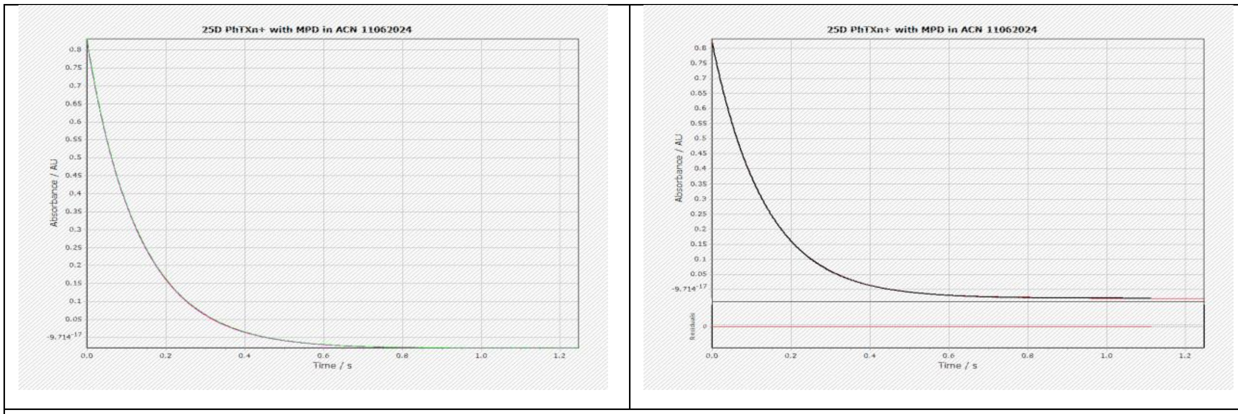

25°C D

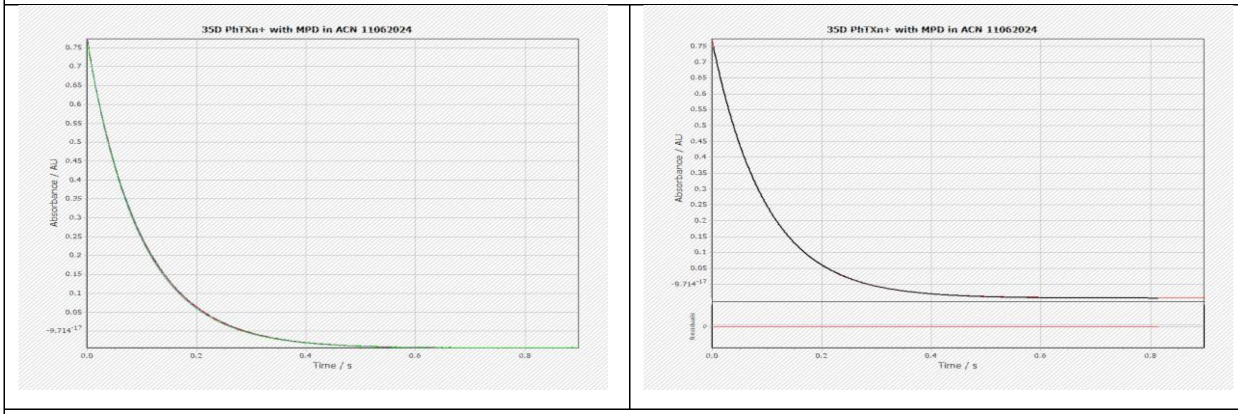

35°C D

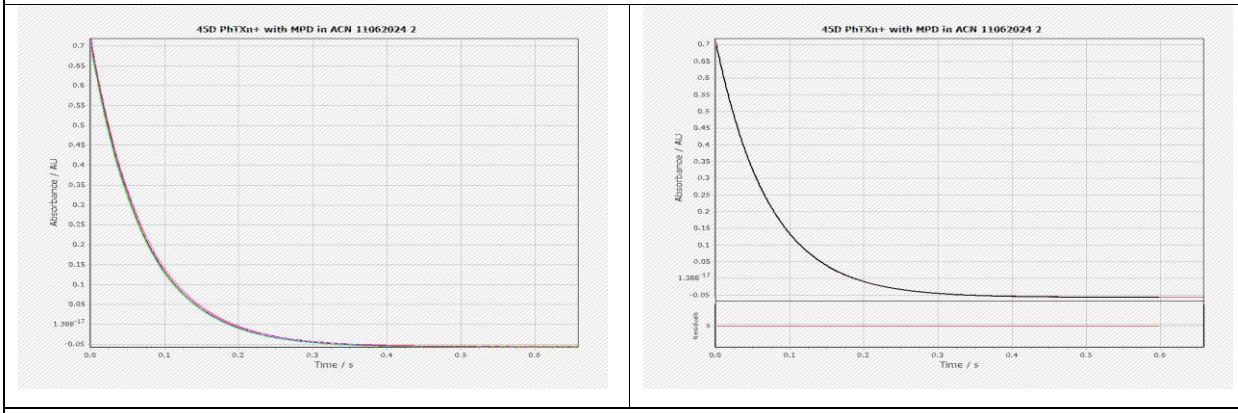

45°C D

Primary kinetic data for the rate constants in Table S3

Day 1 data (November 12, 2023)

	Pseudo-first-order rate constants k_H^{pfo} (s^{-1})									
**Temp (°C)**	**Trial H1**	**Trial H2**	**Trial H3**	**Trial H4**	**Trial H5**	**Trial H6**	**Average k_H^{pfo} (s^{-1})**	**Stdev**	**k_{2H} ($M^{-1}s^{-1}$)**	**Stdev [a]**
45	4.7568	4.7267	4.8213	4.8234	4.8055	4.7520	4.7810	0.0410	7.97E+02	6.8278
35	3.3486	3.3766	3.3399	3.3654	3.3461	3.3622	3.3565	0.0139	5.59E+02	2.3087
25	2.2562	2.2786	2.2672	2.2556	2.2715	2.2828	2.2686	0.0113	3.78E+02	1.8767
15	1.4971	1.5000	1.5090	1.5198	1.4860	1.5106	1.5037	0.0119	2.51E+02	1.9786
5	0.9482	0.9571	0.9520	0.9654	0.9566	0.9507	0.9550	0.0062	1.59E+02	1.0257

Temp (°C)	Trial D1	Trial D2	Trial D3	Trial D4	Trial D5	Trial D6	Average k_D^{pfo} (s^{-1})	Stdev	k_{2D} ($M^{-1}s^{-1}$)	Stdev [a]
45	1.2323	1.2273	1.2385	1.2468	1.2402	1.2291	1.2357	0.0075	2.06E+02	1.2441
35	0.8006	0.8219	0.8233	0.8245	0.8120	0.8141	0.8161	0.0091	1.36E+02	1.5201
25	0.5373	0.5391	0.5360	0.5380	0.5352	0.5347	0.5367	0.0017	8.95E+01	0.2819
15	0.3285	0.3322	0.3323	0.3337	0.3320	0.3327	0.3319	0.0018	5.53E+01	0.2947
5	0.2007	0.1983	0.2001	0.1999	0.1999	0.1996	0.1998	0.0008	3.33E+01	0.1316

[a] = (Stdev(for k^{pfo})/k^{pfo})*k_{2H}

Six kinetic runs together with the averaged data	First-order kinetic fit of the averaged data as an example

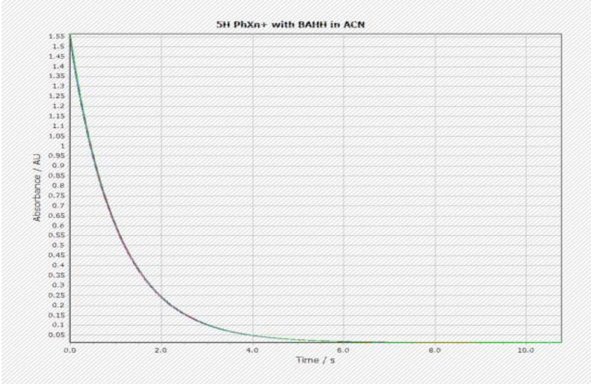

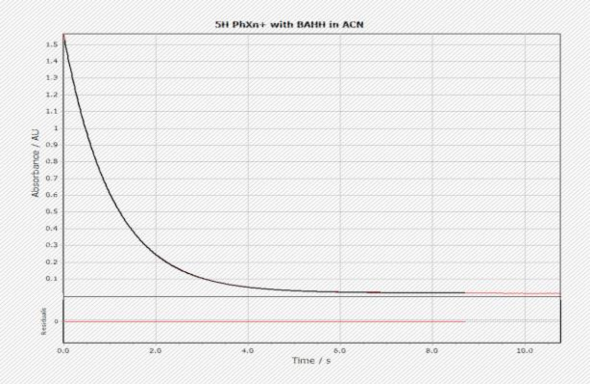

5°C H

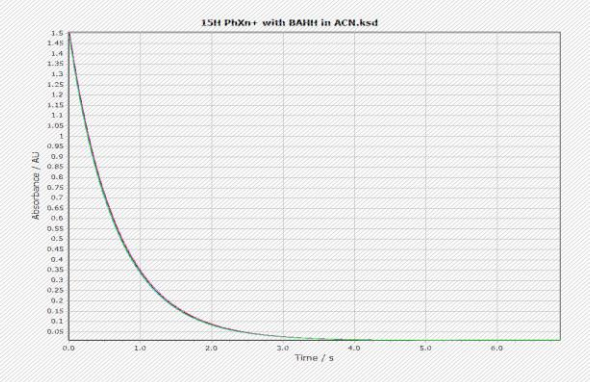

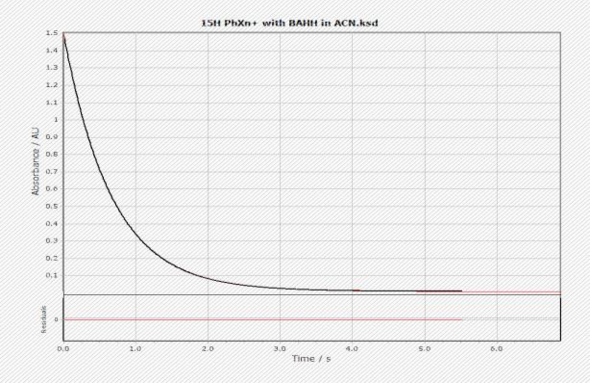

15°C H

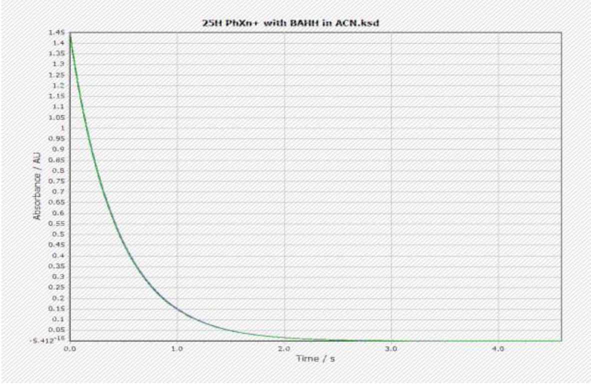

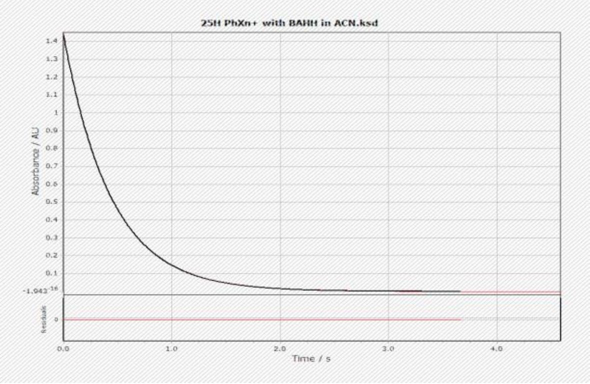

25°C H

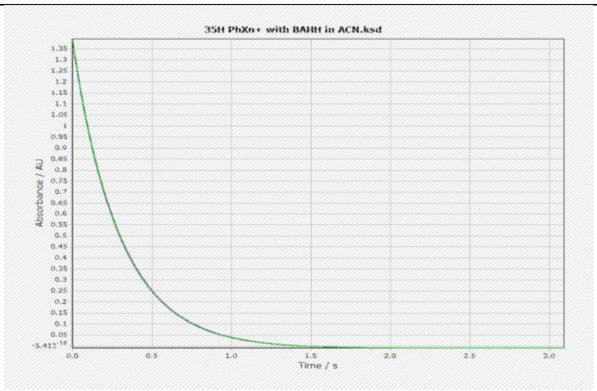

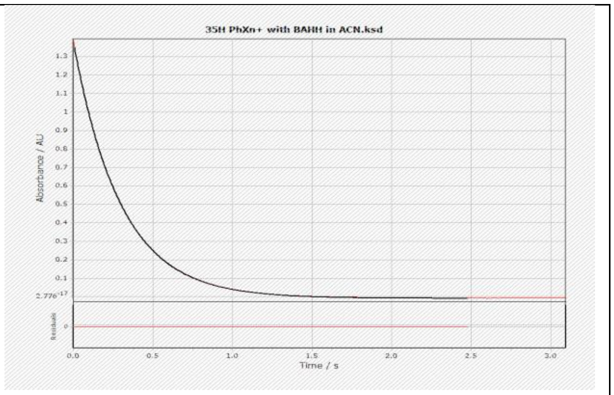

35°C H

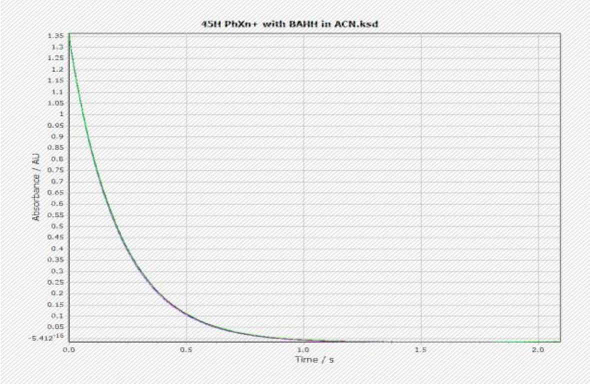

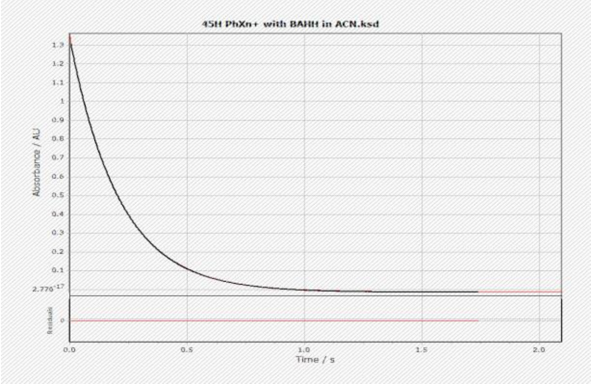

45°C H

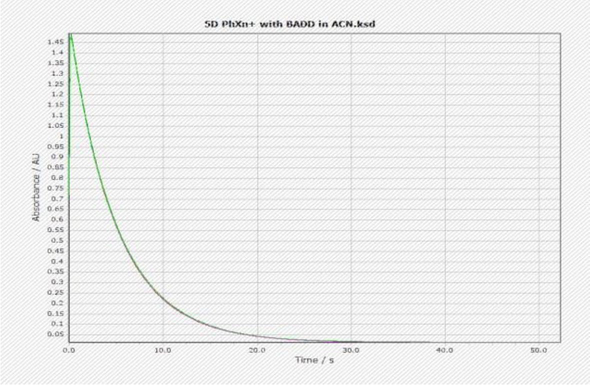

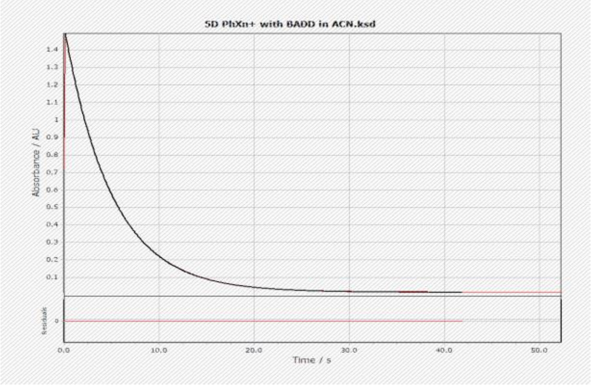

5°C D

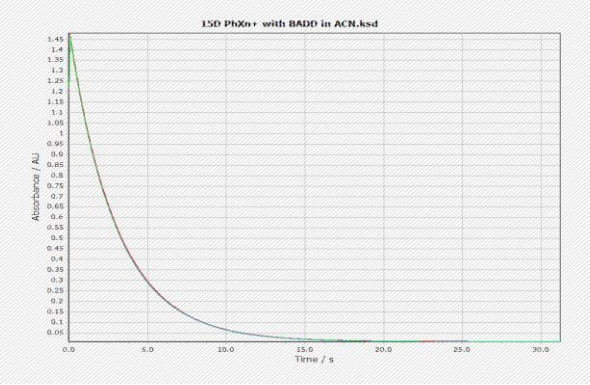

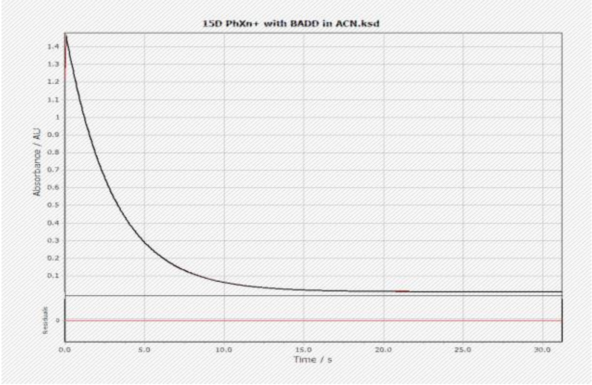

15°C D

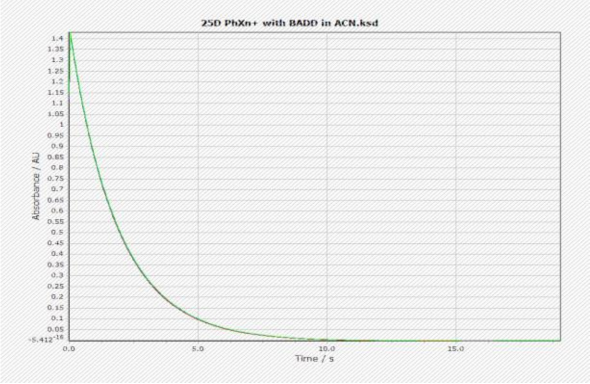

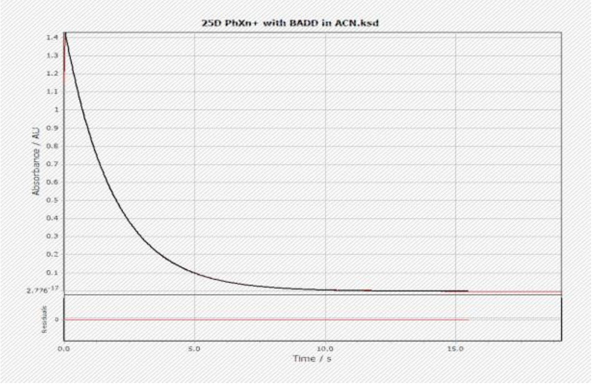

25°C D

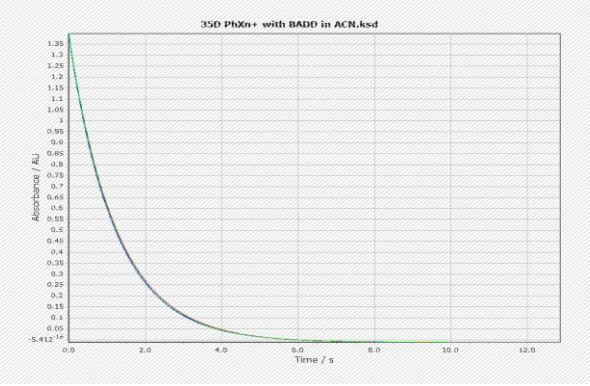

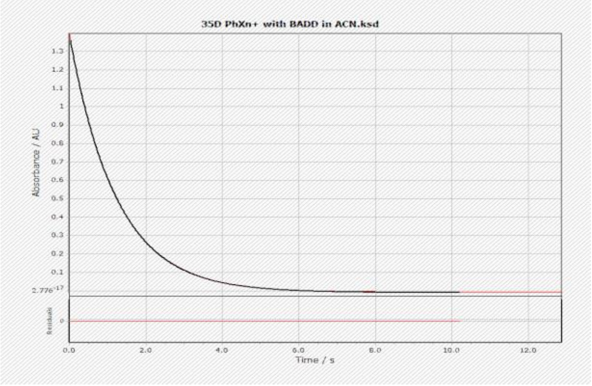

35°C D

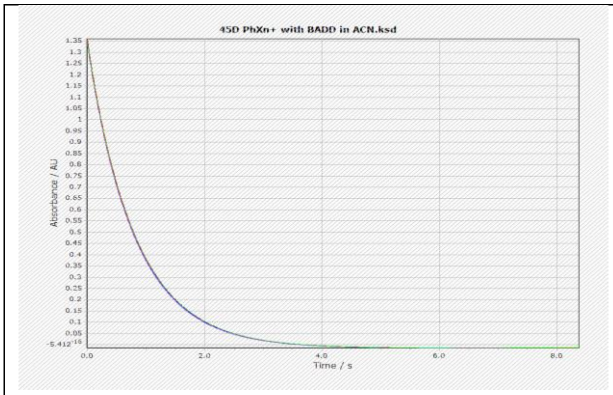	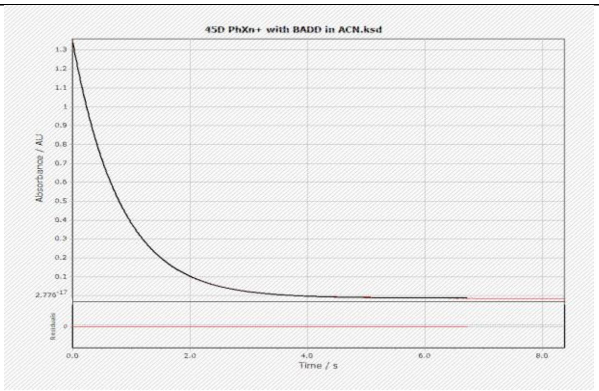
45℃ D	

Day 2 data (November 17, 2023)

	Pseudo-first-order rate constants k_H^{pfo} (s^{-1})									
**Temp (°C)**	**Trial H1**	**Trial H2**	**Trial H3**	**Trial H4**	**Trial H5**	**Trial H6**	**Average k_H^{pfo} (s^{-1})**	**Stdev**	**k_{2H} ($M^{-1}s^{-1}$)**	**Stdev [a]**
45	2.4738	2.4952	2.4924	2.4939	2.4636	2.5153	2.4890	0.0181	8.30E+02	6.0388
35	1.6893	1.6913	1.6871	1.6997	1.7091	1.7220	1.6998	0.0136	5.67E+02	4.5315
25	1.1453	1.1503	1.1577	1.1429	1.1557	1.1565	1.1514	0.0062	3.84E+02	2.0808
15	0.7612	0.7527	0.7540	0.7518	0.7568	0.7513	0.7546	0.0038	2.25E+02	1.2541
5	0.4896	0.4838	0.4812	0.4785	0.4867	0.4875	0.4846	0.0042	1.62E+02	1.3941
**Temp (°C)**	**Trial D1**	**Trial D2**	**Trial D3**	**Trial D4**	**Trial D5**	**Trial D6**	**Average k_D^{pfo} (s^{-1})**	**Stdev**	**k_{2D} ($M^{-1}s^{-1}$)**	**Stdev [a]**
45	0.6237	0.6371	0.6255	0.6243	0.6232	0.6282	0.6270	0.0053	2.09E+02	1.7564
35	0.4108	0.4046	0.4168	0.4115	0.4092	0.4068	0.4099	0.0042	1.37E+02	1.4110
25	0.2655	0.2620	0.2638	0.2653	0.2636	0.2638	0.2640	0.0013	8.80E+01	0.4222
15	0.1640	0.1641	0.1631	0.1639	0.1636	0.1630	0.1636	0.0005	5.45E+01	0.1540
5	0.1002	0.1001	0.1000	0.0990	0.0994	0.0999	0.0998	0.0005	3.33E+01	0.1587

[a] = (Stdev(for k^{pfo})/k^{pfo})*k_{2H}

Six kinetic runs together with the averaged data	First-order kinetic fit of the averaged data as an example
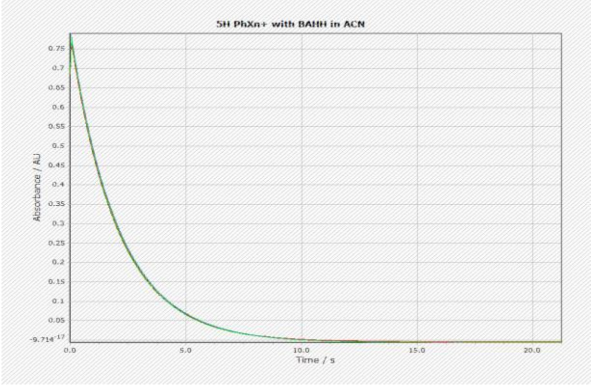	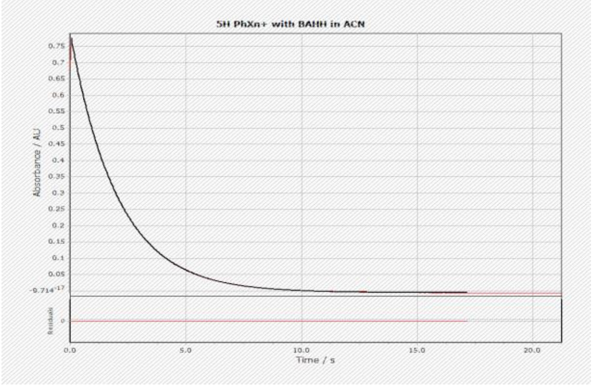
5℃ H	

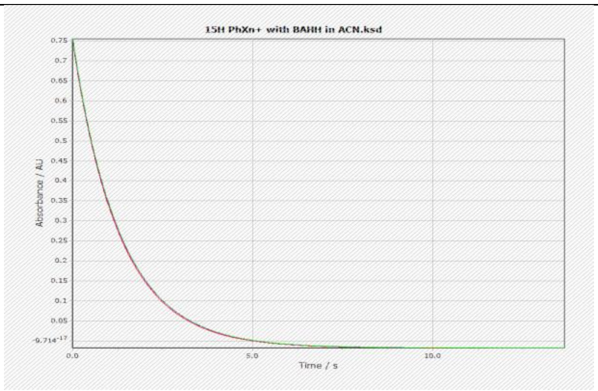
15H PhXn+ with BAHt in ACN.ksd
Absorbance / AU
Time / s

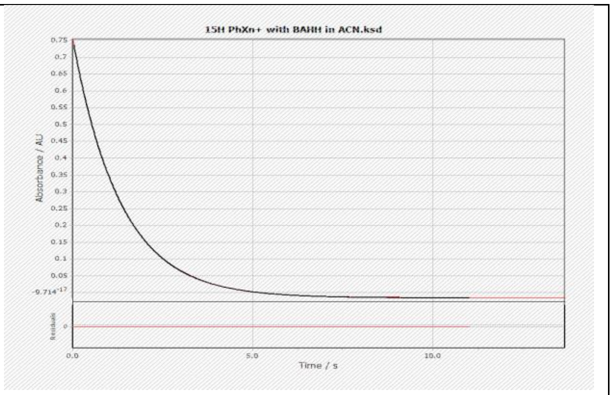
15H PhXn+ with BAHt in ACN.ksd
Absorbance / AU
Residuals
Time / s

15℃ H

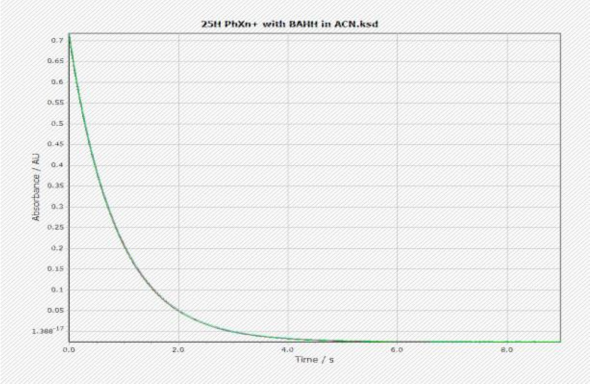
25H PhXn+ with BAHt in ACN.ksd
Absorbance / AU
Time / s

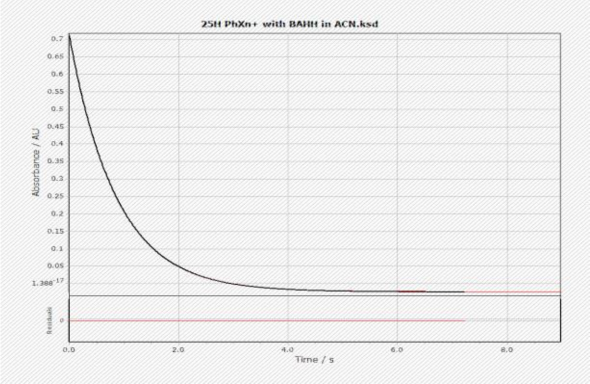
25H PhXn+ with BAHt in ACN.ksd
Absorbance / AU
Residuals
Time / s

25℃ H

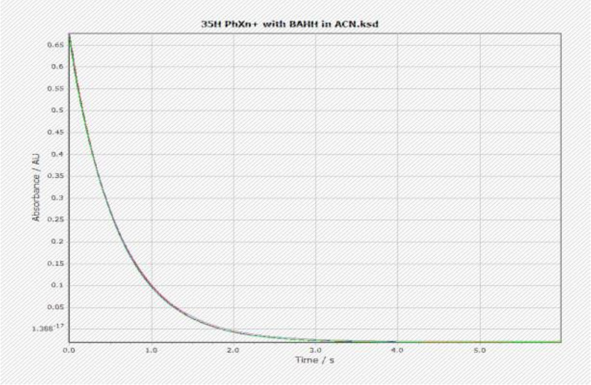
35H PhXn+ with BAHt in ACN.ksd
Absorbance / AU
Time / s

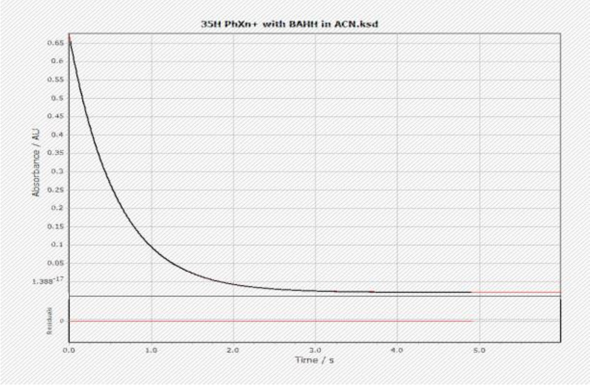
35H PhXn+ with BAHt in ACN.ksd
Absorbance / AU
Residuals
Time / s

35℃ H

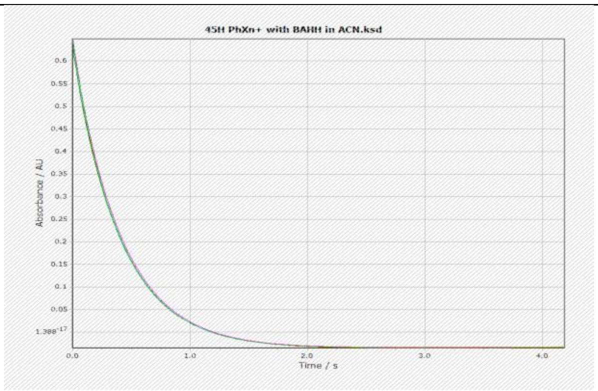

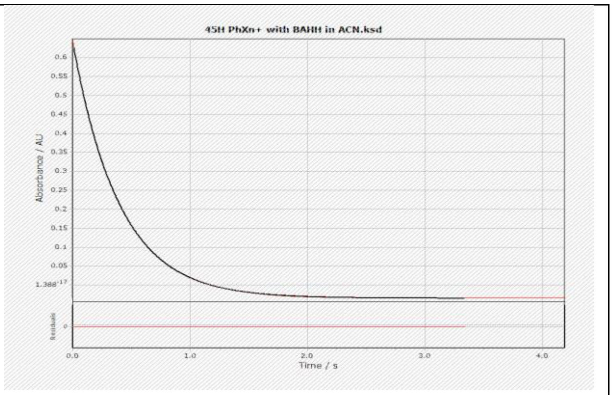

45°C H

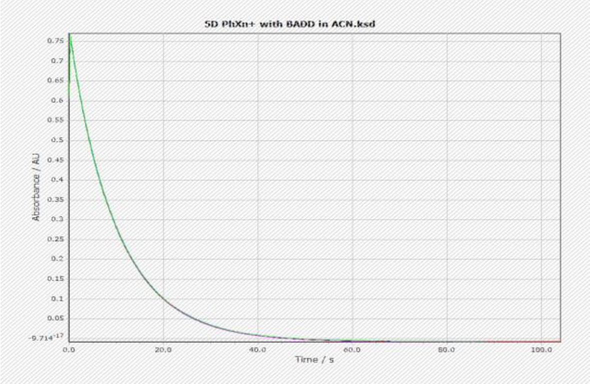

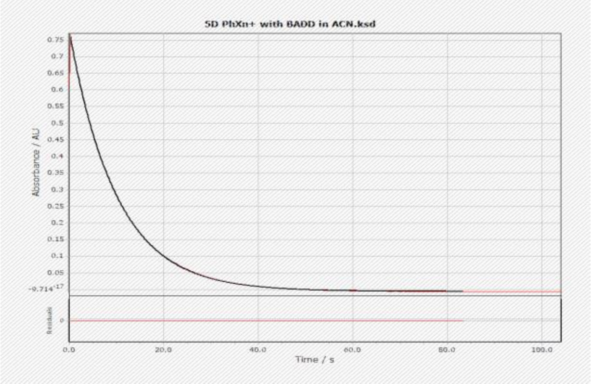

5°C D

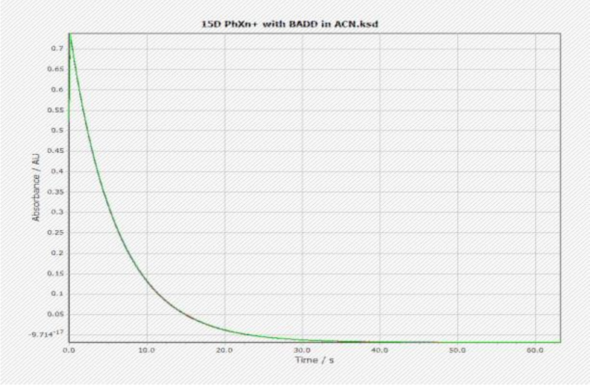

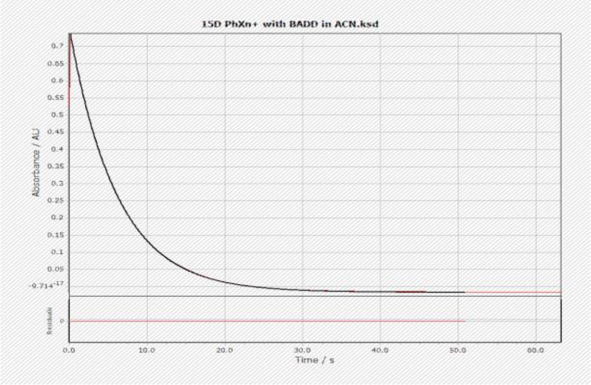

15°C D

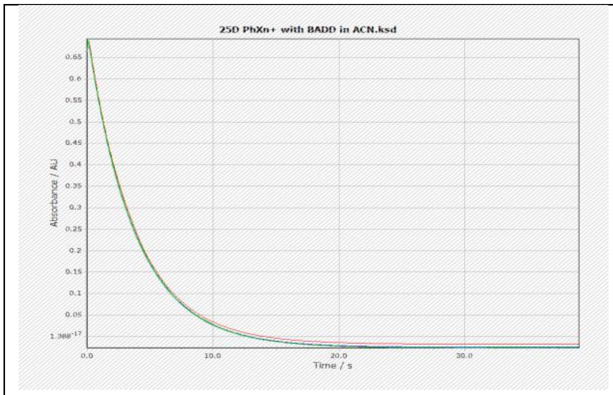

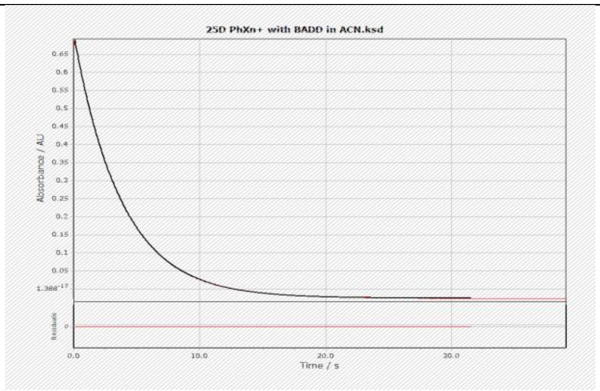

25°C D

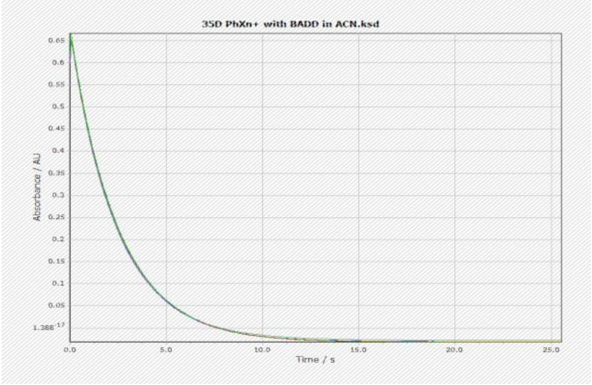

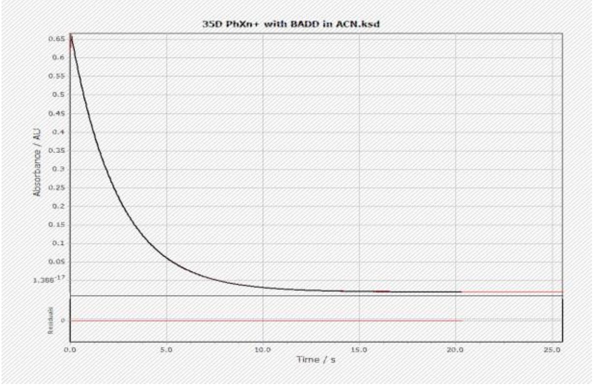

35°C D

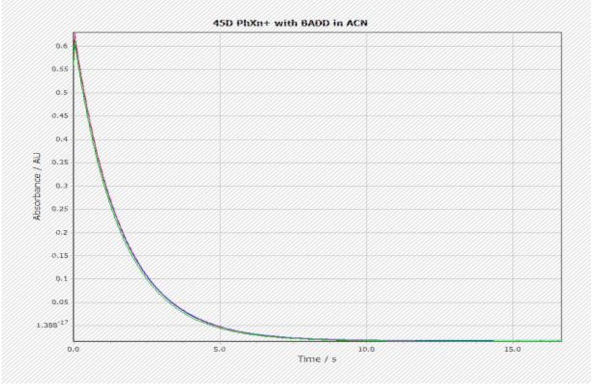

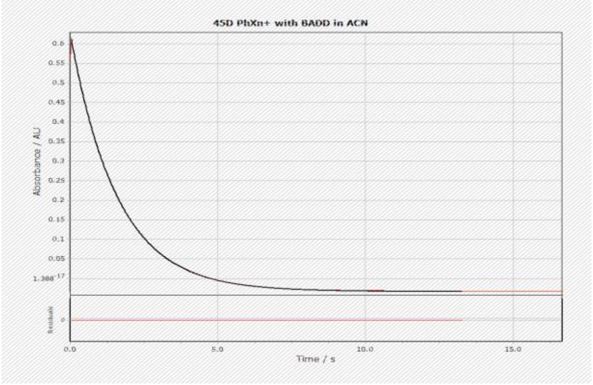

45°C D

Day 3 data (November 27, 2023)

	Pseudo-first-order rate constants k_H^{pfo} (s^{-1})									
**Temp (°C)**	**Trial H1**	**Trial H2**	**Trial H3**	**Trial H4**	**Trial H5**	**Trial H6**	**Average k_H^{pfo} (s^{-1})**	**Stdev**	**k_{2H} ($M^{-1}s^{-1}$)**	**Stdev [a]**
45	2.4210	2.4243	2.4322	2.4550	2.4209	2.4659	2.4365	0.0196	8.12E+02	6.4310
35	1.6911	1.7080	1.6798	1.6926	1.7135	1.6921	1.6962	0.0124	5.65E+02	4.1236
25	1.1325	1.1198	1.1292	1.1315	1.1216	1.1231	1.1263	0.0055	3.75E+02	1.8228
15	0.7441	0.7472	0.7427	0.7483	0.7523	0.7428	0.7462	0.0038	2.49E+02	1.2517
5	0.4782	0.4785	0.4820	0.4782	0.4804	0.4784	0.4793	0.0016	1.60E+02	0.5276

Temp (°C)	Trial D1	Trial D2	Trial D3	Trial D4	Trial D5	Trial D6	Average k_D^{pfo} (s^{-1})	Stdev	k_{2D} ($M^{-1}s^{-1}$)	Stdev [a]
45	0.6289	0.6383	0.6239	0.6356	0.6372	0.6253	0.6315	0.0063	2.11E+02	2.1015
35	0.4127	0.4147	0.4136	0.4154	0.4109	0.4129	0.4134	0.0016	1.38E+02	0.5359
25	0.2704	0.2696	0.2693	0.2696	0.2679	0.2661	0.2688	0.0016	8.96E+01	0.5275
15	0.1679	0.1674	0.1675	0.1670	0.1676	0.1670	0.1674	0.0003	5.58E+01	0.1092
5	0.1015	0.1018	0.1012	0.1014	0.1016	0.1012	0.1014	0.0002	3.38E+01	0.0803

[a] = (Stdev(for k^{pfo})/k^{pfo})*k_{2H}

<table>
<tr><td>Six kinetic runs together with the averaged data</td><td>First-order kinetic fit of the averaged data as an example</td></tr>
<tr><td>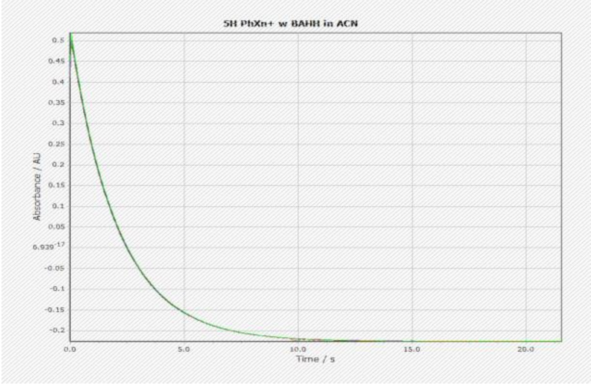
</td><td>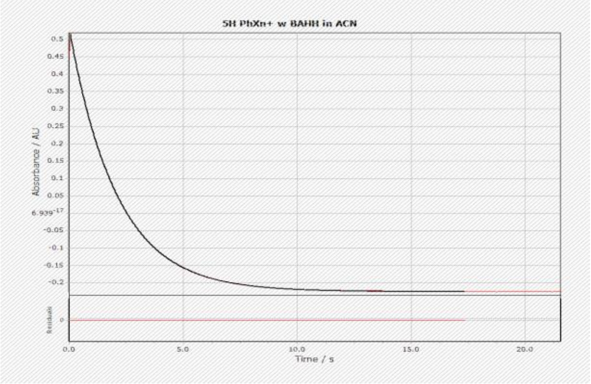
</td></tr>
<tr><td colspan="2">5°C H</td></tr>
<tr><td>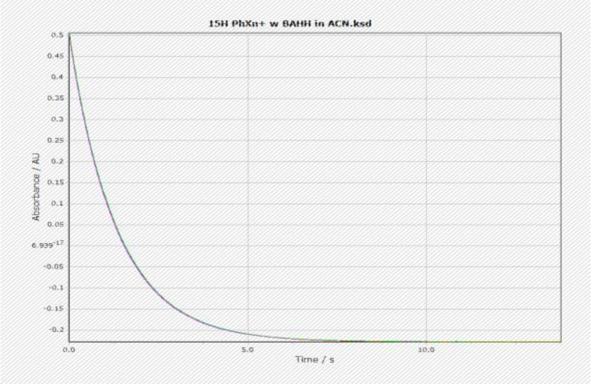
</td><td>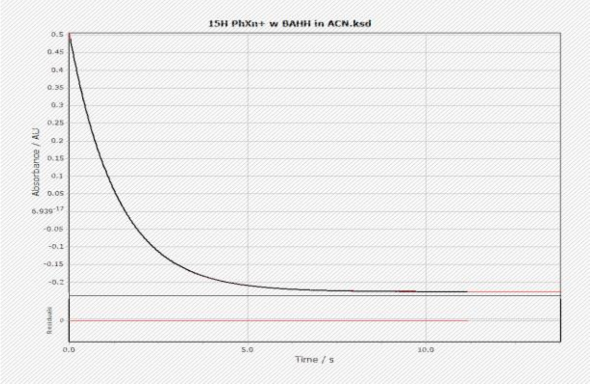
</td></tr>
<tr><td colspan="2">15°C H</td></tr>
<tr><td>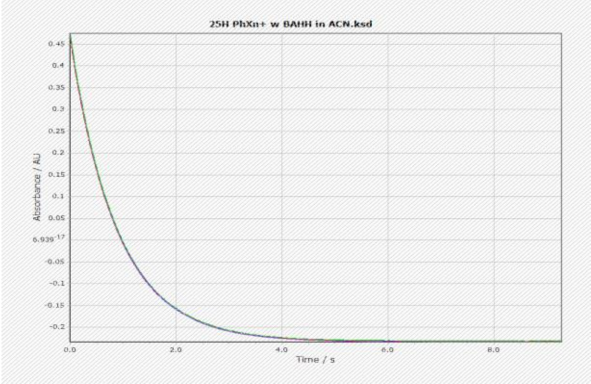
</td><td>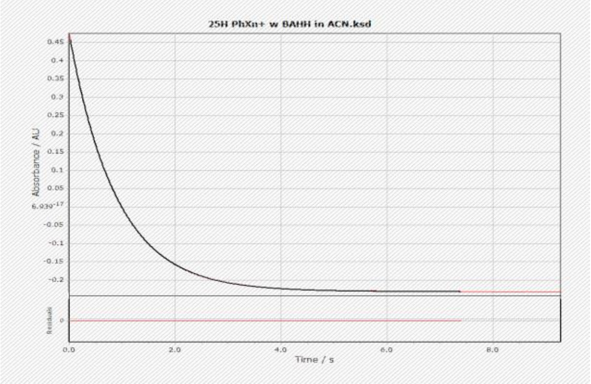
</td></tr>
<tr><td colspan="2">25°C H</td></tr>
</table>

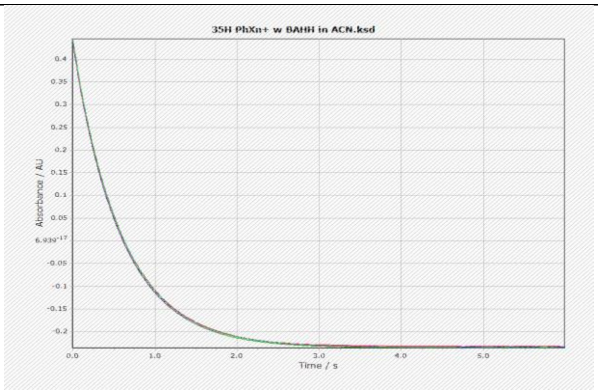

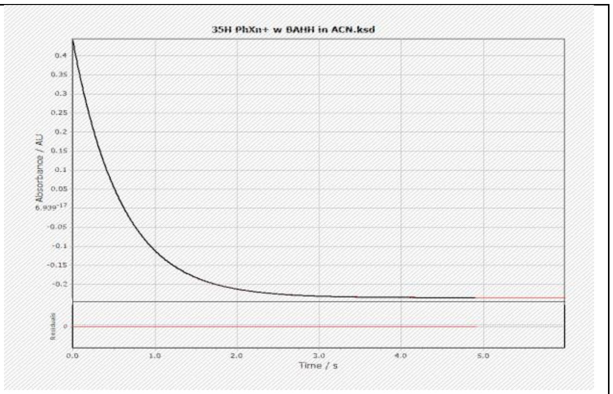

35°C H

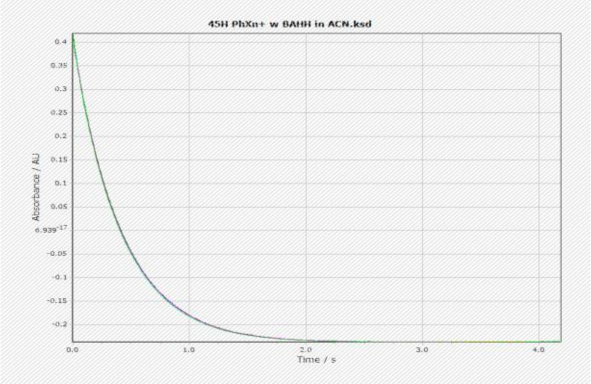

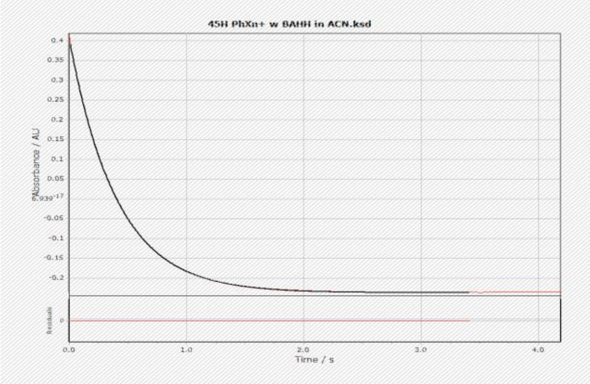

45°C H

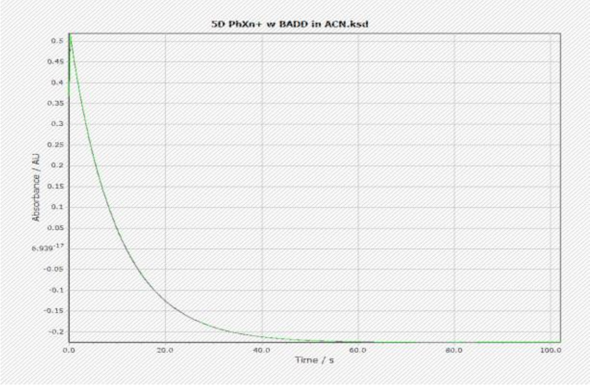

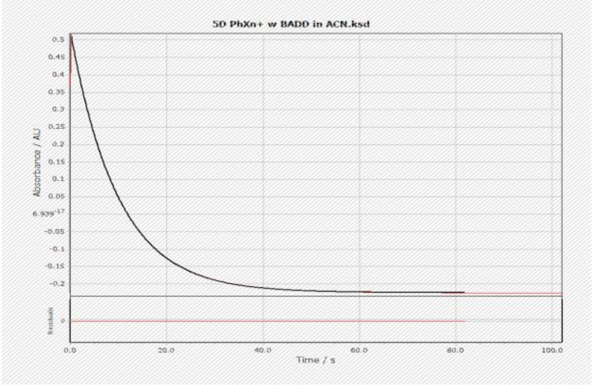

5°C D

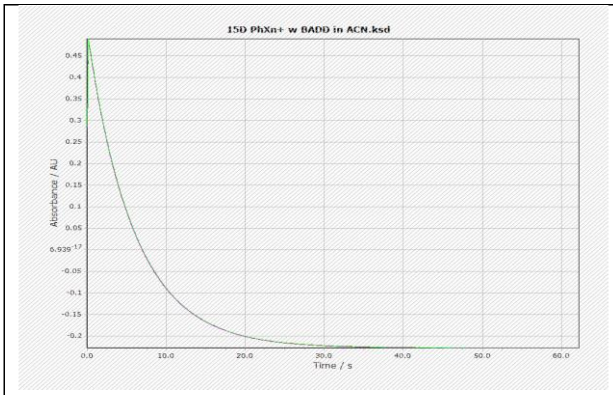

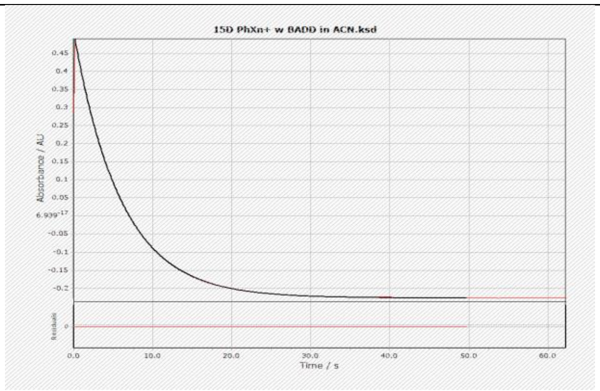

15°C D

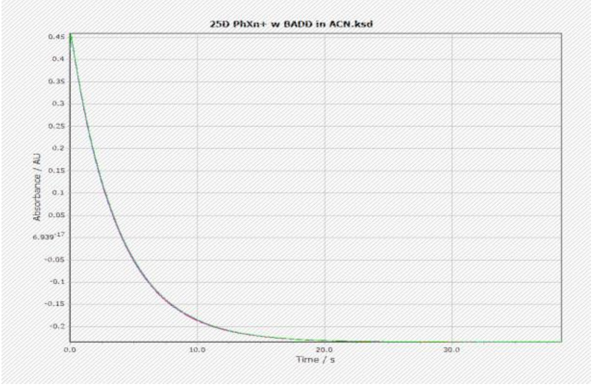

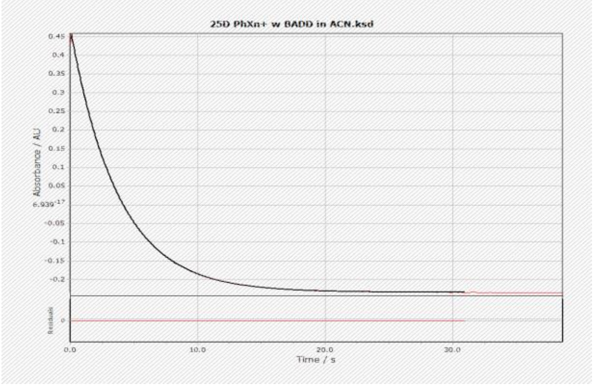

25°C D

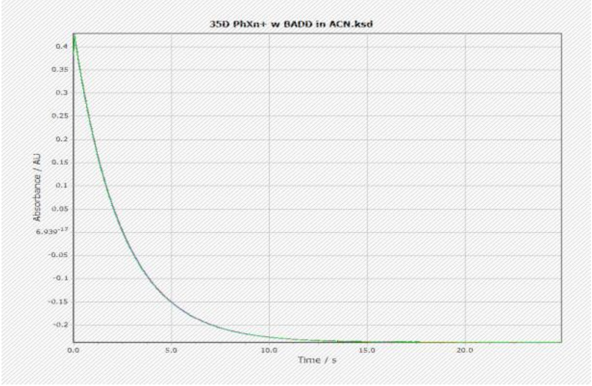

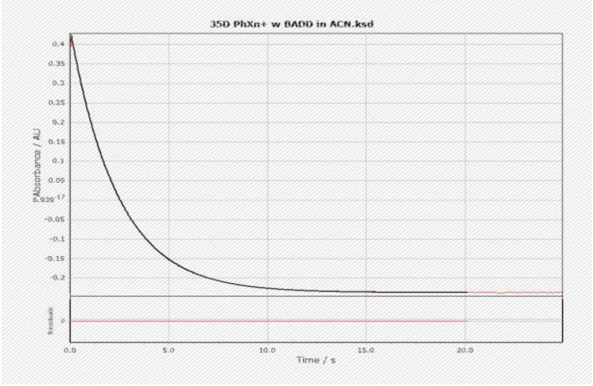

35°C D

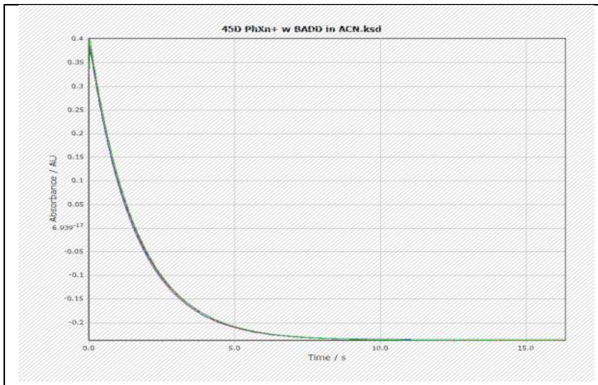

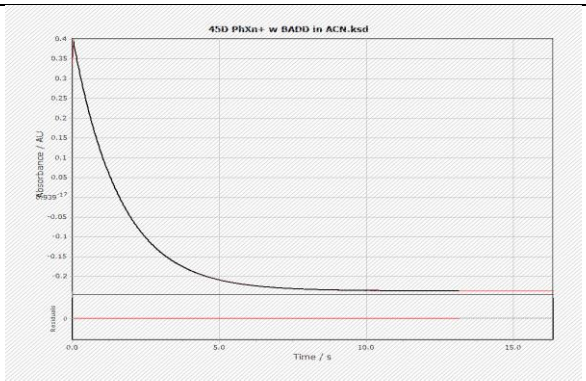

45℃ D

Primary kinetic data for the rate constants in Table S4

Day 1 data (September 11, 2024)

Temp (°C)	Trial H1	Trial H2	Trial H3	Trial H4	Trial H5	Trial H6	Average k_H^{pfo} (s^{-1})	Stdev	k_{2H} ($M^{-1}s^{-1}$)	Stdev [a]
	Pseudo-first-order rate constants k^{pfo} (s^{-1})									
45	2.0071	1.9785	2.0230	2.0162	2.0278	2.0366	2.0149	0.0205	6.72E+02	6.8220
35	1.3981	1.3883	1.4022	1.3898	1.4037	1.4145	1.3994	0.0097	4.66E+02	3.2331
25	0.9663	0.9576	0.9600	0.9728	0.9668	0.9568	0.9634	0.0063	3.21E+02	2.0901
15	0.6466	0.6507	0.6488	0.6486	0.6487	0.6562	0.6499	0.0033	2.17E+02	1.1117
5	0.4276	0.4228	0.4260	0.4250	0.4287	0.4269	0.4262	0.0021	1.42E+02	0.6877

Temp (°C)	Trial D1	Trial D2	Trial D3	Trial D4	Trial D5	Trial D6	Average k_D^{pfo} (s^{-1})	Stdev	k_{2D} ($M^{-1}s^{-1}$)	Stdev [a]
45	0.4576	0.4578	0.4631	0.4555	0.4558	0.4601	0.4583	0.0029	1.53E+02	0.9536
35	0.3059	0.3040	0.3000	0.3046	0.3049	0.3019	0.3035	0.0022	1.01E+02	0.7286
25	0.1999	0.1999	0.1978	0.1989	0.1980	0.1997	0.1990	0.0010	6.63E+01	0.3199
15	0.1247	0.1252	0.1262	0.1245	0.1264	0.1249	0.1253	0.0008	4.18E+01	0.2655
5	0.0761	0.0764	0.0766	0.0770	0.0766	0.0765	0.0765	0.0003	2.55E+01	0.1001

[a] = (Stdev(for k^{pfo})/k^{pfo})*k_{2H}

Six kinetic runs together with the averaged data	First-order kinetic fit of the averaged data as an example

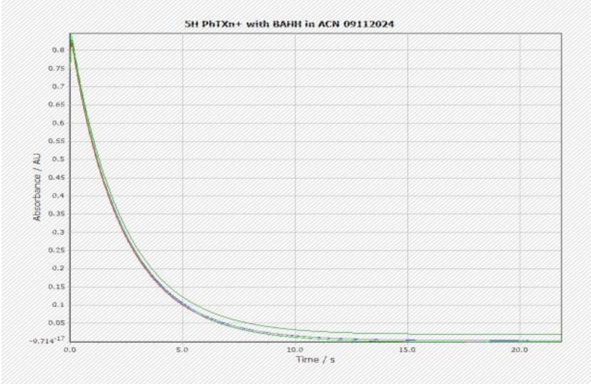

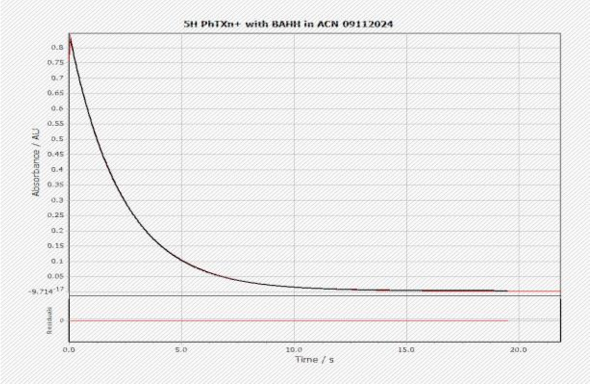

5℃ H

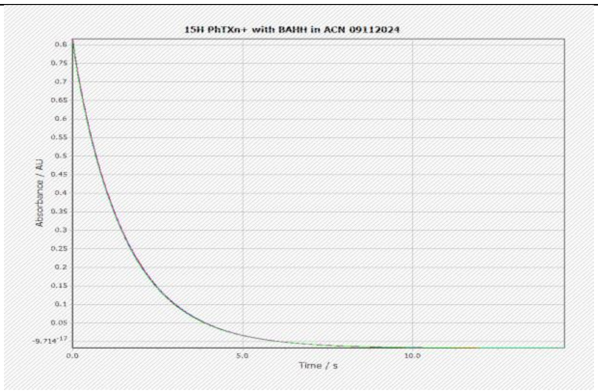

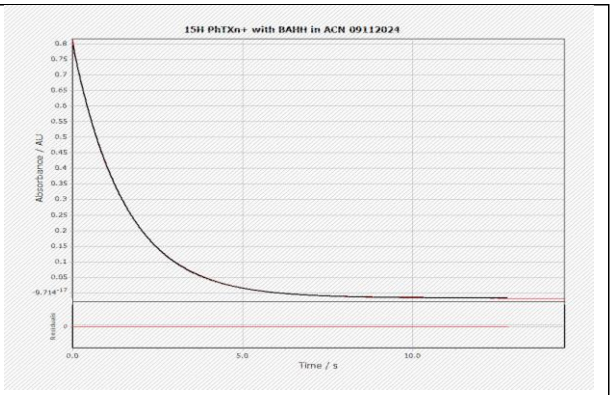

15°C H

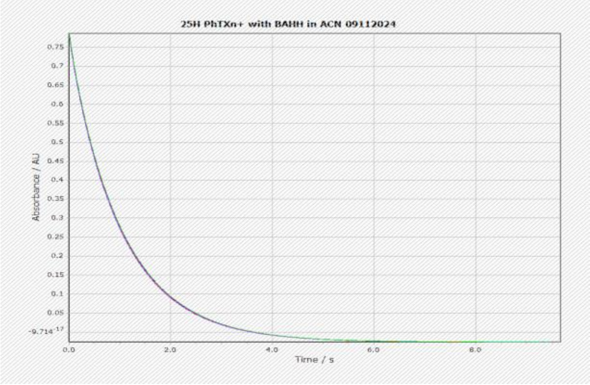

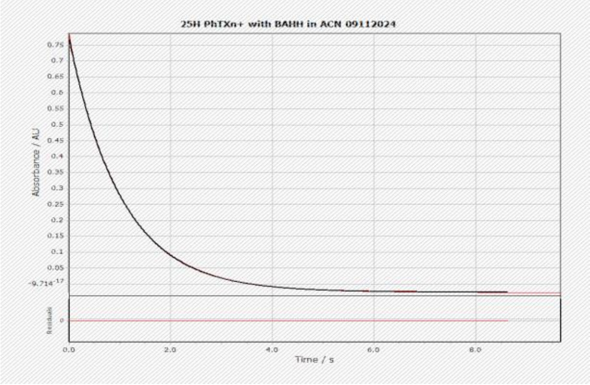

25°C H

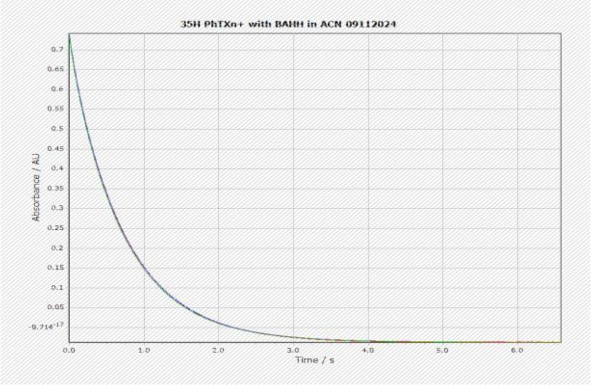

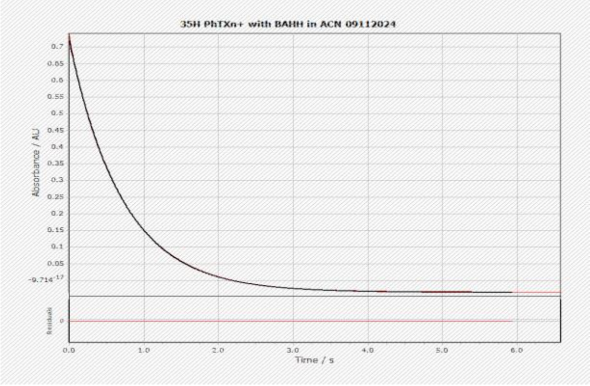

35°C H

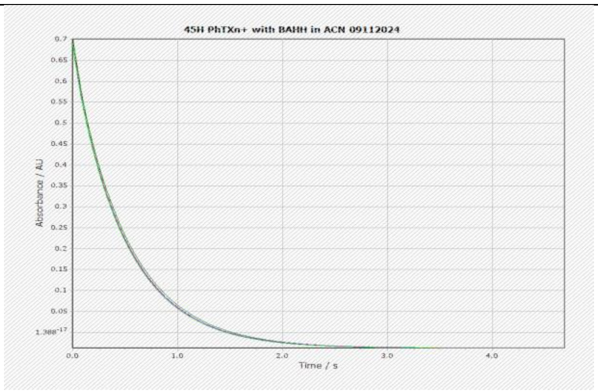

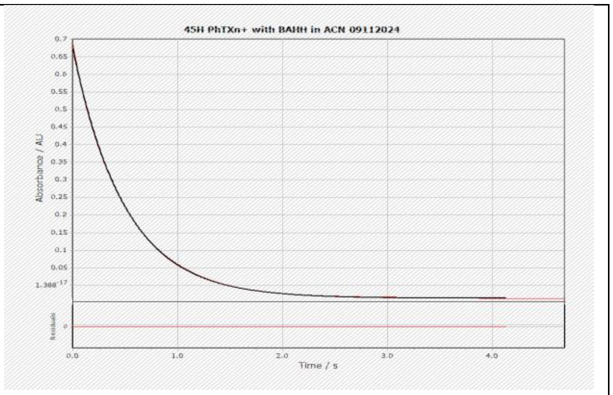

45°C H

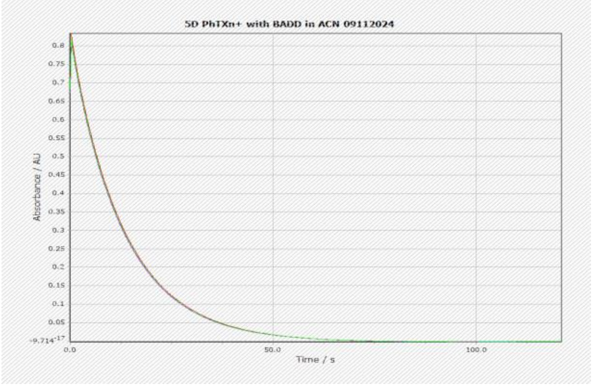

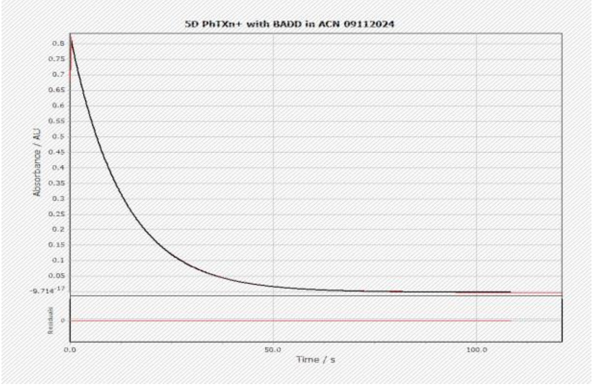

5°C D

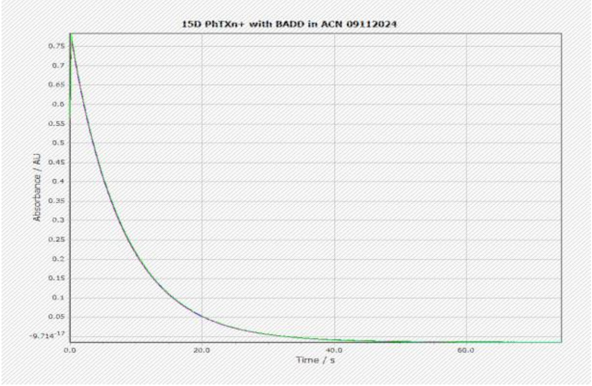

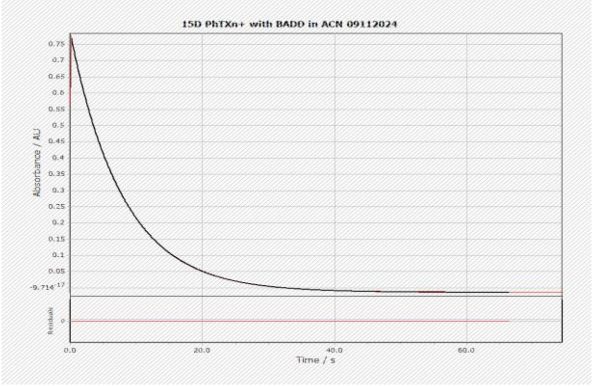

15°C D

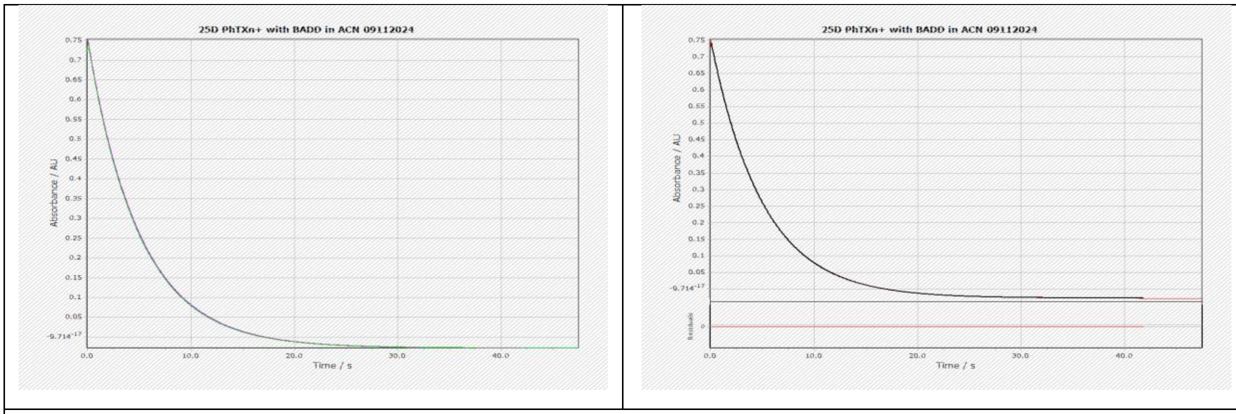

25°C D

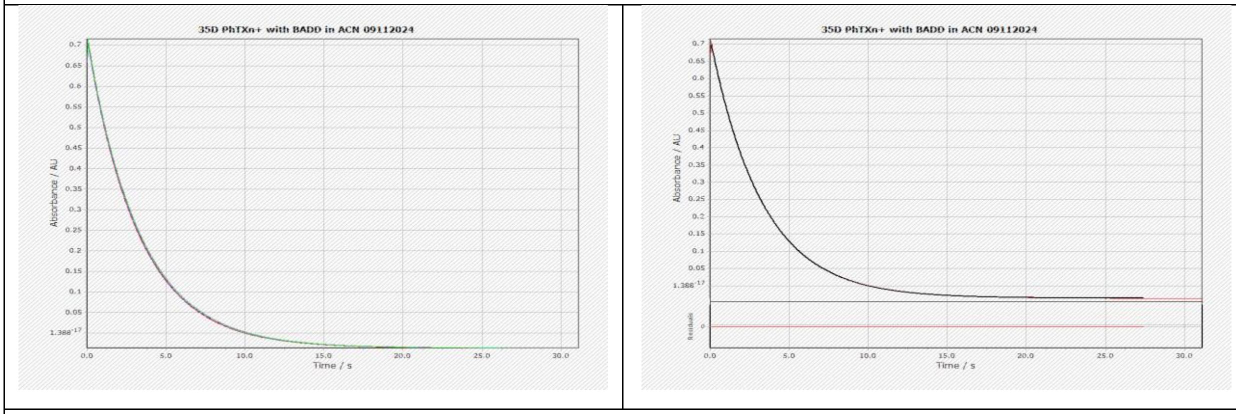

35°C D

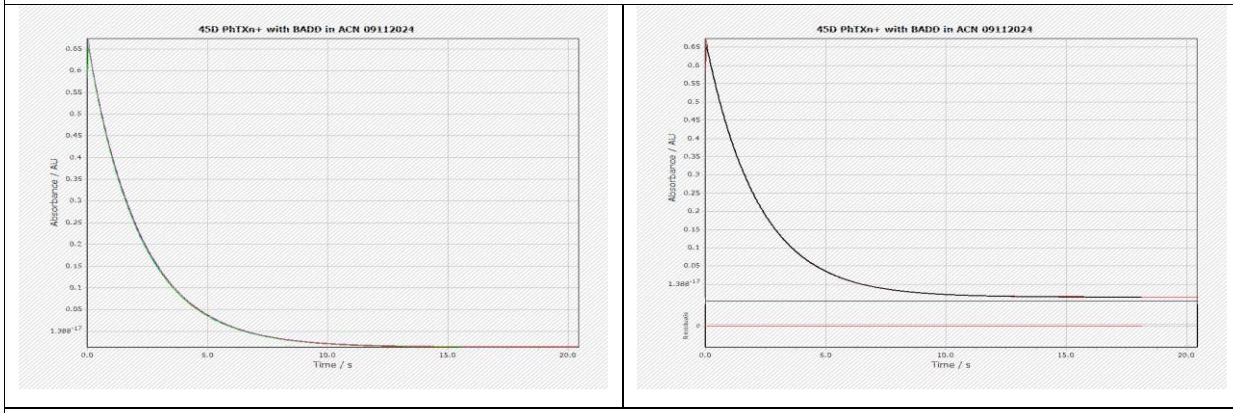

45°C D

Day 2 data (September 25, 2024)

	Pseudo-first-order rate constants k^{pfo} (s^{-1})									
**Temp (°C)**	**Trial H1**	**Trial H2**	**Trial H3**	**Trial H4**	**Trial H5**	**Trial H6**	**Average k_H^{pfo} (s^{-1})**	**Stdev**	**k_{2H} ($M^{-1}s^{-1}$)**	**Stdev [a]**
45	1.9291	1.9275	1.9340	1.9712	1.9641	1.9578	1.9473	0.0193	6.49E+02	6.4345
35	1.3577	1.3696	1.3498	1.3608	1.3558	1.3309	1.3541	0.0131	4.51E+02	4.3606
25	0.9290	0.9281	0.9237	0.9310	0.9280	0.9386	0.9297	0.0050	3.10E+02	1.6520
15	0.6290	0.6299	0.6329	0.6298	0.6268	0.6255	0.6290	0.0026	2.10E+02	0.8622
5	0.4067	0.4073	0.4079	0.4093	0.4059	0.4083	0.4075	0.0012	1.36E+02	0.4064

Temp (°C)	Trial D1	Trial D2	Trial D3	Trial D4	Trial D5	Trial D6	Average k_D^{pfo} (s^{-1})	Stdev	k_{2D} ($M^{-1}s^{-1}$)	Stdev [a]
45	0.4475	0.4437	0.4477	0.4398	0.4442	0.4438	0.4444	0.0029	1.48E+02	0.9768
35	0.2971	0.2960	0.3008	0.3013	0.2981	0.2962	0.2982	0.0023	9.94E+01	0.7657
25	0.1966	0.1954	0.1972	0.1948	0.1929	0.1931	0.1950	0.0018	6.50E+01	0.5864
15	0.1218	0.1225	0.1223	0.1218	0.1225	0.1214	0.1221	0.0004	4.07E+01	0.1494
5	0.0745	0.0740	0.0743	0.0748	0.0743	0.0740	0.0743	0.0003	2.48E+01	0.1055

[a] = (Stdev(for k^{pfo})/k^{pfo})*k_{2H}

Six kinetic runs together with the averaged data	First-order kinetic fit of the averaged data as an example
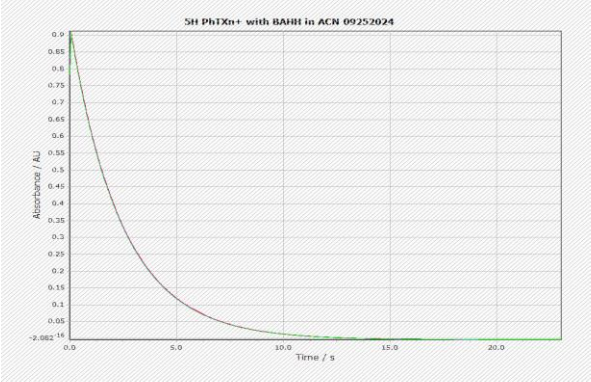	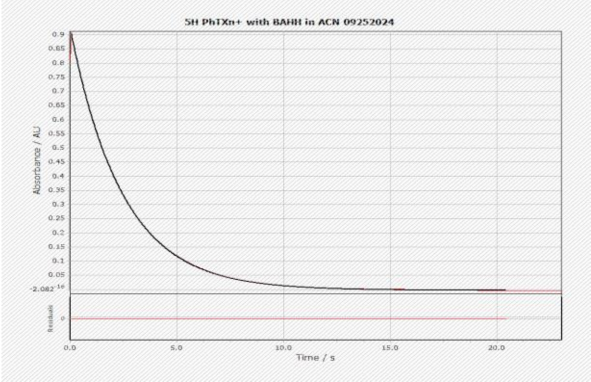

5℃ H

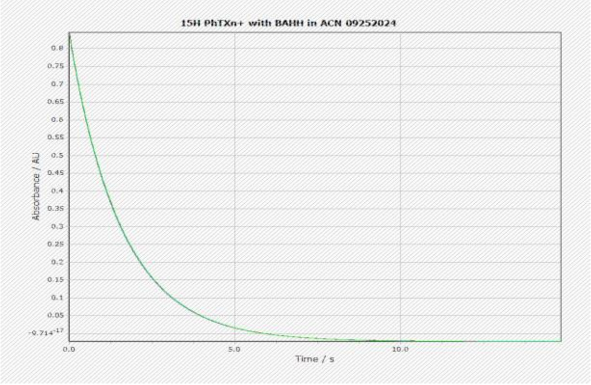	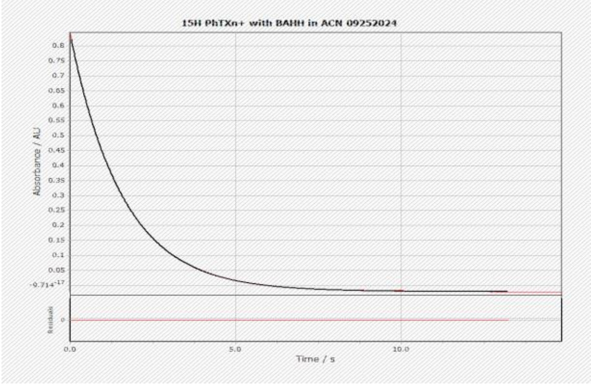

15℃ H

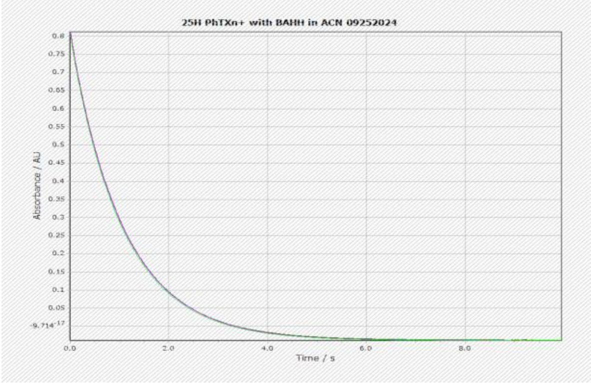	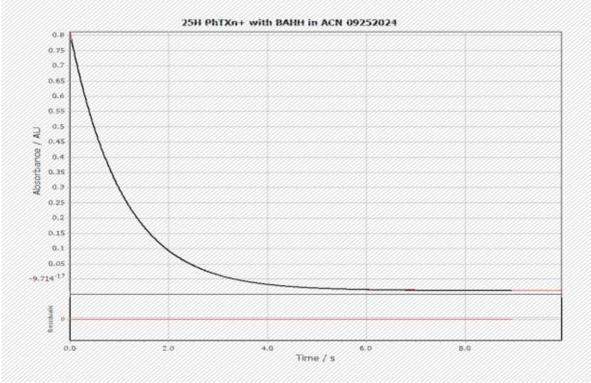

25℃ H

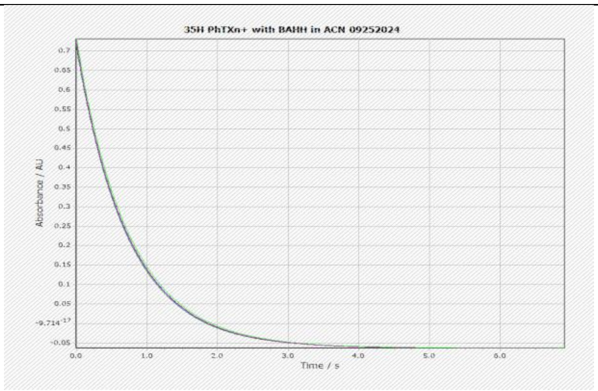

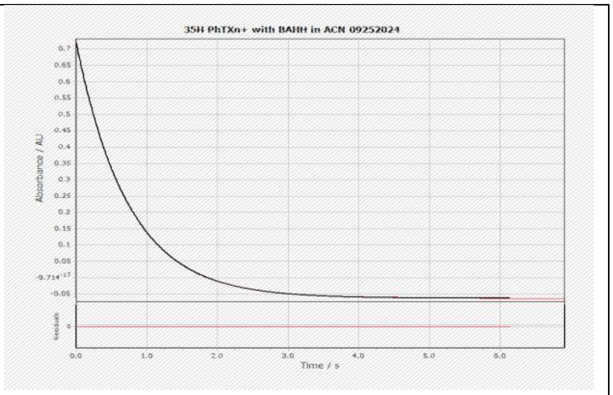

35°C H

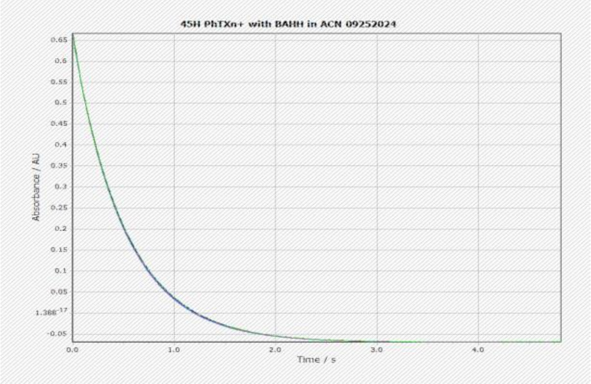

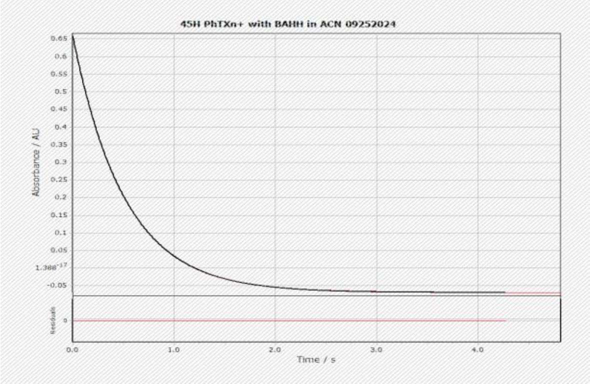

45°C H

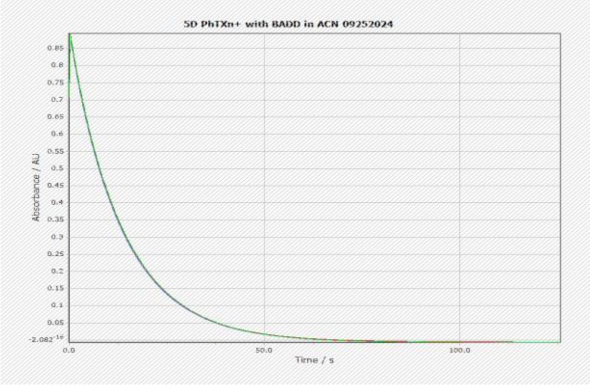

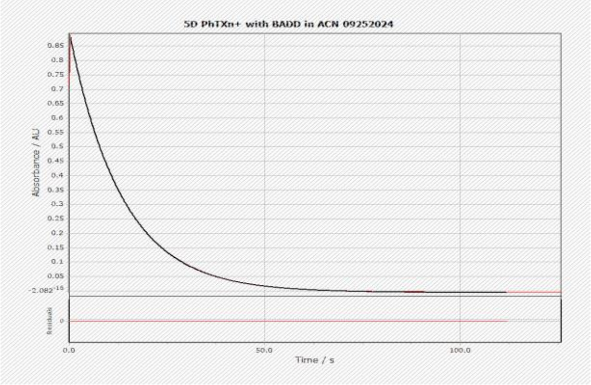

5°C D

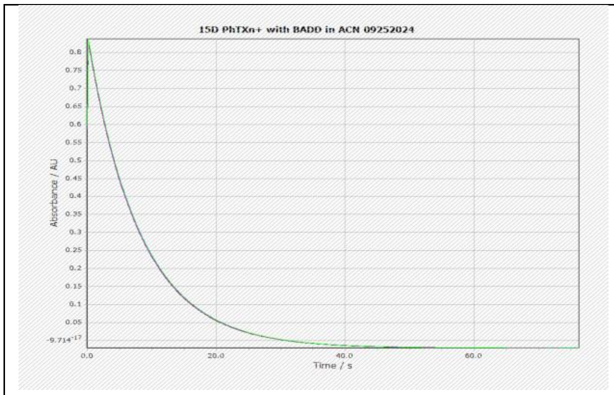

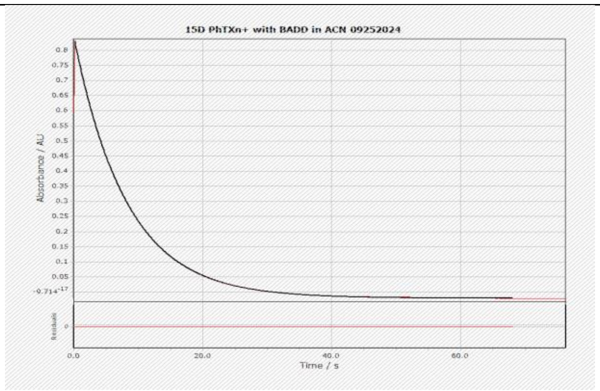

15°C D

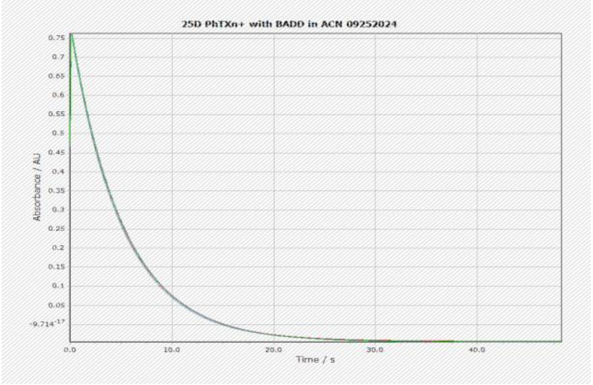

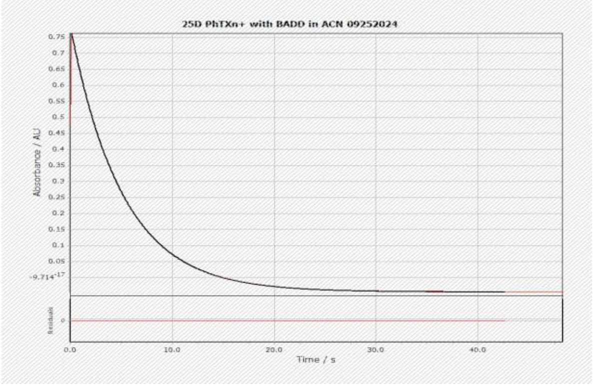

25°C D

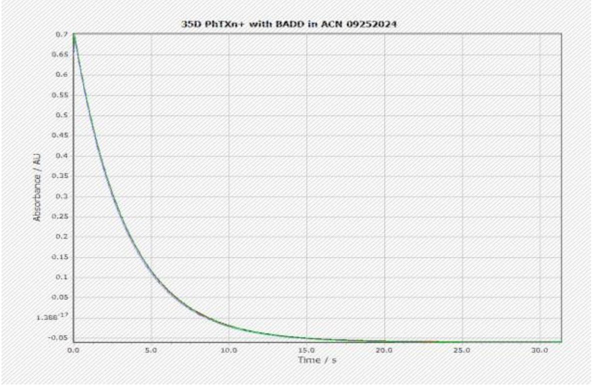

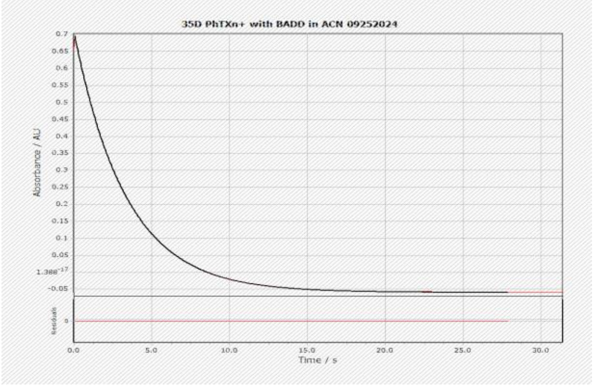

35°C D

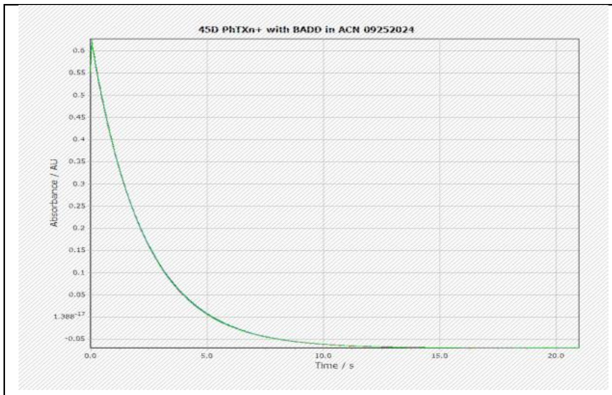 	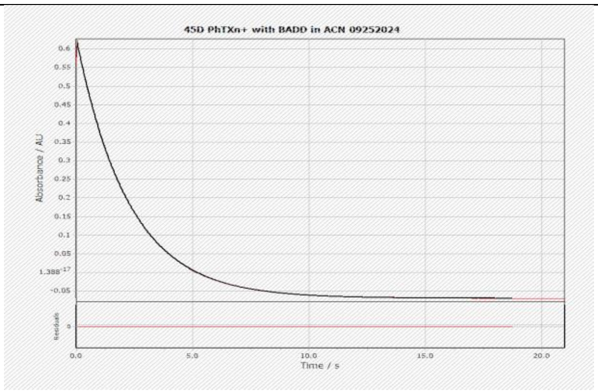
45℃ D	

Day 3 data (September 27, 2024)

Pseudo-first-order rate constants

**Temp (°C)**	k^{pfo} (s^{-1}) **Trial H1**	**Trial H2**	**Trial H3**	**Trial H4**	**Trial H5**	**Trial H6**	**Average k_H^{pfo} (s^{-1})**	**Stdev**	**k_{2H} (M^{-1}s^{-1})**	**Stdev [a]**
45	1.9615	1.9773	1.9541	1.9708	1.9768	1.9491	1.9649	0.0119	6.55E+02	3.9681
35	1.3531	1.3582	1.3679	1.3662	1.3846	1.3452	1.3625	0.0137	4.54E+02	4.5659
25	0.9568	0.9639	0.9594	0.9603	0.9710	0.9583	0.9616	0.0052	3.21E+02	1.7285
15	0.6410	0.6308	0.6356	0.6371	0.6346	0.6403	0.6366	0.0038	2.12E+02	1.2626
5	0.4153	0.4130	0.4099	0.4122	0.4126	0.4101	0.4122	0.0020	1.37E+02	0.6645

**Temp (°C)**	**Trial D1**	**Trial D2**	**Trial D3**	**Trial D4**	**Trial D5**	**Trial D6**	**Average k_D^{pfo} (s^{-1})**	**Stdev**	**k_{2D} (M^{-1}s^{-1})**	**Stdev [a]**
45	0.4491	0.4470	0.4432	0.4456	0.4452	0.4439	0.4457	0.0021	1.49E+02	0.7159
35	0.2991	0.3024	0.2998	0.1970	0.3004	0.3002	0.3006	0.0012	1.00E+02	0.4031
25	0.1987	0.1970	0.1966	0.1970	0.1947	0.1949	0.1965	0.0015	6.55E+01	0.5026
15	0.1217	0.1223	0.1229	0.1218	0.1229	0.1221	0.1223	0.0005	4.08E+01	0.1718
5	0.0743	0.0738	0.0742	0.0739	0.0748	0.0738	0.0742	0.0004	2.47E+01	0.1204

[a] = (Stdev(for k^{pfo})/k^{pfo})*k_{2H}

Six kinetic runs together with the averaged data	First-order kinetic fit of the averaged data as an example
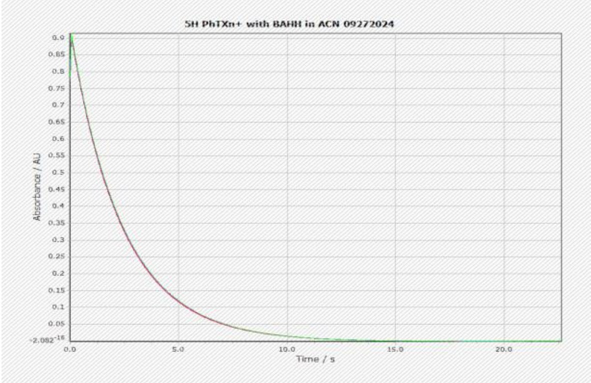 	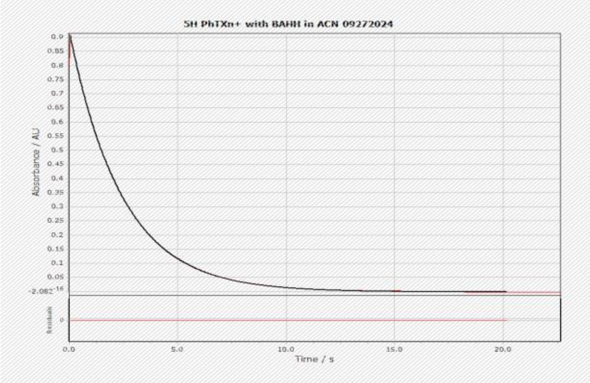
5℃ H	

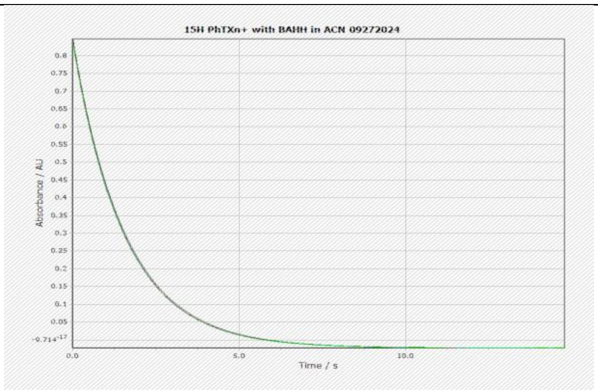

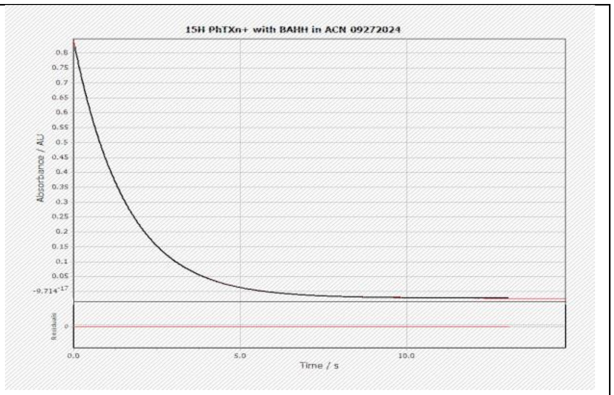

15°C H

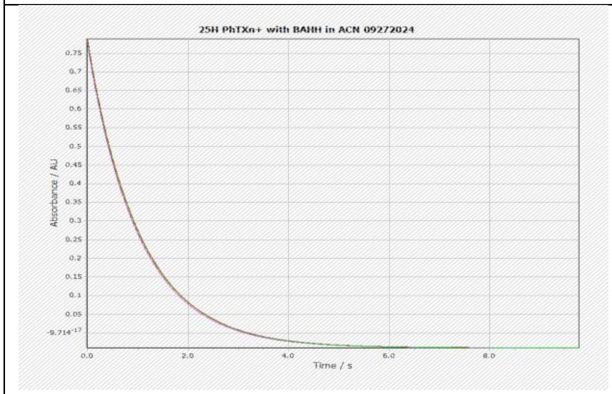

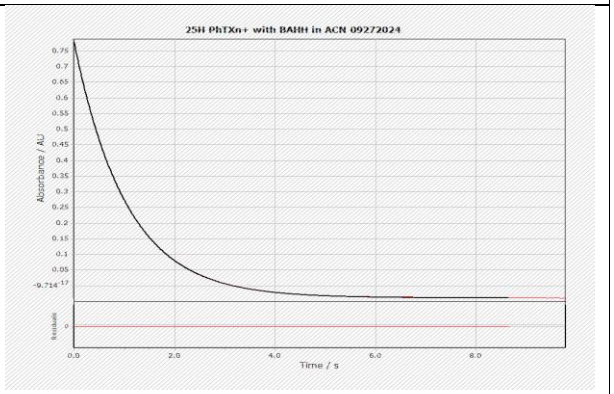

25°C H

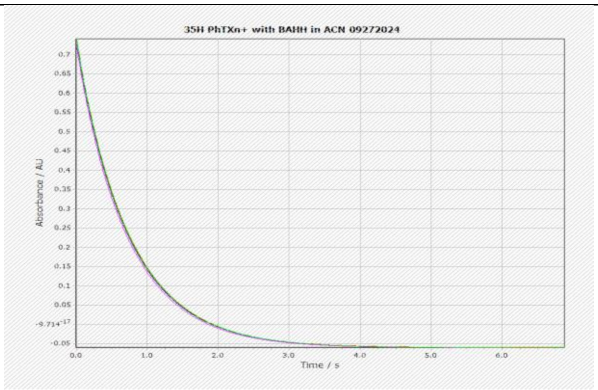

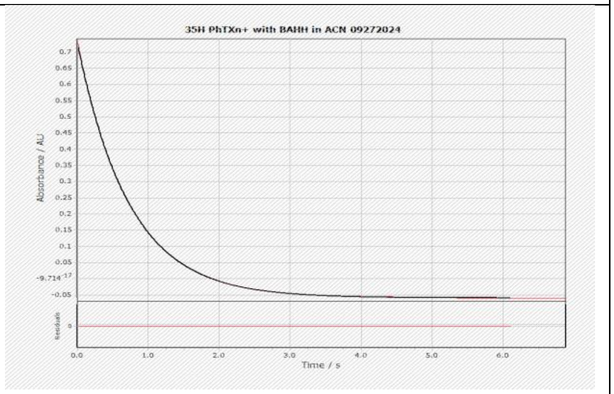

35°C H

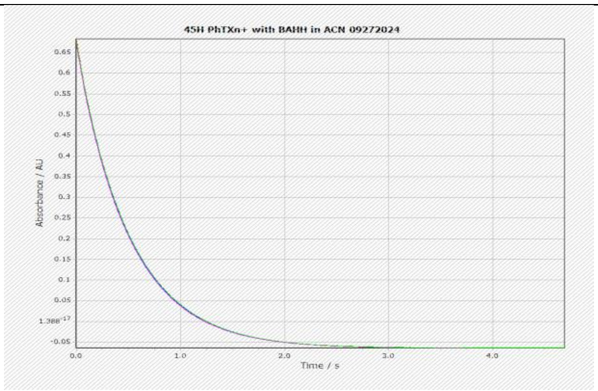

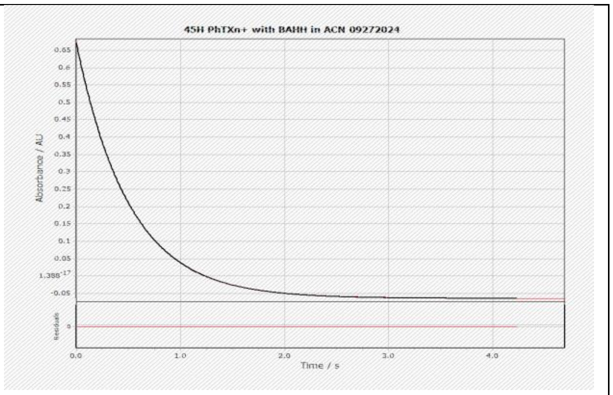

45°C H

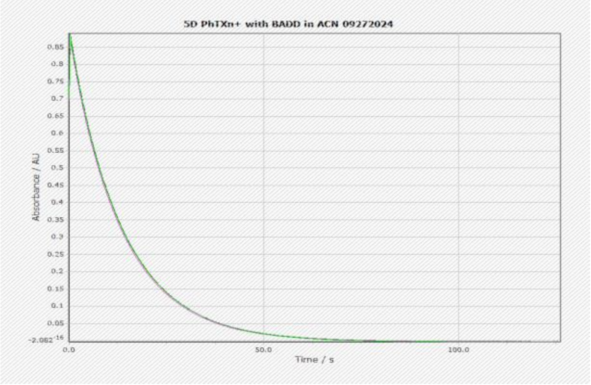

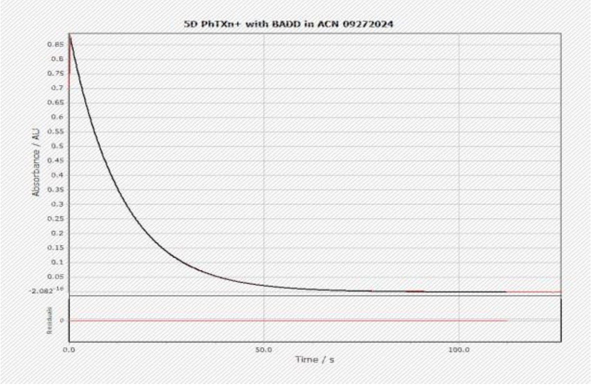

5°C D

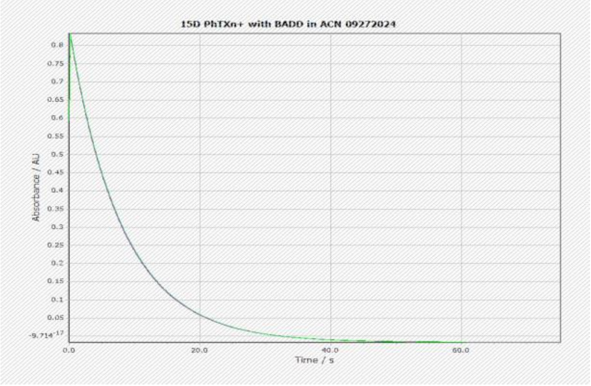

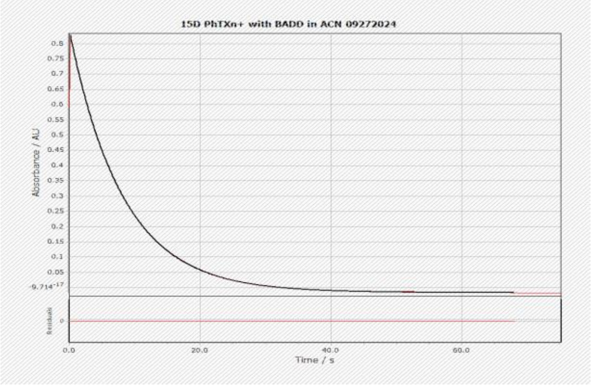

15°C D

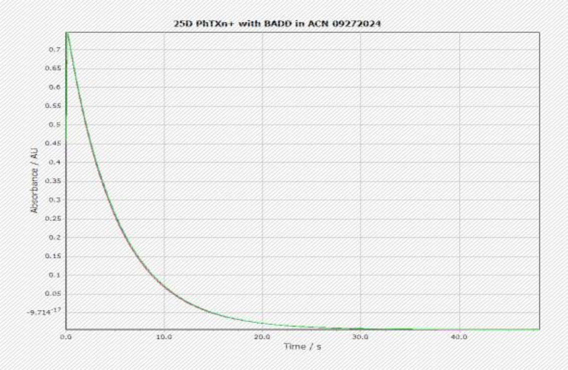

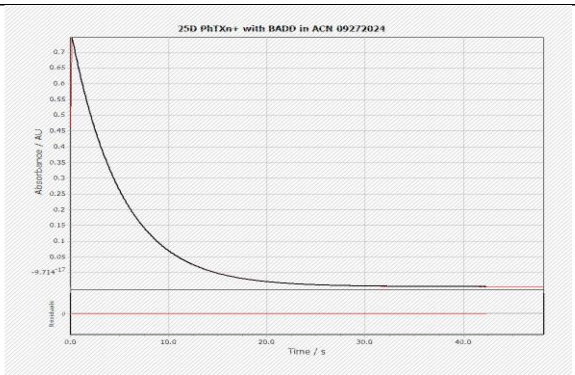

25°C D

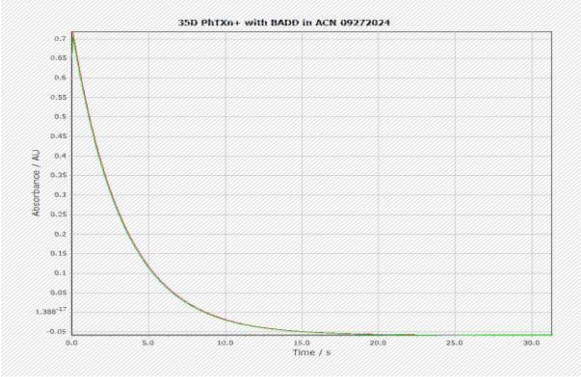

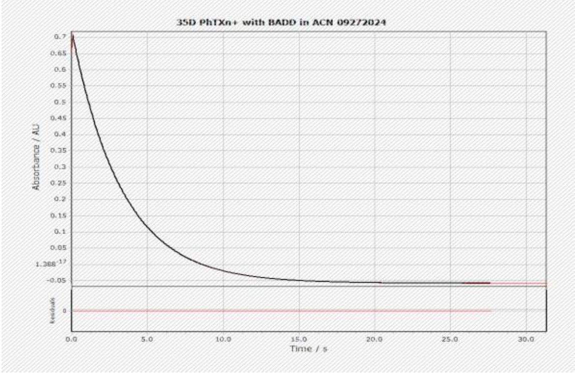

35°C D

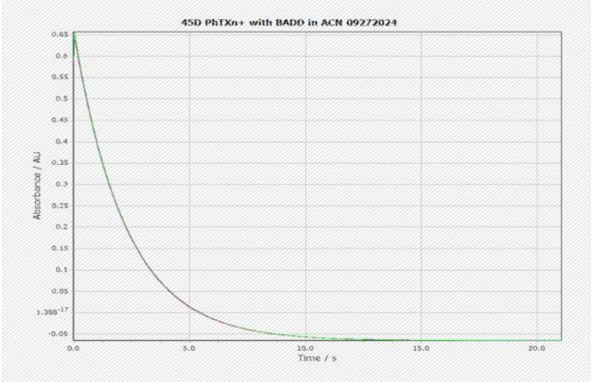

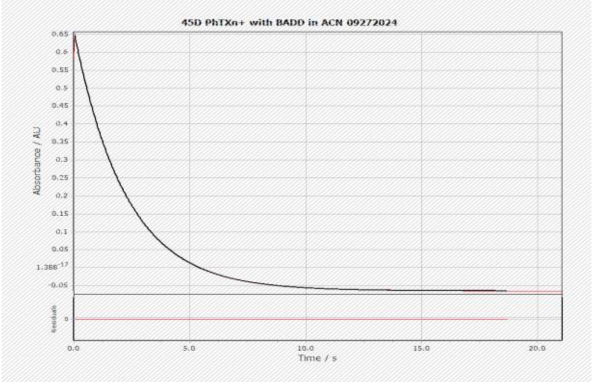

45°C D

Primary kinetic data for the rate constants in Table S5

Day 1 data (December 2, 2023)

	Pseudo-first-order rate constants k^{pfo} (s^{-1})									
**Temp (°C)**	**Trial H1**	**Trial H2**	**Trial H3**	**Trial H4**	**Trial H5**	**Trial H6**	**Average k_H^{pfo} (s^{-1})**	**Stdev**	**k_{2H} ($M^{-1}s^{-1}$)**	**Stdev [a]**
45	40.5261	41.4615	40.9346	41.1674	41.5123	41.4637	41.1776	0.3894	8.45E+04	7.98E+02
35	35.0338	34.6865	34.9050	35.2660	34.7257	35.0527	34.9450	0.2186	7.17E+04	4.48E+02
25	29.2573	29.0916	29.3154	29.1293	29.1783	29.2768	29.2081	0.0887	5.99E+04	1.82E+02
15	24.3875	24.3874	24.6236	24.3481	24.5424	24.4860	24.4613	0.1080	5.02E+04	2.22E+02
5	20.0471	20.0212	19.8377	20.2229	20.0563	19.8654	20.00084	0.1411	4.10E+04	2.89E+02

Temp (°C)	Trial D1	Trial D2	Trial D3	Trial D4	Trial D5	Trial D6	Average k_D^{pfo} (s^{-1})	Stdev	k_{2D} ($M^{-1}s^{-1}$)	Stdev [a]
45	13.8717	13.6287	13.9437	13.8794	13.7202	13.8907	12.6826	0.1209	2.60E+04	10.1664
35	11.3556	11.3266	11.2680	11.3126	11.2665	11.1871	10.3003	0.0595	2.11E+04	9.9584
25	9.1905	9.1660	9.2309	9.2309	9.2309	9.3143	8.4042	0.0559	1.72E+04	9.7550
15	7.2389	7.2637	7.1690	7.1953	7.2126	7.1673	6.4889	0.0385	1.33E+04	9.4963
5	5.5104	5.6766	5.7006	5.6989	5.6390	5.7077	5.075	0.0754	1.04E+04	9.1471

[a] = (Stdev(for k^{pfo})/k^{pfo})*k_{2H}

Six kinetic runs together with the averaged data	First-order kinetic fit of the averaged data as an example
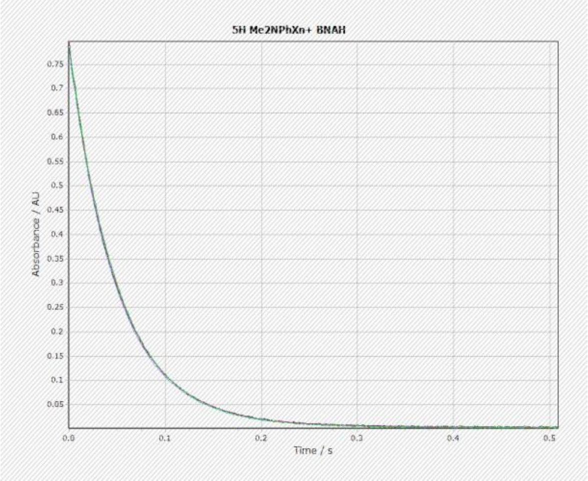	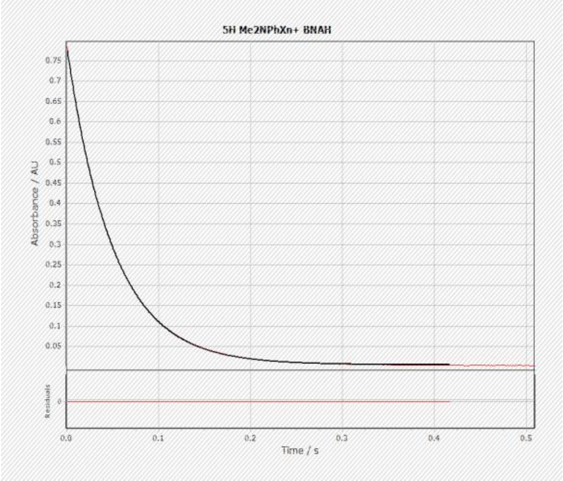

5℃ H

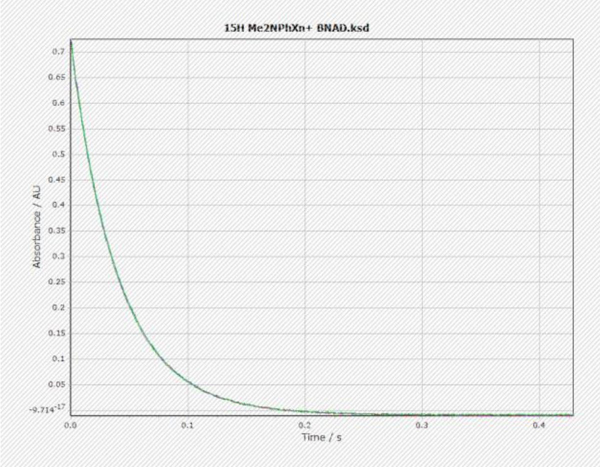	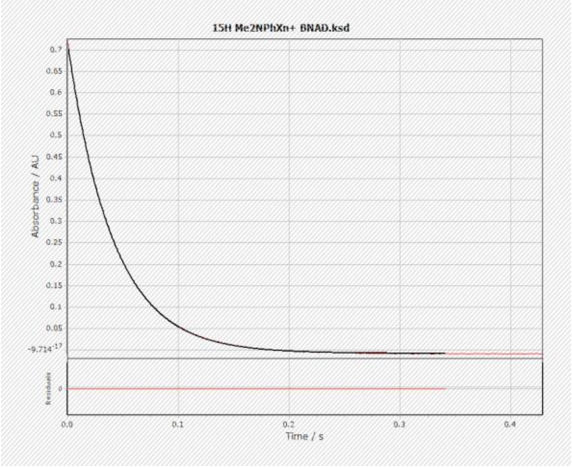

15℃ H

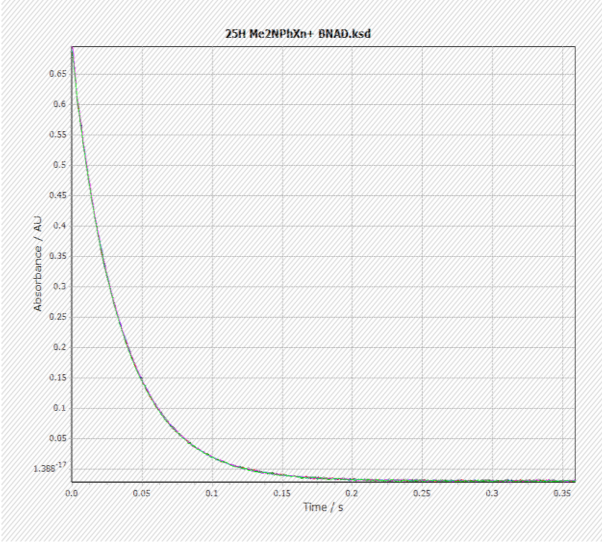
25H Me2NPhXn+ BNAD.ksd
Absorbance / AU
Time / s

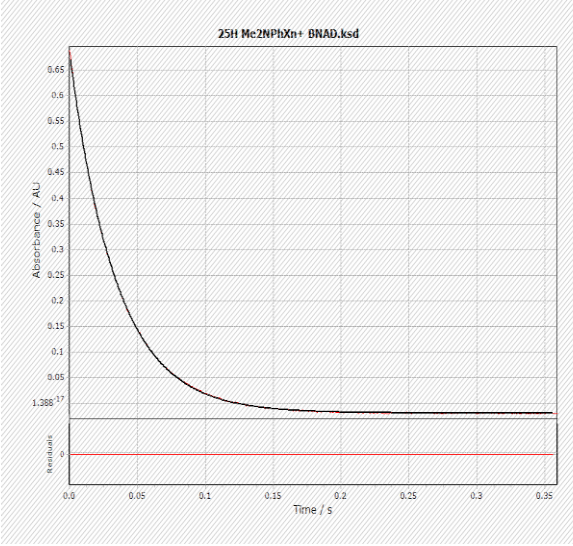
25H Me2NPhXn+ BNAD.ksd
Absorbance / AU
Residuals
Time / s

25℃ H

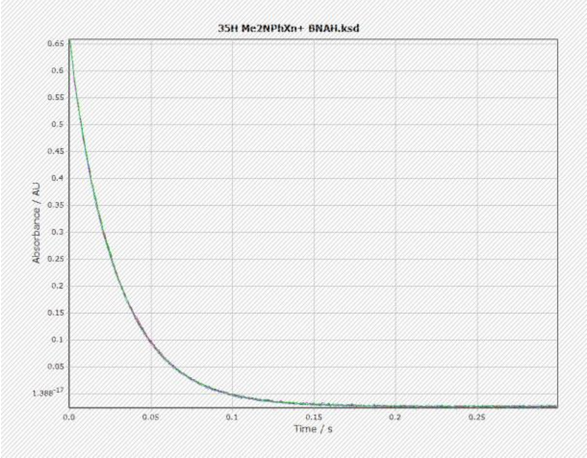
35H Me2NPhXn+ BNAH.ksd
Absorbance / AU
Time / s

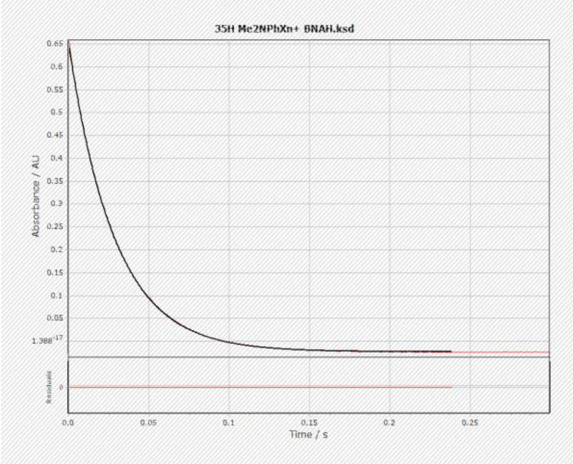
35H Me2NPhXn+ BNAH.ksd
Absorbance / AU
Residuals
Time / s

35℃ H

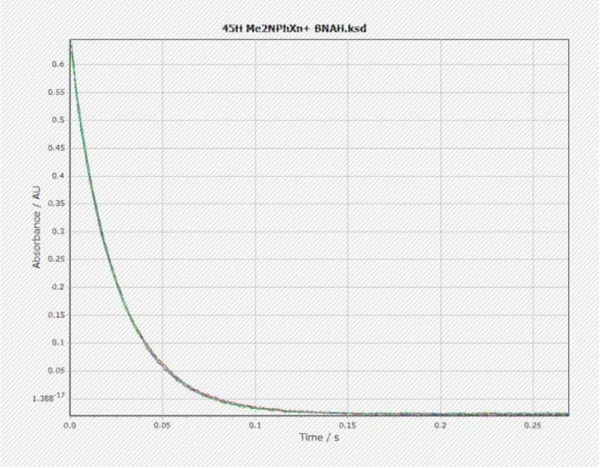
45H Me2NPhXn+ BNAH.ksd
Absorbance / AU
Time / s

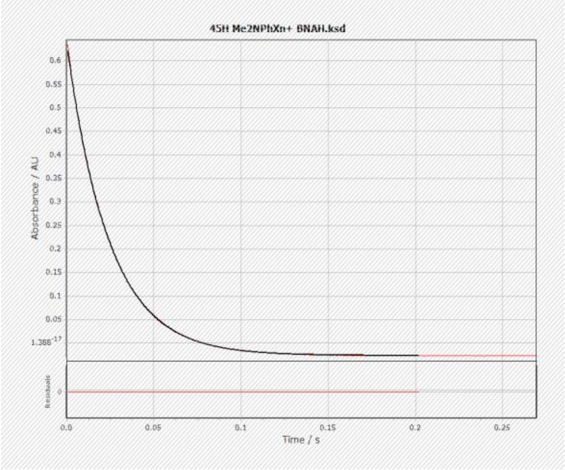
45H Me2NPhXn+ BNAH.ksd
Absorbance / AU
Residuals
Time / s

45℃ H

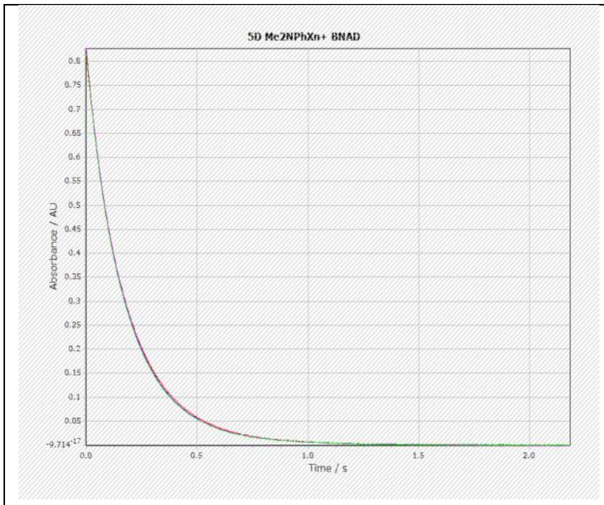

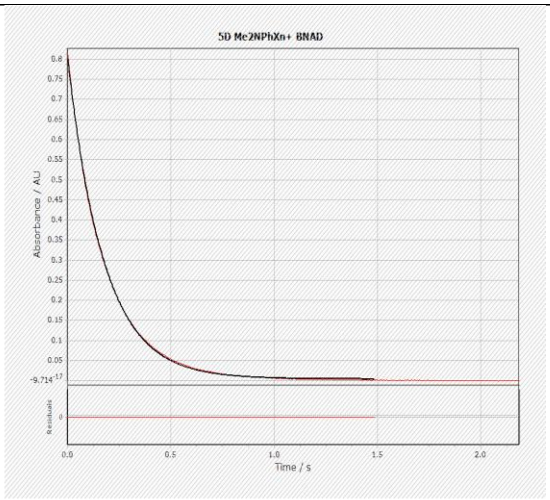

5°C D

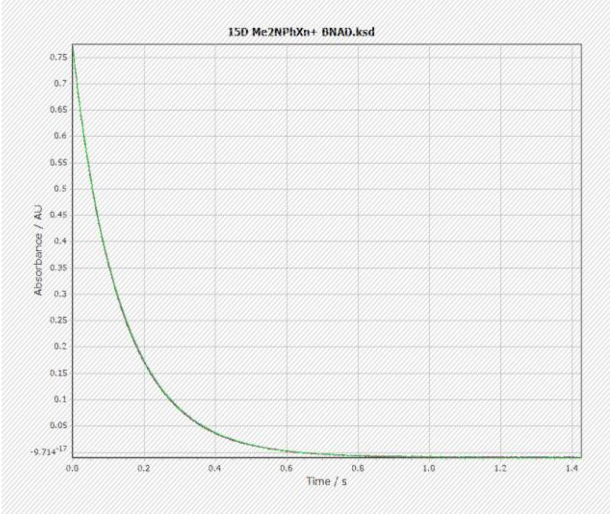

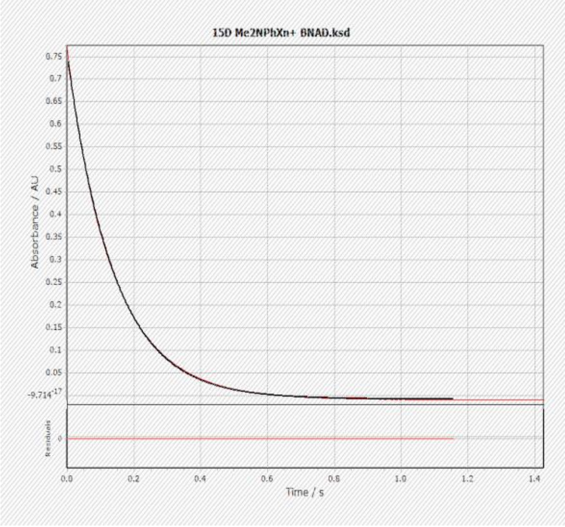

15°C D

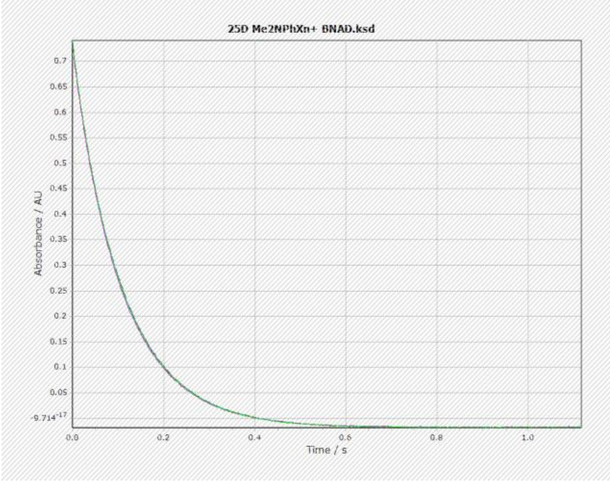

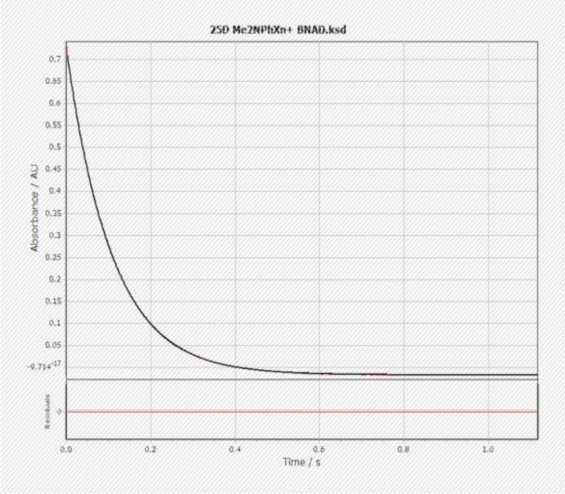

25°C D

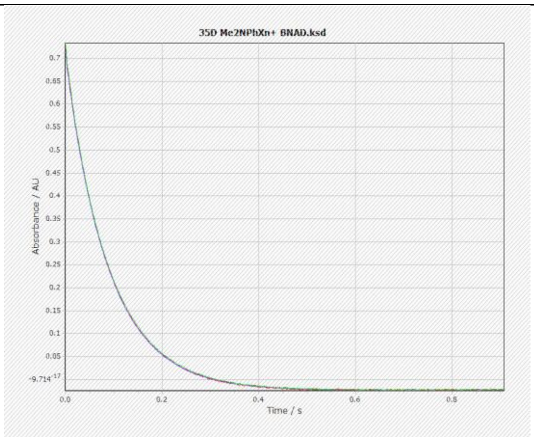

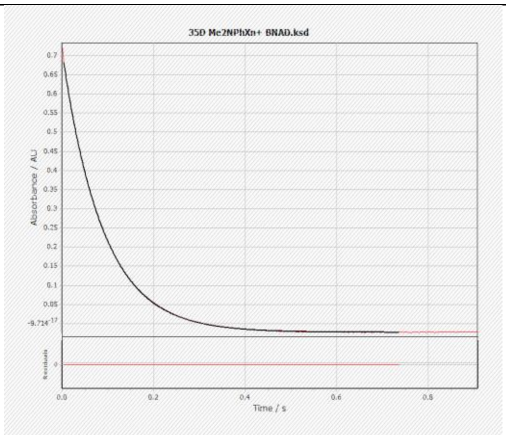

35°C D

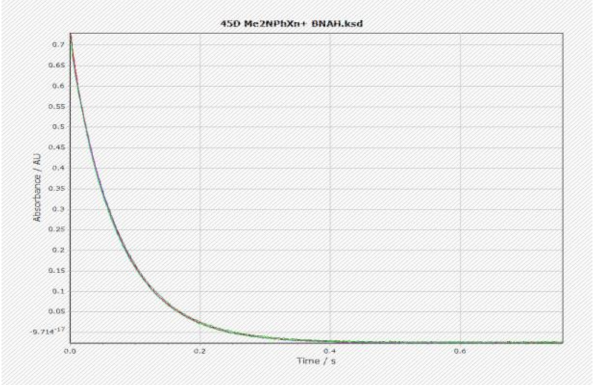

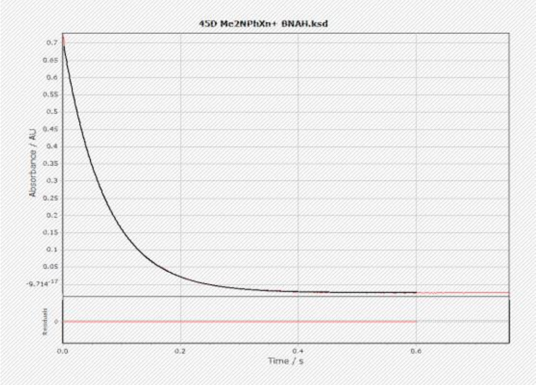

45°C D

Day 2 data (December 14, 2023)

Pseudo-first-order rate constants

Temp (°C)	k^{pfo} (s^{-1}) Trial H1	Trial H2	Trial H3	Trial H4	Trial H5	Trial H6	Average k_H^{pfo} (s^{-1})	Stdev	k_{2H} ($M^{-1}s^{-1}$)	Stdev [a]
45	36.7959	37.4474	37.2086	36.9088	37.3612	37.7517	37.2456	0.3542	7.64E+04	7.27E+02
35	31.1909	31.3132	31.8404	30.9735	31.5845	31.7835	31.4477	0.3448	6.45E+04	7.07E+02
25	26.2928	26.7320	26.2588	26.6498	26.1589	26.3694	26.4103	0.2291	5.42E+04	4.70E+02
15	22.0651	22.1647	21.8376	21.9206	22.0521	22.2289	22.0448	0.1464	4.52E+04	3.00E+02
5	18.0481	17.9545	17.9989	17.9632	18.0552	18.0559	18.0126	0.0468	3.69E+04	0.96E+02
**Temp (°C)**	**Trial D1**	**Trial D2**	**Trial D3**	**Trial D4**	**Trial D5**	**Trial D6**	**Average k_D^{pfo} (s^{-1})**	**Stdev**	**k_{2D} ($M^{-1}s^{-1}$)**	**Stdev [a]**
45	14.2998	14.1179	14.1015	14.2564	14.3549	14.4412	13.3043	0.1332	2.73E+04	2.73E+02
35	11.6450	11.8158	11.6943	11.6139	11.5931	11.6792	10.8496	0.0794	2.23E+04	1.63E+02
25	9.4018	9.4198	9.4903	9.5010	9.4113	9.4547	8.7397	0.0422	1.79E+04	0.87E+02
15	7.4674	7.5496	7.4657	7.5157	7.4927	7.5154	6.8951	0.0323	1.41E+04	0.66E+02
5	5.8186	5.9192	5.9024	5.8897	5.8915	5.8700	5.3765	0.0350	1.10E+04	0.72E+02

[a] = (Stdev(for k^{pfo})/k^{pfo})*k_{2H}

<table>
<tr><td>Six kinetic runs together with the averaged data</td><td>First-order kinetic fit of the averaged data as an example</td></tr>
<tr><td>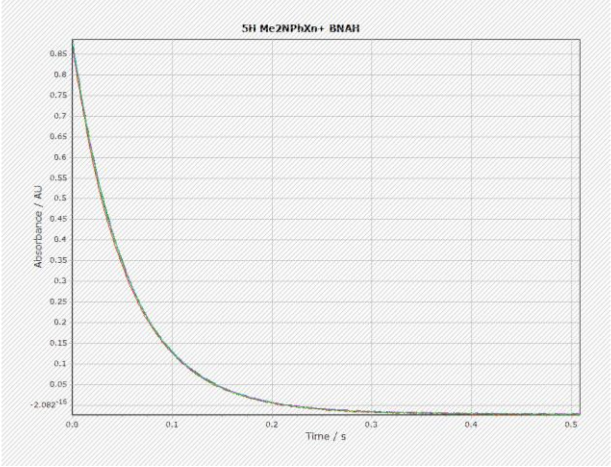
</td><td>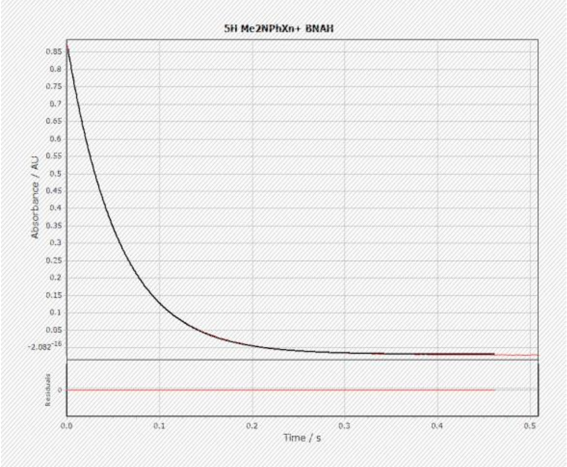
</td></tr>
<tr><td colspan="2">5℃ H</td></tr>
<tr><td>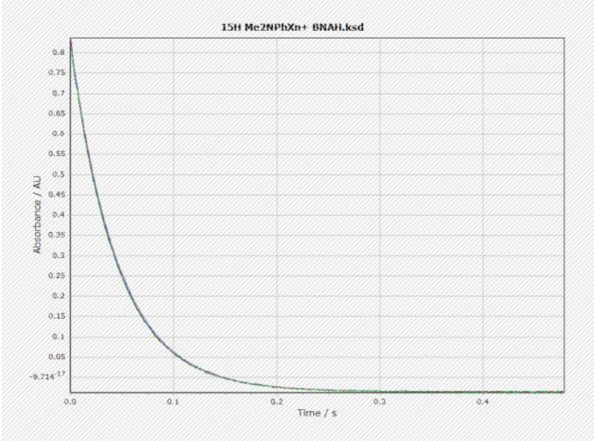
</td><td>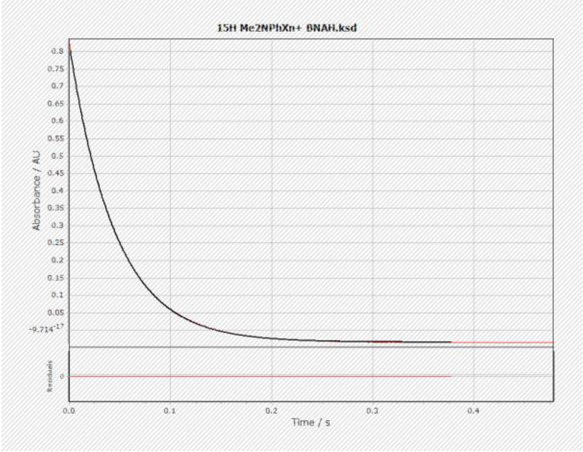
</td></tr>
<tr><td colspan="2">15℃ H</td></tr>
<tr><td>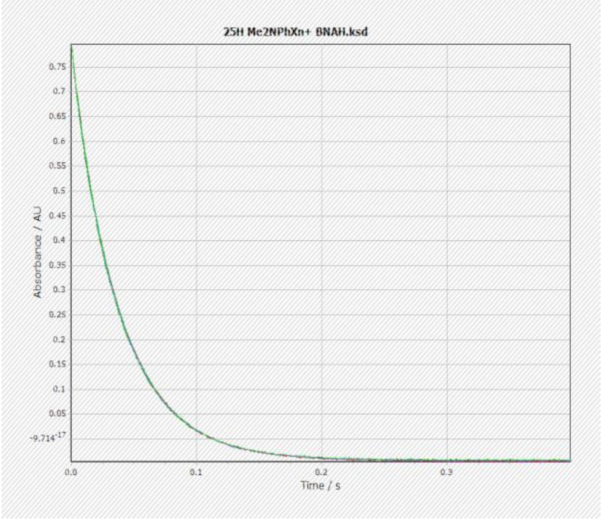
</td><td>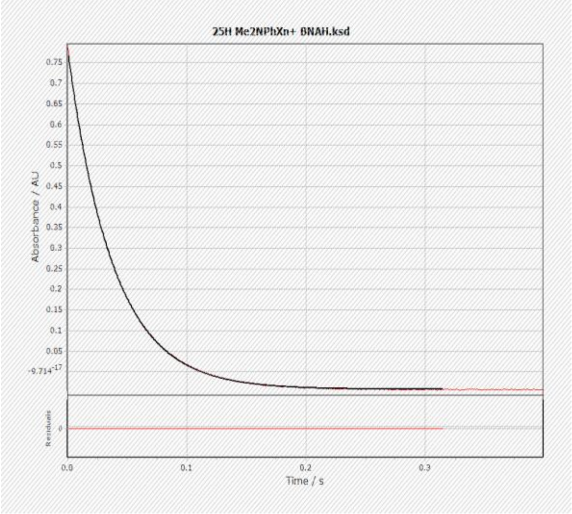
</td></tr>
<tr><td colspan="2">25℃ H</td></tr>
</table>

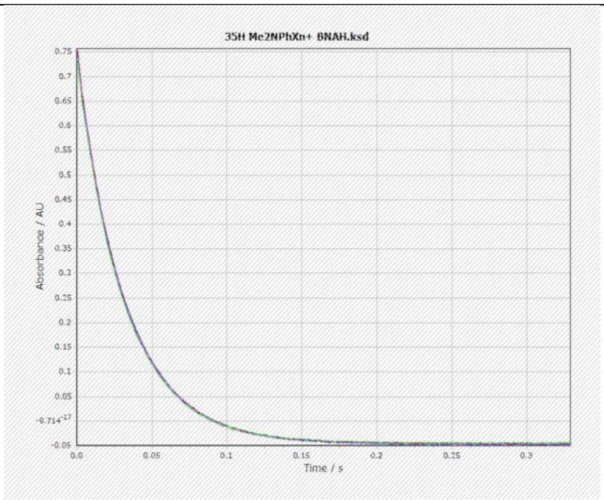
35H Me2NPhXn+ BNAH.ksd
Absorbance / AU
Time / s

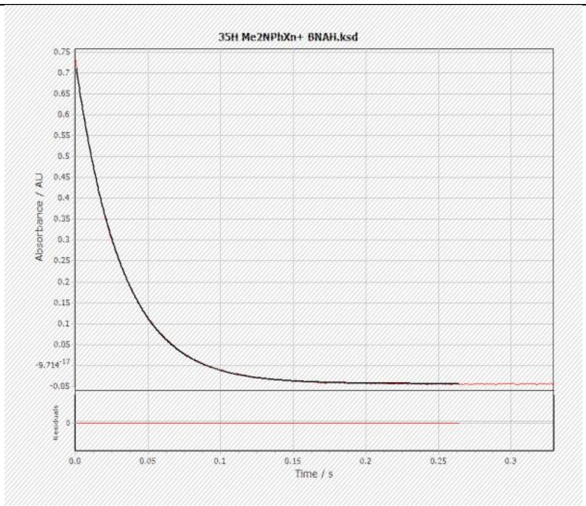
35H Me2NPhXn+ BNAH.ksd
Absorbance / AU
Residuals
Time / s

35℃ H

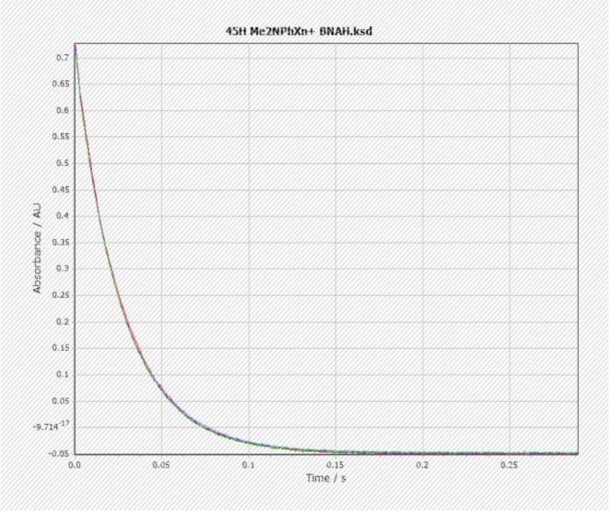
45H Me2NPhXn+ BNAH.ksd
Absorbance / AU
Time / s

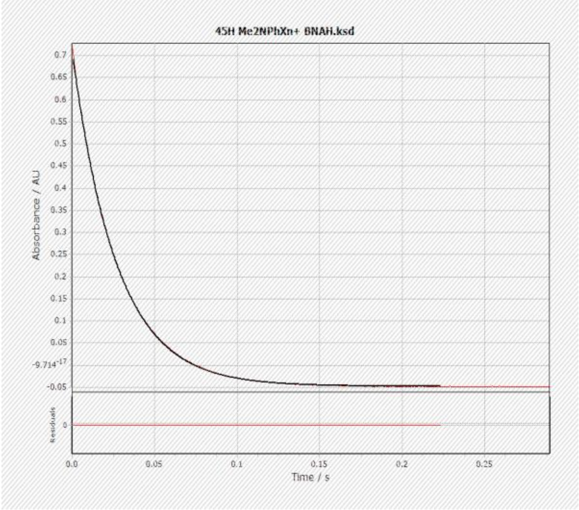
45H Me2NPhXn+ BNAH.ksd
Absorbance / AU
Residuals
Time / s

45℃ H

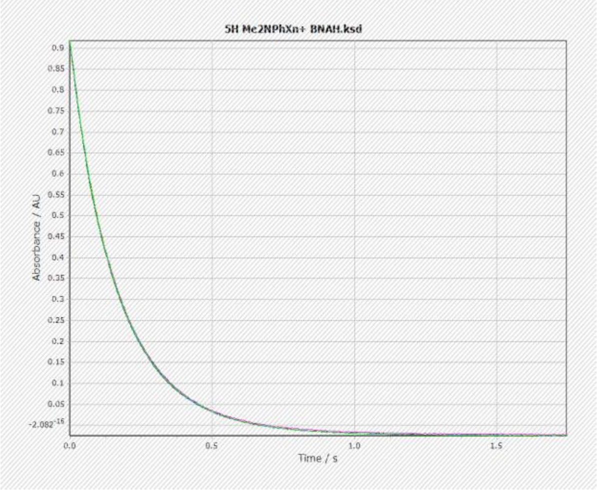
5H Me2NPhXn+ BNAH.ksd
Absorbance / AU
Time / s

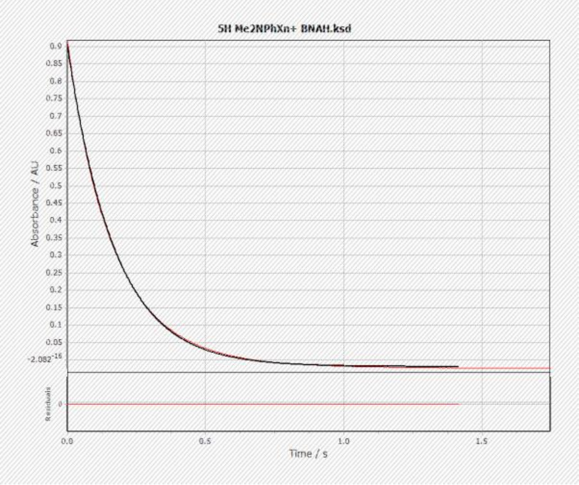
5H Me2NPhXn+ BNAH.ksd
Absorbance / AU
Residuals
Time / s

5℃ D

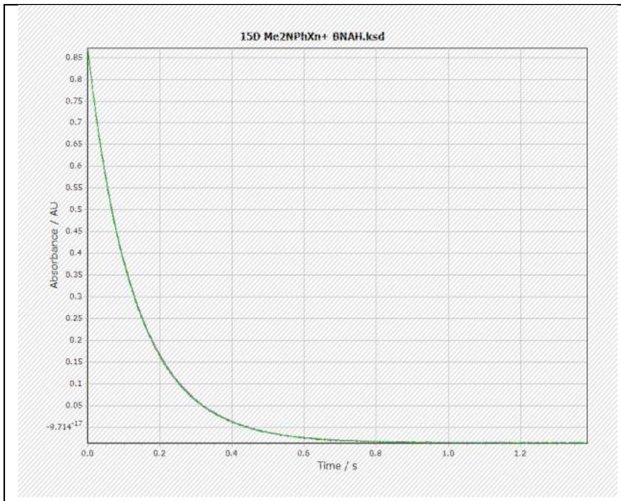
15D Me2NPhXn+ BNAH.ksd
Absorbance / AU
Time / s

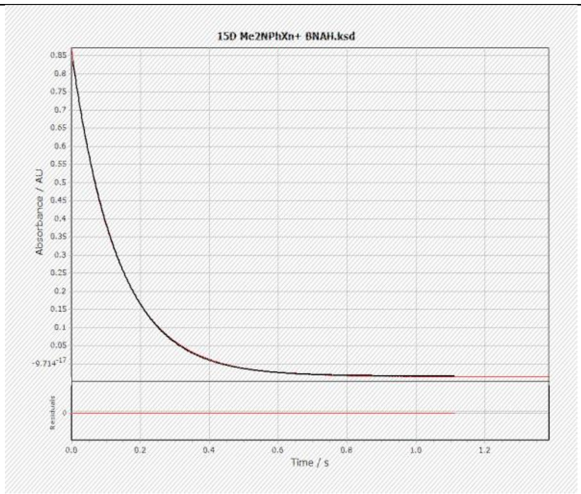
15D Me2NPhXn+ BNAH.ksd
Absorbance / AU
Residuals
Time / s

15℃ D

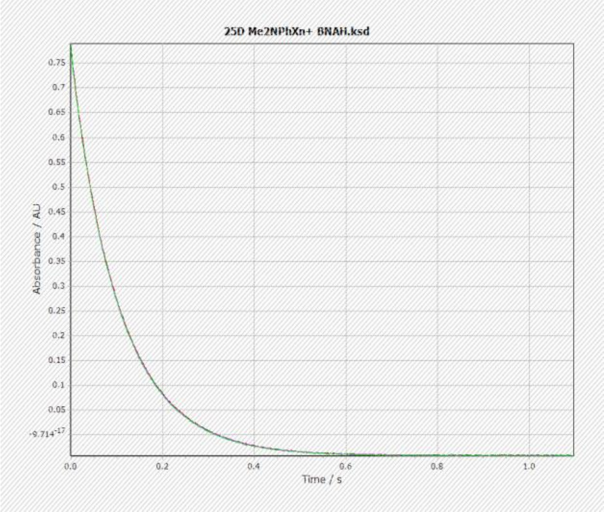
25D Me2NPhXn+ BNAH.ksd
Absorbance / AU
Time / s

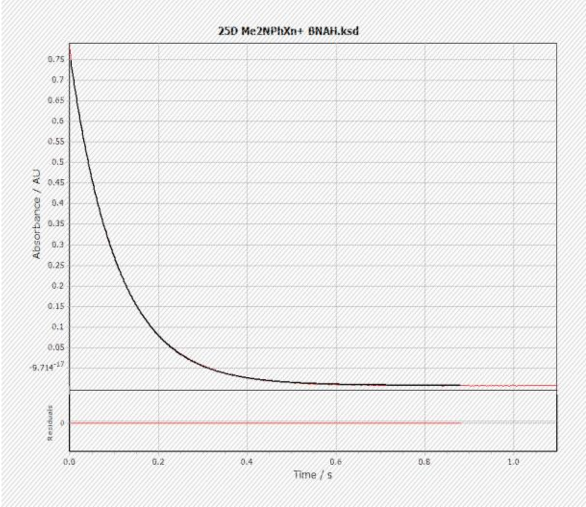
25D Me2NPhXn+ BNAH.ksd
Absorbance / AU
Residuals
Time / s

25℃ D

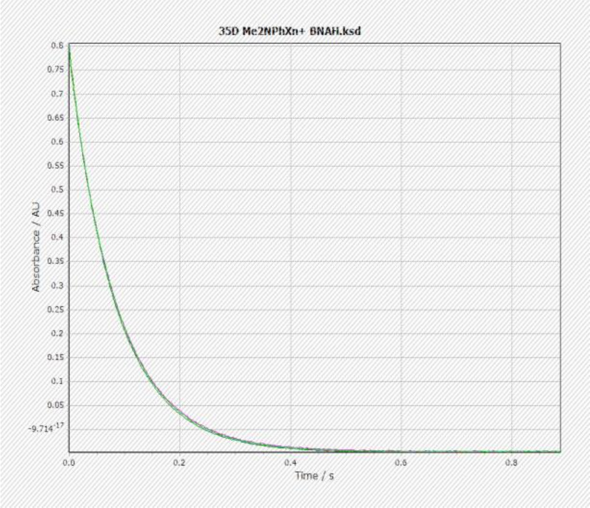
35D Me2NPhXn+ BNAH.ksd
Absorbance / AU
Time / s

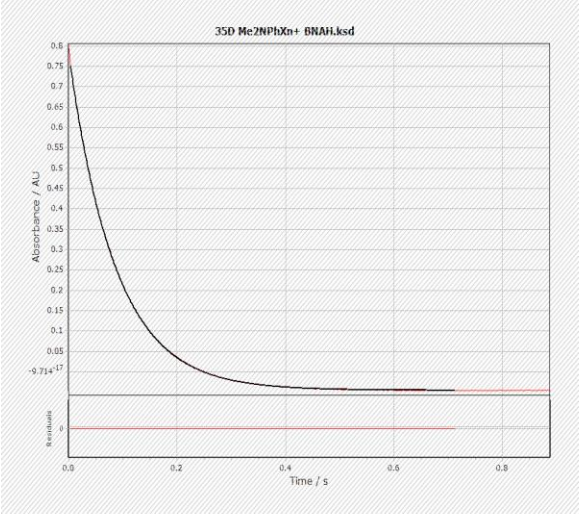
35D Me2NPhXn+ BNAH.ksd
Absorbance / AU
Residuals
Time / s

35℃ D

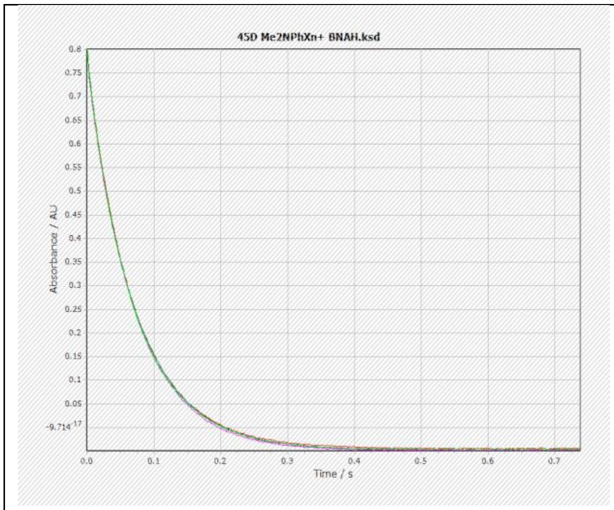

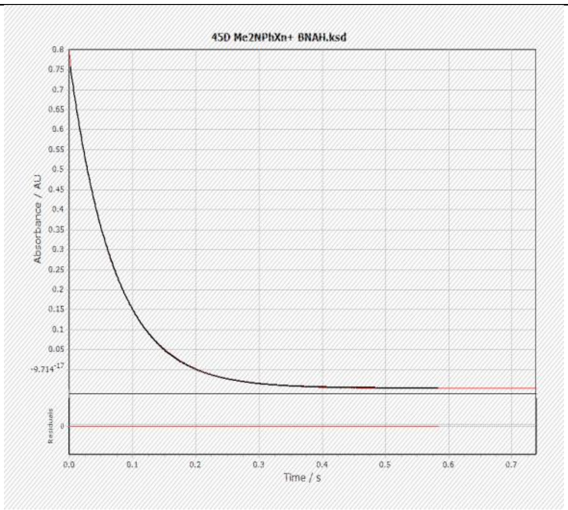

45℃ D

Day 3 data (December 18, 2023)

Temp (°C)	Pseudo-first-order rate constants k^{pfo} (s^{-1}) Trial H1	Trial H2	Trial H3	Trial H4	Trial H5	Trial H6	Average k_H^{pfo} (s^{-1})	Stdev	k_{2H} ($M^{-1}s^{-1}$)	Stdev [a]
45	37.7204	37.3625	37.8144	37.3661	37.9852	37.3526	37.6002	0.2761	7.71E+04	5.66E+02
35	31.5231	31.7739	32.5830	31.9520	32.0965	31.9960	31.9874	0.3547	6.56E+04	7.27E+02
25	26.7189	26.5350	26.5384	26.6582	26.3480	26.9288	26.6212	0.1972	5.46E+04	4.04E+02
15	21.9265	22.0798	22.1297	22.0306	22.1130	21.9613	22.0461	0.0735	4.52E+04	1.51E+02
5	18.0303	18.2118	18.0460	17.9502	18.2311	18.2326	18.1170	0.1231	3.72E+04	2.52E+02
**Temp (°C)**	**Trial D1**	**Trial D2**	**Trial D3**	**Trial D4**	**Trial D5**	**Trial D6**	**Average k_D^{pfo} (s^{-1})**	**Stdev**	**k_{2D} ($M^{-1}s^{-1}$)**	**Stdev [a]**
45	14.2596	14.1892	14.4030	14.2508	14.4471	14.2610	13.3310	0.1001	2.73E+04	2.05E+02
35	11.7021	11.6392	11.6425	11.6207	11.6864	11.6981	10.8181	0.0348	2.22E+04	0.71E+02
25	9.3159	9.3826	9.5525	9.3573	9.3999	9.3532	8.6758	0.0829	1.79E+04	1.70E+02
15	7.4765	7.4115	7.4617	7.4938	7.4586	7.4582	6.8523	0.0275	1.41E+04	0.56E+02
5	5.8677	5.9293	5.9184	5.8713	5.7974	5.8676	5.3643	0.0456	1.10E+04	0.94E+02

[a] = (Stdev(for k^{pfo})/k^{pfo})*k_{2H}

Six kinetic runs together with the averaged data	First-order kinetic fit of the averaged data as an example
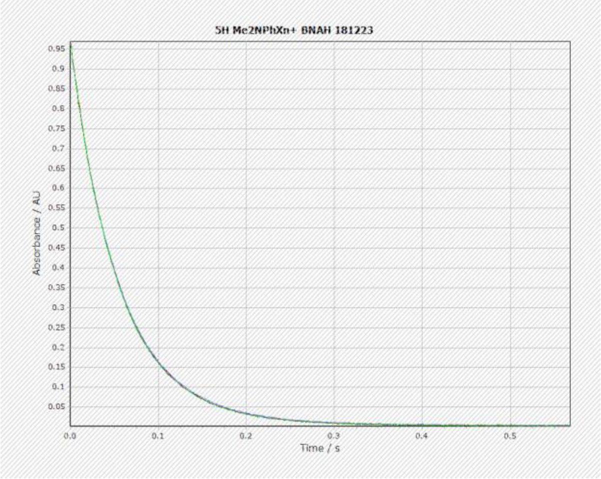	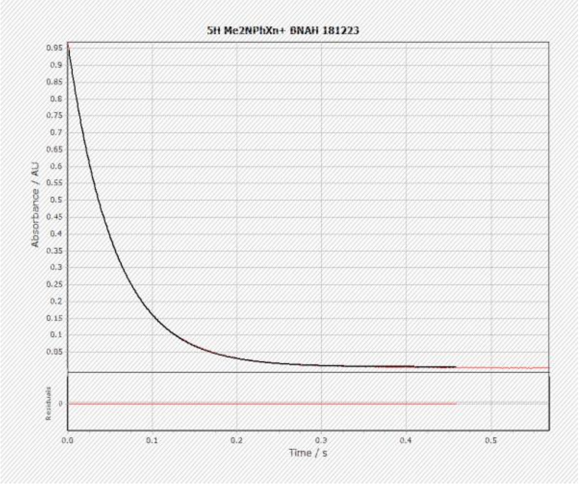
5℃ H	
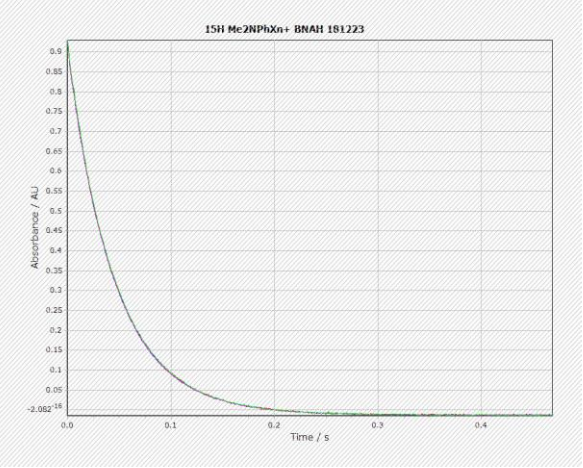	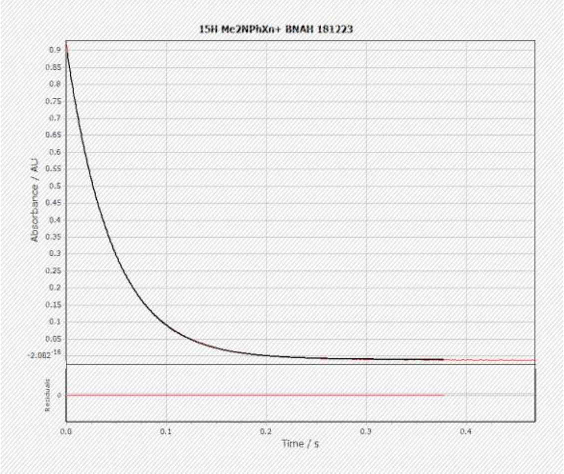
15℃ H	
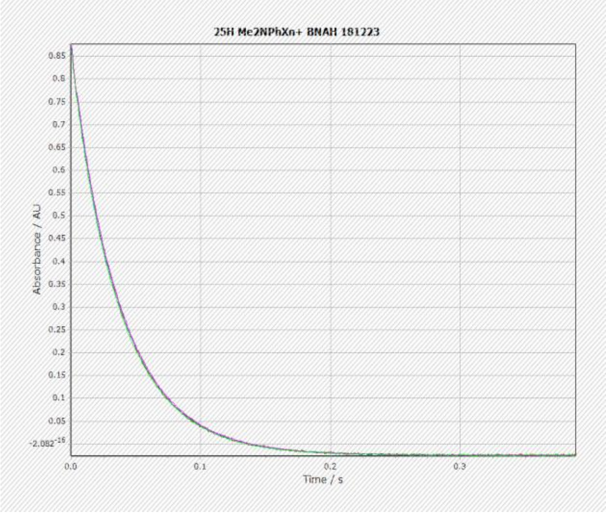	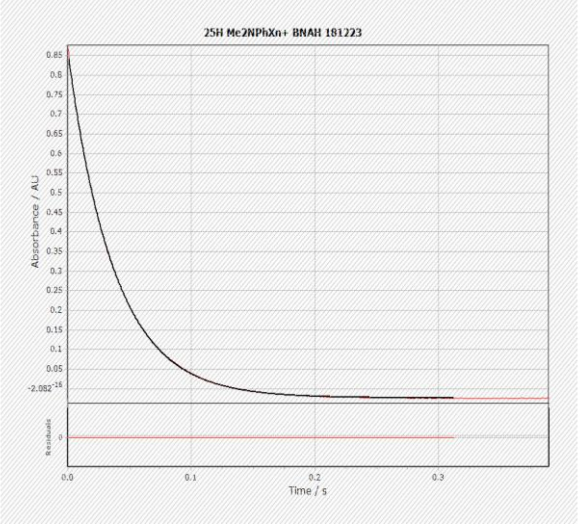
25℃ H	

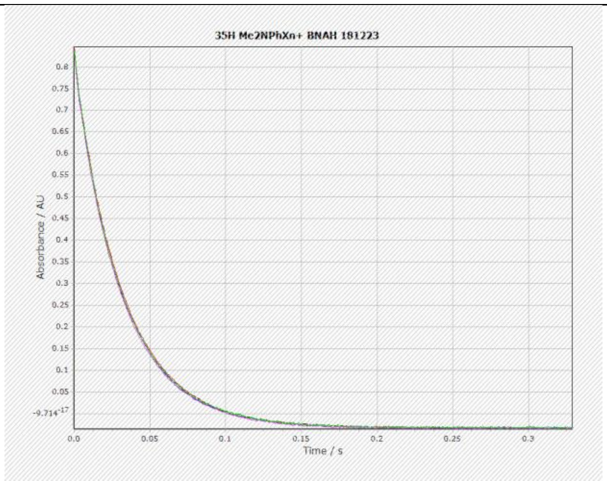

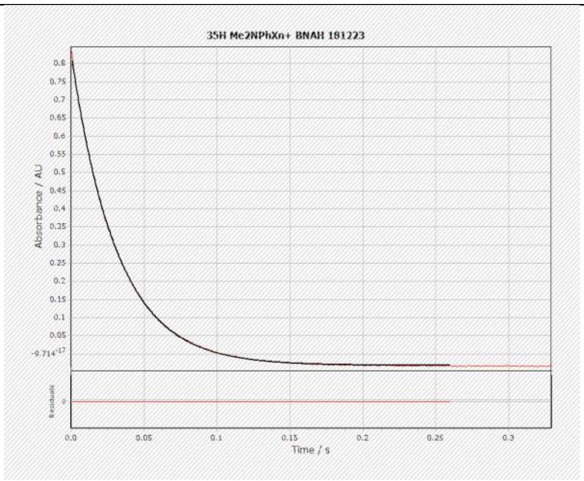

35°C H

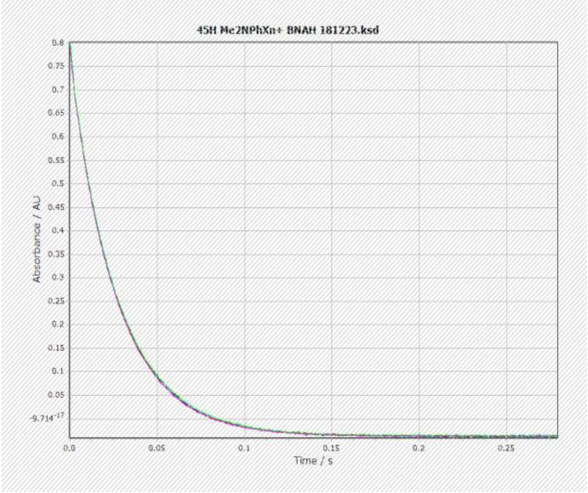

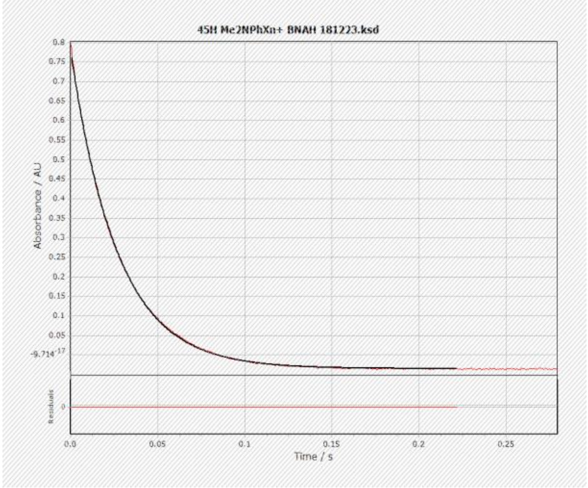

45°C H

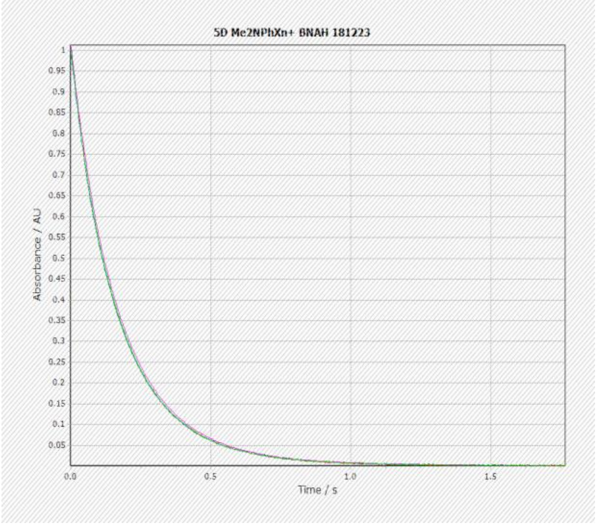

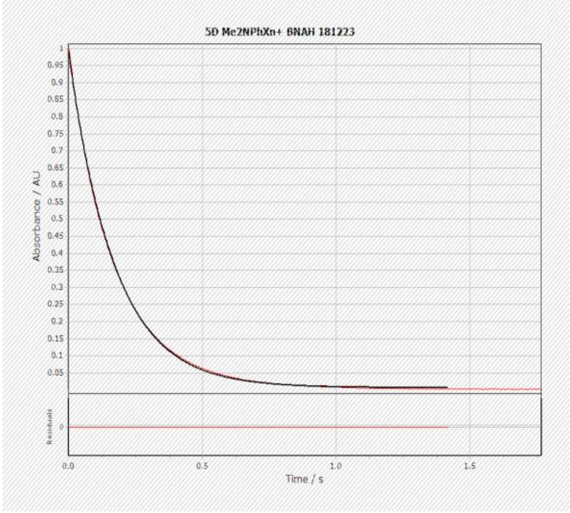

5°C D

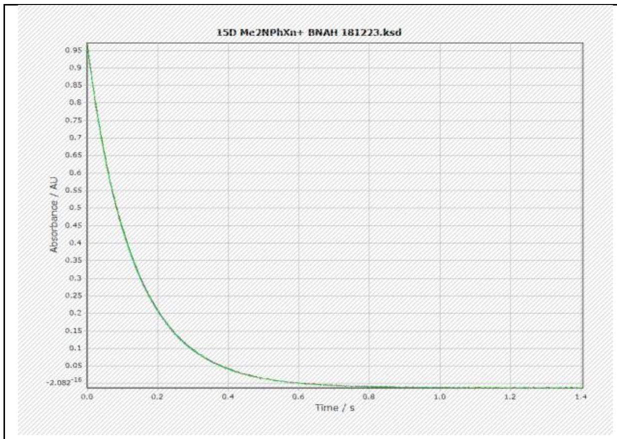
15D Me2NPhXn+ BNAH 181223.ksd
Absorbance / AU
Time / s

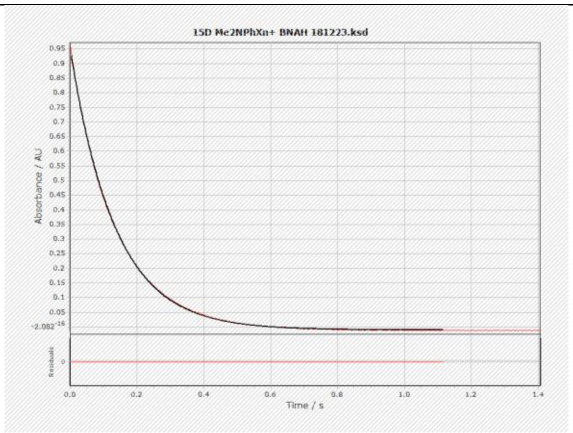
15D Me2NPhXn+ BNAH 181223.ksd
Absorbance / AU
Residuals
Time / s

15℃ D

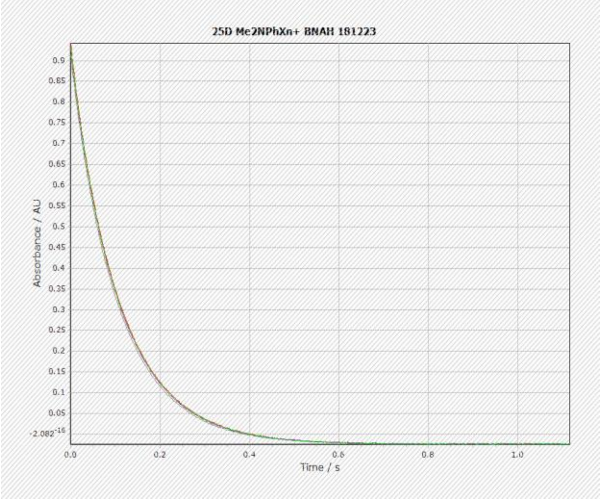
25D Me2NPhXn+ BNAH 181223
Absorbance / AU
Time / s

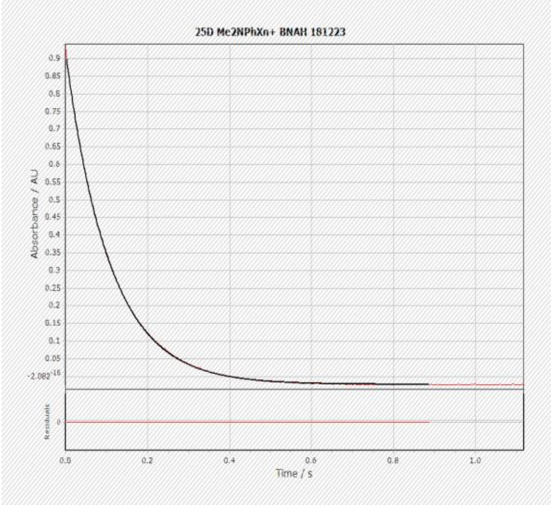
25D Me2NPhXn+ BNAH 181223
Absorbance / AU
Residuals
Time / s

25℃ D

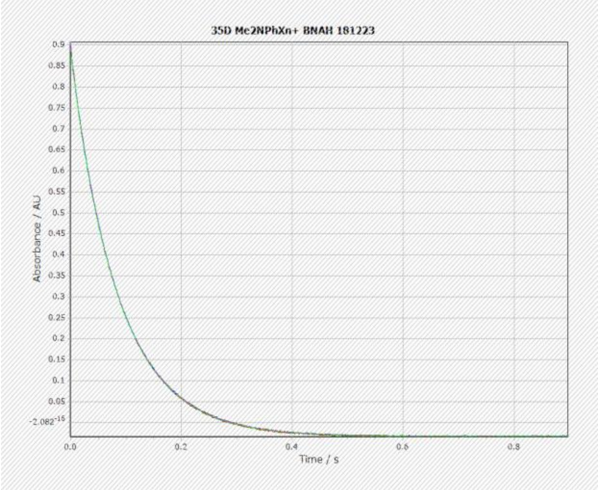
35D Me2NPhXn+ BNAH 181223
Absorbance / AU
Time / s

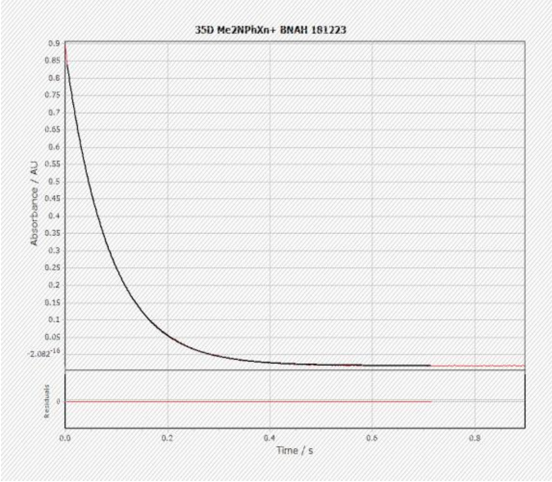
35D Me2NPhXn+ BNAH 181223
Absorbance / AU
Residuals
Time / s

35℃ D

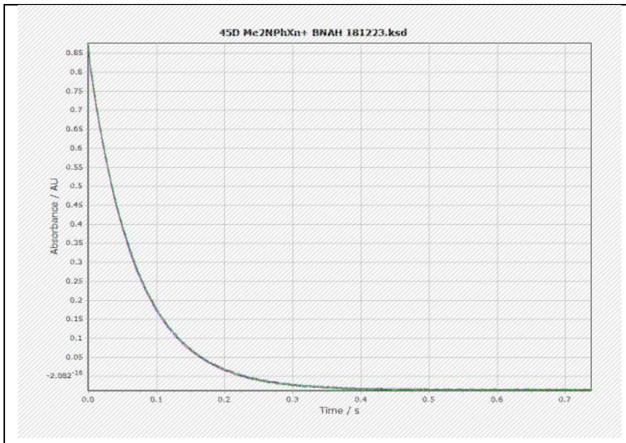

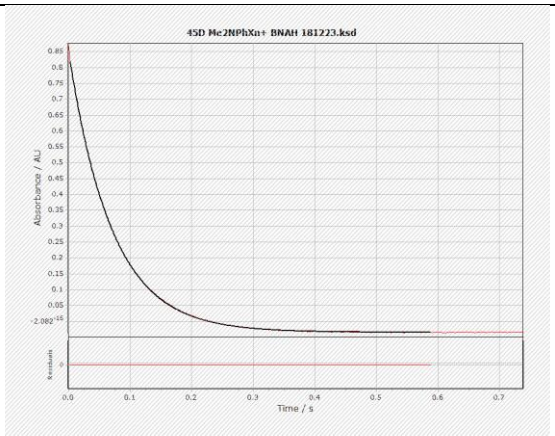

45℃ D

Primary kinetic data for the rate constants in Table S6

Day 1 data (January 26, 2024)

Pseudo-first-order rate constants

k^{pfo} (s^{-1})

Temp (°C)	Trial H1	Trial H2	Trial H3	Average k_H^{pfo} (s^{-1})	Stdev	k_{2H} ($M^{-1}s^{-1}$)	Stdev [a]
55	0.020263	0.020383	0.020495	0.020380	0.000116	3.40	0.019337
45	0.012974	0.013019	0.013203	0.013065	0.000121	2.18	0.020221
35	0.008371	0.008413	0.008504	0.008429	0.000068	1.40	0.011331
25	0.004957	0.005001	0.005046	0.005001	0.000045	0.83	0.007417
15	0.002922	0.002976	0.002994	0.002964	0.000037	0.49	0.006245

Temp (°C)	Trial D1	Trial D2	Trial D3	Average k_D^{pfo} (s^{-1})	Stdev	k_{2D} ($M^{-1}s^{-1}$)	Stdev [a]
55	0.005643	0.005630	0.005618	0.005016	0.000013	8.36E-01	0.00084
45	0.003506	0.003503	0.003508	0.003107	0.000003	5.18E-01	0.000419
35	0.002119	0.002120	0.002125	0.001859	0.000003	3.10E-01	0.000536
25	0.001224	0.001225	0.001230	0.001069	0.000003	1.78E-01	0.000536
15	0.000669	0.000665	0.000672	0.000573	0.000004	9.55E-02	0.000585

[a] = (Stdev(for k^{pfo})/k^{pfo})*k_{2H}

Three kinetic runs together with the averaged data	First-order kinetic fit of the averaged data as an example
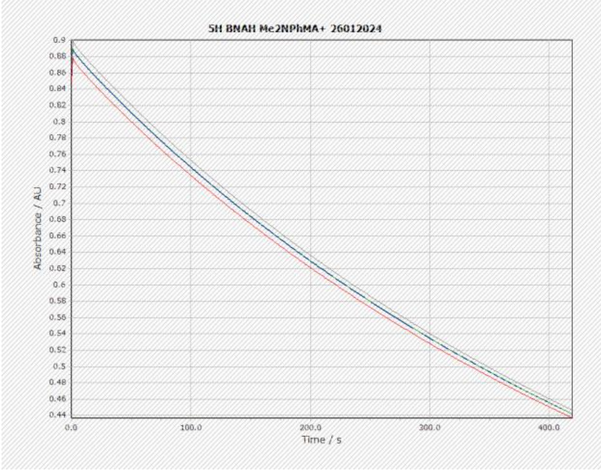	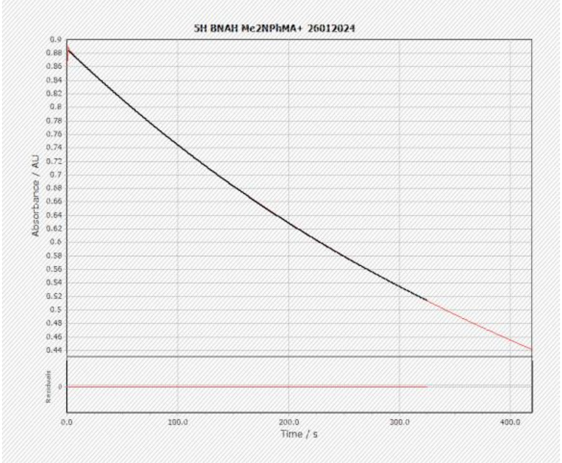
5°C H	
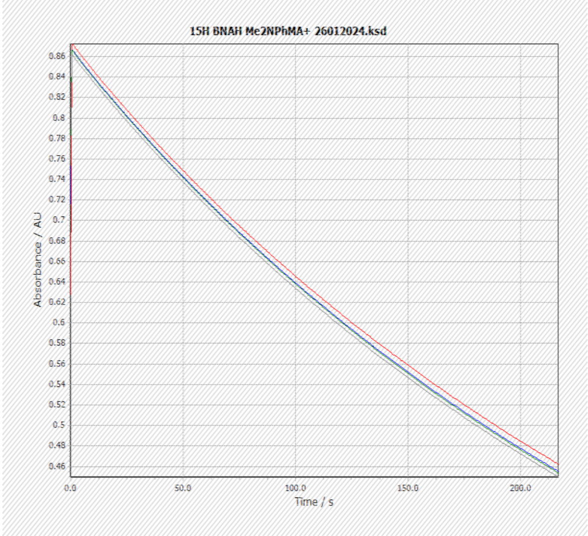	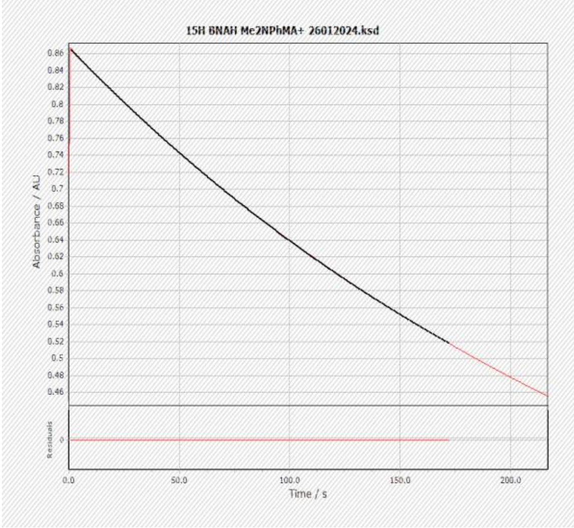
15°C H	
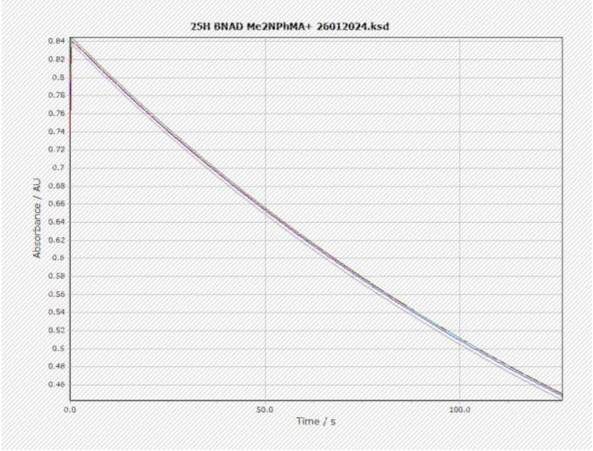	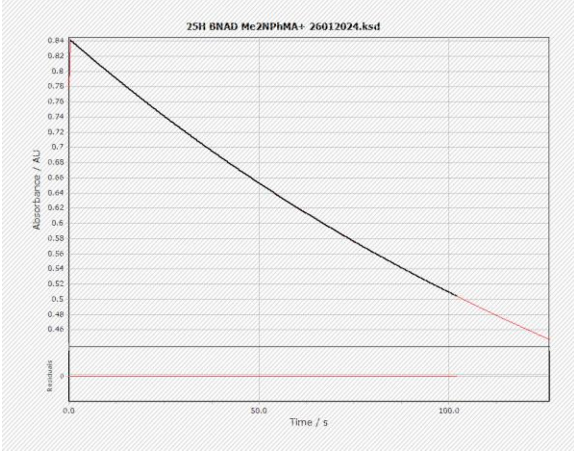
25°C H	

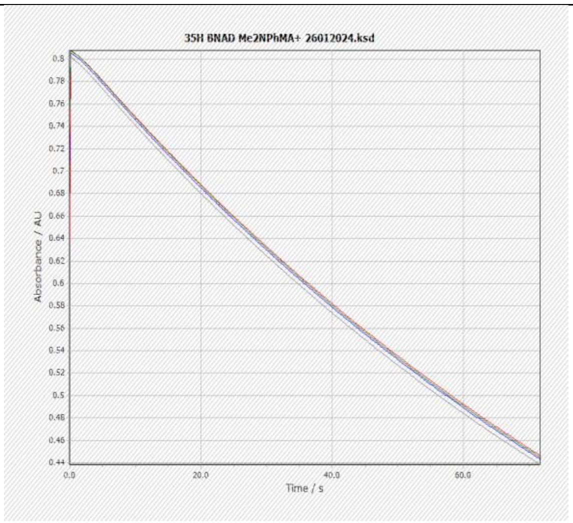
35H BNAD Me2NPhMA+ 26012024.ksd
Absorbance / AU
Time / s
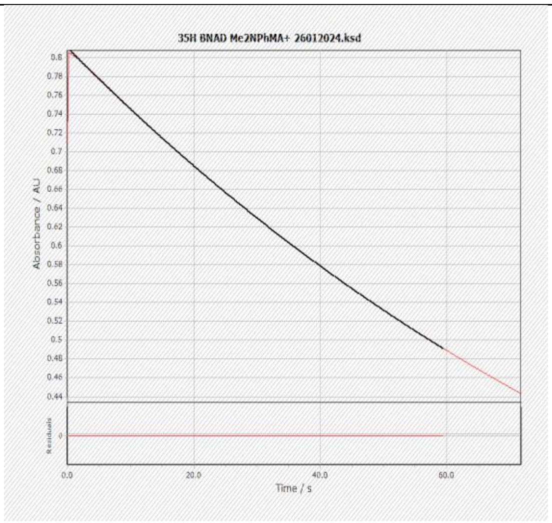
35H BNAD Me2NPhMA+ 26012024.ksd
Absorbance / AU
Residuals
Time / s

35℃ H

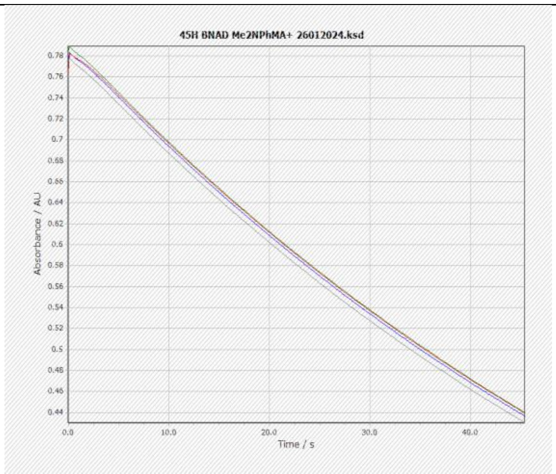
45H BNAD Me2NPhMA+ 26012024.ksd
Absorbance / AU
Time / s
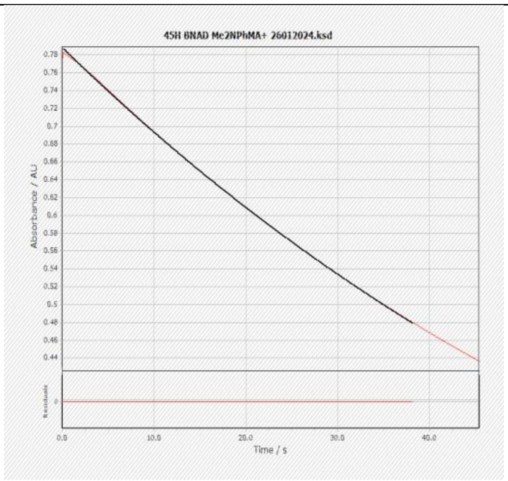
45H BNAD Me2NPhMA+ 26012024.ksd
Absorbance / AU
Residuals
Time / s

45℃ H

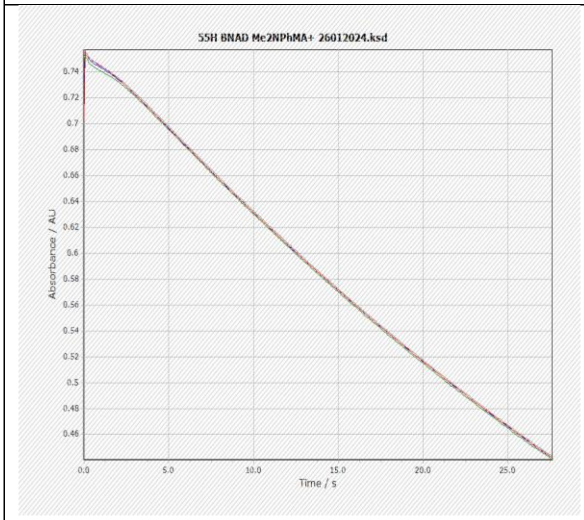
55H BNAD Me2NPhMA+ 26012024.ksd
Absorbance / AU
Time / s
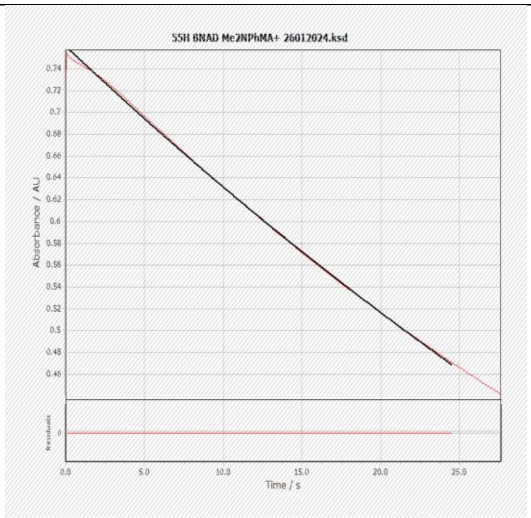
55H BNAD Me2NPhMA+ 26012024.ksd
Absorbance / AU
Residuals
Time / s

55℃ H

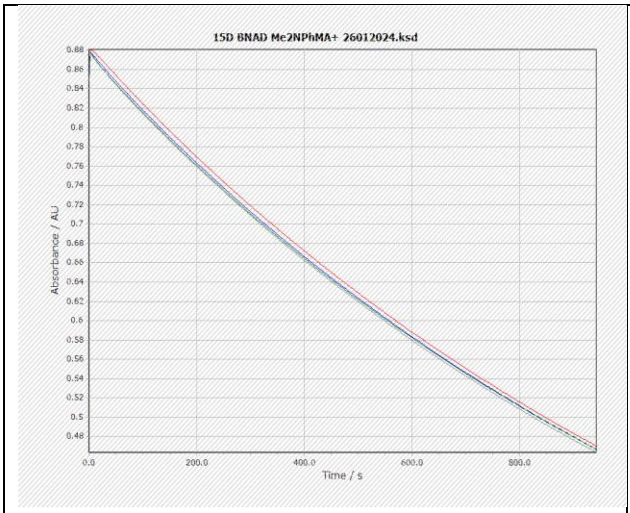

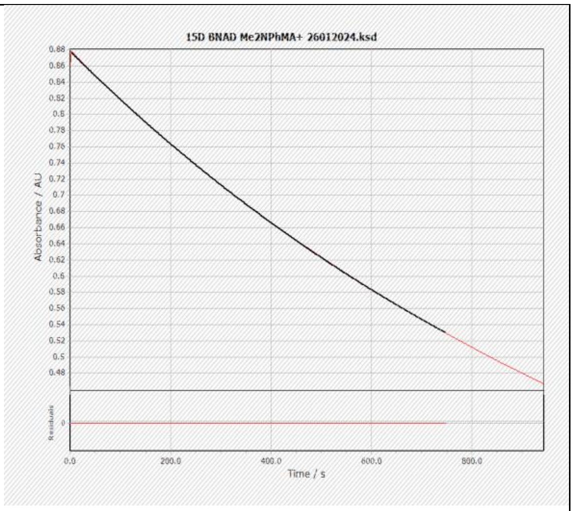

15°C D

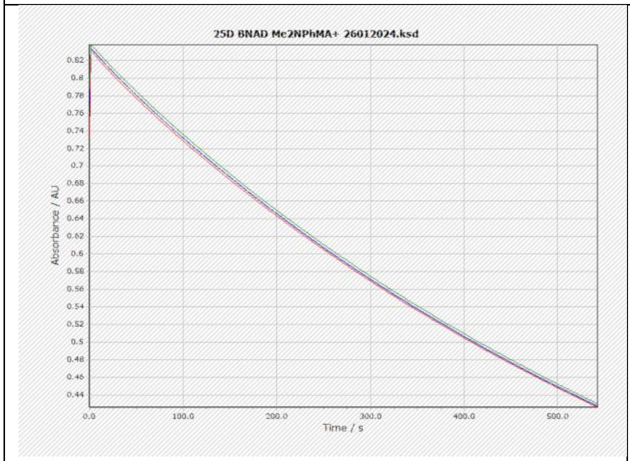

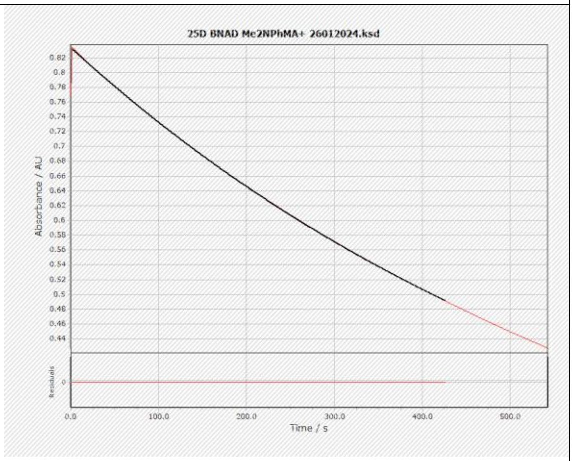

25°C D

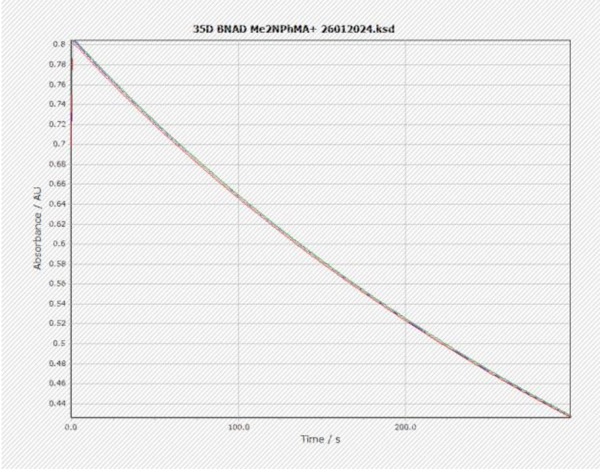

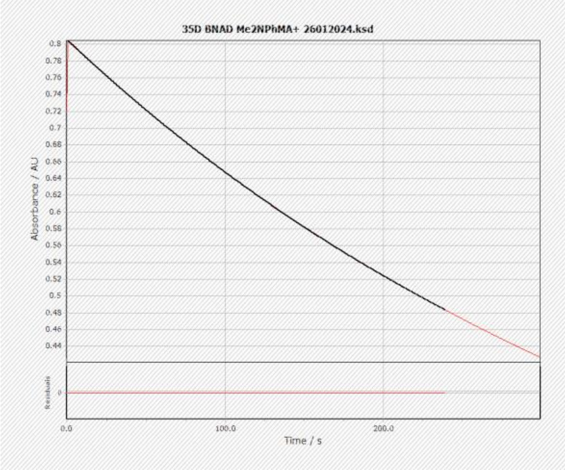

35°C D

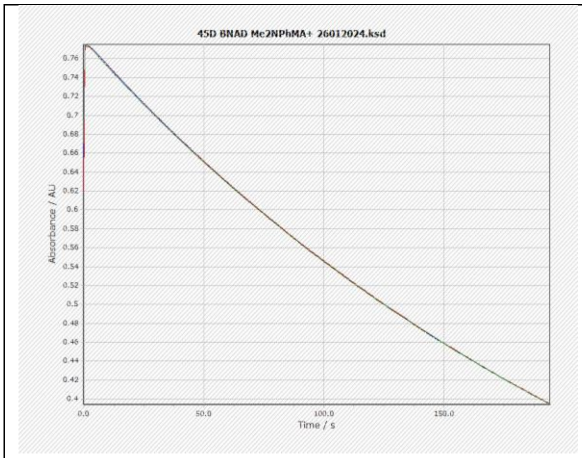	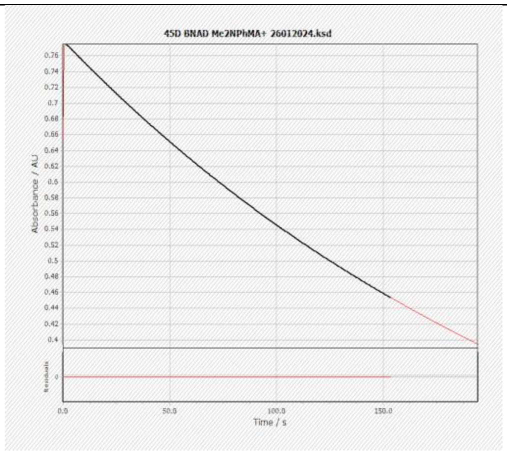
45°C D	
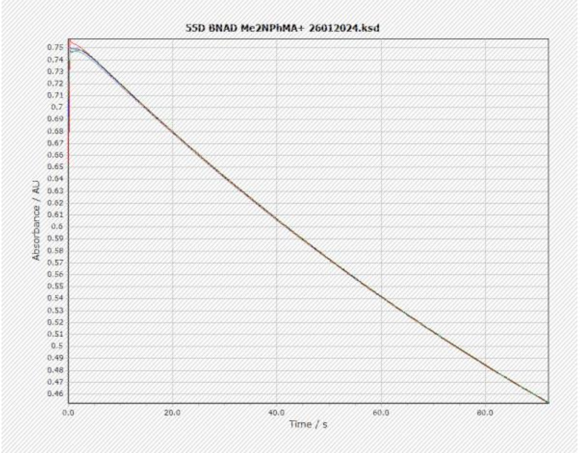	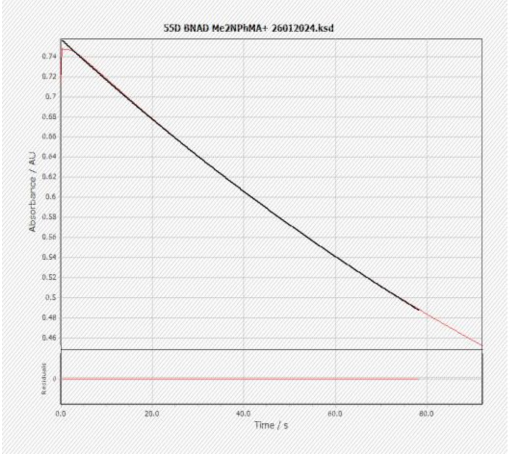
55°C D	

Day 2 data (February 6, 2024)

	Pseudo-first-order rate constants						
	k^{pfo} (s^{-1})						
**Temp (°C)**	**Trial H1**	**Trial H2**	**Trial H3**	**Average k_H^{pfo} (s^{-1})**	**Stdev**	**k_{2H} ($M^{-1}s^{-1}$)**	**Stdev [a]**
55	0.020798	0.021289	0.021519	0.021202	0.000368	3.53	0.061382
45	0.014310	0.014172	0.014069	0.014184	0.000121	2.36	0.020154
35	0.005316	0.005240	0.005168	0.008637	0.000038	1.44	0.006345
25	0.005316	0.005240	0.005168	0.005241	0.000074	0.87	0.012335
15	0.002969	0.002964	0.002944	0.002959	0.000013	0.49	0.002205

**Temp (°C)**	**Trial D1**	**Trial D2**	**Trial D3**	**Average k_D^{pfo} (s^{-1})**	**Stdev**	**k_{2D} ($M^{-1}s^{-1}$)**	**Stdev [a]**
55	0.005589	0.005605	0.005586	0.004943	0.000010	8.24E-01	0.001702
45	0.003428	0.003456	0.003429	0.002990	0.000016	4.98E-01	0.002648
35	0.002062	0.002065	0.002083	0.001796	0.000011	2.99E-01	0.001893
25	0.001203	0.001205	0.001197	0.001033	0.000004	1.72E-01	0.000694
15	0.000664	0.000666	0.000657	0.000567	0.000005	9.44E-02	0.000788

[a] = (Stdev(for k^{pfo})/k^{pfo})*k_{2H}

<table>
<tr><td>Three kinetic runs together with the averaged data</td><td>First-order kinetic fit of the averaged data as an example</td></tr>
<tr><td>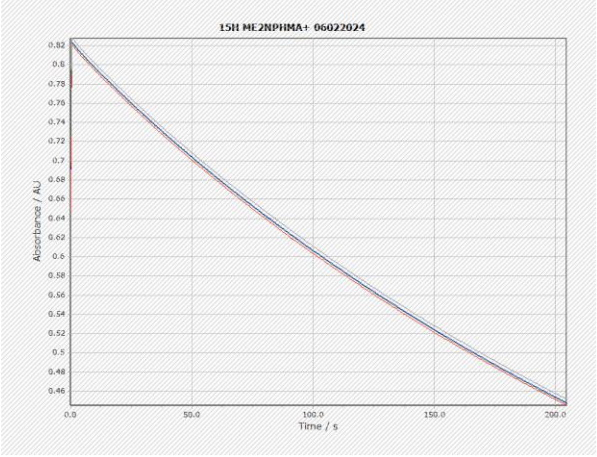
</td><td>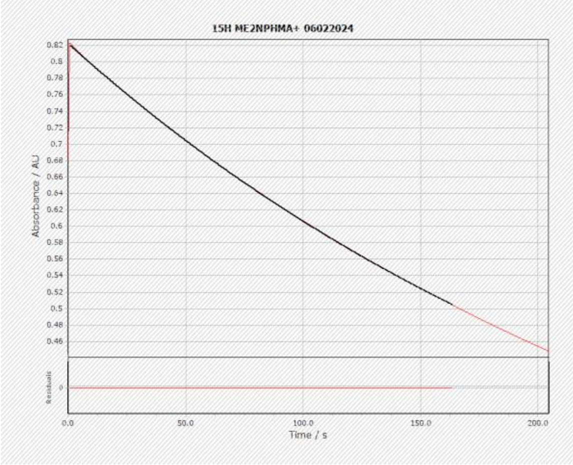
</td></tr>
<tr><td colspan="2">15℃ H</td></tr>
<tr><td>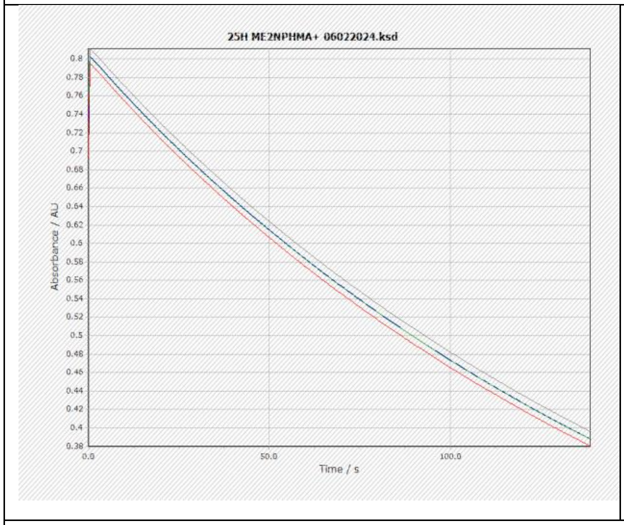
</td><td>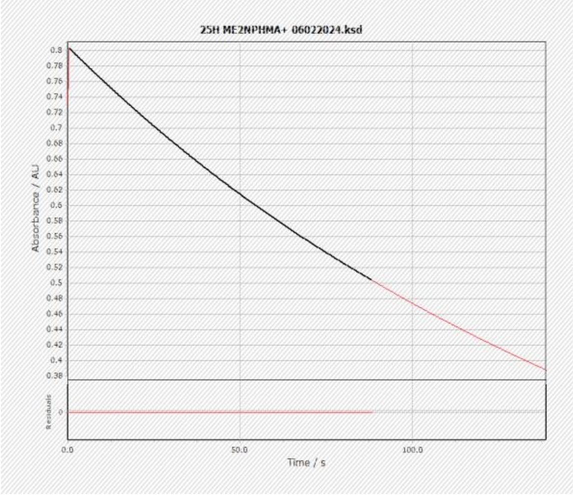
</td></tr>
<tr><td colspan="2">25℃ H</td></tr>
<tr><td>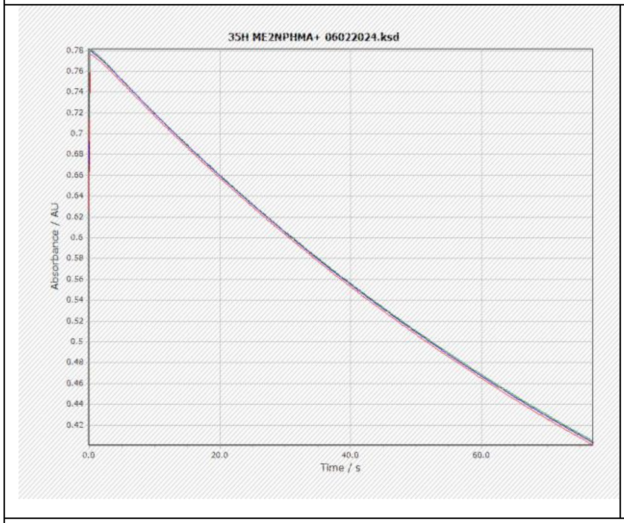
</td><td>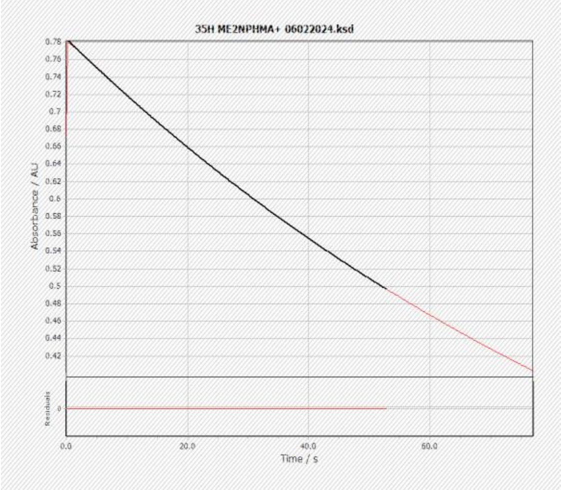
</td></tr>
<tr><td colspan="2">35℃ H</td></tr>
</table>

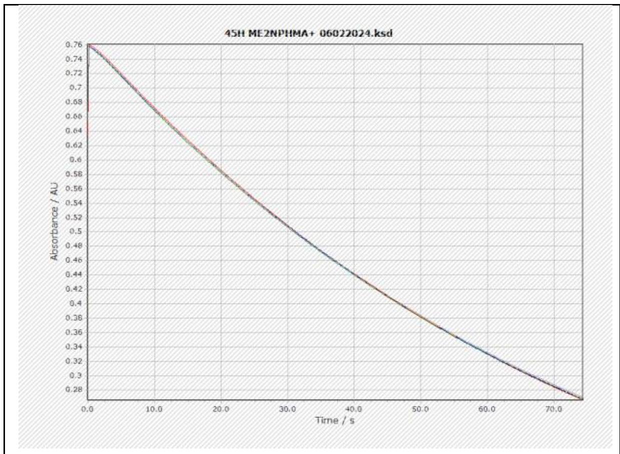
45H ME2NPHMA+ 06022024.ksd
Absorbance / AU
Time / s

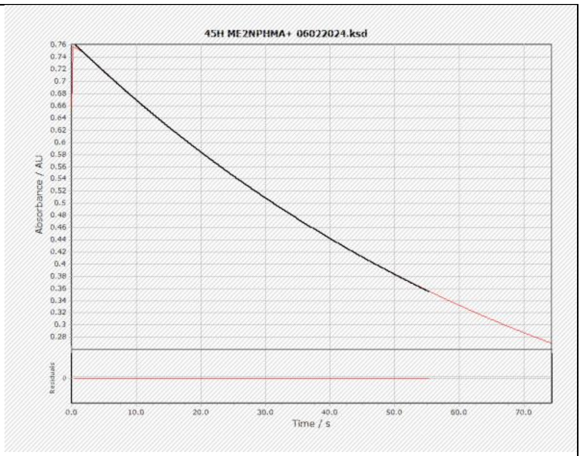
45H ME2NPHMA+ 06022024.ksd
Absorbance / AU
Residuals
Time / s

45℃ H

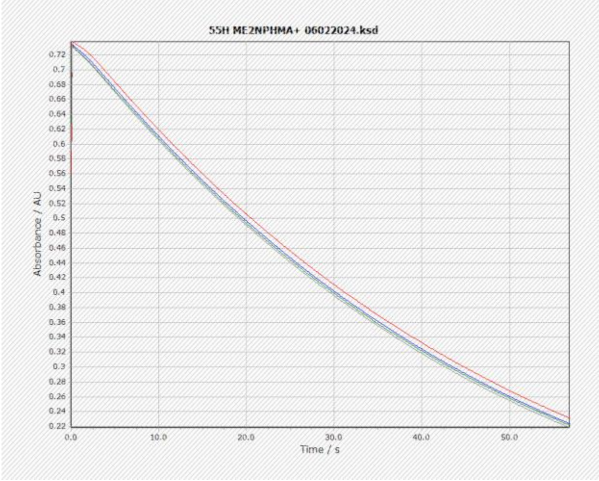
55H ME2NPHMA+ 06022024.ksd
Absorbance / AU
Time / s

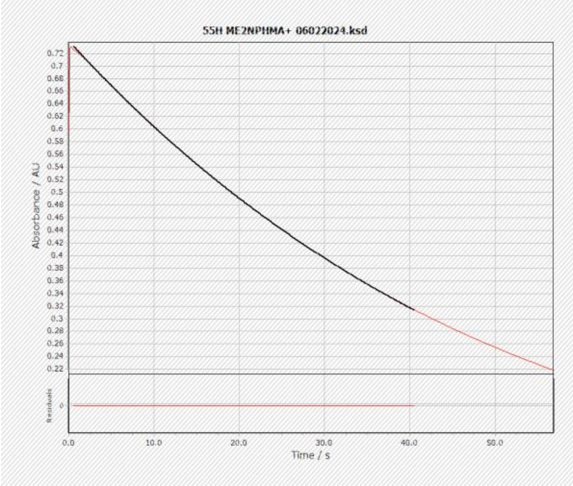
55H ME2NPHMA+ 06022024.ksd
Absorbance / AU
Residuals
Time / s

55℃ H

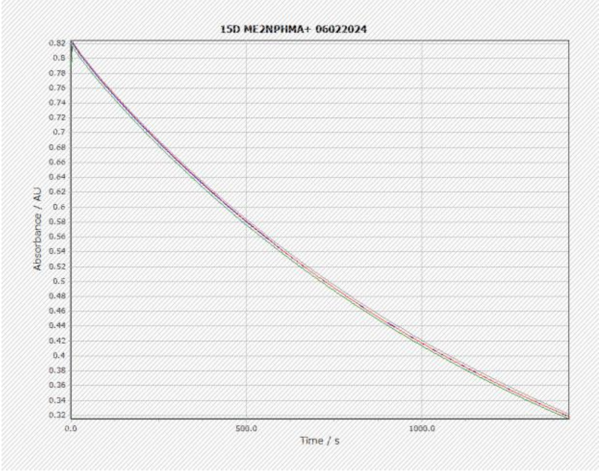
15D ME2NPHMA+ 06022024
Absorbance / AU
Time / s

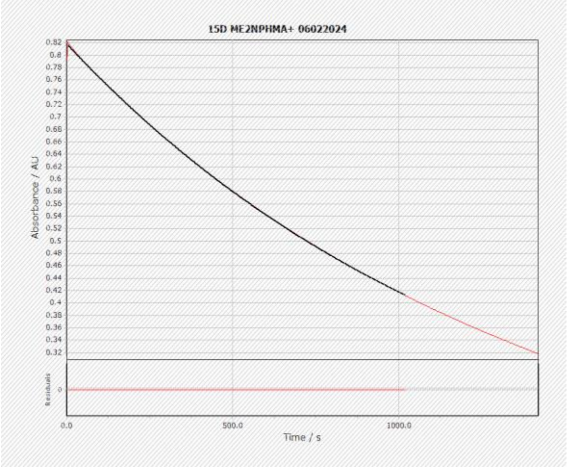
15D ME2NPHMA+ 06022024
Absorbance / AU
Residuals
Time / s

15℃ D

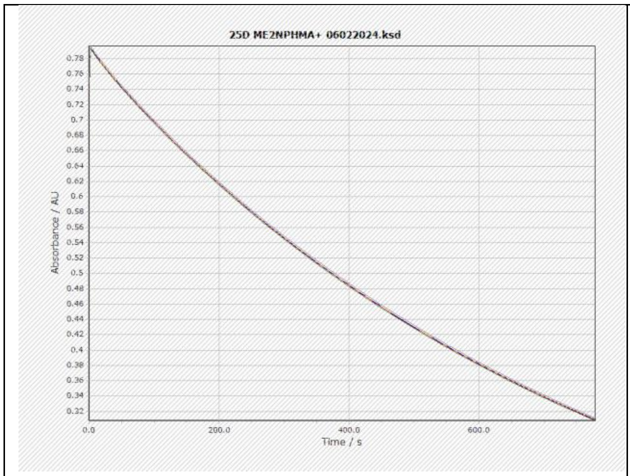

25D ME2NPHMA+ 06022024.ksd
Absorbance / AU
Time / s

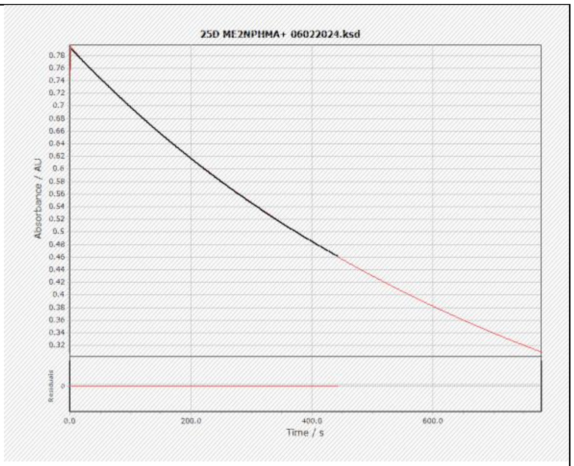

25D ME2NPHMA+ 06022024.ksd
Absorbance / AU
Residuals
Time / s

25℃ D

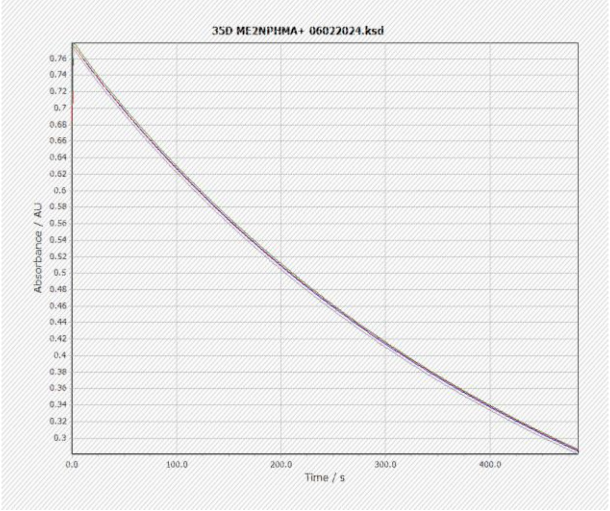

35D ME2NPHMA+ 06022024.ksd
Absorbance / AU
Time / s

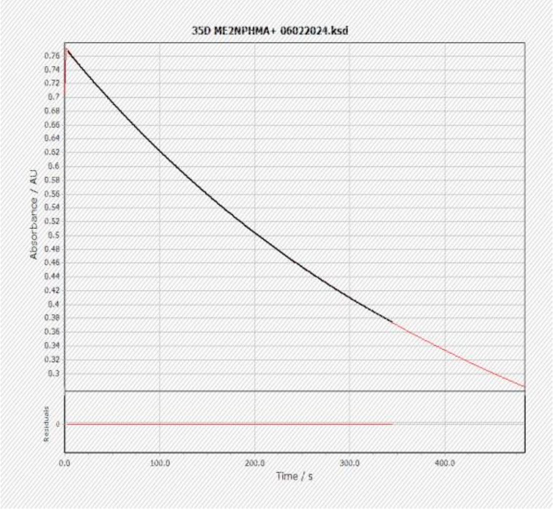

35D ME2NPHMA+ 06022024.ksd
Absorbance / AU
Residuals
Time / s

35℃ D

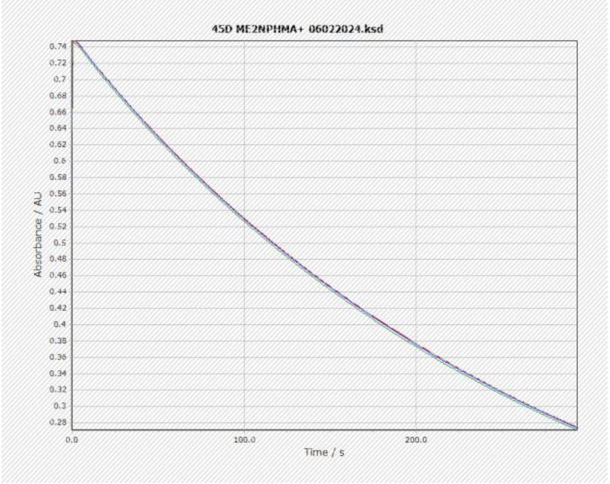

45D ME2NPHMA+ 06022024.ksd
Absorbance / AU
Time / s

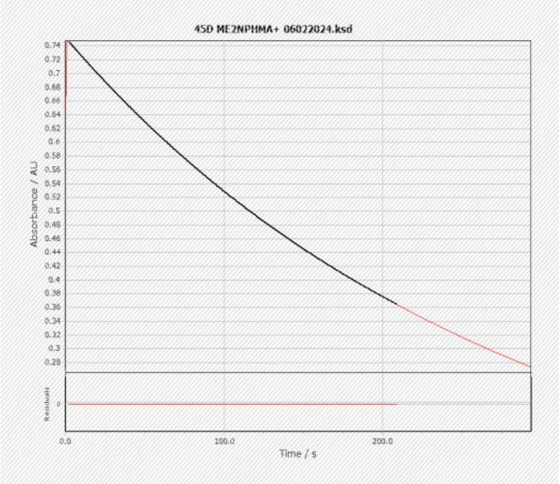

45D ME2NPHMA+ 06022024.ksd
Absorbance / AU
Residuals
Time / s

45℃ D

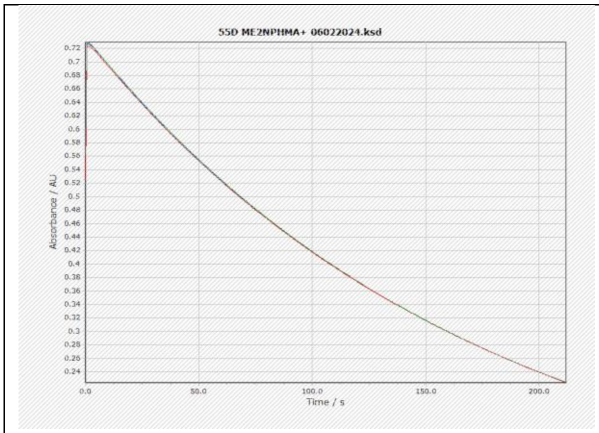

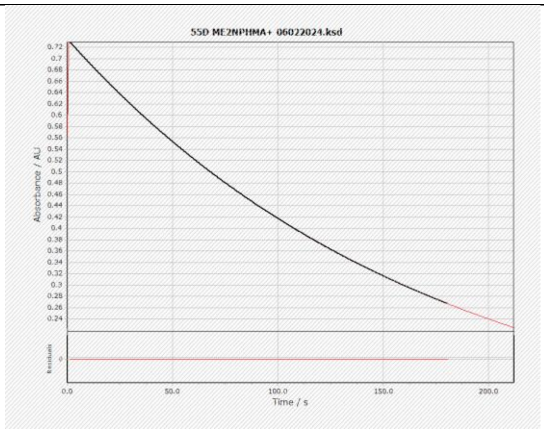

55°C D

Day 3 data (March 3, 2024)

Temp (°C)	Pseudo-first-order rate constants k^{pfo} (s^{-1}) Trial H1	Trial H2	Trial H3	Average k_H^{pfo} (s^{-1})	Stdev	k_{2H} ($M^{-1}s^{-1}$)	Stdev [a]
55	0.018994	0.016225	0.019054	0.019091	0.000120	3.18	0.019977
45	0.012692	0.012650	0.012386	0.012576	0.000187	2.10	0.031113
35	0.007887	0.007943	0.008021	0.007950	0.000055	1.33	0.009192
25	0.004811	0.004844	0.004838	0.004831	0.000004	0.81	0.000707
15	0.002804	0.002792	0.002776	0.002784	0.000011	0.46	0.001886
**Temp (°C)**	**Trial D1**	**Trial D2**	**Trial D3**	**Average k_D^{pfo} (s^{-1})**	**Stdev**	**k_{2D} ($M^{-1}s^{-1}$)**	**Stdev [a]**
55	0.005410	0.005399	0.005357	0.004818	0.000028	8.03E-01	0.004662
45	0.003454	0.003433	0.003451	0.003066	0.000011	5.11E-01	0.001893
35	0.002062	0.002033	0.002023	0.001793	0.000020	2.99E-01	0.003376
25	0.001181	0.001210	0.001204	0.001047	0.000015	1.74E-01	0.002551
15	0.000602	0.000604	0.000592	0.000508	0.000006	8.47E-02	0.001072

[a] = (Stdev(for k^{pfo})/k^{pfo})*k_{2H}

Three kinetic runs together with the averaged data	First-order kinetic fit of the averaged data as an example
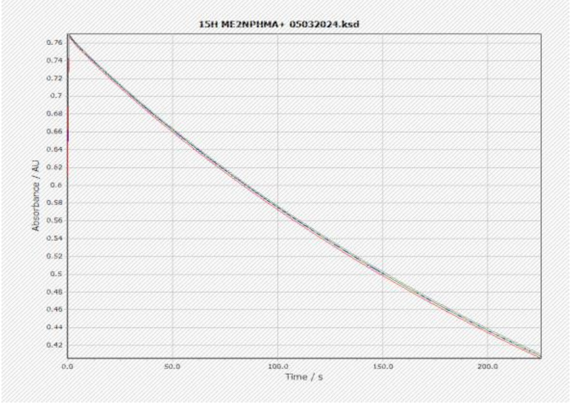	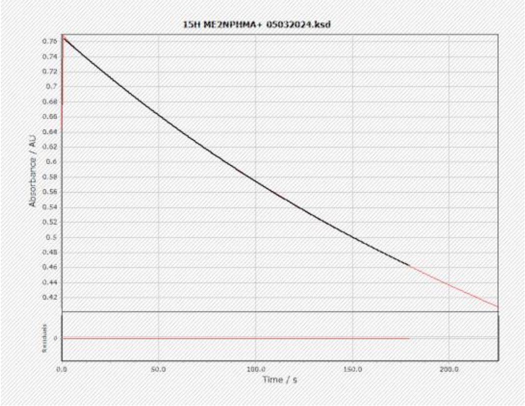

15°C H

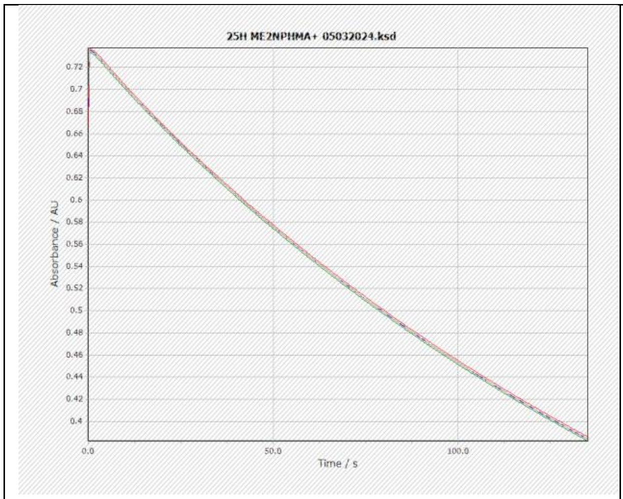
25H ME2NPHMA+ 05032024.ksd
Absorbance / AU
Time / s

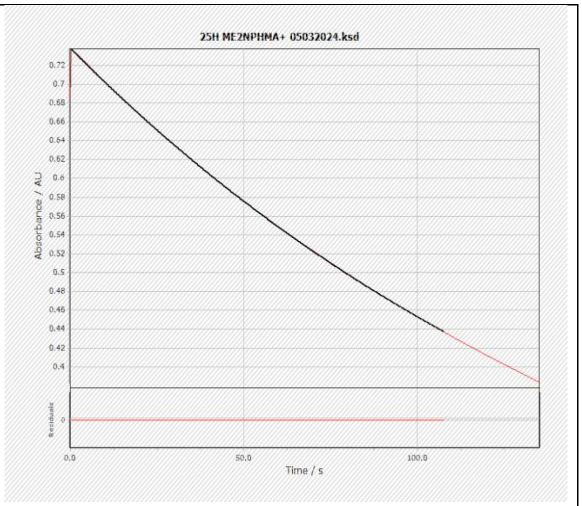
25H ME2NPHMA+ 05032024.ksd
Absorbance / AU
Residuals
Time / s

25℃ H

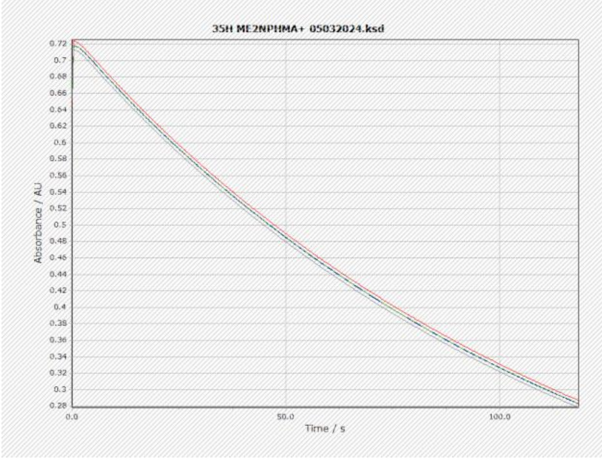
35H ME2NPHMA+ 05032024.ksd
Absorbance / AU
Time / s

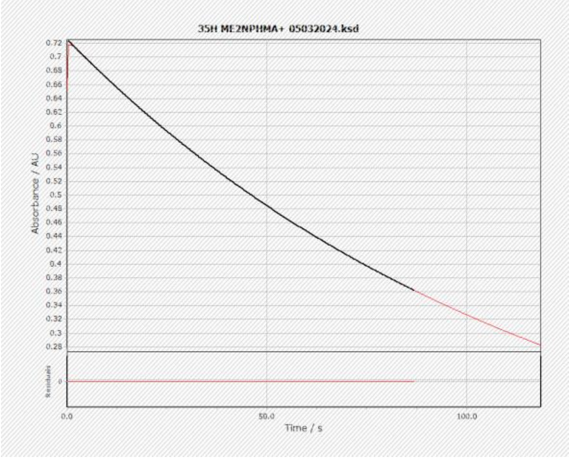
35H ME2NPHMA+ 05032024.ksd
Absorbance / AU
Residuals
Time / s

35℃ H

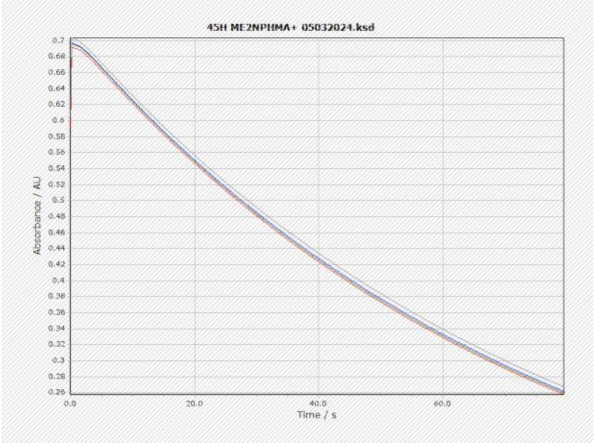
45H ME2NPHMA+ 05032024.ksd
Absorbance / AU
Time / s

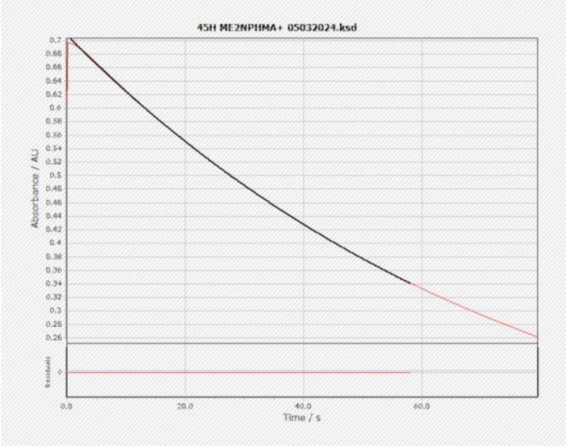
45H ME2NPHMA+ 05032024.ksd
Absorbance / AU
Residuals
Time / s

45℃ H

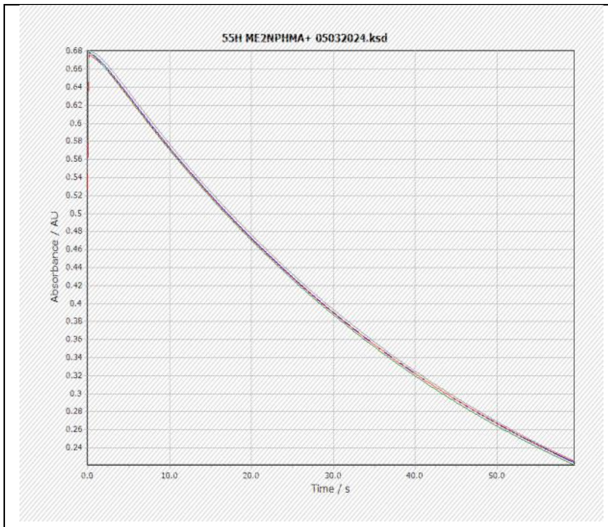

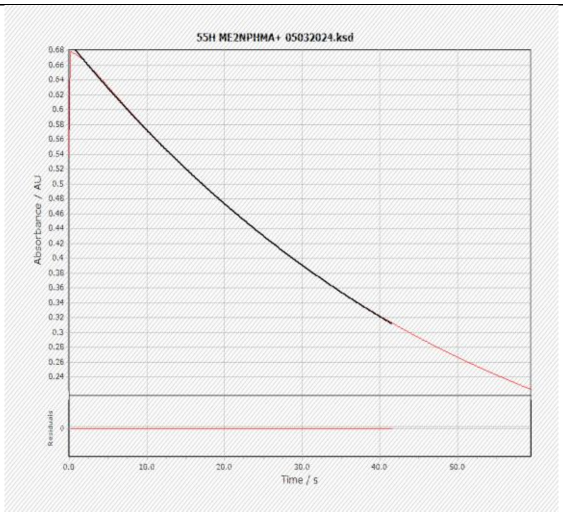

55°C H

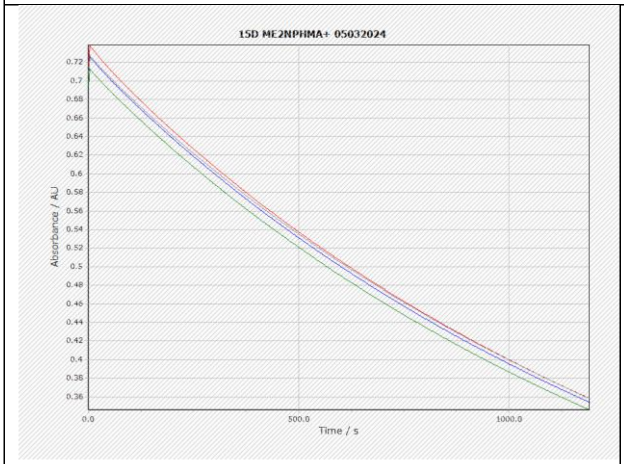

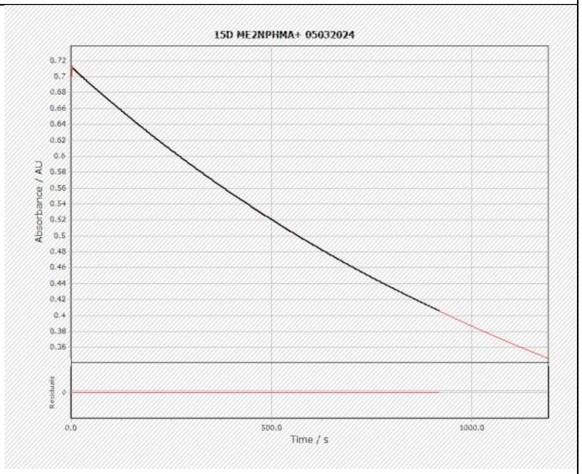

15°C D

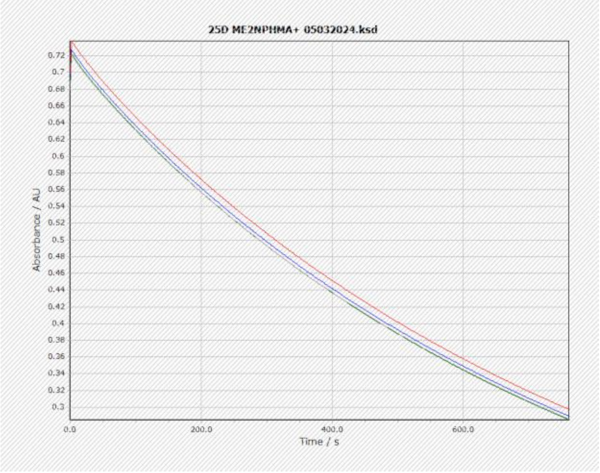

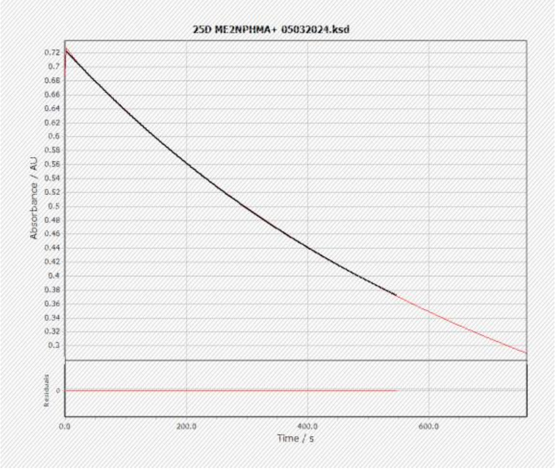

25°C D

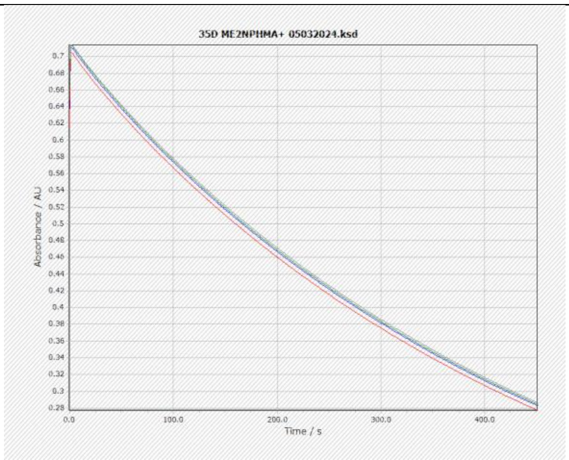

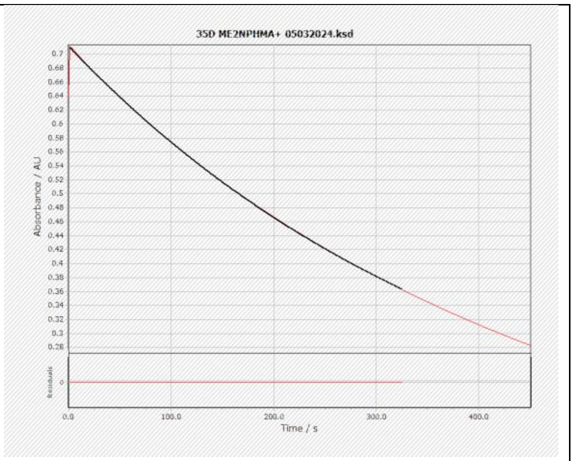

35°C D

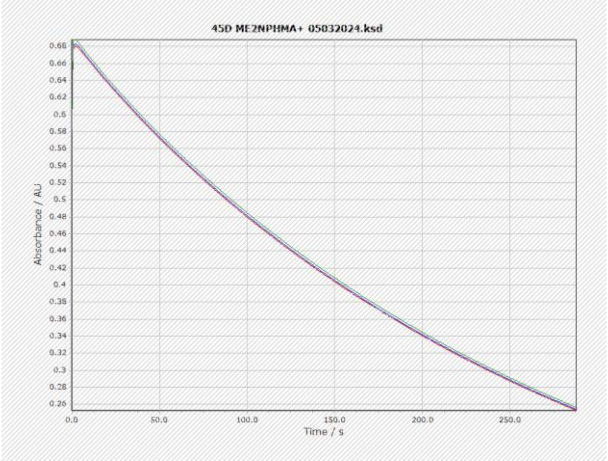

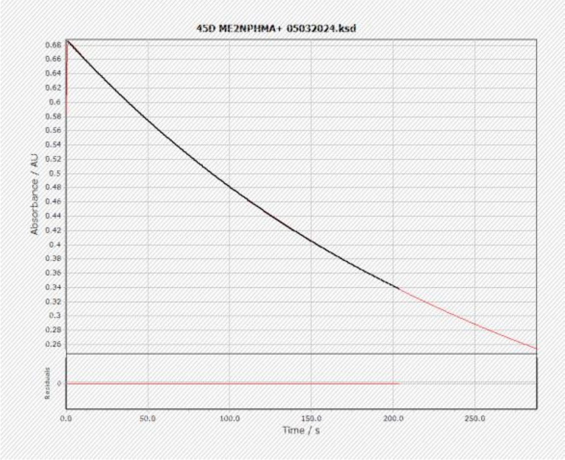

45°C D

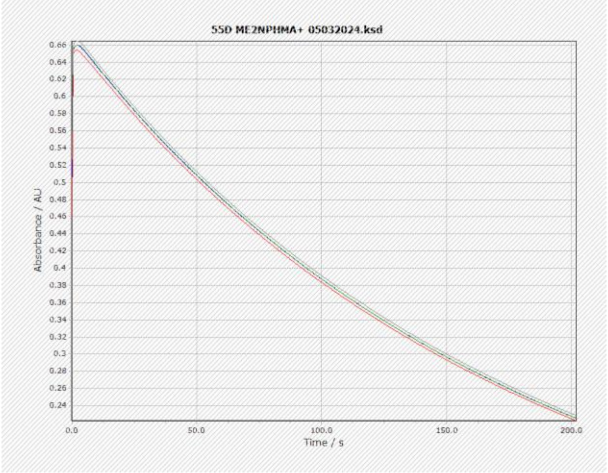

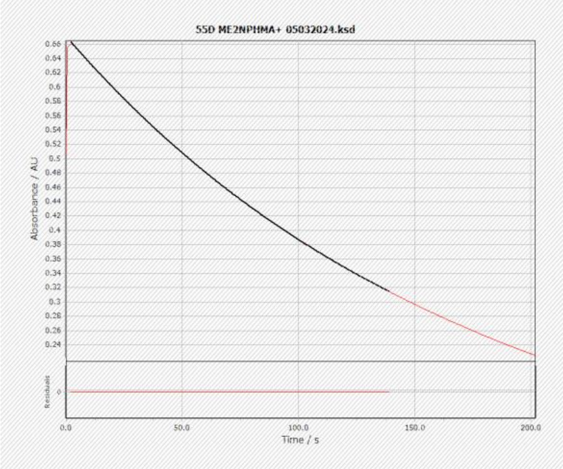

55°C D

Primary kinetic data for the rate constants in Table S7 for *G*PhXn$^+$

Date of Measurements	*G*PhXn$^+$	Pseudo-first-order rate constants k_H^{pfo} (s^{-1})						**Average k_H^{pfo} (s^{-1})**	**Stdev**	**k_{2H} (M^{-1}s^{-1})**	**Stdev [a]**
		**Trial H1**	**Trial H2**	**Trial H3**	**Trial H4**	**Trial H5**	**Trial H6**				
11/25/2024	MeO	3.52125	3.53450	3.56707	3.55793	3.56994	3.59510	3.55763	0.02647	1.78E+03	1.32E+01
11/25/2024	MeO	3.60332	3.57176	3.59032	3.59499	3.63189	3.6349	3.60453	0.02465	1.80E+03	1.23E+01
11/25/2024	MeO	3.40995	3.39107	3.40755	3.38874	3.40810	3.40480	3.40170	0.00932	1.70E+03	4.66E+00
11/25/2024	MeO	3.42524	3.47681	3.44846	3.44852	3.43886	3.44811	3.44767	0.01692	1.72E+03	8.46E+00
10/16/2024	H [b]	7.52611	7.56053	7.60628	7.58128	7.60970	7.55241	7.57272	0.03256	3.79E+03	1.63E+01
10/22/2024	H [b]	7.33278	7.34396	7.29723	7.28320	7.26482	7.34025	7.31037	0.03319	3.66E+03	1.66E+01
11/01/2024	H [b]	7.55722	7.54263	7.59693	7.60923	7.56872	7.53709	7.56864	0.02916	3.78E+03	1.46E+01
12/11/2024	Br	10.9890	10.9522	11.0438	11.0508	10.9563	11.1275	11.0199	0.06736	5.51E+03	3.37E+01
12/11/2024	Br	11.0870	11.1192	11.1482	11.2128	11.0328	11.2273	11.1379	0.07443	5.57E+03	3.72E+01
12/11/2024	Br	11.0793	11.0077	11.1555	11.1655	10.9878	10.8633	11.0432	0.11449	5.52E+03	5.72E+01
12/11/2024	Br	11.0996	11.0836	11.0365	10.9859	11.2381	11.0536	11.0829	0.08576	5.54E+03	4.29E+01
12/11/2024	CF_3	15.4967	15.7241	15.4980	15.6505	15.4853	15.4627	15.5529	0.10743	7.78E+03	5.37E+01
12/11/2024	CF_3	15.7970	15.6654	15.8205	15.5939	15.5823	15.5450	15.6673	0.11651	7.83E+03	5.83E+01
12/11/2024	CF_3	15.4368	15.5614	15.5532	15.5699	15.5423	15.3618	15.5042	0.08517	7.75E+03	4.26E+01
12/11/2024	CF_3	15.5435	15.3936	15.6899	15.6621	15.5675	15.6811	15.5896	0.11374	7.79E+03	5.69E+01
12/11/2024	CN	19.3522	19.4376	19.3970	19.4702	19.3601	19.4453	19.4104	0.04823	9.71E+03	2.41E+01
12/11/2024	CN	19.7442	19.6567	19.5553	19.6589	19.5124	19.6824	19.6350	0.08555	9.82E+03	4.28E+01
12/11/2024	CN	19.3616	19.3654	19.4278	19.2972	19.1553	19.6825	19.3816	0.17417	9.69E+03	8.71E+01
12/11/2024	CN	19.5499	19.4731	19.6423	19.4747	19.4176	19.6363	19.5323	0.09296	9.77E+03	4.65E+01

[a] = (Stdev(for k^{pfo})/k^{pfo})*k_{2H}; [b] data from the T-dependence of KIE studies (see Table S1)

Six runs together with averaged data	First-order kinetic fit of averaged data

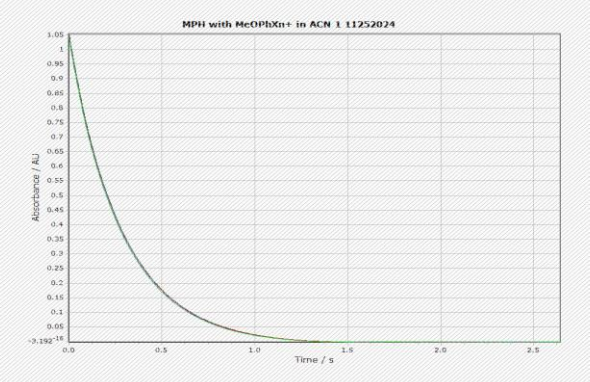

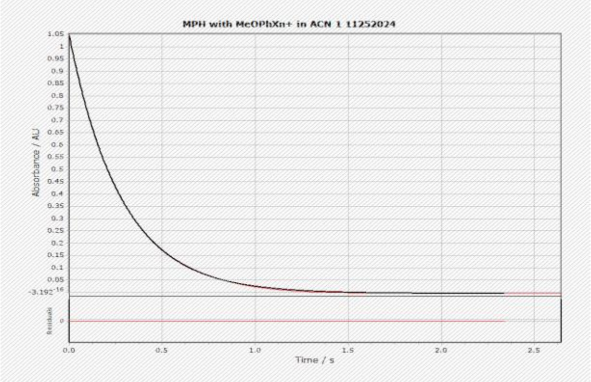

MeOPhXn$^+$

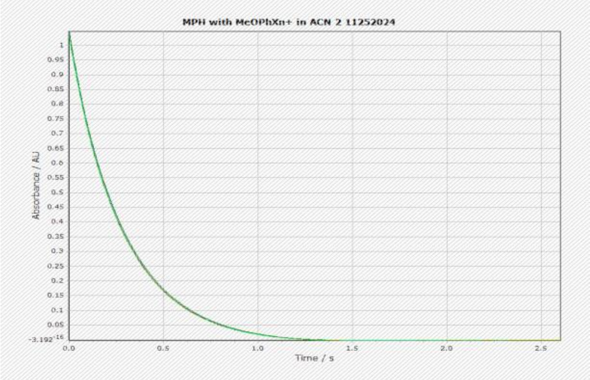

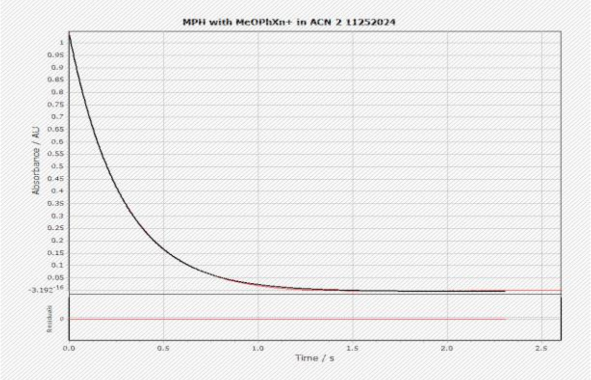

MeOPhXn$^+$

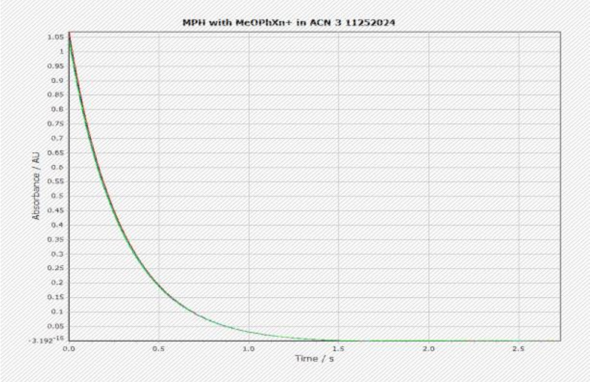

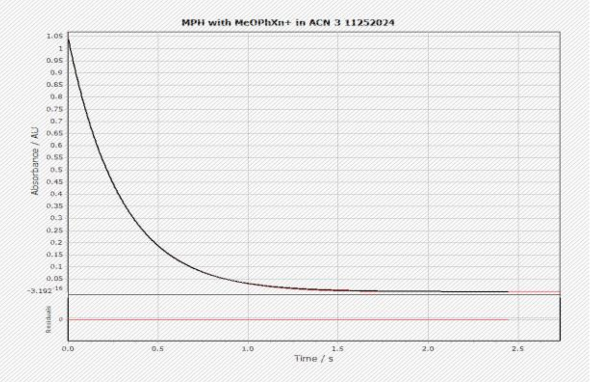

MeOPhXn$^+$

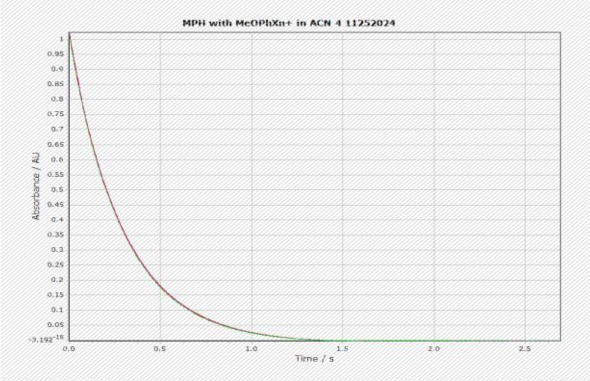

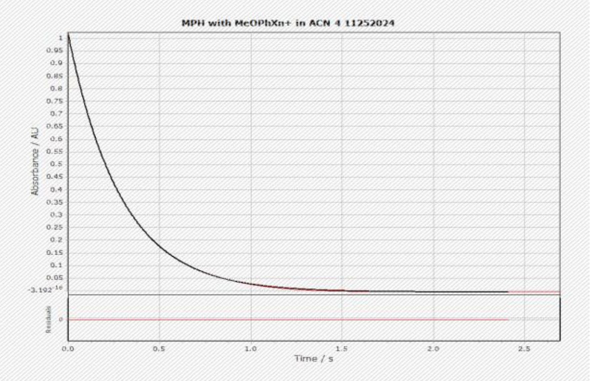

MeOPhXn$^+$

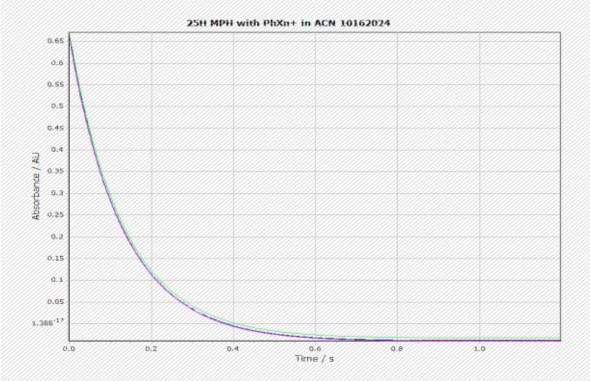

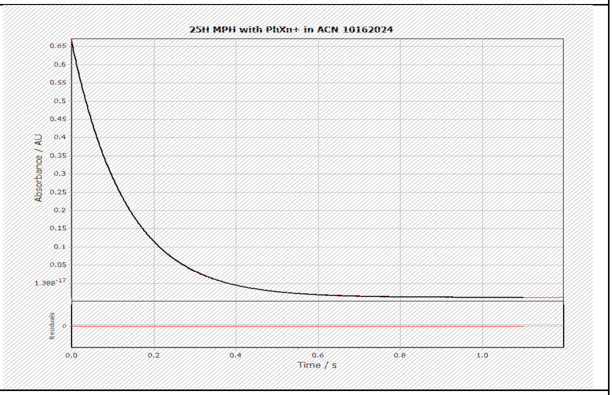

PhXn$^+$

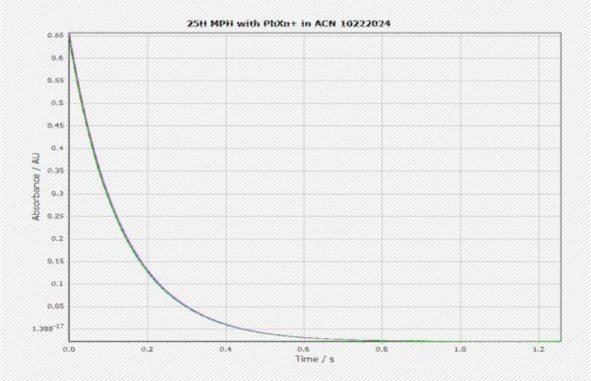

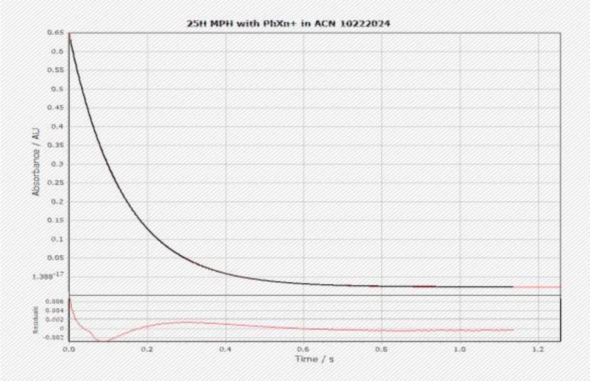

PhXn$^+$

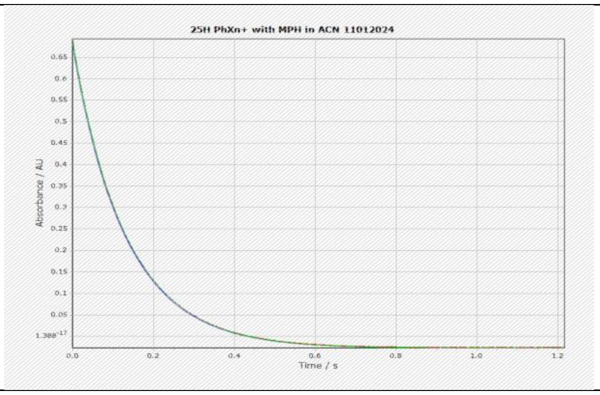

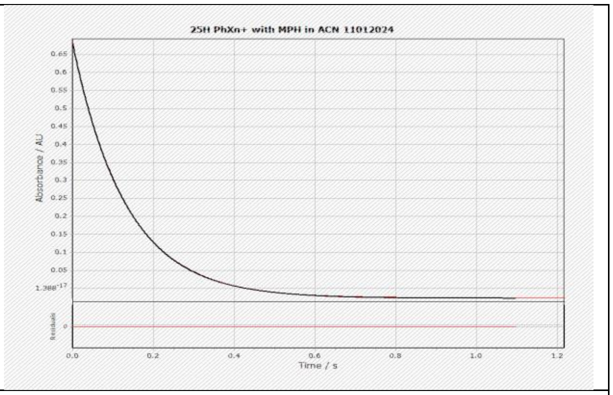

PhXn^{+}

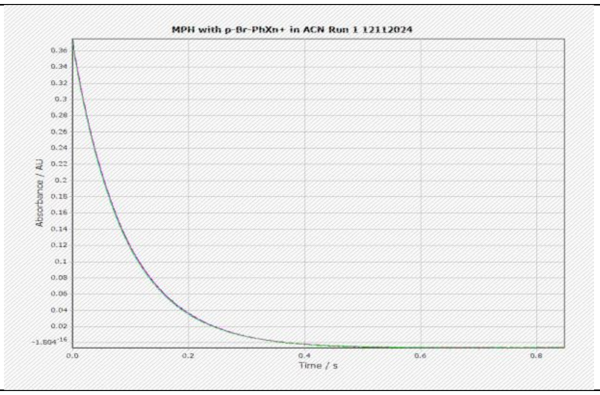

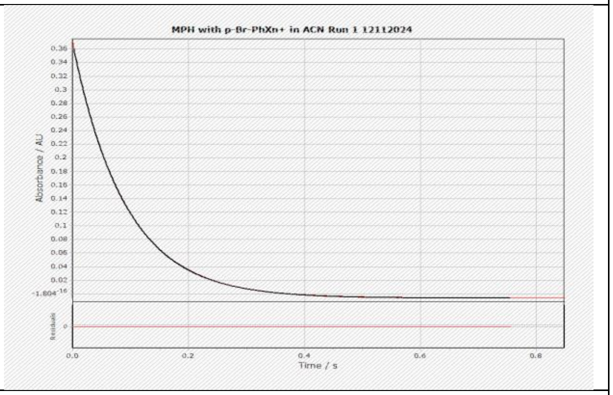

BrPhXn^{+}

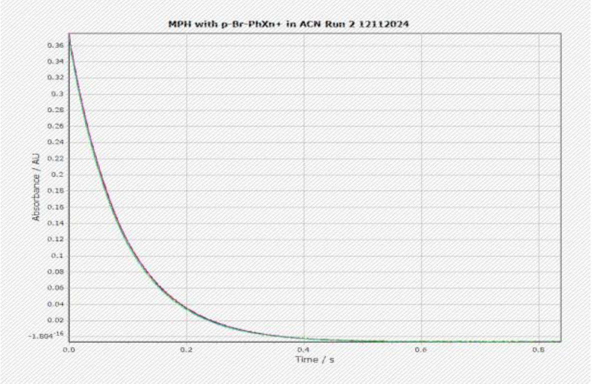

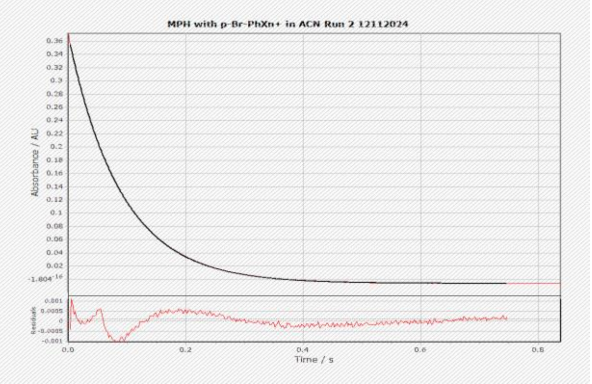

BrPhXn^{+}

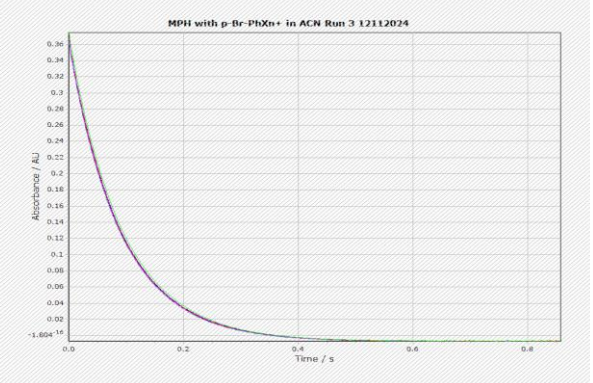

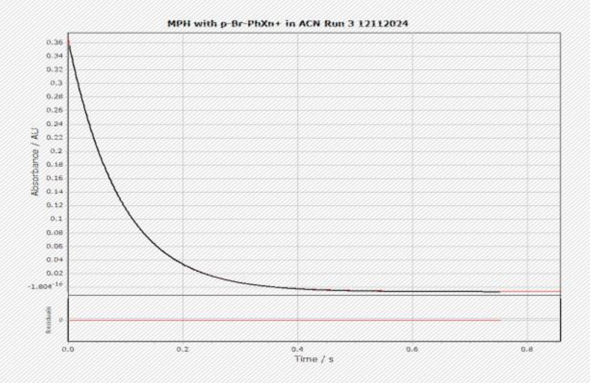

BrPhXn^{+}

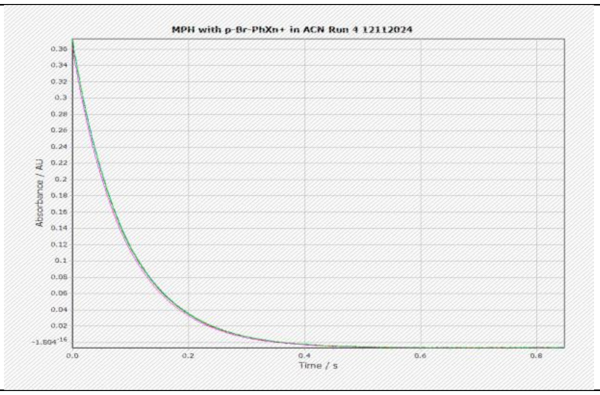	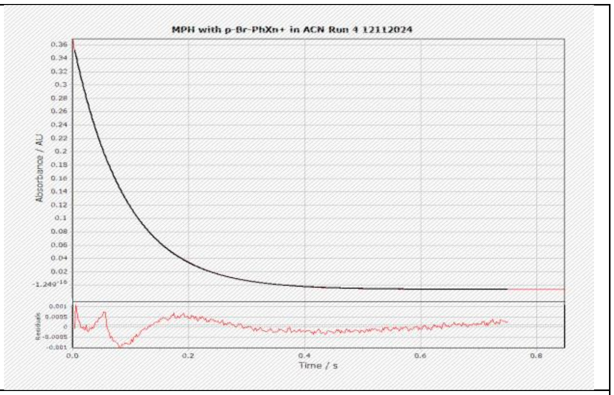
BrPhXn+	
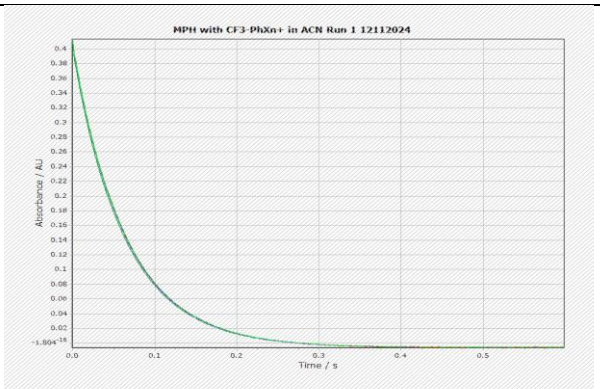	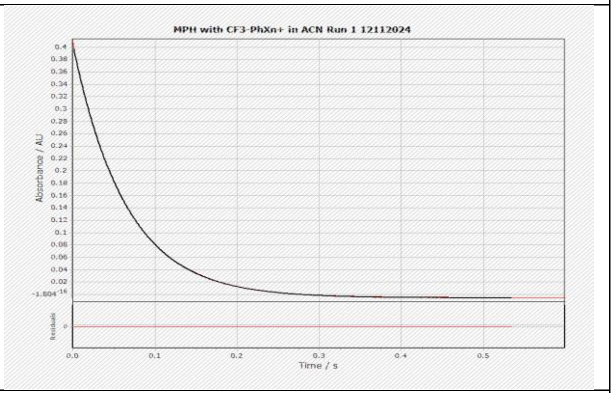
CF_3PhXn^+	
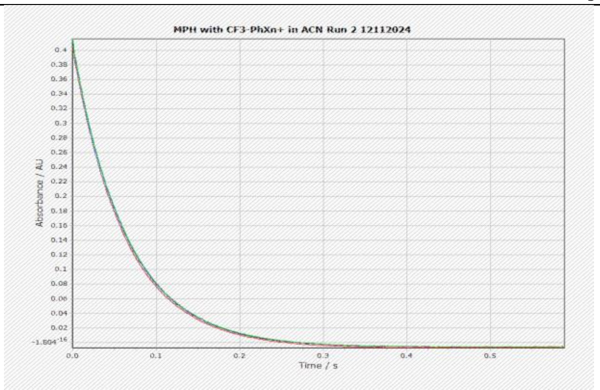	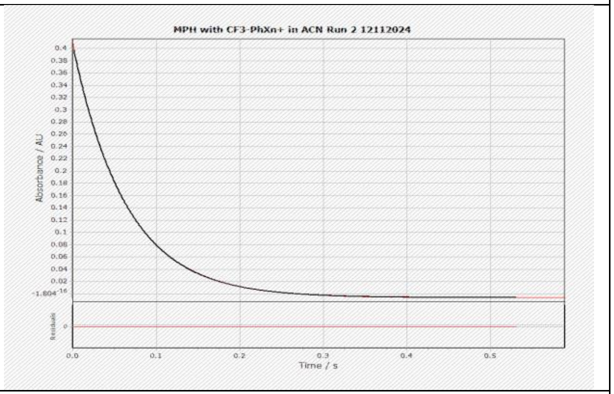
CF_3PhXn^+	
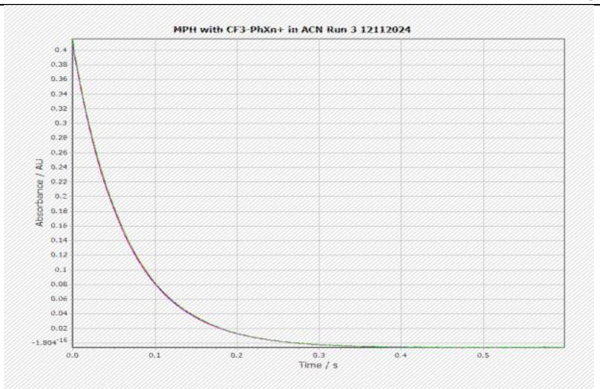	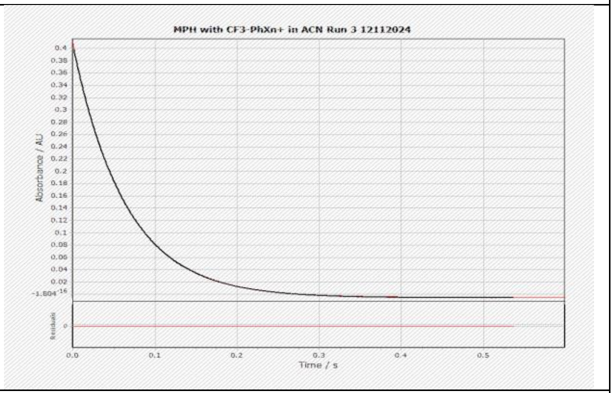
CF_3PhXn^+	

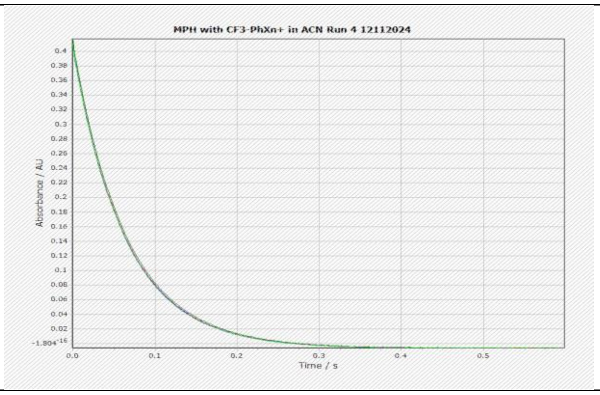	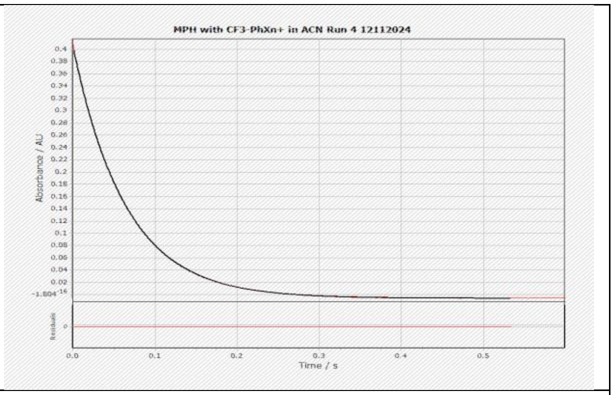
CF_3PhXn^+	
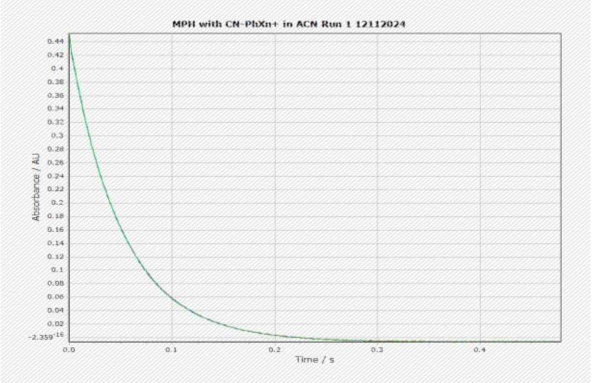	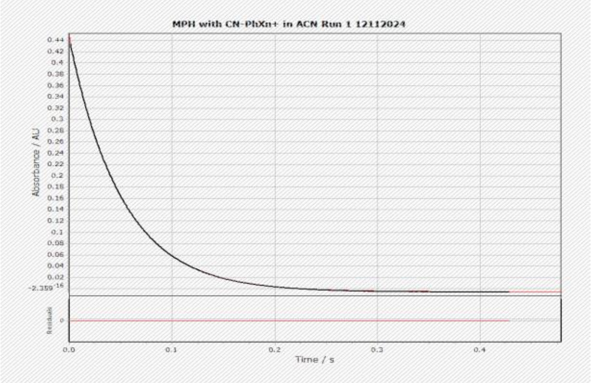
$CNPhXn^+$	
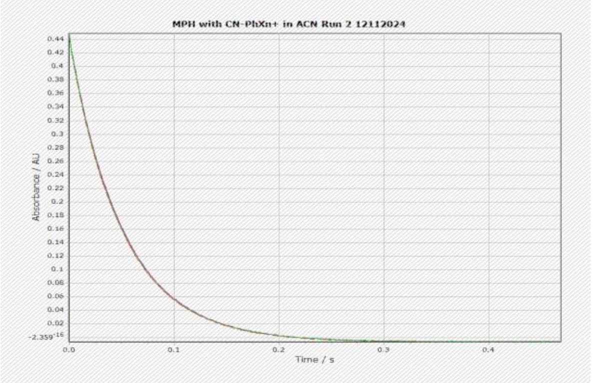	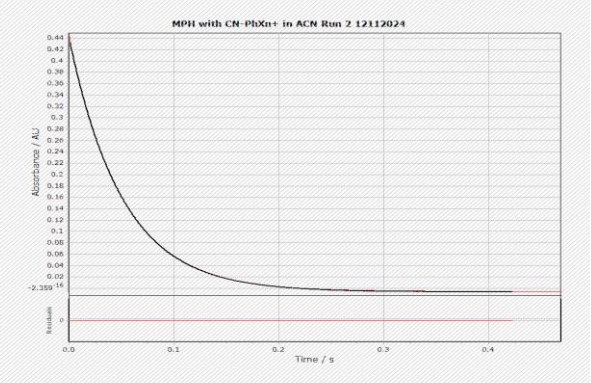
$CNPhXn^+$	
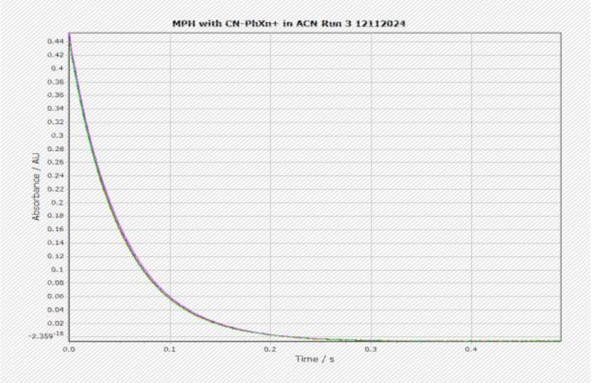	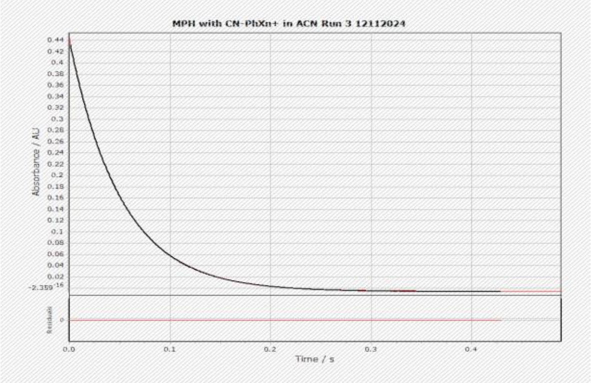
$CNPhXn^+$	

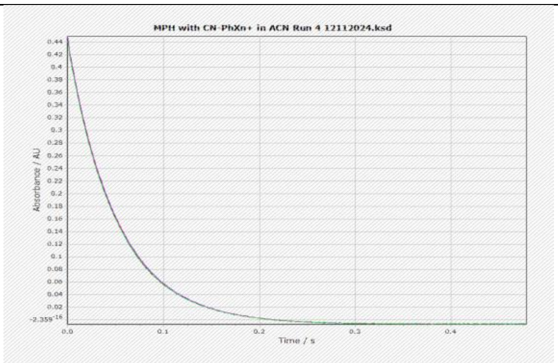	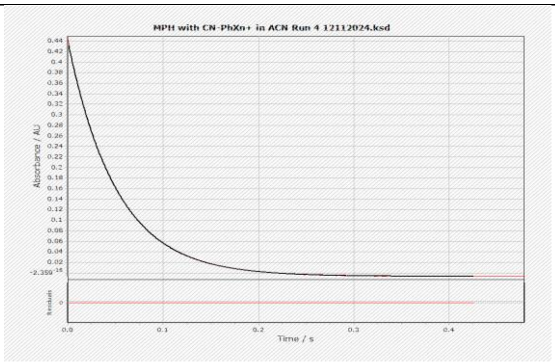
CNPhXn+	

Primary kinetic data for the rate constants in Table S7 for GPhTXn+

Date of Measurements	*G*PhTXn+	Pseudo-first-order rate constants k_H^{pfo} (s^{-1}) Trial H1	Trial H2	Trial H3	Trial H4	Trial H5	Trial H6	Average k_H^{pfo} (s^{-1})	Stdev	k_{2H} ($M^{-1}s^{-1}$)	Stdev [a]
11/26/2024	MeO	18.8136	19.1827	18.8684	18.8713	19.0285	19.0314	18.9660	0.13917	9.48E+03	6.96E+01
11/26/2024	MeO	19.0374	18.8767	19.1792	19.0140	19.2654	19.1205	19.0822	0.13664	9.54E+03	6.83E+01
11/26/2024	MeO	18.9830	18.9098	18.9745	18.9578	18.8426	18.9694	18.9395	0.05407	9.47E+03	2.70E+01
11/26/2024	MeO	18.9811	19.1750	19.0505	18.9294	19.0271	19.1390	19.0504	0.09315	9.53E+03	4.66E+01
11/26/2024	Me	24.5215	24.6414	24.3467	24.5772	24.7245	24.6813	24.5821	0.13621	1.23E+04	6.81E+01
11/26/2024	Me	24.8045	24.7962	24.3207	24.4936	24.5610	25.0249	24.6668	0.25491	1.23E+04	1.27E+02
11/26/2024	Me	24.7370	24.6464	24.2622	24.7323	24.4227	24.4497	24.5417	0.19293	1.23E+04	9.65E+01
11/26/2024	Me	24.7603	24.7430	24.7004	24.8951	24.6205	24.9178	24.7729	0.11445	1.24E+04	5.72E+01
10/23/2024	H [b]	25.8034	25.7478	25.8690	25.7807	26.0115	25.5489	25.7936	0.15193	1.29E+04	7.59E+01
10/30/2024	H [b]	26.6896	26.4046	26.1876	26.3413	26.0852	26.8467	26.4258	0.29226	1.32E+04	1.46E+02
11/06/2024	H [b]	26.8717	26.5236	26.5390	26.4154	26.4086	26.5382	26.5494	0.16887	1.33E+04	8.44E+01
11/26/2024	Cl	34.6370	34.0838	34.9028	34.3124	34.9137	34.9506	34.6334	0.36246	1.73E+04	1.81E+02
11/26/2024	Cl	34.2336	34.6179	35.1420	34.8551	34.9691	34.3908	34.7014	0.34977	1.74E+04	1.75E+02
11/26/2024	Cl	34.8860	34.6526	34.5727	34.7078	34.4179	34.8182	34.6759	0.16920	1.73E+04	8.46E+01
11/26/2024	Cl	34.7652	34.7936	35.0840	34.7366	34.9608	35.2505	34.9318	0.20519	1.75E+04	1.03E+02
12/11/2024	CF_3	42.8680	42.8179	43.0550	42.9929	43.2658	43.1233	43.0205	0.16550	2.15E+04	8.28E+01
12/11/2024	CF_3	43.1657	43.5632	42.8641	43.4440	43.0406	42.8172	43.1492	0.30395	2.16E+04	1.52E+02
12/11/2024	CF_3	42.4769	43.1889	42.4134	42.7862	42.5940	43.0000	42.7432	0.30607	2.14E+04	1.53E+02
12/11/2024	CF_3	43.4564	42.8883	42.6806	42.8491	42.7037	43.2102	42.9647	0.30663	2.15E+04	1.53E+02

[a] = (Stdev(for k^{pfo})/k^{pfo})*k_{2H}; [b] data from the T-dependence of KIE studies (see Table S2)

Six runs together with averaged data	First-order kinetic fit of averaged data
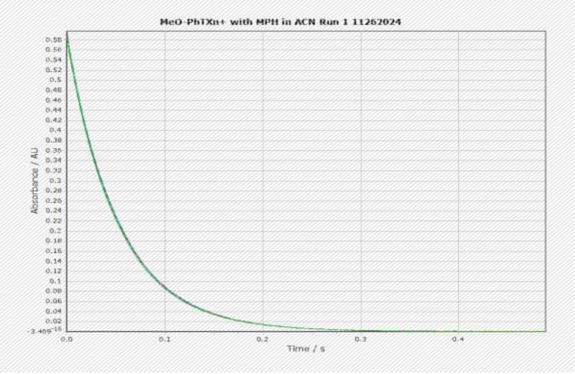	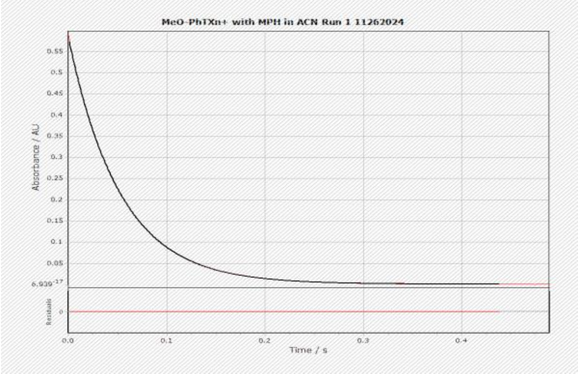
MeOPhTXn+	

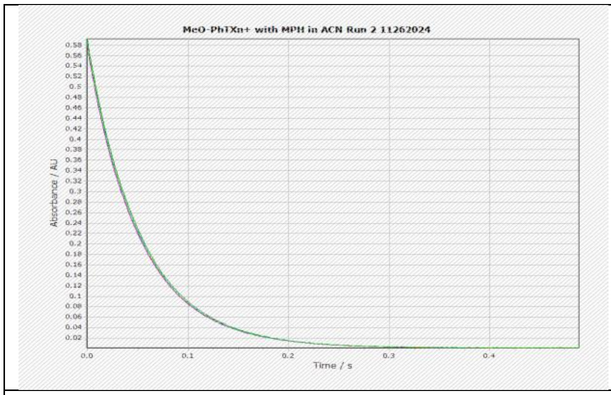

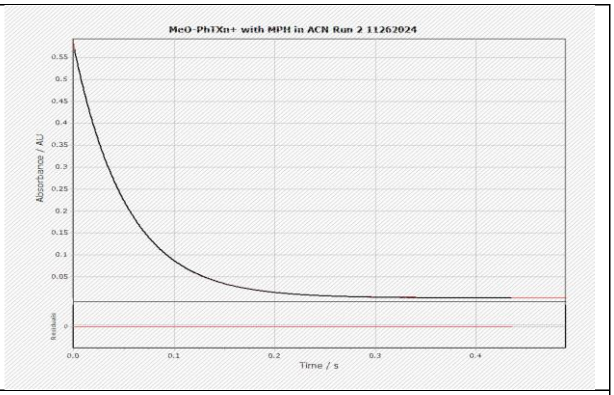

MeOPhTXn$^+$

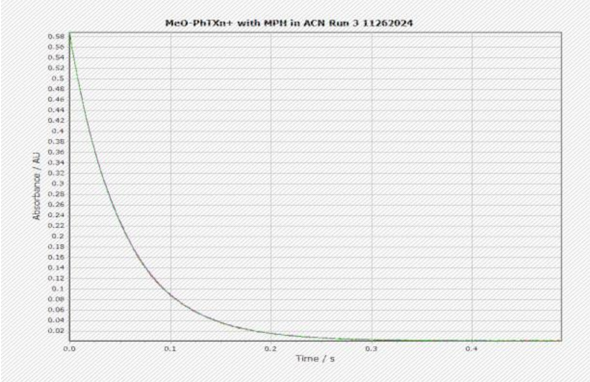

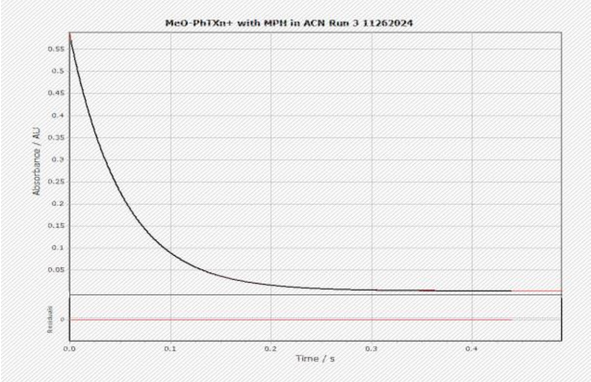

MeOPhTXn$^+$

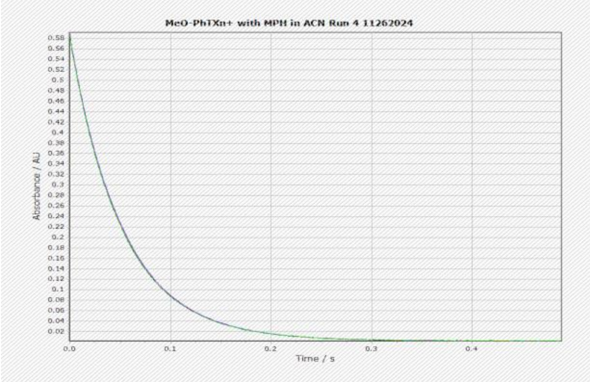

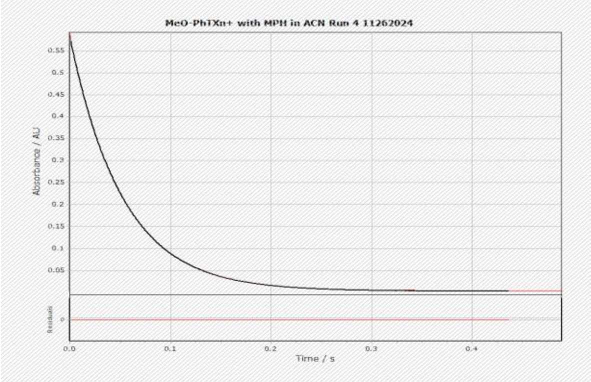

MeOPhTXn$^+$

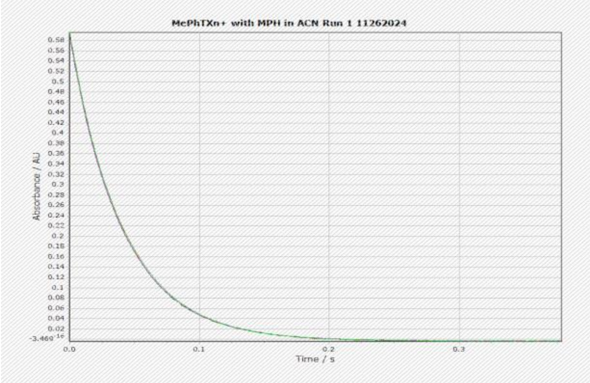

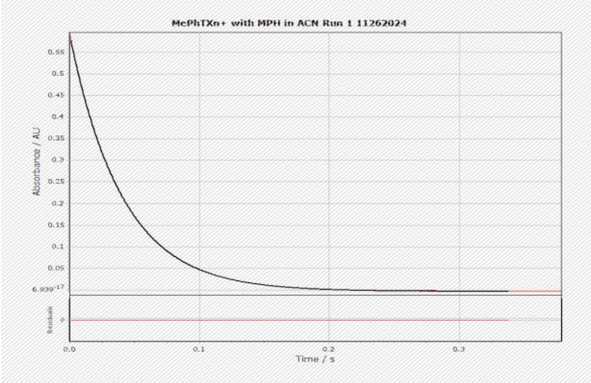

MePhTXn$^+$

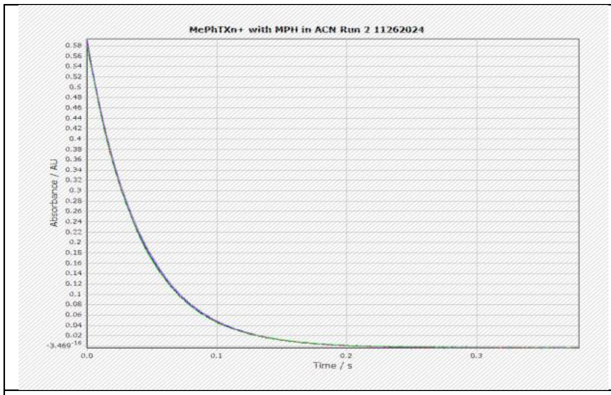

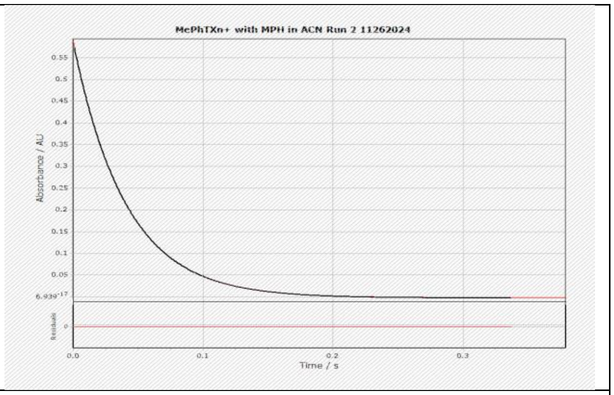

MePhTXn^{+}

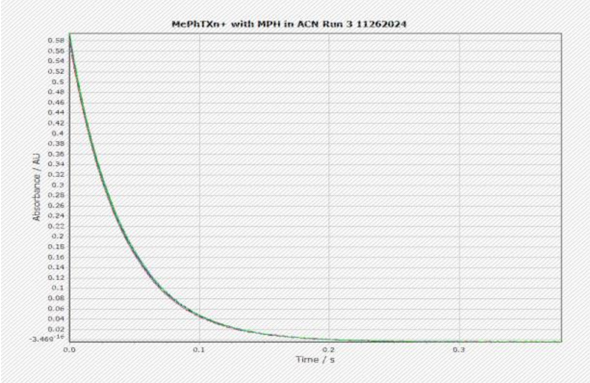

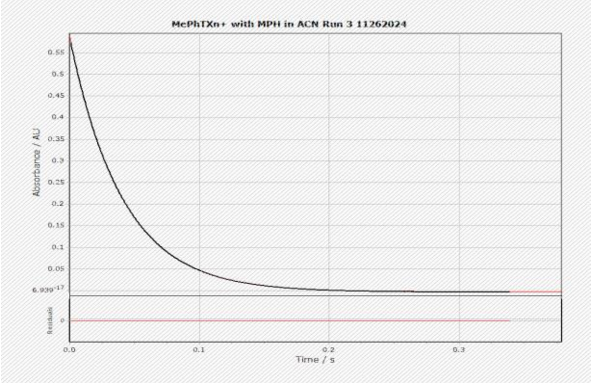

MePhTXn^{+}

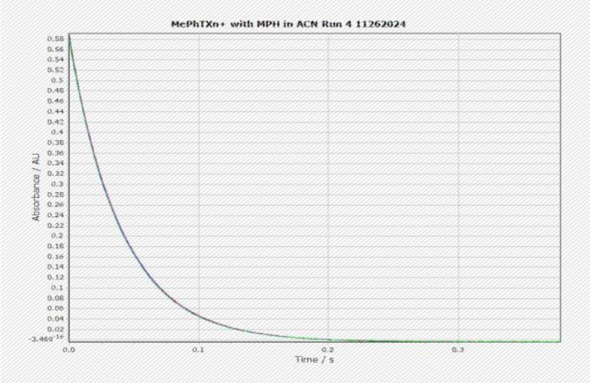

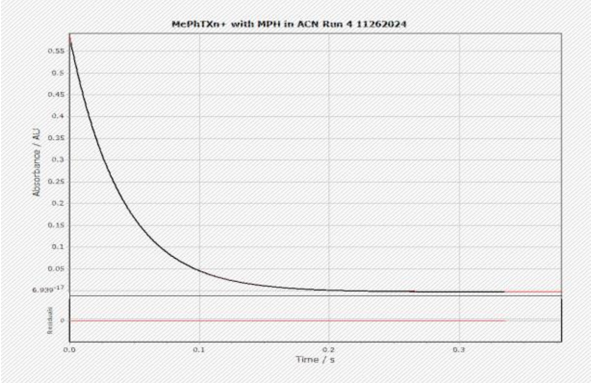

MePhTXn^{+}

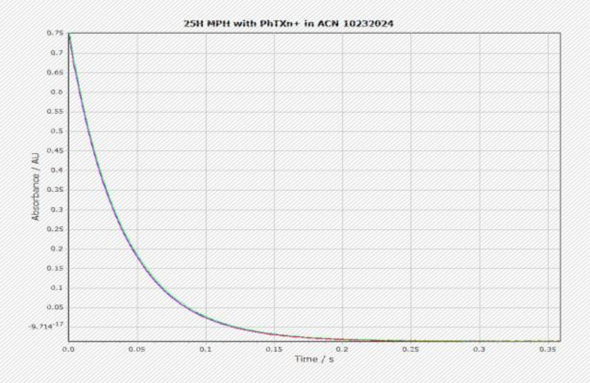

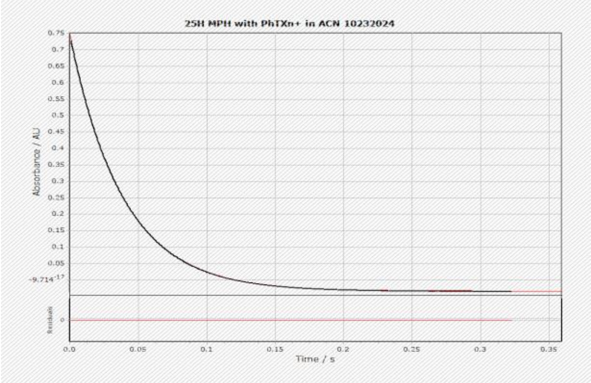

PhTXn^{+}

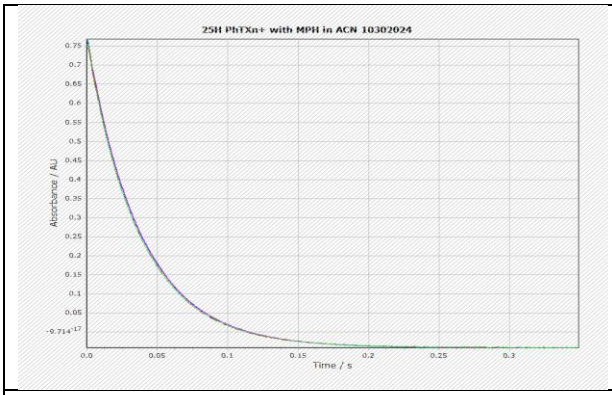

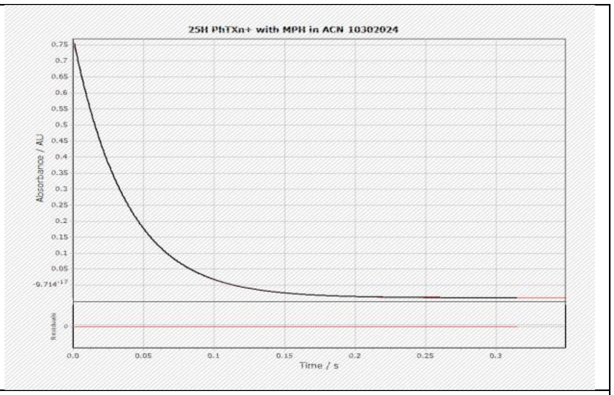

PhTXn$^+$

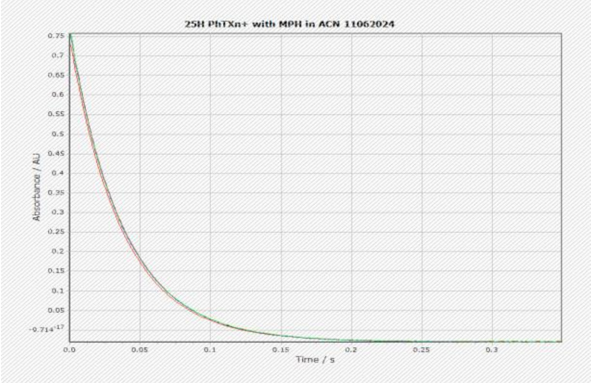

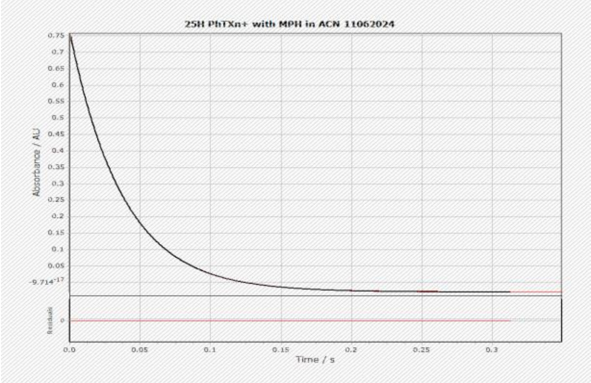

PhTXn$^+$

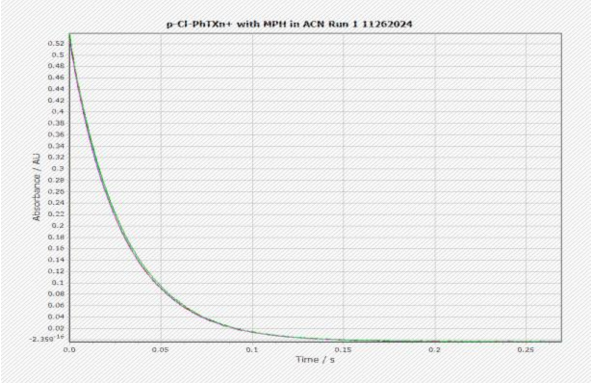

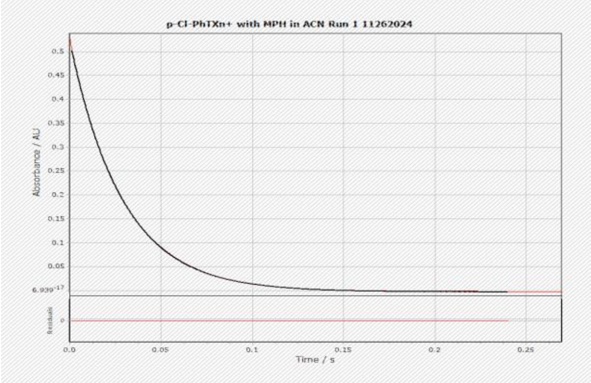

ClPhTXn$^+$

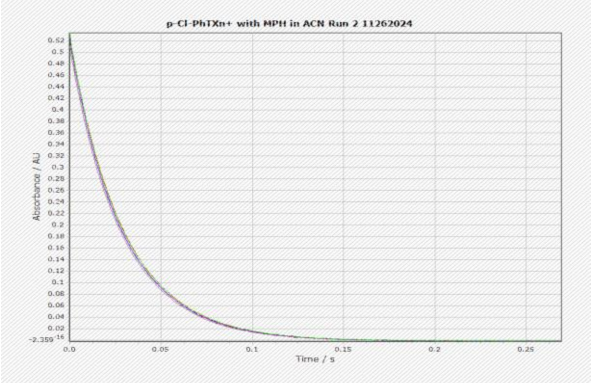

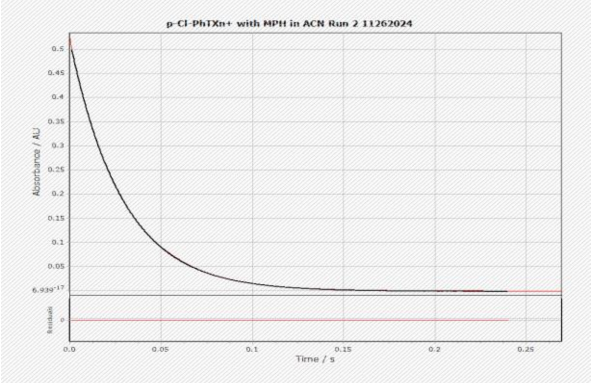

ClPhTXn$^+$

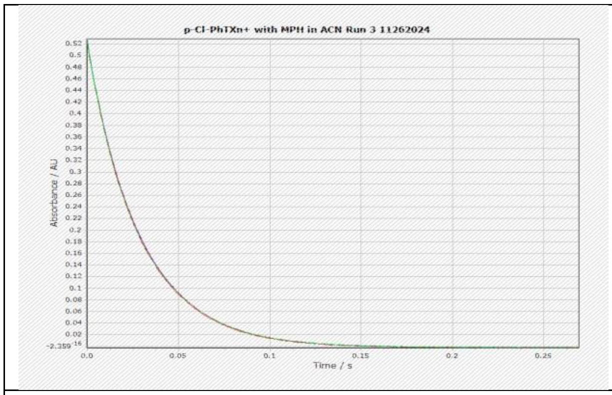

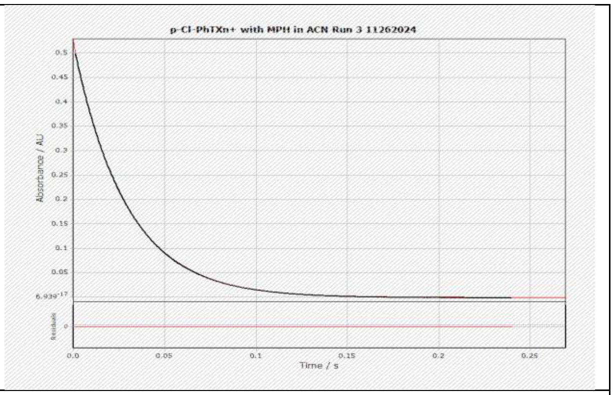

$ClPhTXn^+$

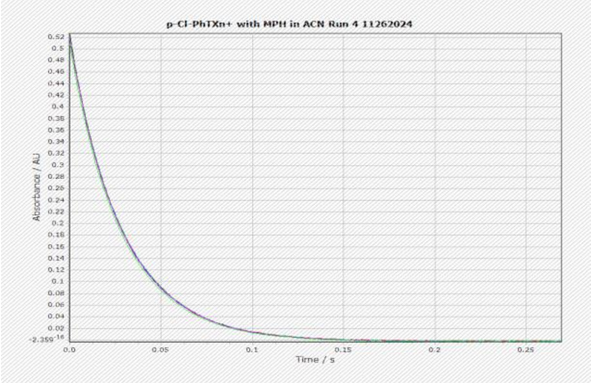

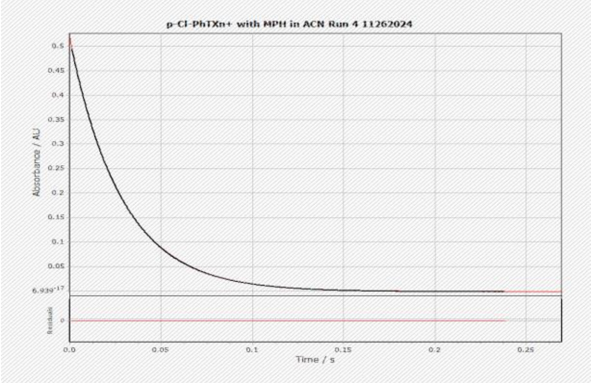

$ClPhTXn^+$

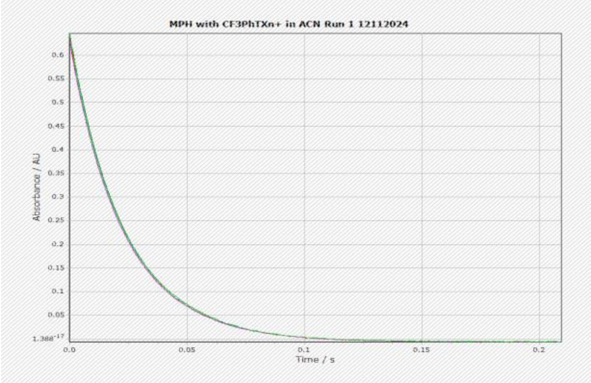

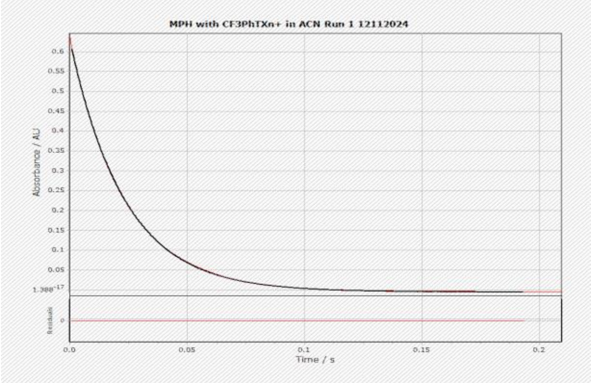

CF_3PhTXn^+

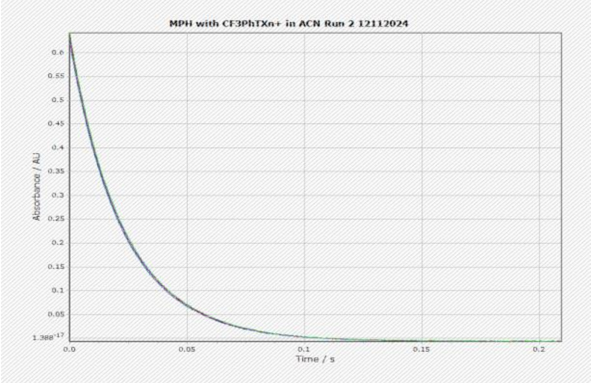

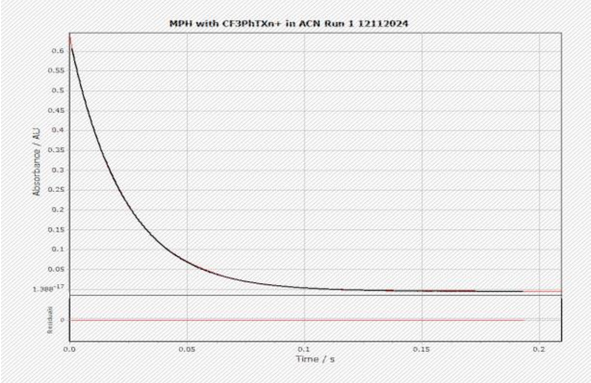

CF_3PhTXn^+

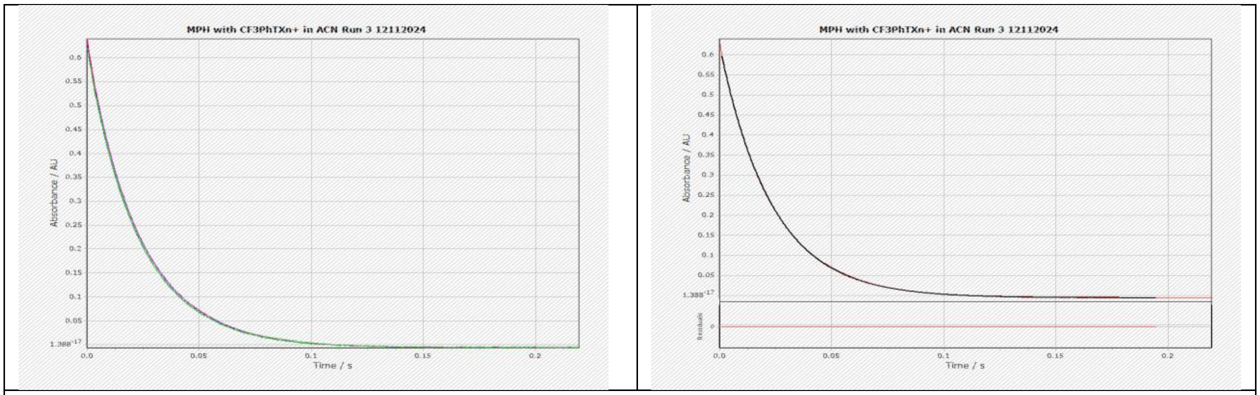

CF_3PhTXn^+

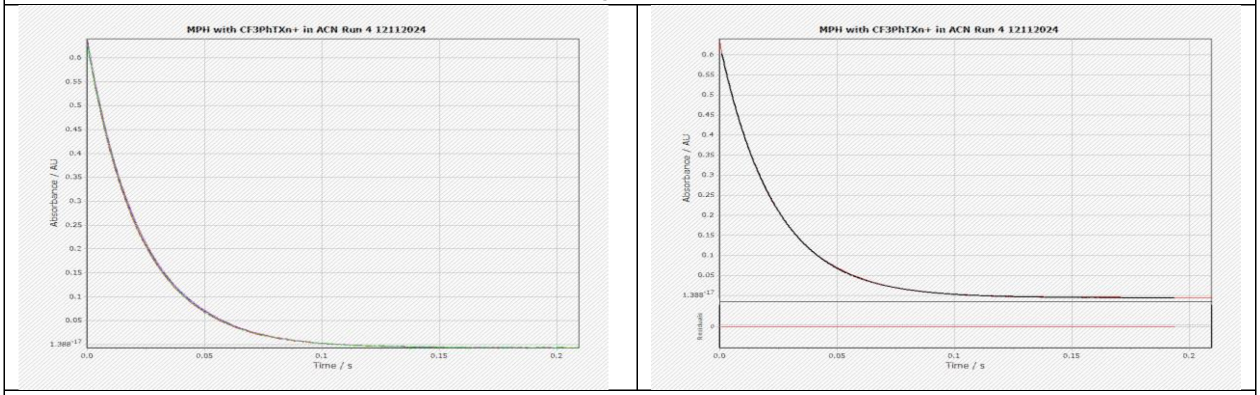

CF_3PhTXn^+

Primary kinetic data for the rate constants of *G*PhXn$^+$ in Table S8

Date of Measure-ments	***G*PhXn$^+$**	Pseudo-first-order rate constants k_H^{pfo} (s^{-1}) **Trial H1**	**Trial H2**	**Trial H3**	**Trial H4**	**Trial H5**	**Trial H6**	**Average k_H^{pfo} (s^{-1})**	**Stdev**	**k_{2H} (M^{-1}s^{-1})**	**Stdev [a]**
09/24/2024	MeO	0.3056	0.2986	0.2994	0.2981	0.3012	0.3054	0.3014	0.0034	150.6917	1.6863
09/24/2024	MeO	0.2939	0.2967	0.2954	0.2974	0.3005	0.3021	0.2977	0.0031	148.8375	1.5418
09/26/2024	MeO	0.2977	0.2983	0.2996	0.3004	0.3012	0.3007	0.2996	0.0014	149.8208	0.6948
09/26/2024	MeO	0.3088	0.3015	0.3057	0.3060	0.3049	0.3038	0.3051	0.0024	152.5492	1.2133
11/12/2023	H [b]	2.2562	2.2786	2.2672	2.2556	2.2715	2.2828	2.2686	0.0113	378.1069	1.8767
11/17/2023	H [b]	1.1453	1.1503	1.1577	1.1429	1.1557	1.1565	1.1514	0.0062	383.7967	2.0808
11/22/2023	H [b]	1.1325	1.1198	1.1292	1.1315	1.1216	1.1231	1.1263	0.0055	375.4256	1.8228
12/11/2024	Br	1.2609	1.2430	1.2509	1.2464	1.2438	1.2425	1.2479	0.0071	623.9517	3.5457
12/11/2024	Br	1.2625	1.2450	1.2324	1.2434	1.2561	1.2401	1.2466	0.0110	623.2883	5.4804
12/11/2024	Br	1.1533	1.1510	1.1527	1.1538	1.1483	1.1618	1.1535	0.0045	576.7433	2.2708
12/11/2024	Br	1.1694	1.1575	1.1408	1.1645	1.1606	1.1536	1.1577	0.0100	578.8608	4.9779
12/11/2024	CF_3	1.9686	1.9759	1.9683	1.9515	1.9806	1.9672	1.9687	0.0099	984.3408	4.9594
12/11/2024	CF_3	1.9607	1.9570	1.9577	1.9584	1.9565	1.9791	1.9616	0.0087	980.7783	4.3573
12/11/2024	CF_3	1.7160	1.7015	1.7172	1.7033	1.7383	1.7429	1.7199	0.0174	859.9392	8.6760
12/11/2024	CF_3	1.7890	1.7909	1.7856	1.8017	1.7887	1.7985	1.7924	0.0063	896.2033	3.1446
12/11/2024	CN	2.6069	2.6006	2.5707	2.5833	2.5747	2.6034	2.5899	0.0157	1294.967	7.8352
12/11/2024	CN	2.5684	2.5550	2.5467	2.5691	2.5765	2.5712	2.5645	0.0112	1282.225	5.6225
12/11/2024	CN	2.3789	2.3538	2.3865	2.3605	2.3417	2.3575	2.3631	0.0166	1181.571	8.2985
12/11/2024	CN	2.3535	2.3755	2.3505	2.3495	2.3548	2.3519	2.3559	0.0098	1177.970	4.8865

[a] = (Stdev(for k^{pfo})/k^{pfo})*k_{2H}; [b] data from the T-dependence of KIE studies (see Table S3)

Six runs together with averaged data	First-order kinetic fit of averaged data
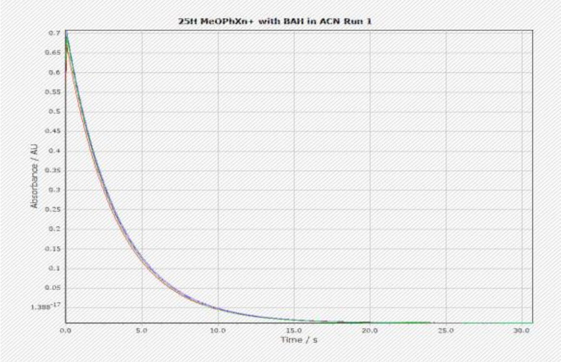	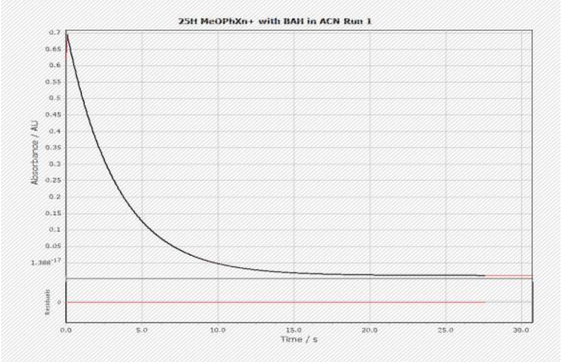
MeOPhXn$^+$	
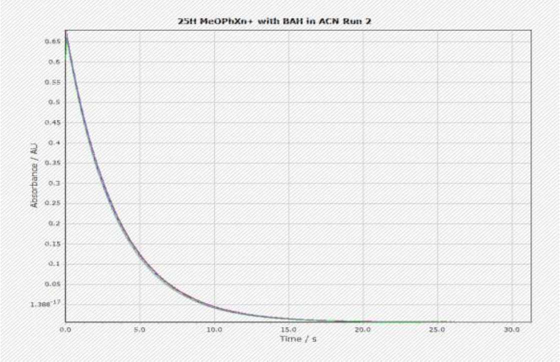	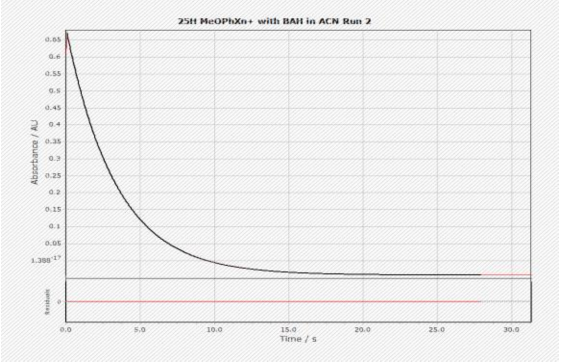
MeOPhXn$^+$	
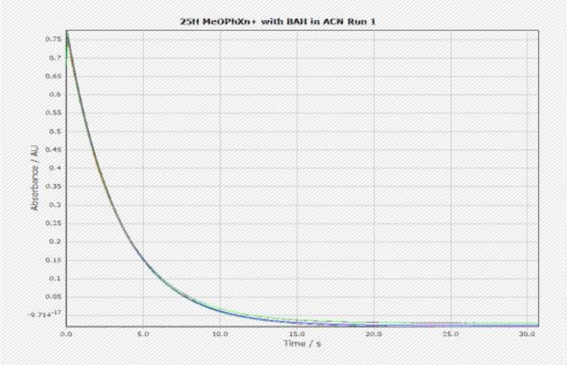	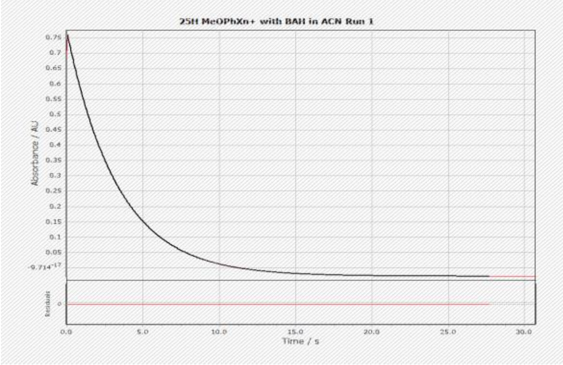
MeOPhXn$^+$	
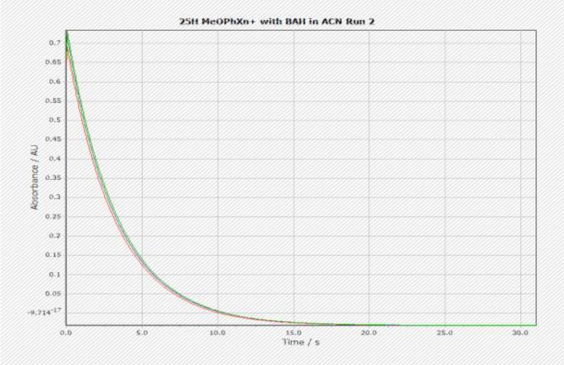	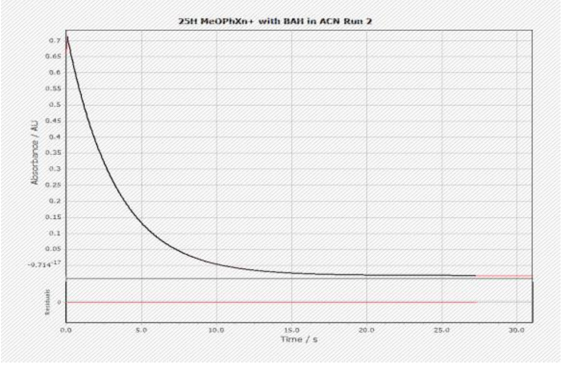
MeOPhXn$^+$	

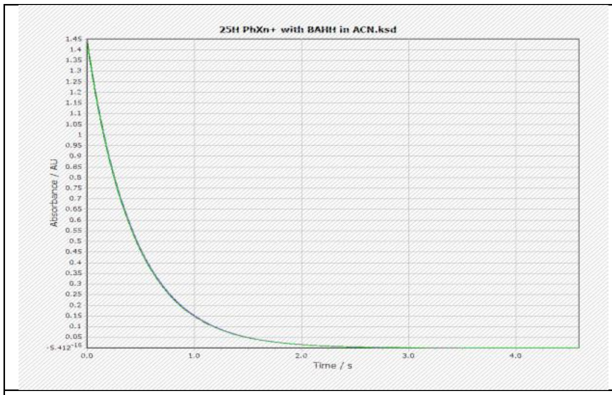

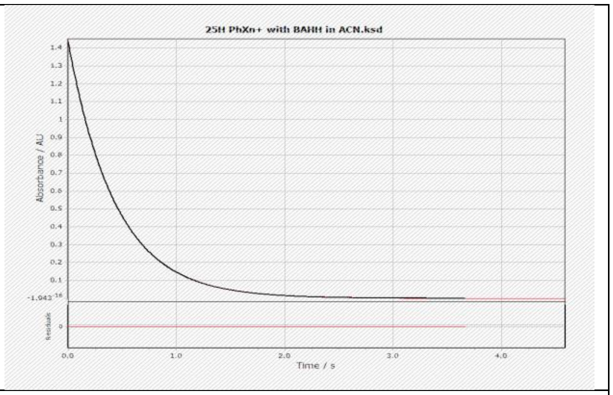

PhXn+

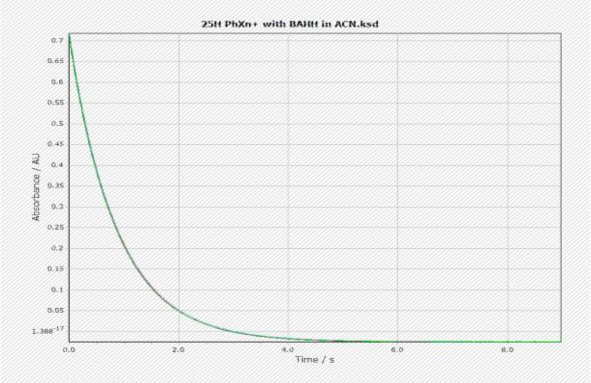

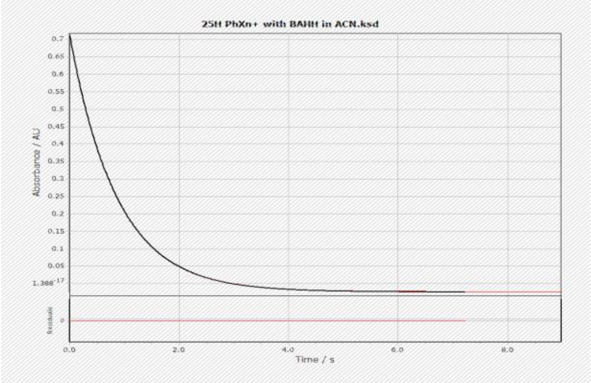

PhXn+

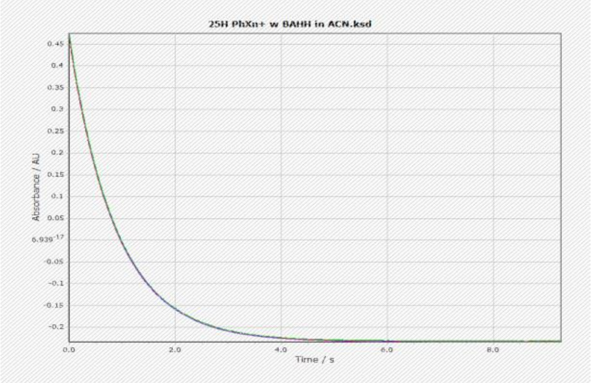

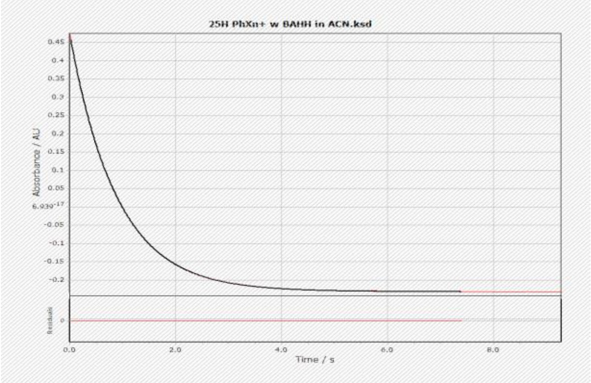

PhXn+

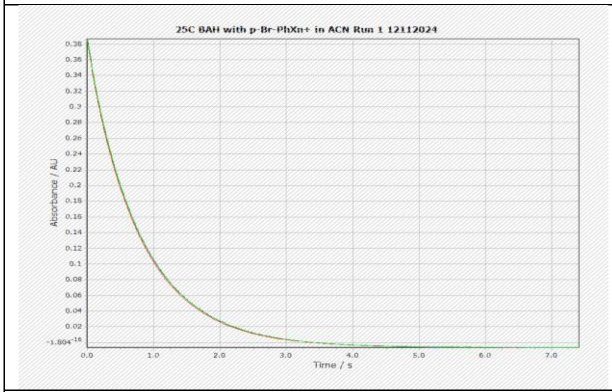

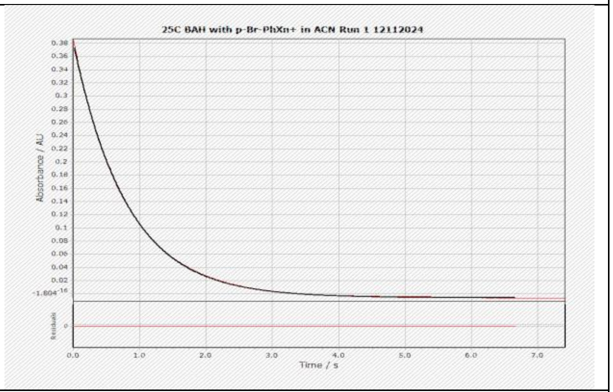

BrPhXn+

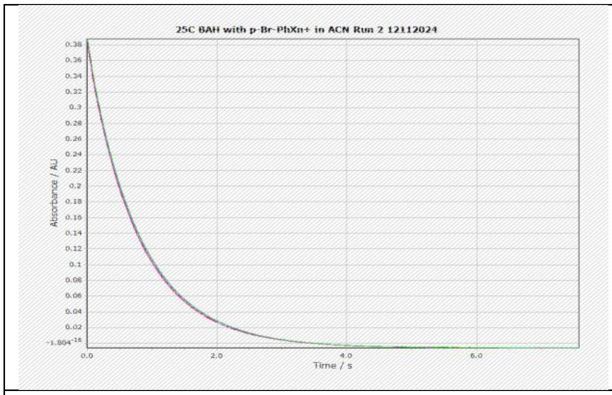

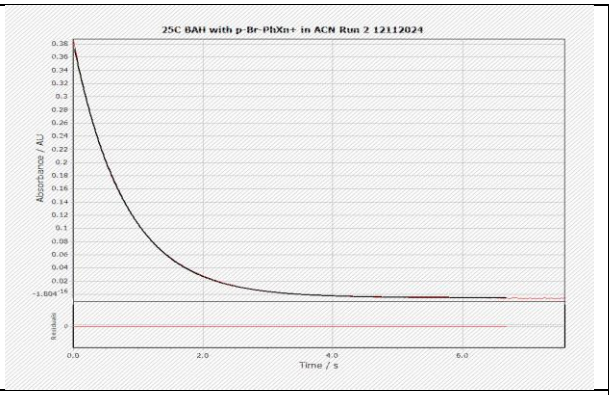

BrPhXn^{+}

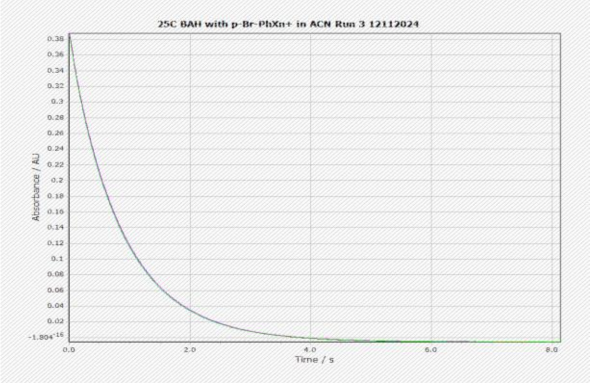

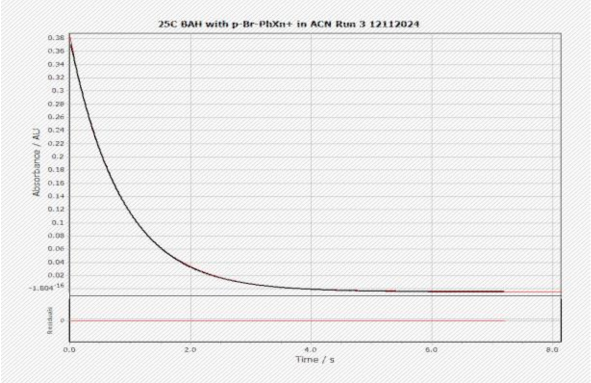

BrPhXn^{+}

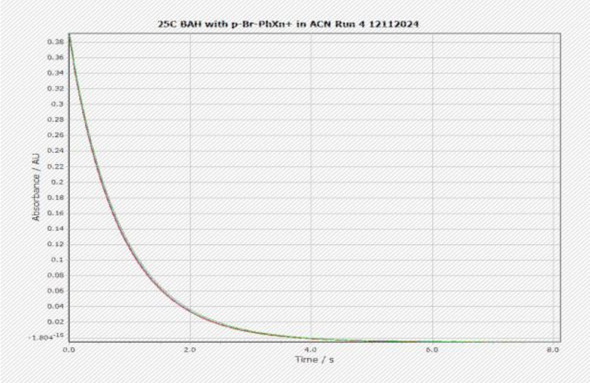

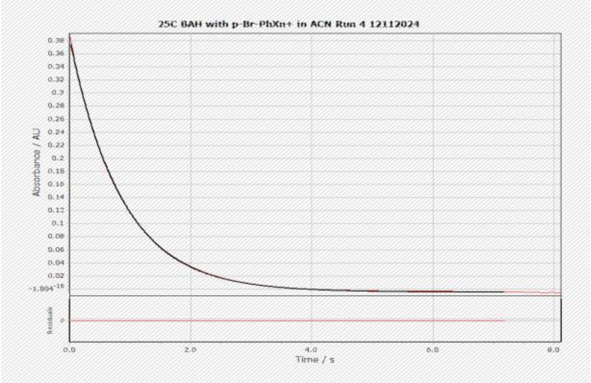

BrPhXn^{+}

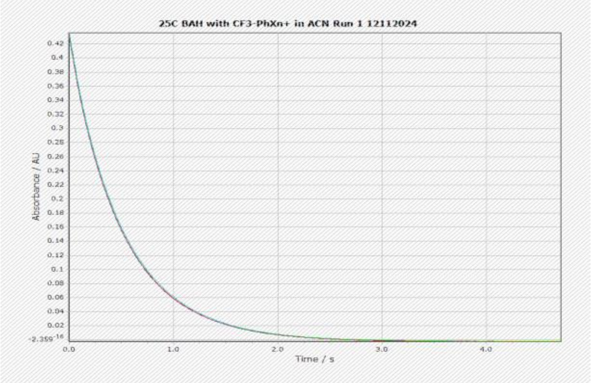

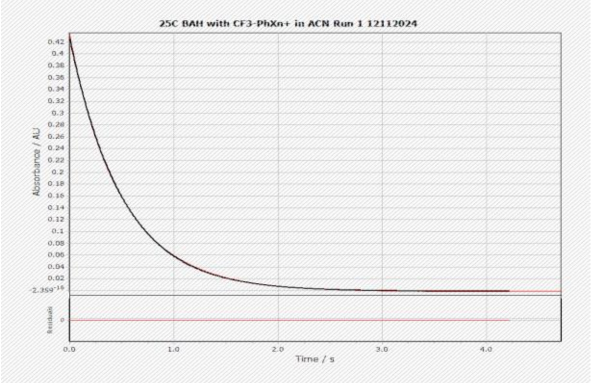

CF_3PhXn^{+}

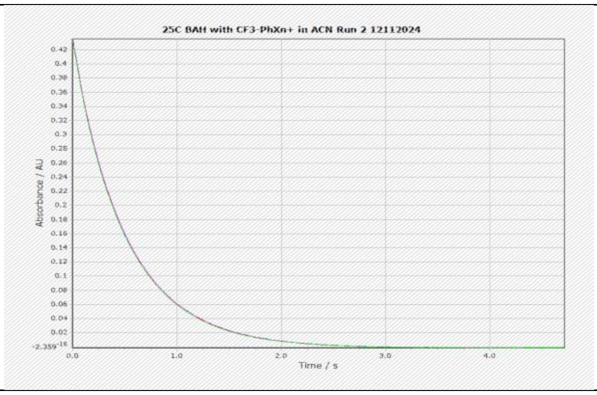

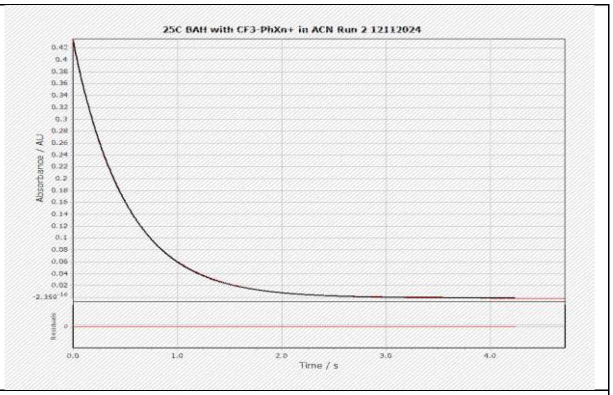

CF_3PhXn^+

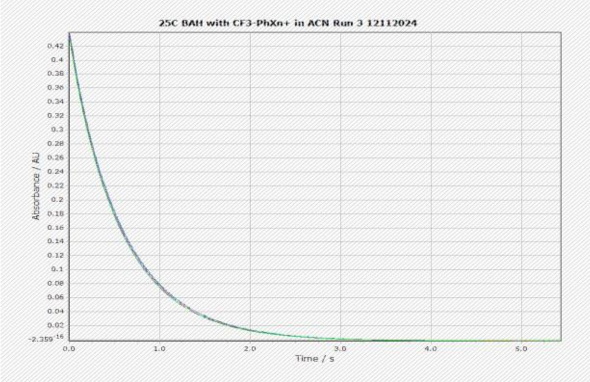

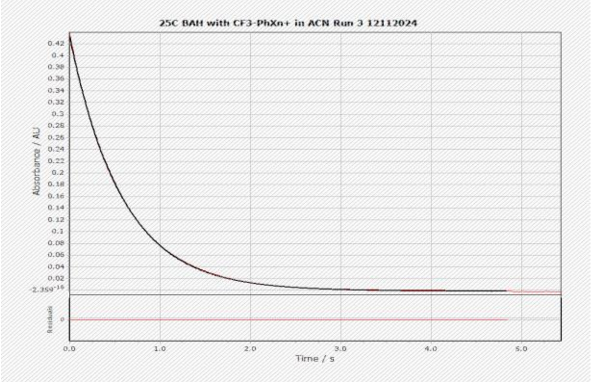

CF_3PhXn^+

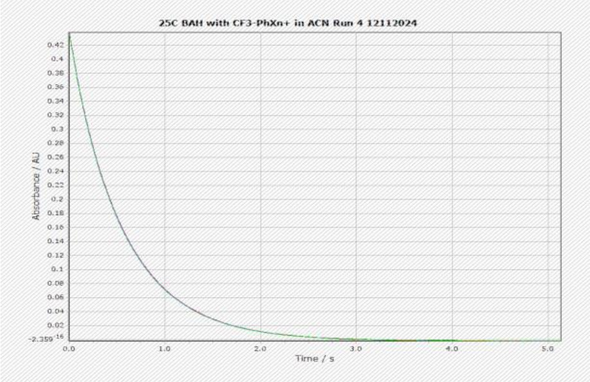

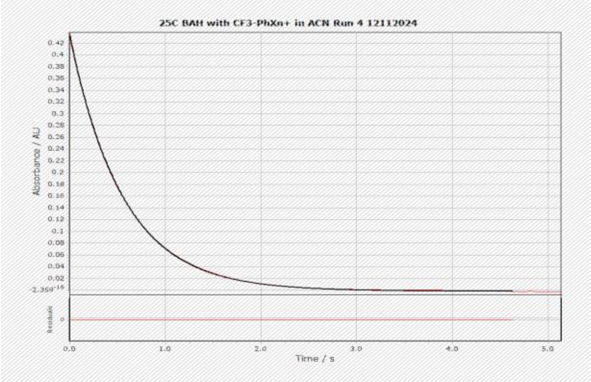

CF_3PhXn^+

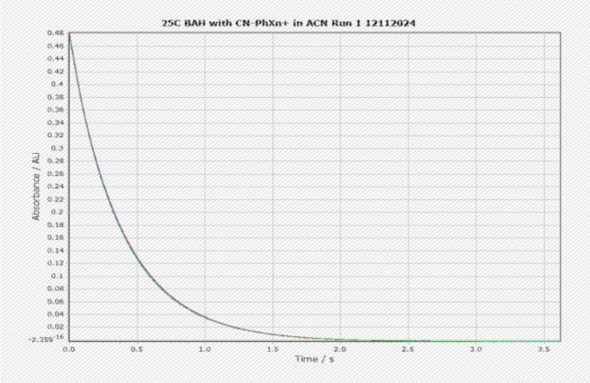

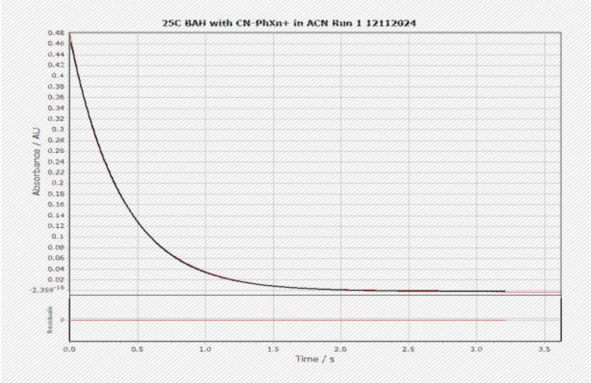

$CNPhXn^+$

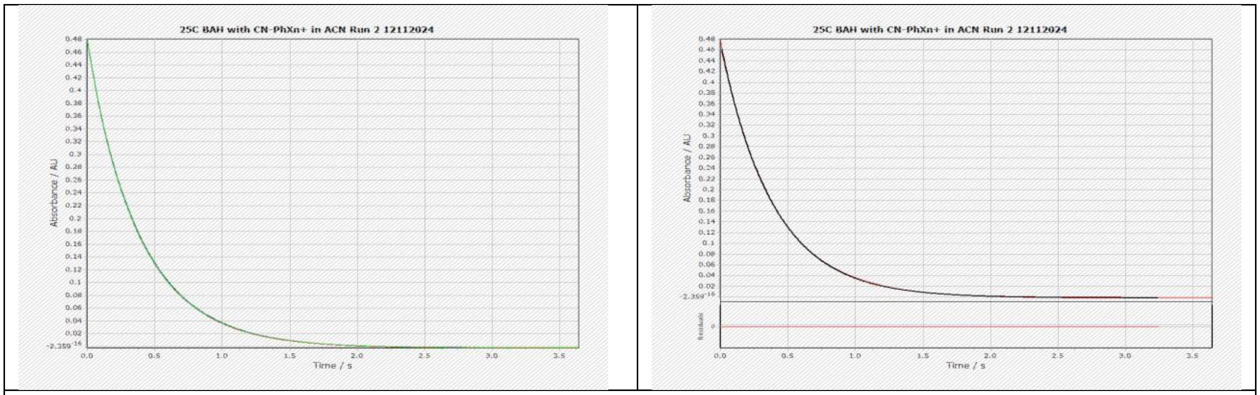

CNPhXn$^+$

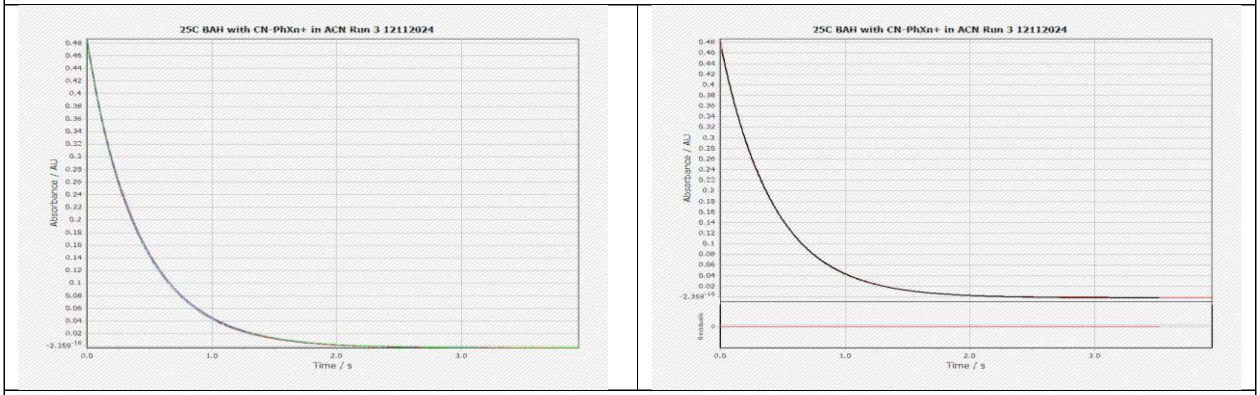

CNPhXn$^+$

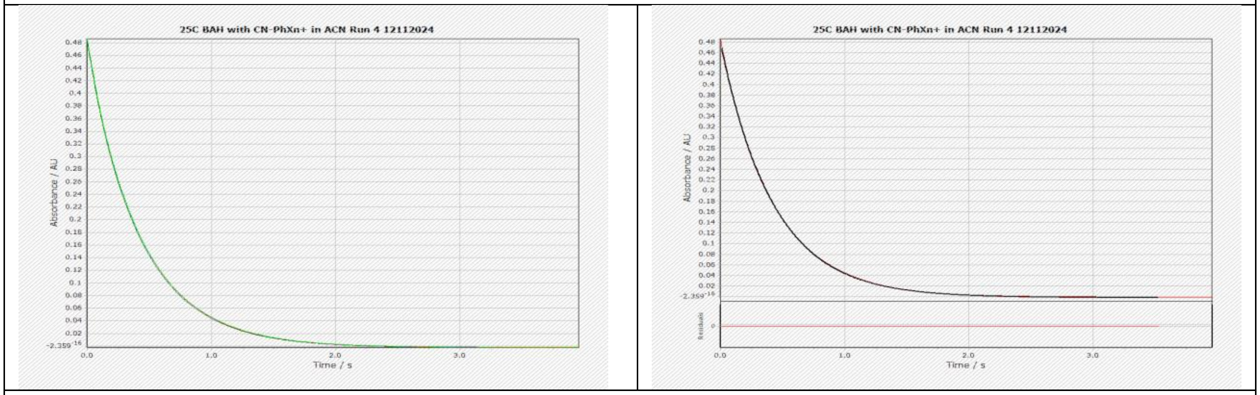

CNPhXn$^+$

Primary kinetic data for the rate constants of *G*PhTXn$^+$ in Table S8

		Pseudo-first-order rate constants k_H^{pfo} (s^{-1})									
Date of Measure-ments	***G*PhXn$^+$**	**Trial H1**	**Trial H2**	**Trial H3**	**Trial H4**	**Trial H5**	**Trial H6**	**Average k_H^{pfo} (s^{-1})**	**Stdev**	**k_{2H} (M^{-1}s^{-1})**	**Stdev [a]**
10/1/2024	MeO	0.4352	0.4324	0.4390	0.4347	0.4359	0.4355	0.4354	0.0022	217.7133	1.0752
10/1/2024	MeO	0.4426	0.4402	0.4363	0.4415	0.4349	0.4362	0.4386	0.0032	219.3100	1.6041
10/1/2024	MeO	0.5930	0.5865	0.5831	0.5801	0.5852	0.5898	0.5863	0.0046	293.1300	2.3081
10/1/2024	MeO	0.5910	0.5927	0.5827	0.5860	0.5901	0.5927	0.5892	0.0040	294.5950	2.0155
10/1/2024	Me	0.5922	0.5912	0.5889	0.5914	0.5885	0.5932	0.5909	0.0018	295.4417	0.9226
10/1/2024	Me	0.5920	0.5927	0.5943	0.5951	0.5986	0.6004	0.5955	0.0033	297.7633	1.6683
10/1/2024	Me	0.5930	0.5865	0.5831	0.5801	0.5852	0.5898	0.5863	0.0046	293.1300	2.3081
10/1/2024	Me	0.4443	0.4444	0.4392	0.4479	0.4461	0.4456	0.4446	0.0030	222.3008	1.4770

9/11/2024	H [b]	0.9663	0.9576	0.9600	0.9728	0.9668	0.9568	0.9634	0.0063	321.1222	2.0901
9/25/2024	H [b]	0.9290	0.9281	0.9237	0.9310	0.9280	0.9386	0.9297	0.0050	309.9106	1.6520
9/27/2024	H [b]	0.9568	0.9639	0.9594	0.9603	0.9710	0.9583	0.9616	0.0052	320.5389	1.7285
10/1/2024	Cl	0.8929	0.8887	0.9028	0.8860	0.9041	0.8933	0.8946	0.0073	447.3217	3.6662
10/1/2024	Cl	0.9074	0.9098	0.9139	0.9072	0.9040	0.9041	0.9077	0.0037	453.8542	1.8732
10/1/2024	Cl	0.8807	0.8758	0.8761	0.8896	0.8852	0.8821	0.8816	0.0053	440.7867	2.6684
10/1/2024	Cl	0.8797	0.8847	0.8928	0.8882	0.8821	0.8864	0.8857	0.0046	442.8325	2.3129
12/11/2024	CF_3	1.1166	1.1174	1.0981	1.1196	1.1304	1.1383	1.1201	0.0137	560.0342	6.8590
12/11/2024	CF_3	1.1719	1.1458	1.1208	1.1411	1.1610	1.1447	1.1475	0.0176	573.7725	8.7803
12/11/2024	CF_3	1.0916	1.0711	1.0745	1.0765	1.0879	1.0970	1.0831	0.0105	541.5317	5.2478
12/11/2024	CF_3	1.1317	1.1063	1.1051	1.1066	1.1137	1.1077	1.1118	0.0102	555.9200	5.1036

[a] = (Stdev(for k^{pfo})/k^{pfo})*k_{2H}; [b] data from the T-dependence of KIE studies (see Table S4)

<table>
<tr><td>Six runs together with averaged data</td><td>First-order kinetic fit of averaged data</td></tr>
<tr><td>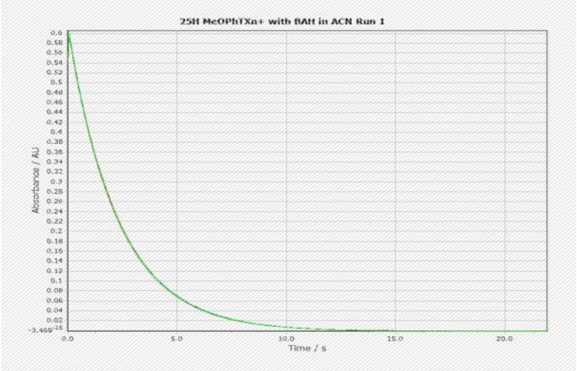
</td><td>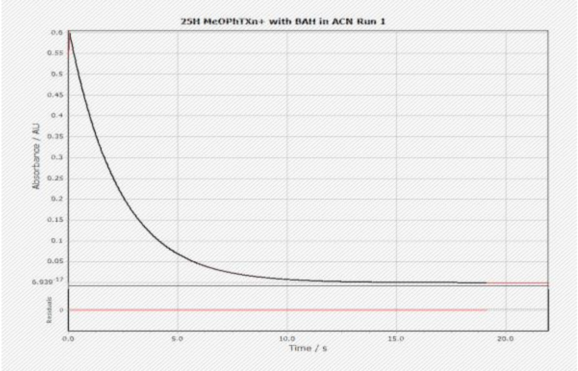
</td></tr>
<tr><td colspan="2">MeOPhTXn+</td></tr>
<tr><td>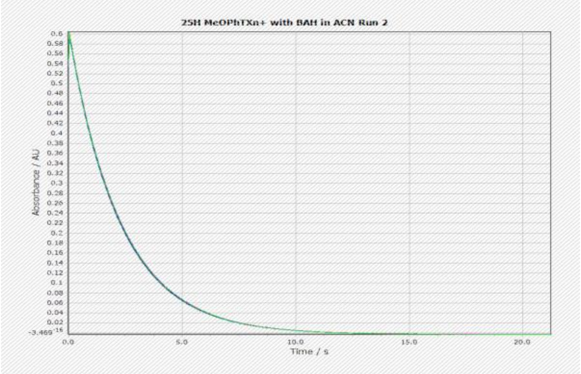
</td><td>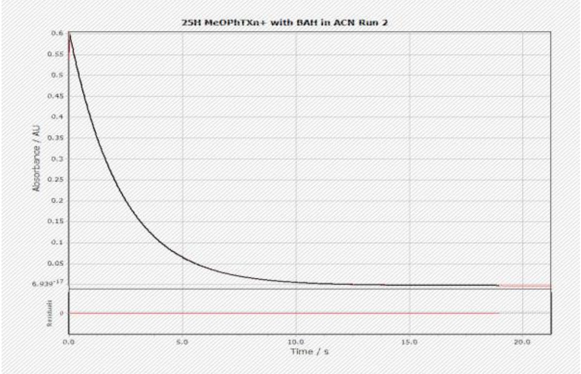
</td></tr>
<tr><td colspan="2">MeOPhTXn+</td></tr>
<tr><td>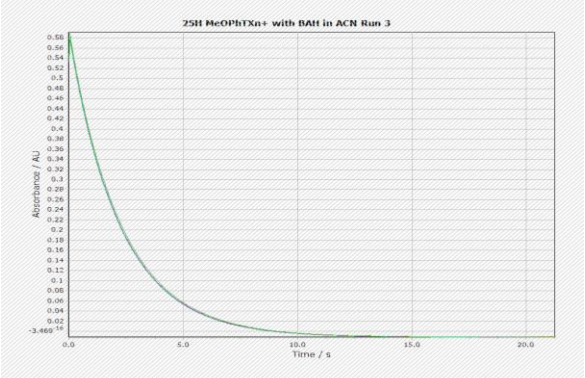
</td><td>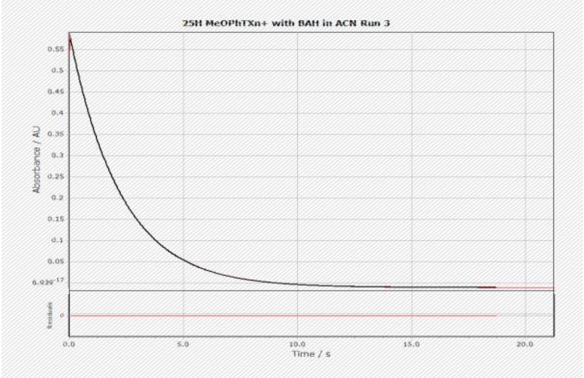
</td></tr>
<tr><td colspan="2">MeOPhTXn+</td></tr>
</table>

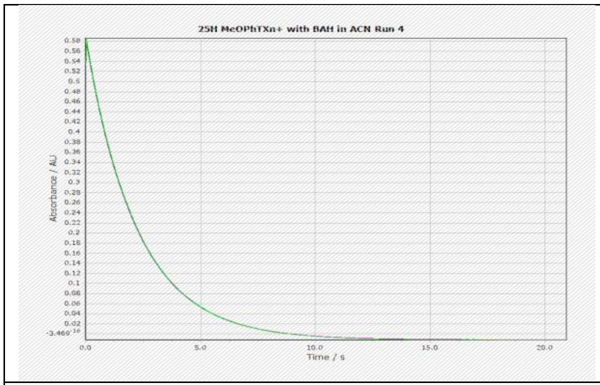

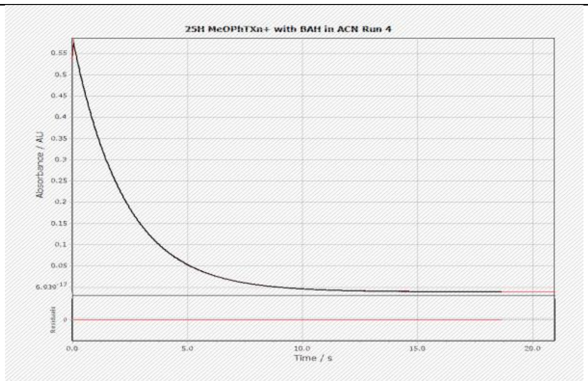

MeOPhTXn^{+}

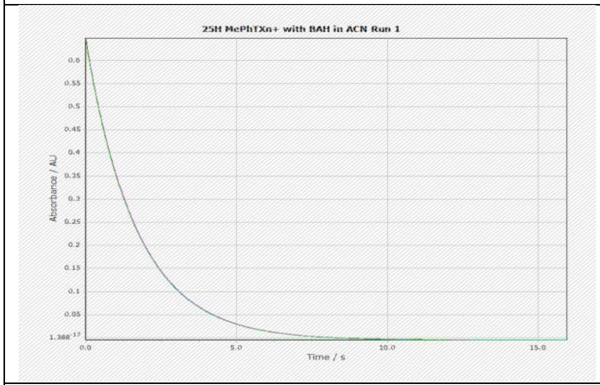

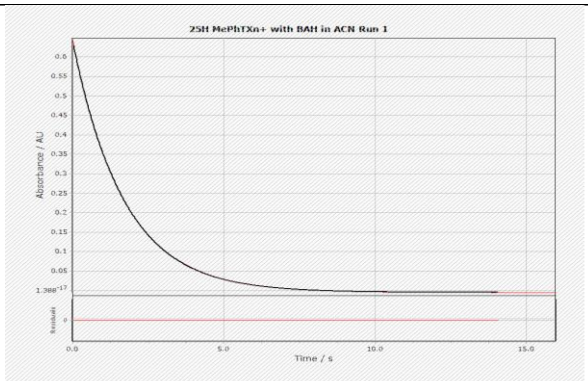

MePhTXn^{+}

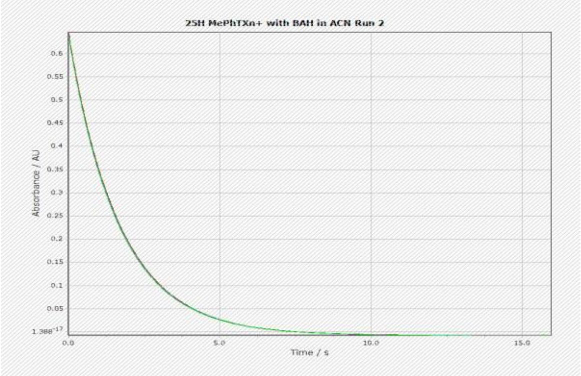

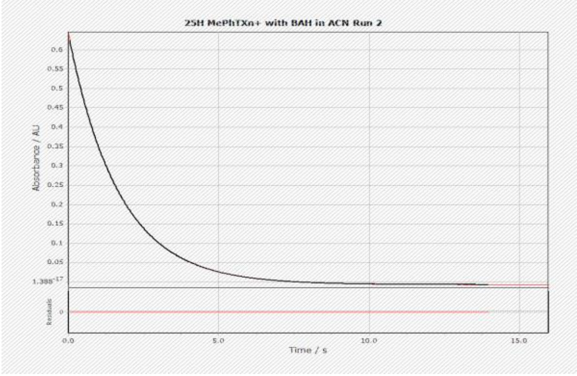

MePhTXn^{+}

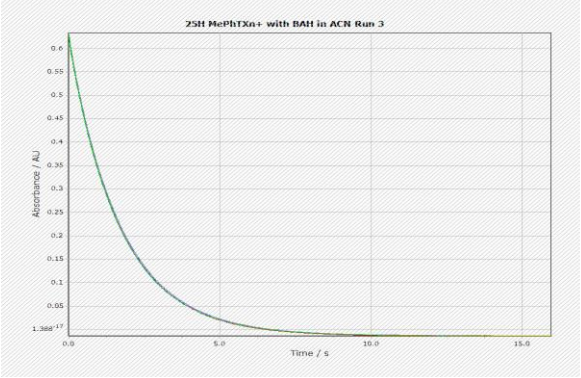

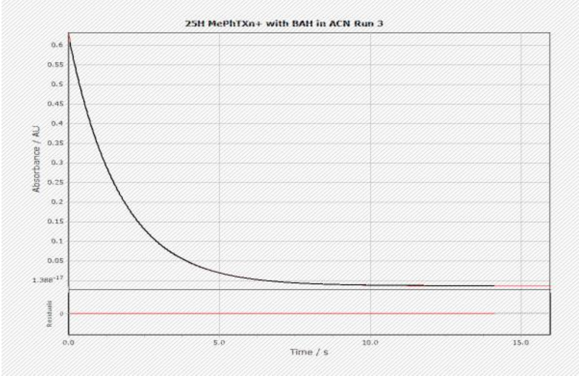

MePhTXn^{+}

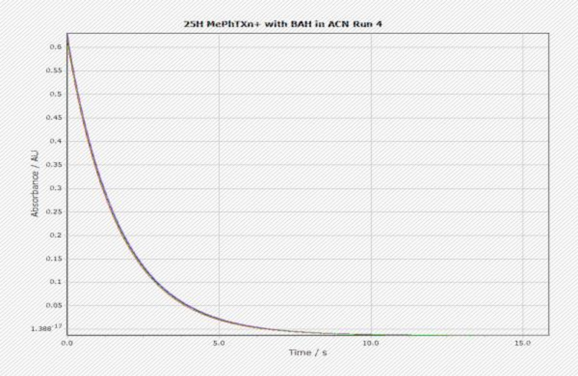

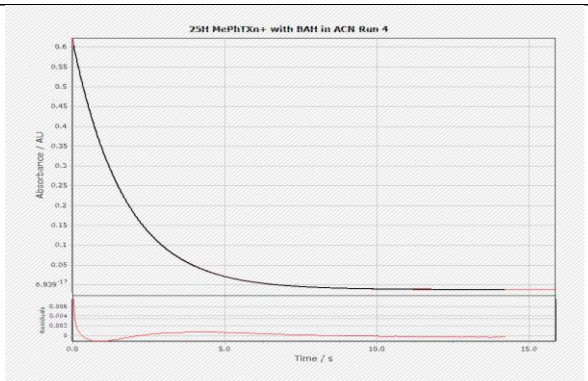

$MePhTXn^{+}$

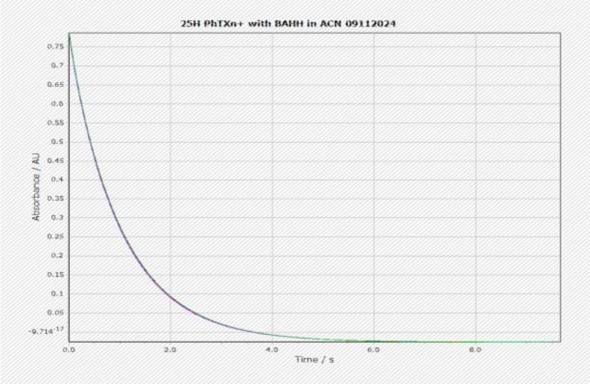

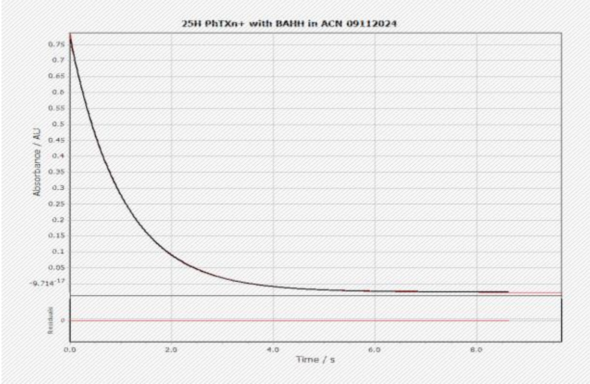

$PhTXn^{+}$

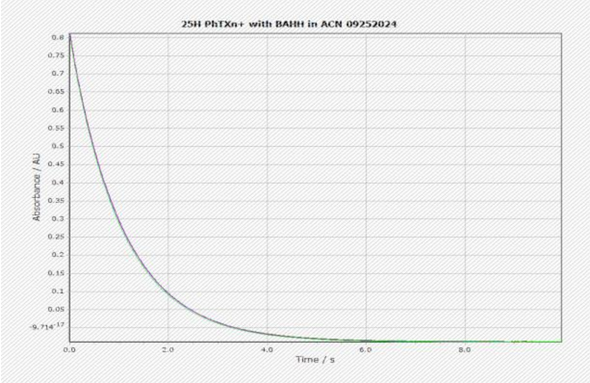

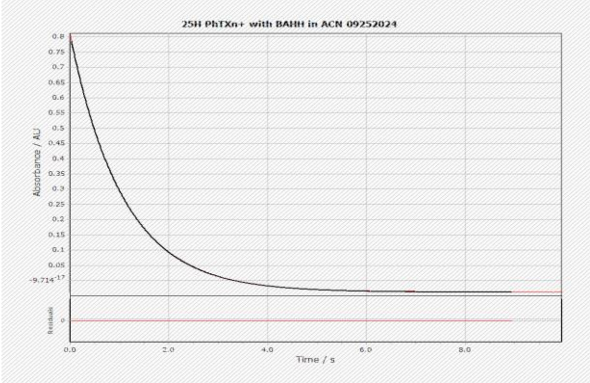

$PhTXn^{+}$

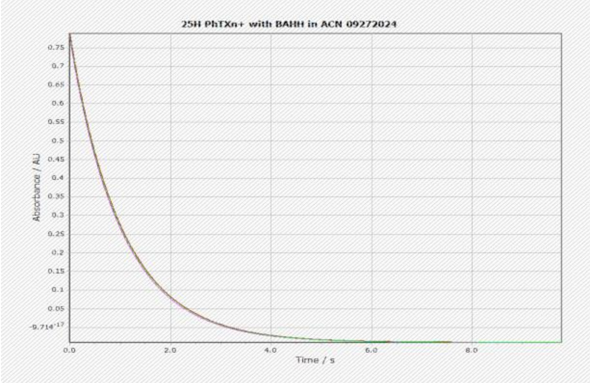

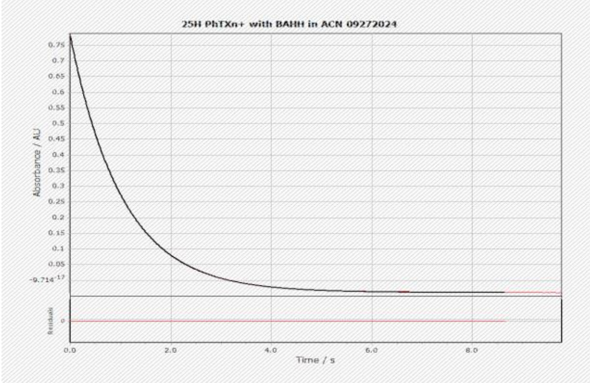

$PhTXn^{+}$

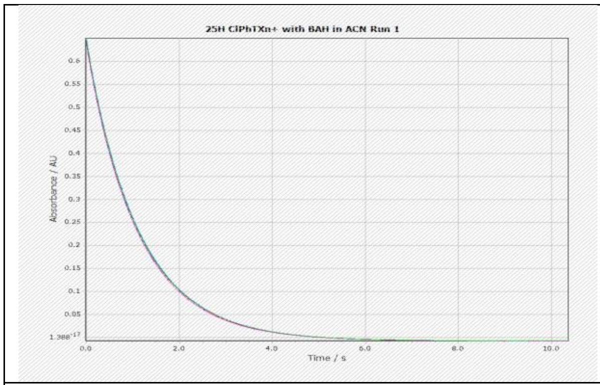

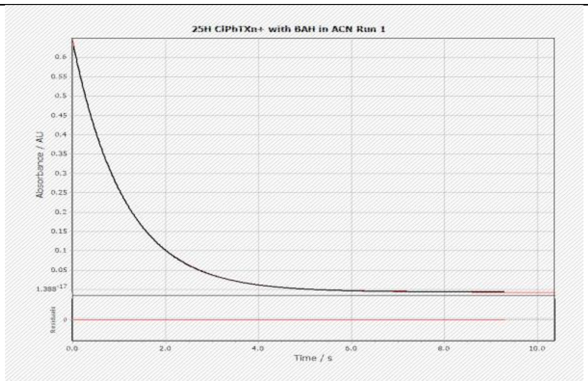

ClPhTXn$^+$

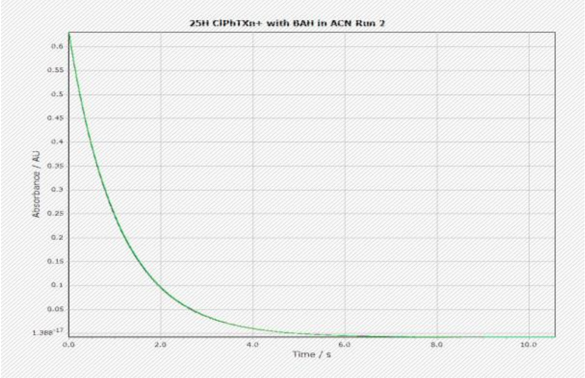

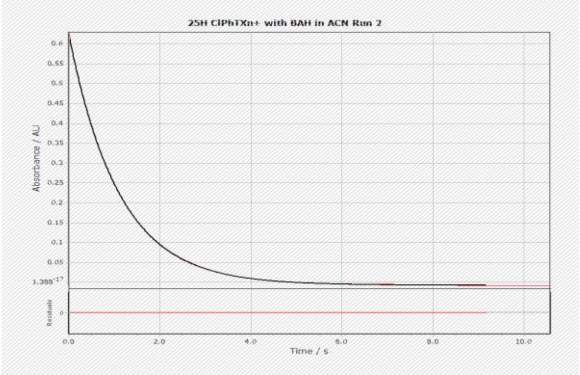

ClPhTXn$^+$

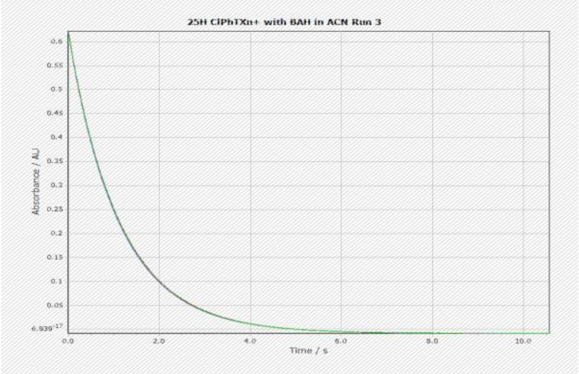

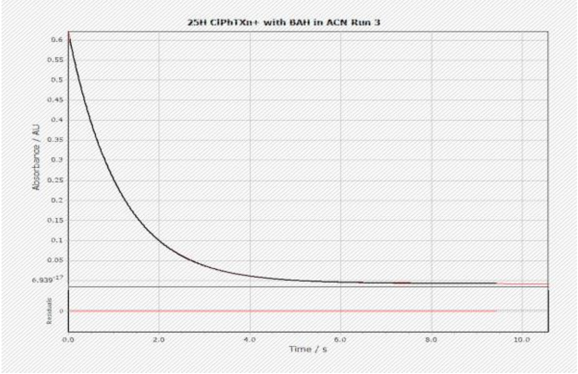

ClPhTXn$^+$

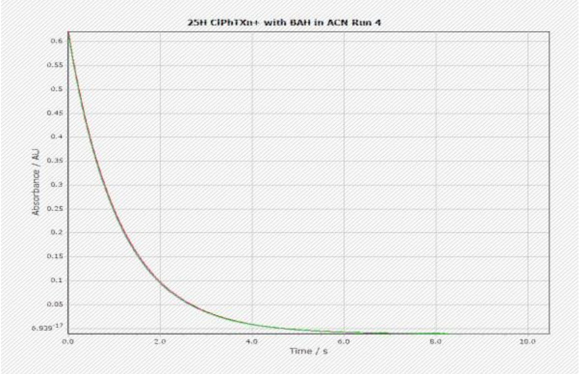

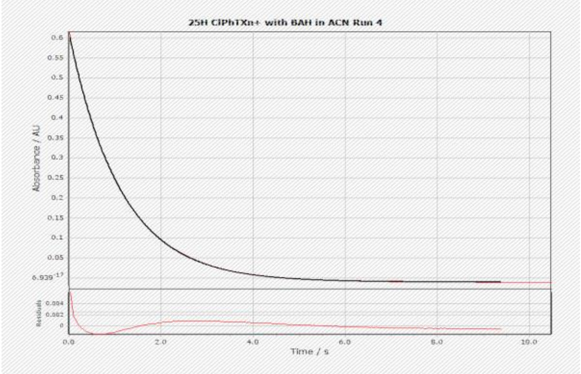

ClPhTXn$^+$

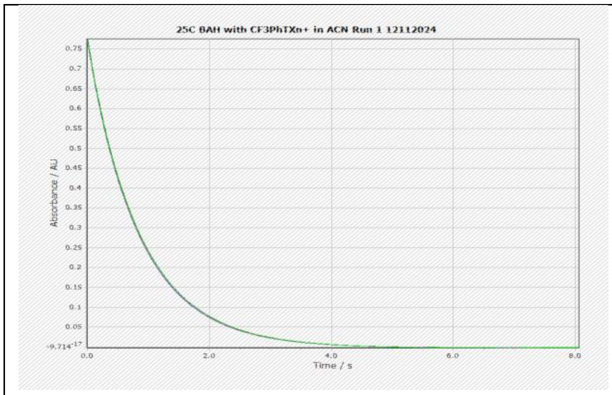

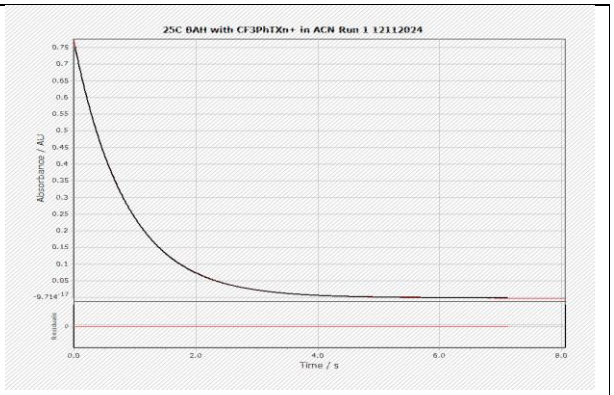

CF$_3$PhTXn$^+$

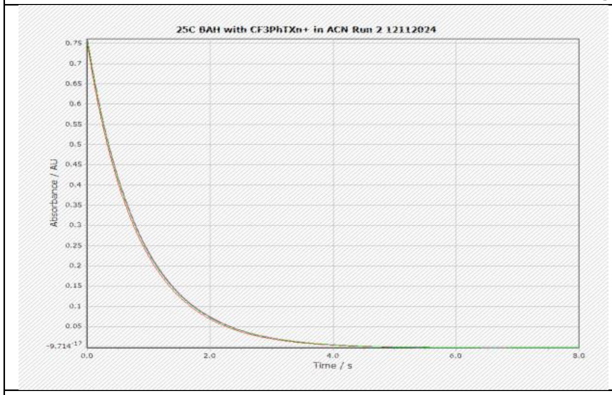

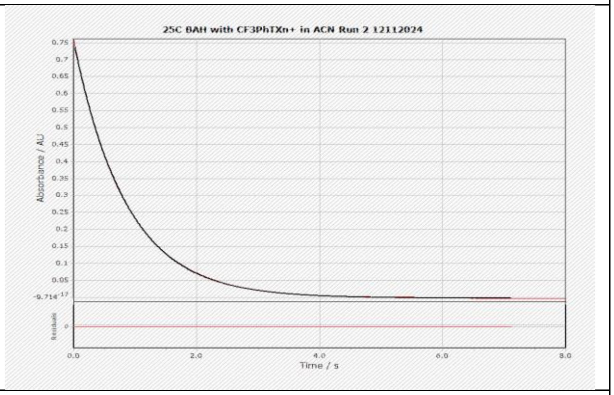

CF$_3$PhTXn$^+$

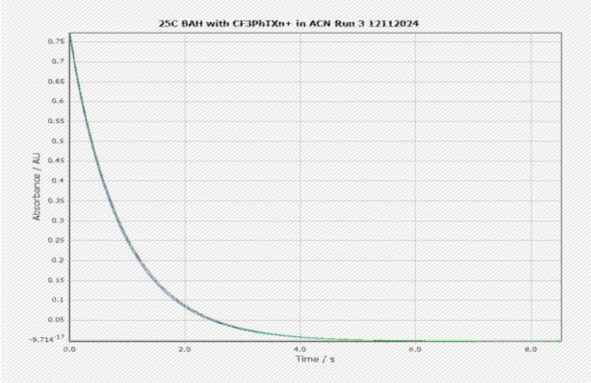

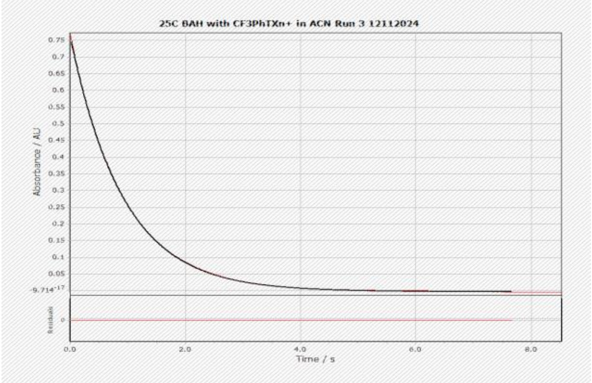

CF$_3$PhTXn$^+$

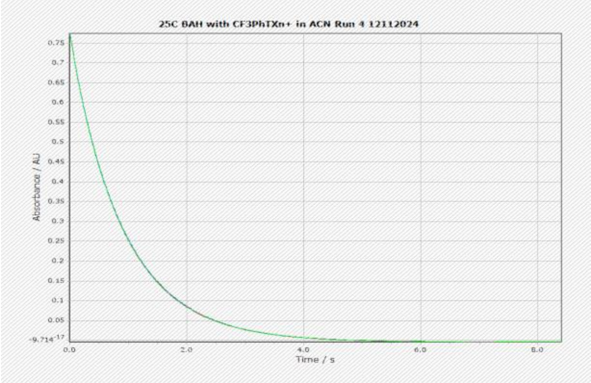

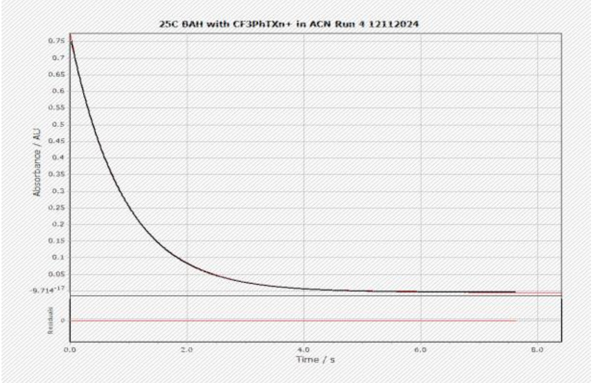

CF$_3$PhTXn$^+$

Primary kinetic data for the rate constants of *G*PhXn$^+$ in Table S9

Date of Measurements	*G*PhXn$^+$	Pseudo-first-order rate constants k_H^{pfo} (s^{-1}) Trial H1	Trial H2	Trial H3	Trial H4	Trial H5	Trial H6	Average k_H^{pfo} (s^{-1})	Stdev	k_{2H} ($M^{-1}s^{-1}$)	Stdev [a]
2/27/2024	MeO	9.33E+02	9.76E+02	9.35E+02				9.48E+02	2.41E+01	1.52E+06	3.87E+04
2/27/2024	MeO	9.74E+02	9.96E+02	1.11E+03				1.03E+03	7.14E+01	1.65E+06	1.15E+05
2/27/2024	MeO	9.50E+02	9.93E+02	1.11E+03				1.02E+03	7.99E+01	1.63E+06	1.28E+05
12/02/2023	Me_2N	29.2573	29.0916	29.3154	29.1293	29.1783	29.2768	29.2081	0.0887	5.99E+04	1.82E+02
12/14/2024	Me_2N	26.2928	26.7320	26.2588	26.6498	26.1589	26.3694	26.4103	0.2291	5.42E+04	4.70E+02
12/18/2024	Me_2N	26.7189	26.5350	26.5384	26.6582	26.348	26.9288	26.6212	0.1972	5.46E+04	4.04E+02

[a] = (Stdev(for k^{pfo})/k^{pfo})*k_{2H}

Six runs together with averaged data	First-order kinetic fit of averaged data
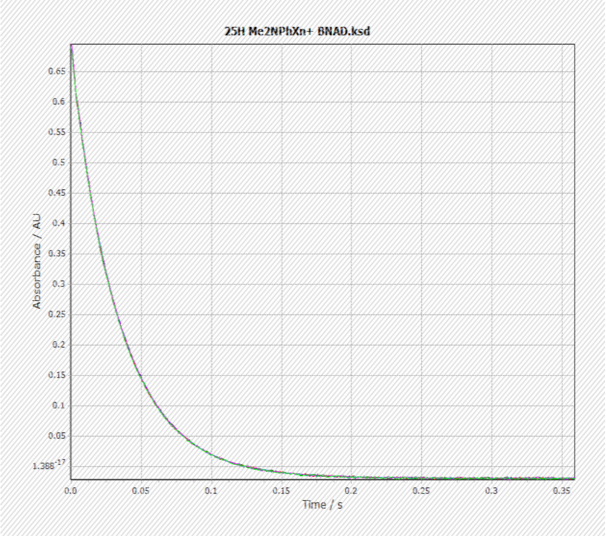	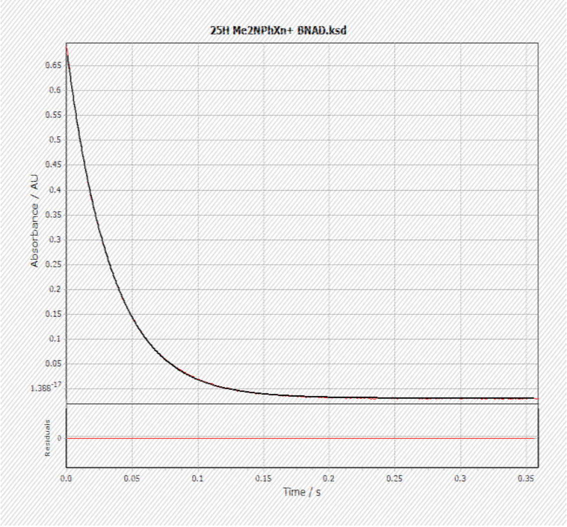

Me_2NPhXn^+

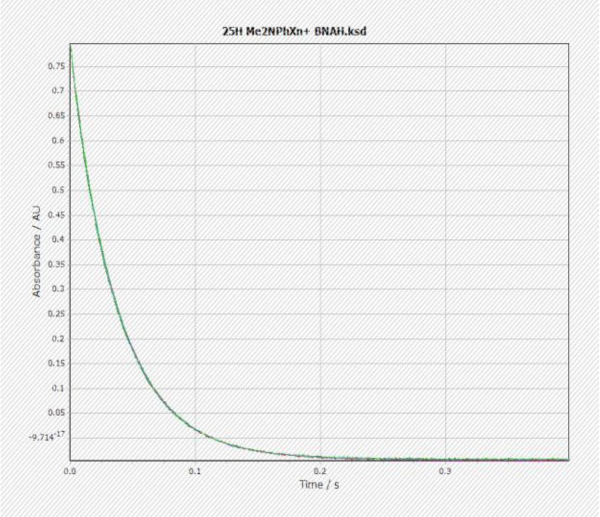	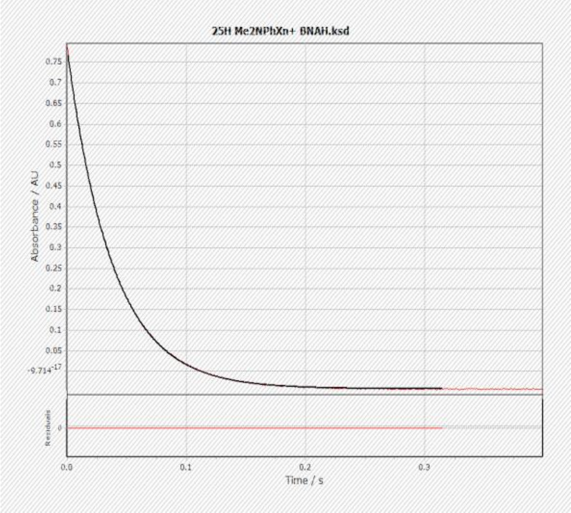

Me_2NPhXn^+

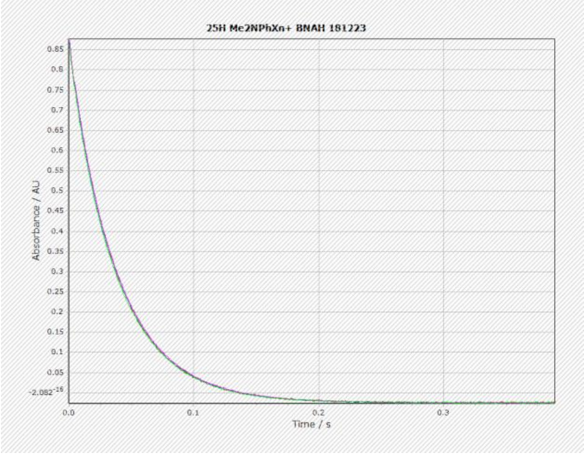

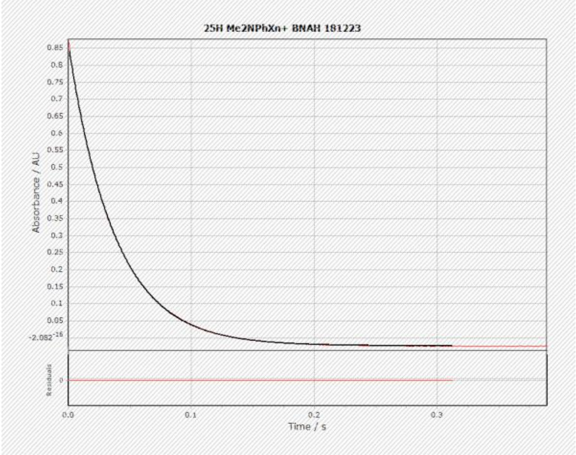

Me_2NPhXn^+

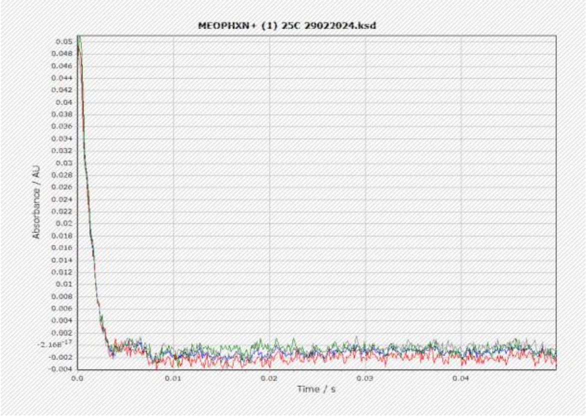

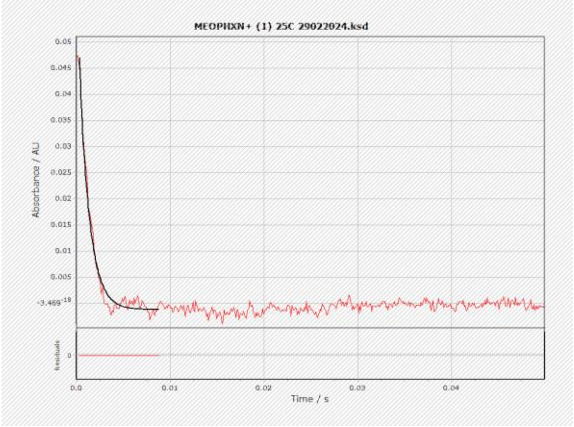

$MeOPhXn^+$

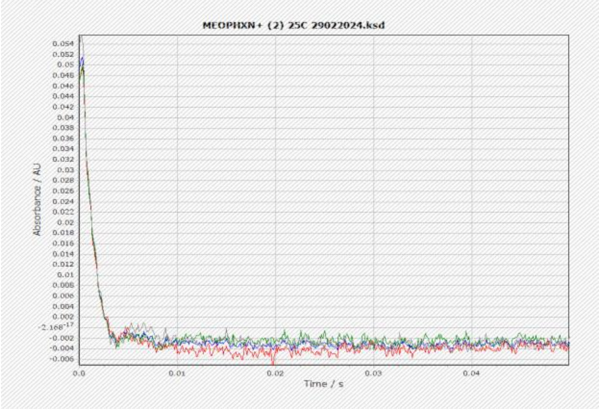

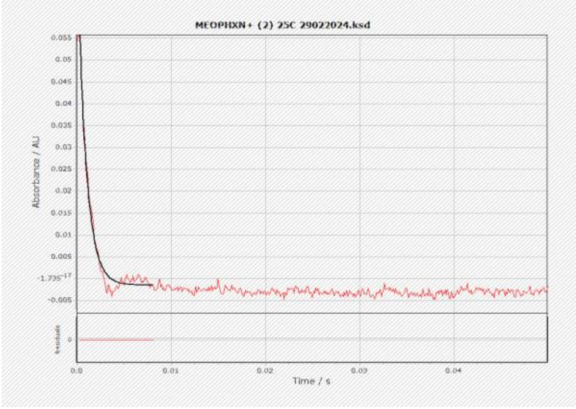

$MeOPhXn^+$

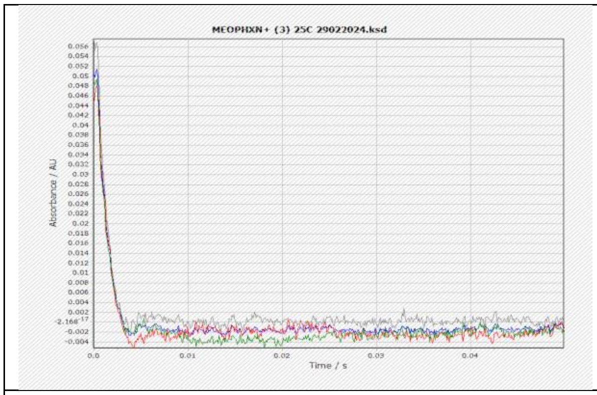

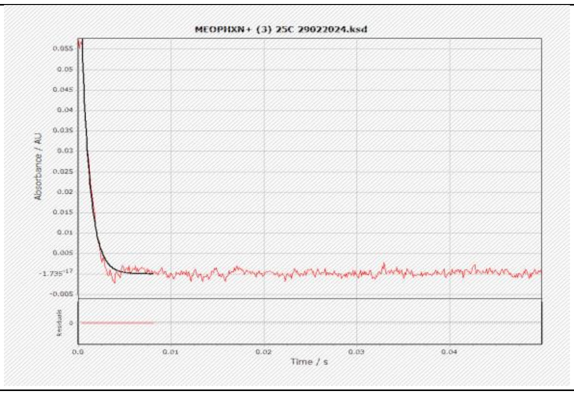

MeOPhXn$^+$

Primary kinetic data for the rate constants of *G*PhMA$^+$ in Table S9

Date of Measure-ments	*G*PhMA$^+$	Trial H1	Trial H2	Trial H3	Trial H4	Trial H5	Trial H6	Average k_H^{pfo} (s^{-1})	Stdev	k_{2H} (M^{-1}s^{-1})	Stdev [a]
1/26/2024	Me_2N	0.00496	0.00500	0.00505				0.00500	0.00004	0.83356	0.00742
2/6/2024	Me_2N	0.00481	0.00484	0.00484				0.00483	0.00002	0.80517	0.00293
3/5/2024	Me_2N	0.00532	0.00524	0.00517				0.00524	0.00007	0.87356	0.01233
2/20/2024	MeO	0.01317	0.01283	0.01321	0.01339	0.01379	0.01422	0.01344	0.00050	2.23917	0.08270
2/22/2024	MeO	0.01476	0.01498	0.01492	0.01499	0.01485	0.01484	0.01489	0.00009	2.48167	0.01491
2/20/2024	MeO	0.01613	0.01586	0.01546	0.01552	0.01530	0.01530	0.01560	0.00033	2.59917	0.05550
2/20/2024	Me	0.01617	0.01668	0.01664	0.01698	0.01739	0.01768	0.01692	0.00055	2.82056	0.09130
2/22/2024	Me	0.01721	0.01679	0.01672	0.01657	0.01655	0.01275	0.01610	0.00166	2.68306	0.27627
2/20/2024	Me	0.01765	0.01721	0.01715	0.01697	0.01700	0.01673	0.01712	0.00031	2.85306	0.05158
2/20/2024	H	0.01661	0.01735	0.01746	0.01745	0.01794	0.01781	0.01744	0.00047	2.90611	0.07761
2/22/2024	H	0.01903	0.01887	0.01864	0.01847	0.01856	0.01820	0.01863	0.00029	3.10472	0.04905
2/20/2024	H	0.01909	0.01921	0.01906	0.01874	0.01857	0.01854	0.01887	0.00029	3.14472	0.04807
2/20/2024	Br	0.03615	0.03858	0.03817	0.03807	0.03757	0.03708	0.03760	0.00088	6.26722	0.14682
2/22/2024	Br	0.03298	0.03257	0.03228	0.03219	0.03218	0.03167	0.03231	0.00044	5.38528	0.07296
2/20/2024	Br	0.03538	0.03417	0.03383	0.03332	0.03297	0.03304	0.03379	0.00091	5.63083	0.15147
2/20/2024	CF_3	0.04236	0.04428	0.04396	0.04404	0.04386	0.04401	0.04375	0.00070	7.29194	0.11597
2/22/2024	CF_3	0.04102	0.04068	0.04003	0.04018	0.04024	0.04021	0.04039	0.00038	6.73222	0.06278
2/20/2024	CF_3	0.04053	0.04017	0.03966	0.03990	0.03905	0.03952	0.03981	0.00052	6.63417	0.08629

[a] = (Stdev(for k^{pfo})/k^{pfo})*k_{2H}

Six 1.5 half-life kinetic runs together with averaged data (For Me_2N substituted $PhMA^+$, three slow runs each)	First-order kinetic fit of the averaged data
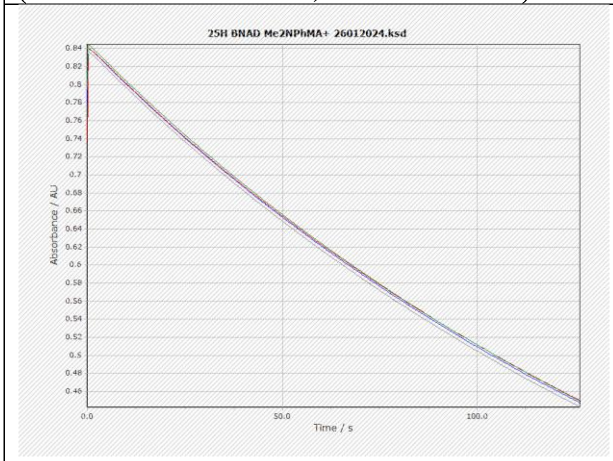	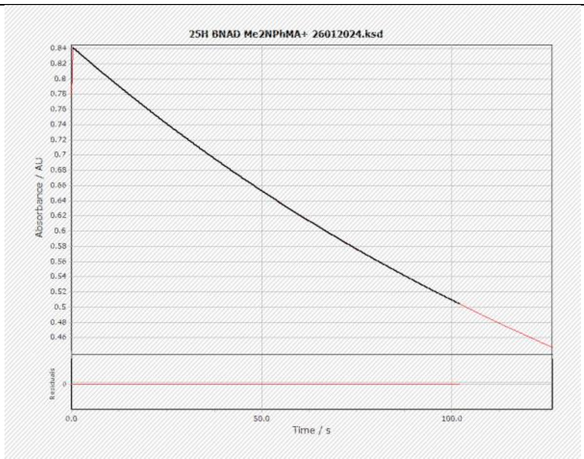
Me_2NPhMA^+	
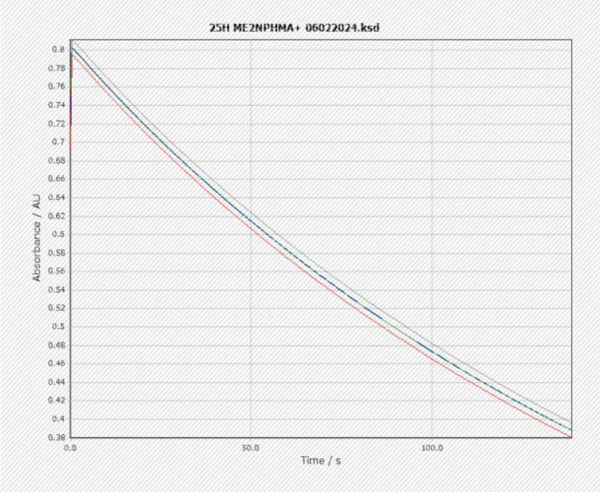	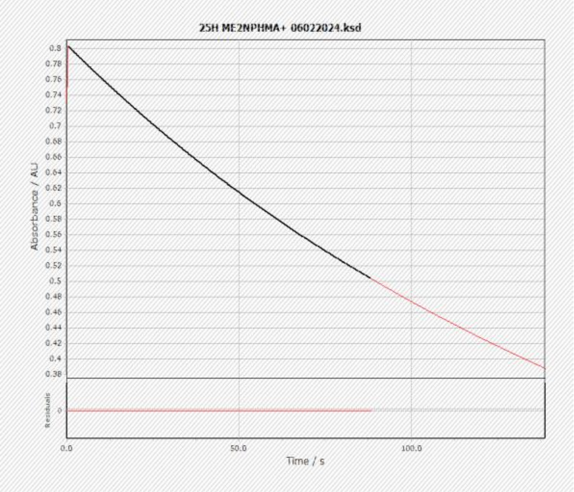
Me_2NPhMA^+	
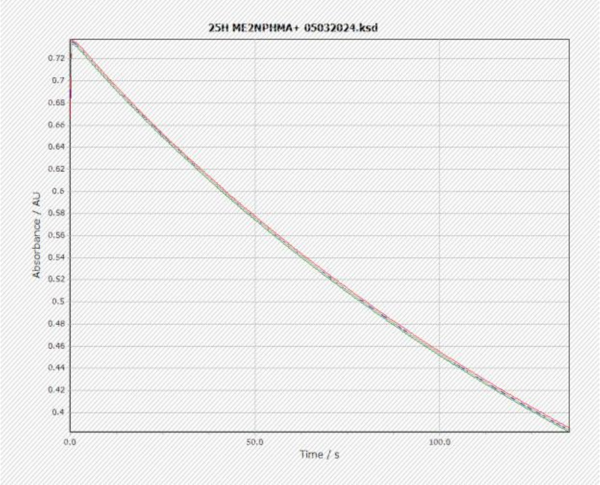	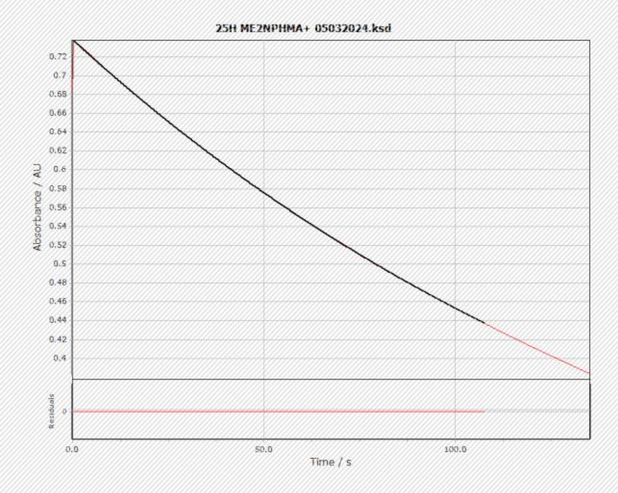
Me_2NPhMA^+	

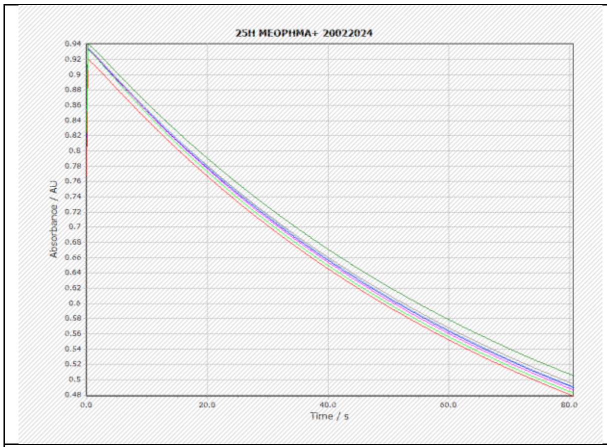

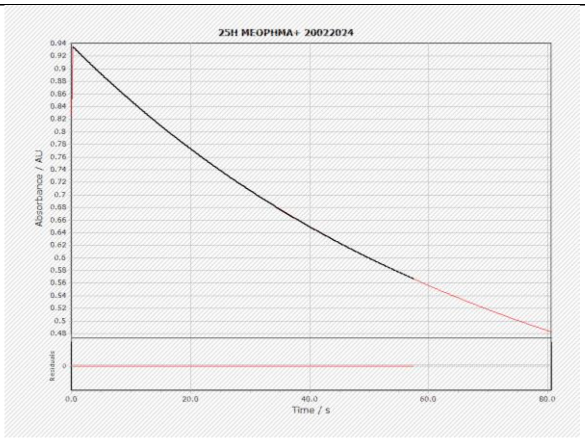

MeOPhMA^+

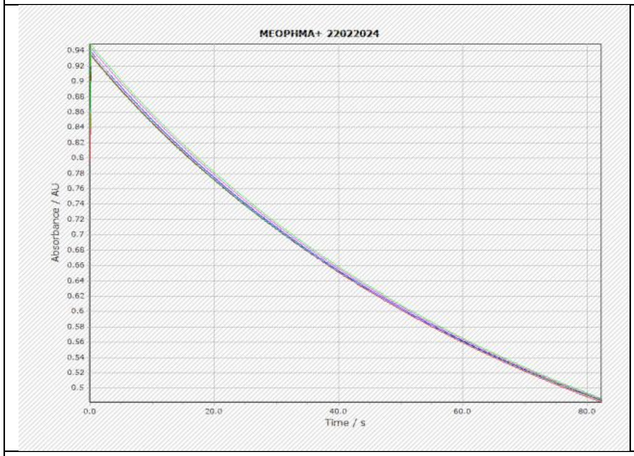

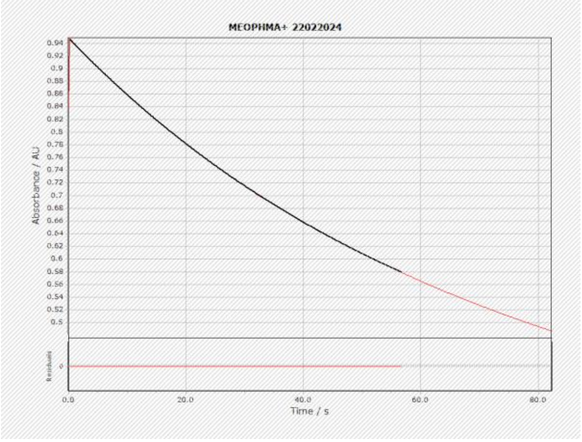

MeOPhMA^+

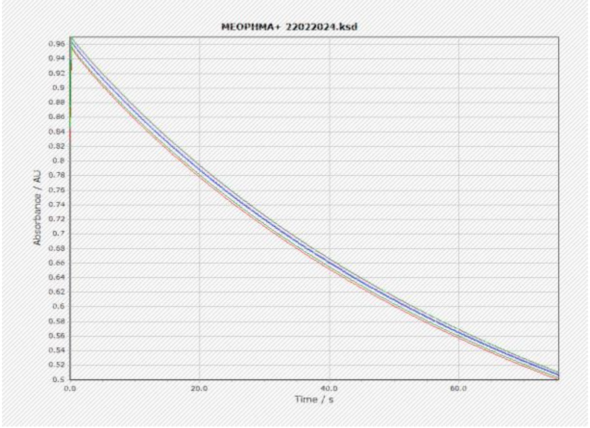

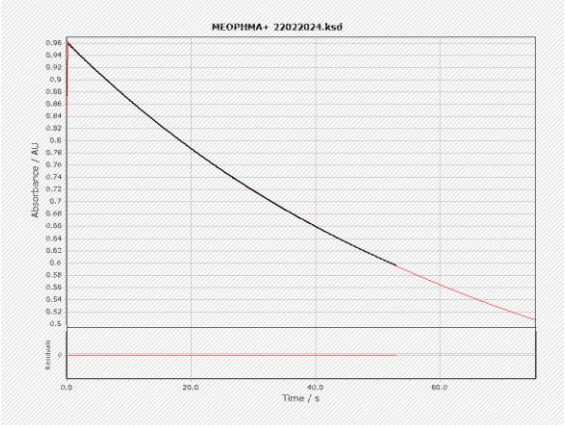

MeOPhMA^+

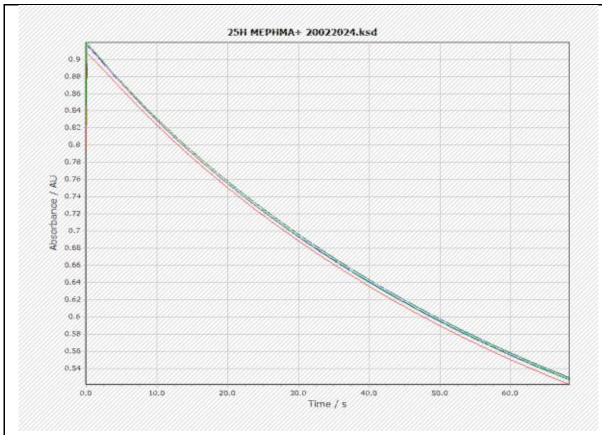

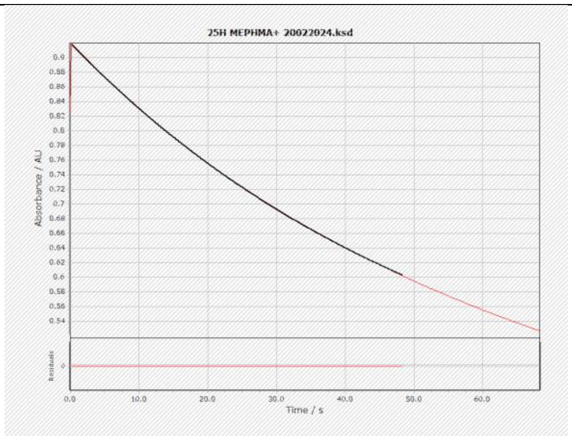

MePhMA$^+$

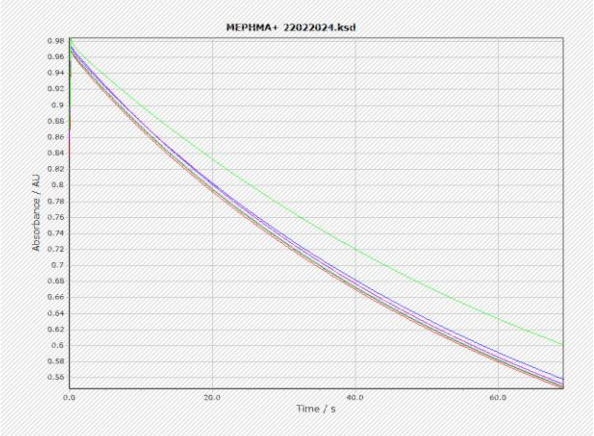

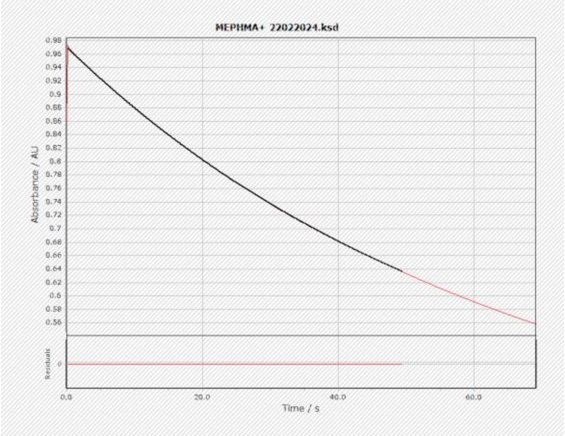

MePhMA$^+$

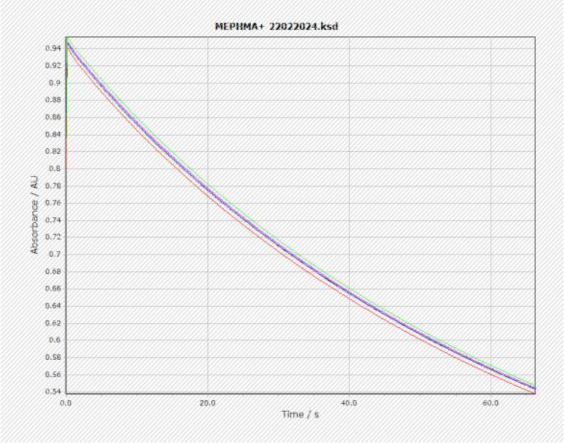

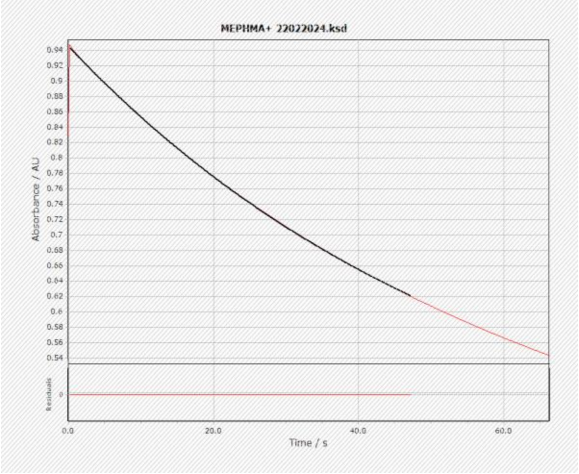

MePhMA$^+$

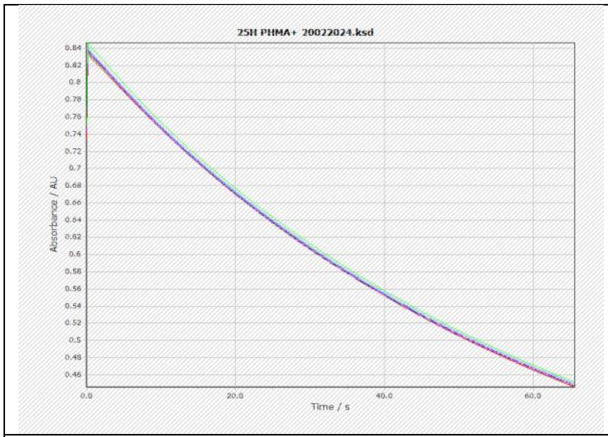

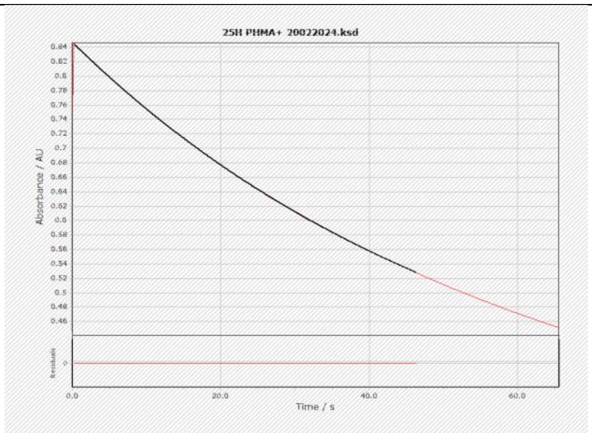

PhMA^{+}

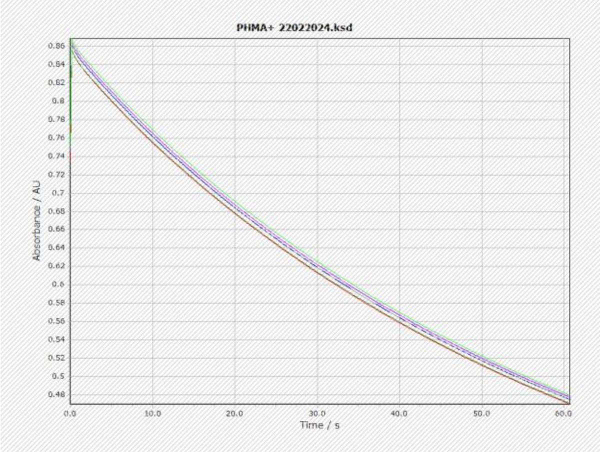

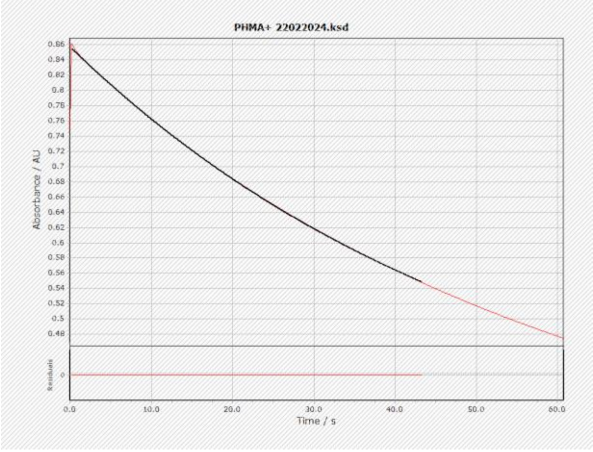

PhMA^{+}

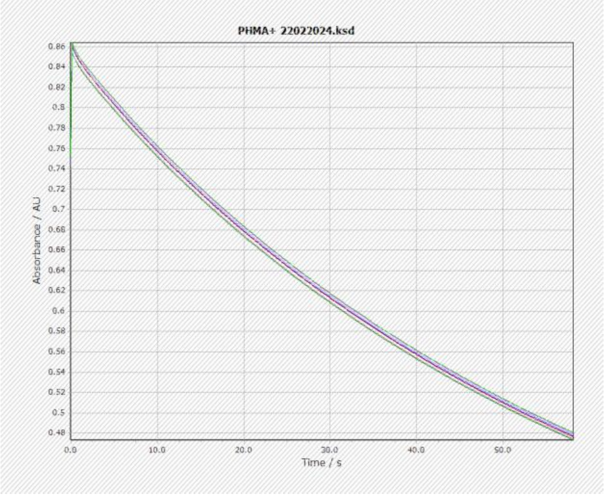

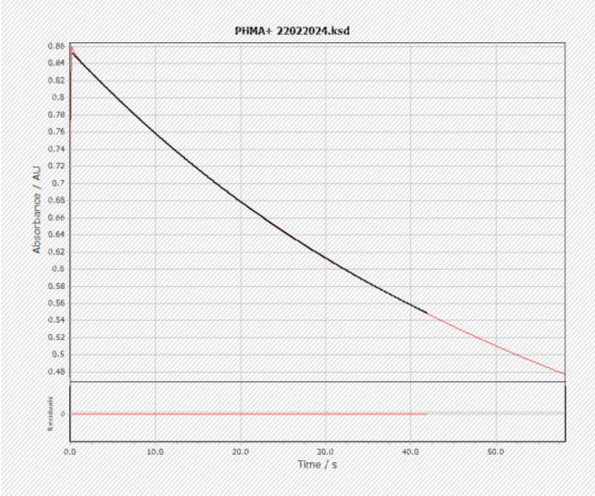

PhMA^{+}

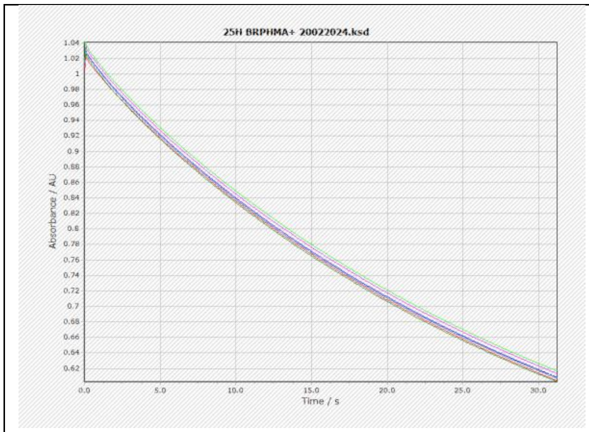

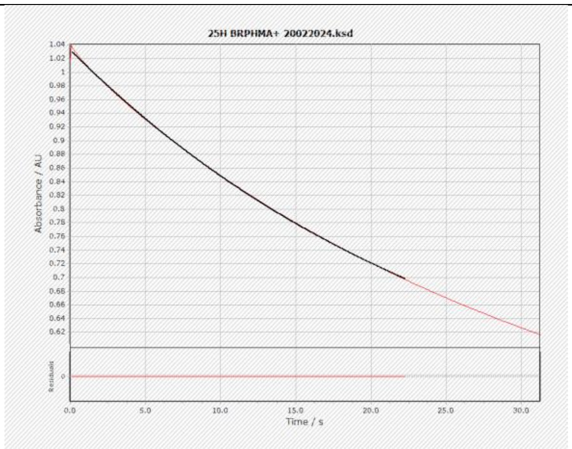

$BrPhMA^+$

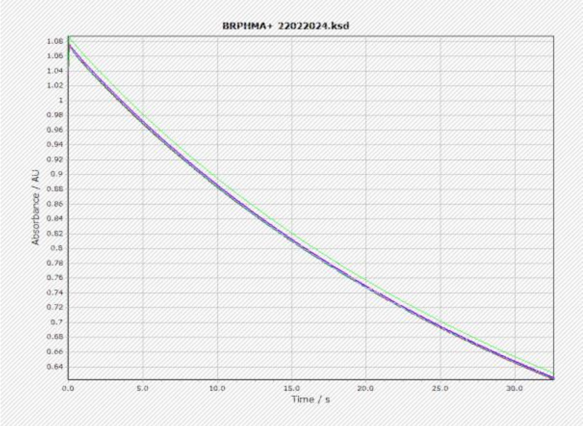

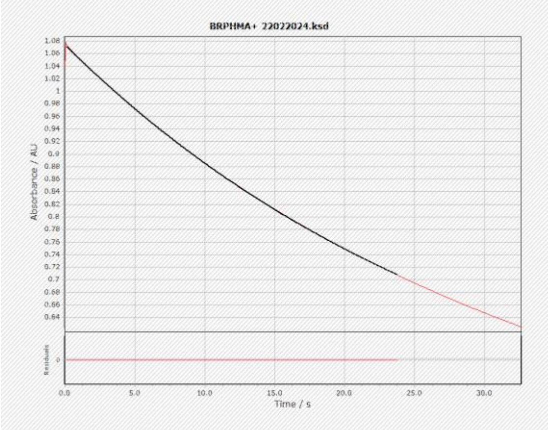

$BrPhMA^+$

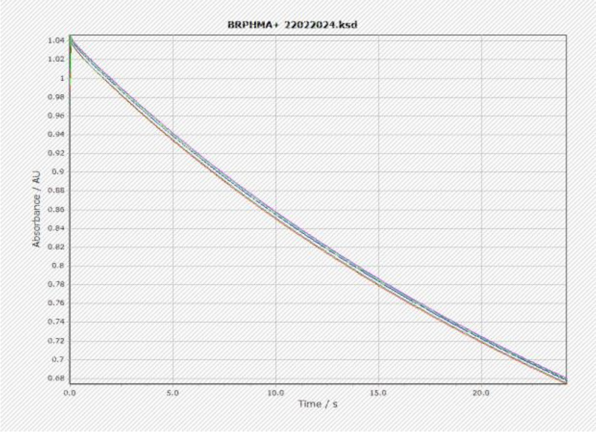

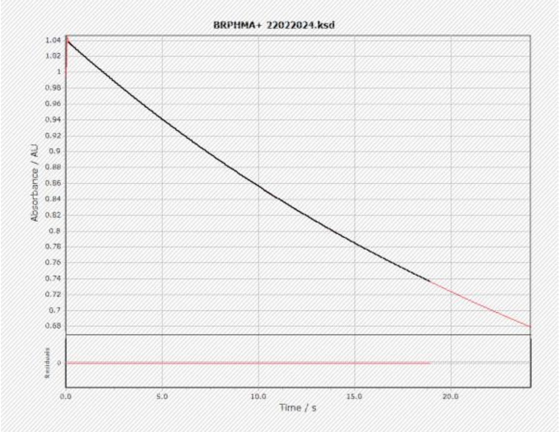

$BrPhMA^+$

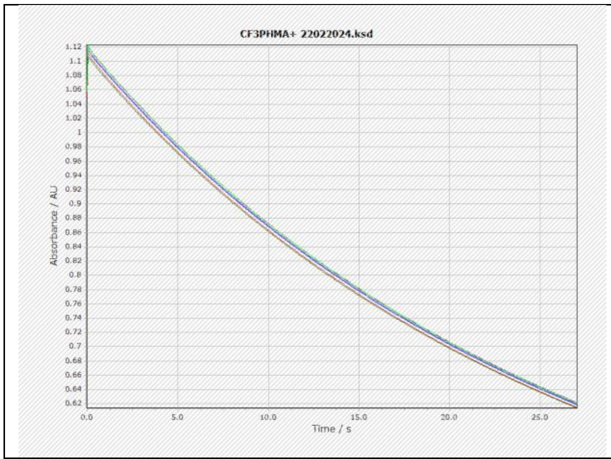

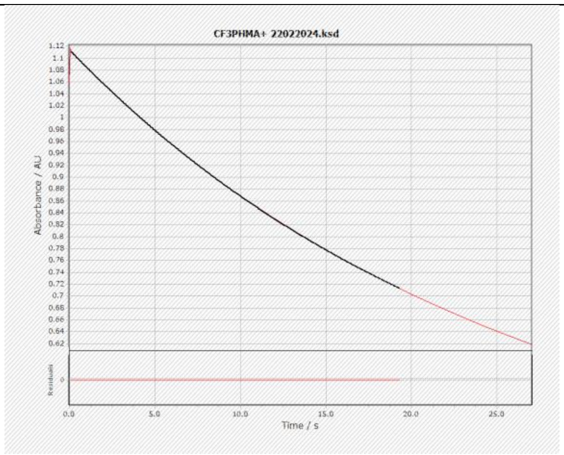

CF$_3$PhMA$^+$

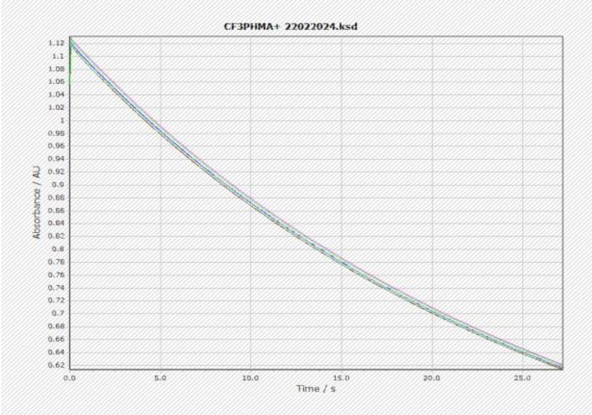

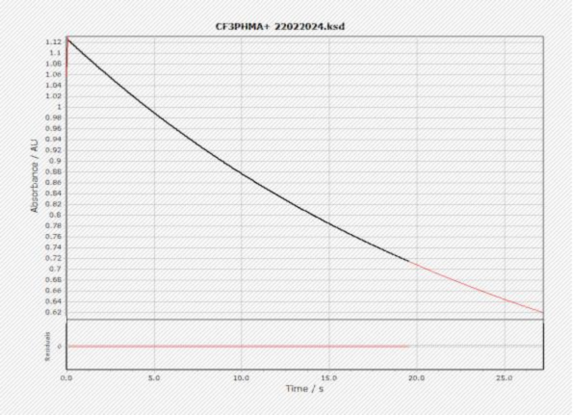

CF$_3$PhMA$^+$

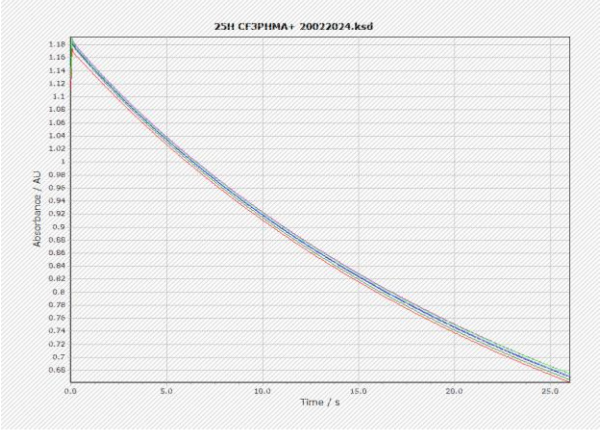

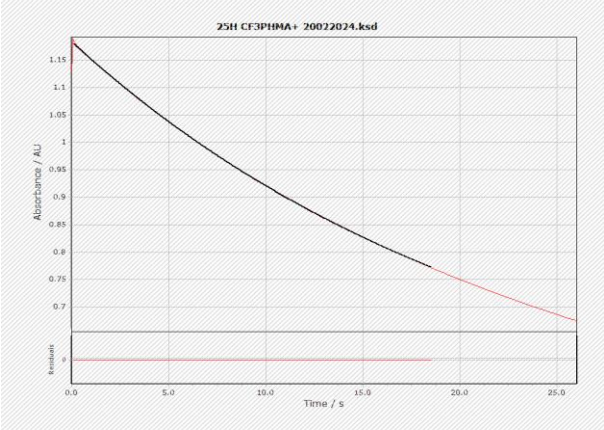

CF$_3$PhMA$^+$
